# Deconstruction of lysergic acid diethylamide

**DOI:** 10.1073/pnas.2603412123

**Published:** 2026-09-08

**Authors:** Andrian G. Basargin, Andras Domokos, Joseph J. Hennessey, Isak K. Aarrestad, Rohini Sambyal, Yara A. Khatib, Johanna Krüger, Lee E. Dunlap, Samuel J. Carter, Isabella A. Rebek, John L. McKee, Serena S. Schalk, Min Liu, James C. Fettinger, Monica A. Gonzalez, Abhay Potluri, Dean J. Tantillo, Oliver Fiehn, John D. McCorvy, David E. Olson

**Affiliations:** ^a^ Institute for Psychedelics and Neurotherapeutics, University of California, Davis, CA 95616; ^b^ Chemistry and Chemical Biology Graduate Program, University of California, Davis, CA 95616; ^c^ Department of Cell Biology, Neurobiology, and Anatomy, Medical College of Wisconsin, Milwaukee, WI 53226; ^d^ Neuroscience Graduate Program, University of California, Davis, CA 95618; ^e^ Department of Chemistry, University of California, Davis, CA 95616; ^f^ Pharmacology and Toxicology Graduate Program, University of California, Davis, CA 95616; ^g^ West Coast Metabolomics Center, University of California, Davis, CA 95616; ^h^ Department of Pharmacology and Toxicology, Neuroscience Research Center, Cancer Center, Medical College of Wisconsin, Milwaukee, WI 53226; ^i^Department of Biochemistry and Molecular Medicine, School of Medicine, University of California, Sacramento, CA 95817; ^j^ Center for Neuroscience, University of California, Davis, CA 95618

**Keywords:** lysergic acid diethylamide, psychedelic, function-oriented synthesis, LSD, 5-HT2A receptor

## Abstract

Of the classic psychedelics, lysergic acid diethylamide (LSD) is perhaps the best-known, but its complex chemical architecture has limited synthetic investigations aimed at generating analogues with improved safety and efficacy profiles. Here, we systematically deconstruct the tetracyclic ergoline core of LSD to create 9 simplified ergoline analogues (i.e., ergologs) and evaluate them in pharmacological assays relevant to 5-HT2A, 5-HT2B, and 5-HT2C receptor function. Our work revealed the key molecular features and minimal pharmacophore of LSD necessary to produce maximal agonism of 5-HT2A receptors and hallucinogenic behavioral effects. Furthermore, we established strategies for reducing the hallucinogenic and cardiotoxic potential of LSD and identified **UCD0094** and **UCD0076** as ergologs with improved safety profiles. In stark contrast to LSD, **UCD0076** exhibits preference for activating 5-HT2C receptors over other 5-HT2 receptors and produces antipsychotic-like properties in mouse behavioral assays.

## Significance

Lysergic acid diethylamide (LSD) is perhaps the best-known psychedelic and is currently being explored as a treatment for neuropsychiatric diseases. Despite being discovered over 8 decades ago, we have an incomplete understanding of how its molecular structure produces its unique pharmacological effects as structure–activity relationship studies have been hindered by the complexity of its tetracyclic ergoline core structure. Here, we apply the principles of function-oriented synthesis to systematically deconstruct the ergoline core of LSD and elucidate which of its molecular features are necessary to produce its notorious pharmacology at serotonin receptors. This information will greatly aid efforts to develop LSD analogues with improved safety and efficacy profiles.

**Fig. 1.**
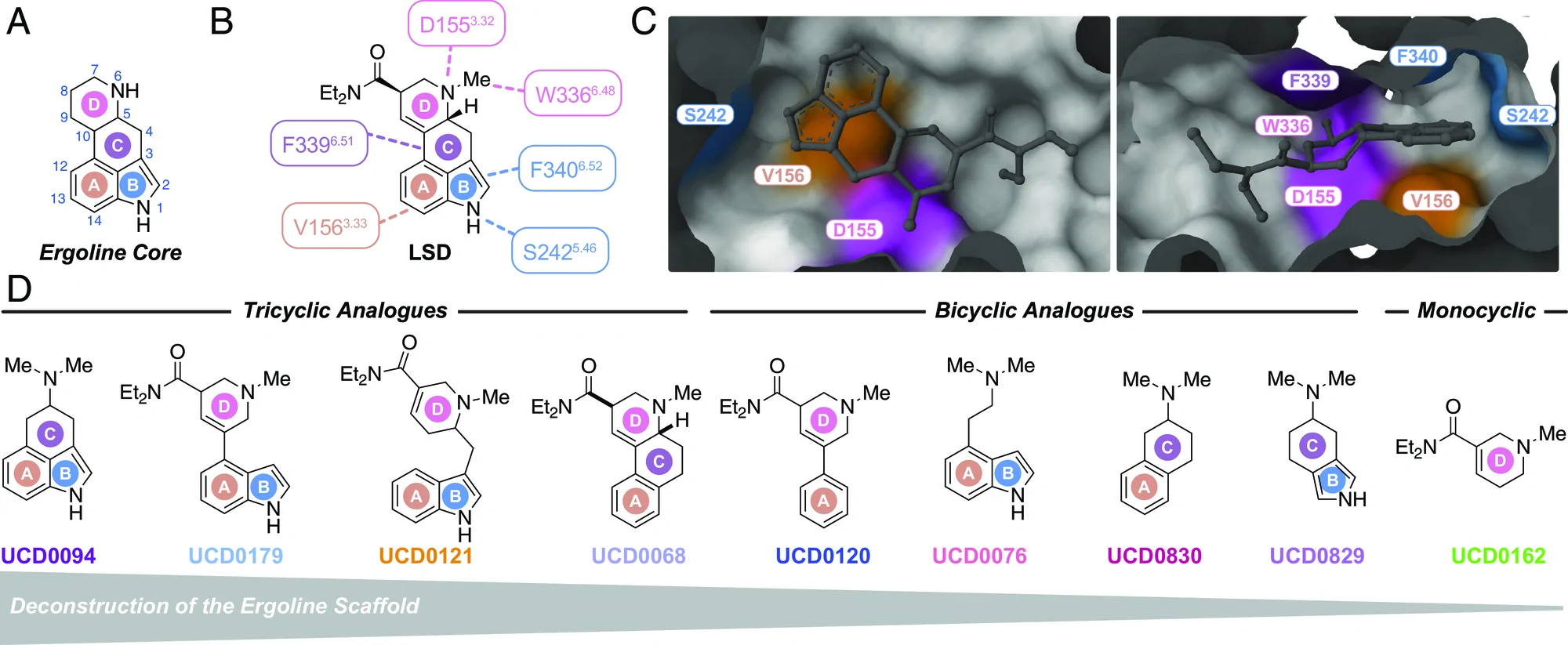
Deconstruction of LSD into simplified ergologs. (*A*) The ergoline core structure is shown with associated numbering and ring labels. (*B*) Key interactions between LSD and residues of the 5-HT2A receptor are indicated by dotted lines. Ballesteros-Weinstein numbering is indicated as superscripts. Interacting rings and residues are matched by color. (*C*) Two orientations of the X-ray crystal structure of the 5-HT2A receptor bound to LSD (PDB: 6WGT) (17) with residues interacting with specific ergoline rings matched by color (orange = A ring, blue = B ring, purple = C ring, pink = D ring). LSD is shown in gray. (*D*) Deletion of ergoline core rings results in simplified tricyclic, bicyclic, and monocyclic LSD analogues. LSD = lysergic acid diethylamide.

The ergoline core (Fig. 1*A*) is a privileged structure in medicinal chemistry (1), as this motif is found in several approved medicines and biologically active compounds (2). Perhaps the best known ergoline is lysergic acid diethylamide (LSD), a potent psychedelic that produces a variety of biological effects by interacting with a wide range of aminergic G protein–coupled receptors (GPCRs), including the 5-HT2 receptor subtypes (3–6). Agonists of 5-HT2A and 5-HT2C receptors, such as LSD, produce several therapeutic effects (7, 8) while agonism of 5-HT2B receptors is believed to increase risk for cardiac valvulopathy (9). The 5-HT2A receptor is particularly relevant to the biological effects of LSD as it is known to mediate both the hallucinogenic (10–12) and neuroplasticity-promoting (13–16) properties of psychedelics.

Two X-ray crystal structures of the 5-HT2A receptor bound to LSD have been solved (18, 19), and they indicate key interactions with the A, B, C, and D rings of the ergoline core (Fig. 1 *B* and *C*). Mutagenesis studies corroborate these findings and demonstrate that many of these interactions are important for the binding and/or efficacy of LSD(17). In particular, a salt bridge between D155^3.32^ and the basic amine of LSD is essential for receptor binding and subsequent activation, while interaction with toggle switch residue W336^6.48^ is also critical. Interestingly, the indole N–H of LSD forms a hydrogen bond with S242^5.46^, which contributes to its slow dissociation kinetics (18). While mutagenesis experiments are extremely useful and provide clues as to which residues interact with LSD, these studies cannot be used to determine the minimal pharmacophore of LSD necessary to produce its biological effects, nor can they alone inform on the design of LSD analogues with improved safety and efficacy profiles. In this regard, structural analogues of LSD can complement and extend findings from structural biology and mutagenesis studies.

Compared to tryptamine- and phenethylamine-based psychedelics, a much smaller number of LSD analogues have been generated. Most of these efforts, including those that created the nonhallucinogenic LSD analogues lisuride and 2-Br-LSD (BOL-148), have focused on modifying the periphery of the ergoline core due to its structural complexity and easy access via semisynthesis from ergot alkaloids. Though rare, modification of LSD’s ergoline core has proven fruitful, as the indolonapthyridine constitutional isomer JRT was recently shown to display potent neuroplasticity-promoting properties and therapeutic potential with an improved safety profile (20). Moreover, skeletal editing of LSD produced a quinoline derivative with lower potency/efficacy for activating the 5-HT2A receptor (21).

Given that the ergoline core structure of LSD contains both tryptamine and phenethylamine structural elements (22), we wanted to understand which components were essential for producing LSD-like pharmacological effects. To solve this problem, we employed function-oriented synthesis—a concept that involves the synthesis of simplified analogues of complex molecular architectures that retain desired biological properties (23). Function-oriented synthesis has been used previously to create useful analogues of complex natural products including bryostatin (24) and ibogaine (25). Here, we reasoned that a systematic deconstruction of LSD’s ergoline core while leaving the basic nitrogen intact (Fig. 1*D*) would enable us to determine the minimal pharmacophore responsible for its hallucinogenic effects. We profiled these ergoline analogues (i.e., ergologs) in assays relevant to 5-HT2A, 5-HT2B, and 5-HT2C receptor function and found ways to modulate the pharmacological properties of LSD.

## Results

### Synthesis of Tricyclic Ergologs.

To delete the D-ring of LSD, we synthesized tricyclic ergolog **UCD0094** from commercially available 3-indolepropionic acid **1** (*SI Appendix*, Fig. S1). First, **1** was esterified and reduced to indoline **2**. Protection of the nitrogen with pivaloyl chloride followed by hydrogenolysis yielded **3** in 84% over two steps. Activation of the acid with thionyl chloride and treatment with a Lewis acid facilitated intramolecular Friedel-Crafts acylation to produce ketone **4** in good yield (67% over 2 steps). Reduction with borohydride and acid-catalyzed dehydration yielded styrene **5**, which was epoxidized and subjected to zinc-mediated rearrangement to produce ketone **7** in excellent yield (78% over 4 steps). Reductive amination, deprotection, and DDQ-mediated oxidation of the resulting indoline furnished **UCD0094** in a total of 13 steps with 8% overall yield.

Deletion of the C-ring could be accomplished by either severing the C11–C12 bond or deleting C4. These two approaches would yield tricyclic analogues with major differences in conformational flexibility, so we decided to synthesize both. To access the more rigid analogue, we first converted 3-bromonicotinic acid **9** to the diethylamide in quantitative yield (*SI Appendix*, Fig. S2). Methylation and reduction yielded tetrahydropyridine **10**, which was cross-coupled to indole-4-boronic acid pinacol ester to furnish **UCD0179** in 4 steps total and 48% overall yield.

The more flexible tricyclic ergolog **UCD0121** proved more challenging to construct (*SI Appendix*, Fig. S3). Our synthesis began with a chemoselective reduction of commercially available diester **11**, to afford alcohol **12**. Conversion of the ester to the diethylamide followed by Swern oxidation produced aldehyde **13** in good yield (56% over 2 steps). Treating the aldehyde with indole yielded a benzylic alcohol that was reduced with Hantzsch ester under acidic conditions to afford **14**. Pyridine methylation and reduction to the tetrahydropyridine completed the synthesis of **UCD0121** in 7 steps and 22% overall yield. As expected, the olefin was observed to be in conjugation with the amide.

To construct the ergoline core lacking the B ring, we first performed an S_N_Ar reaction using commercially available nicotinic acid derivative **16** and methyl malonate (*SI Appendix*, Fig. S4). Next, we alkylated the resulting diester by adding 2-bromobenzyl bromide. In the same reaction flask, we saponified the esters with NaOH and acidified with H_2_SO_4_. Heating the reaction to 80 °C resulted in double decarboxylation to afford the methylene-containing product in 58% yield. Conversion of the acid to the diethylamide **17** followed by methylation of the pyridine and reduction yielded **18**, which was subjected to Miyaura borylation followed by in situ intramolecular Suzuki-Miyaura cross-coupling to afford **UCD0068** in 5 steps total and 11% overall yield. The relative stereochemistry of **UCD0068** was confirmed by a combination of NMR experiments (DEPT-135, HSQC, COSY, 2D NOESY, 1D NOESY, *SI Appendix*, Fig. S4).

### Synthesis of Bicyclic Ergologs.

Ergologs containing only 2 of the 4 core ergoline rings were much more straightforward to prepare. We synthesized **UCD0120** in 4 steps and 58% overall yield by first methylating and reducing the diethylamide of nicotinic acid to the tetrahydropyridine **10**, which was subjected to Pd-catalyzed cross-coupling conditions in the presence of phenylboronic acid (*SI Appendix*, Fig. S5). Similar cross-coupling conditions were used to prepare **21** from pinacol boronate **19** and α-halo amide **20**. Reduction with lithium aluminum hydride yielded **UCD0076** in 2 steps and 33% overall yield (*SI Appendix*, Fig. S6).

Two bicyclic ergologs containing the C ring were envisioned. The first, **UCD0830**, bore the A/C ring fusion and was readily prepared in a single step by subjecting 2-tetralone **22** to reductive amination conditions with dimethylamine (*SI Appendix*, Fig. S7). In contrast, the synthesis of **UCD0829** was much more challenging as it required a multistep sequence to install the B/C ring fusion (*SI Appendix*, Fig. S8). First, commercially available anhydride **23** was reduced to the diol **24**, which was oxidized to the dialdehyde under Swern conditions and trapped as the dimethoxytetrahydrofuran **25**. Treatment of **25** with CbzNH_2_ and a dehydrating agent produced Cbz-protected pyrrole **26**, which was subjected to hydroboration/oxidation to yield alcohol **27**. Oxidation to the ketone followed by reductive amination with dimethylamine and pyrrole deprotection produced **UCD0829** in 7 steps total and 4% overall yield.

### Synthesis of a Monocyclic Ergolog.

Synthesis of a monocyclic ergolog lacking the A, B, and C rings of the ergoline core began with treating *N,N*-diethylnicotinamide **30** with MeI (*SI Appendix*, Fig. S9). The resulting pyridinium **31** was reduced to the tetrahydropyridine with NaCNBH_3_. The synthesis of **UCD0162** was completed in only 2 steps with 32% overall yield. The lack of the A ring precludes styrene formation, and as expected, the olefin was observed to be in conjugation with the amide.

### Ergologs have Reduced Affinity for the 5-HT2A Receptor.

**Fig. 2.**
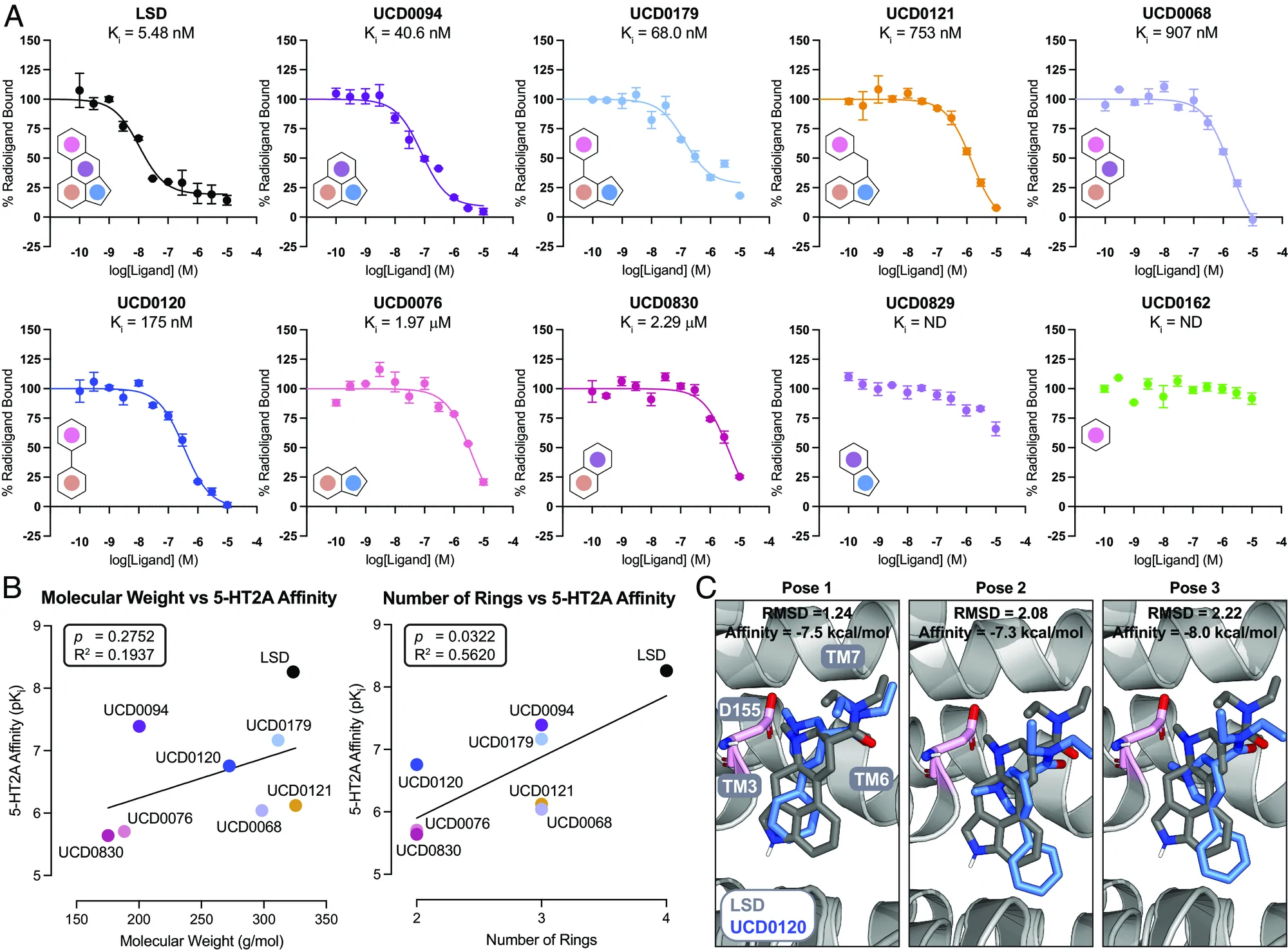
Binding affinity for the psychLight2 5-HT2A receptor. (*A*) Radioligand competition binding assays using membrane preparations from PSYLI2 cells (i.e., HEK293T cells stably expressing the 5-HT2A-based biosensor psychLight2) were performed to determine K_i_ values. Data represent the mean of well averages from 2 to 3 experimental replicates (i.e., independent batches of membrane preparations) ± SEM; N = 3 for experimental compounds, N = 2 for LSD. K_i_ values were not determined (ND) for compounds that did not produce at least 75% inhibition at 10 μM. Schematics indicate structural components of the ergoline core present in each experimental compound. (*B*) Simple linear regression demonstrates that affinity for the psychLight2 5-HT2A receptor correlates with the number of ergoline core rings but not the molecular weights of the freebases. Given that **UCD0829** and **UCD0162** displayed minimal to no affinity for the psychLight2 5-HT2A receptor, they were excluded from the analysis. (*C*) Four conformations of **UCD0120** (blue) were docked into the X-ray crystal structure of the 5-HT2A receptor (PDB: 9AS4) bound to LSD (gray) to create 36 poses. The three poses with the lowest RMSD relative to LSD are shown. A key aspartate residue (D155) is highlighted in pink. LSD = lysergic acid diethylamide.

Given the importance of the 5-HT2A receptor in mediating the biological effects of LSD, we performed radioligand competition binding studies using [^3^H]LSD as the radiotracer to determine the relative affinities of ergologs for psychLight2—a 5-HT2A-based biosensor (Fig. 2*A* and *SI Appendix*, Table S1). Though psychLight2 contains a circularly permuted green fluorescent protein within intracellular loop 3 and would not be predicted to couple strongly to Gq, the binding pocket of psychLight2 is identical to native 5-HT2A receptors (26), and we have previously demonstrated that there are no differences in binding affinities between psychLight2 and native 5-HT2A receptors for 5-HT or 5-MeO-DMT (13). Every ergolog tested displayed lower affinity for the psychLight2 5-HT2A receptor than LSD, with affinities ranging from low nM to >10 μM. Given that entropic driving forces can contribute greatly to binding (27), we explored whether binding affinities correlated with compound molecular weights. However, we only observed a weak, nonsignificant correlation (R^2^ = 0.2, *P* = 0.3, Fig. 2*B*). By contrast, we observed a strong, significant correlation between psychLight2 5-HT2A receptor affinity and number of core ergoline rings present in the ergolog (R^2^ = 0.6, *P* = 0.03, Fig. 2*B*), indicating that specific ergolog conformations and interactions between the rings of the ergoline core and the protein are likely factors driving affinity beyond simple desolvation and release of water upon binding.

In general, tricyclic analogues retained high affinity for the psychLight2 5-HT2A receptor (i.e., nanomolar range), with the ergologs containing the ABC and ABD ring systems exhibiting the greatest binding. Compared to the structurally rigidified ABD ring-containing ergolog **UCD0179**, the binding affinity of the highly flexible ABD ring-containing ergolog **UCD0121** was an order of magnitude lower. Tricyclic ergolog **UCD0068** lacking the B-ring had the lowest affinity for the psychLight2 5-HT2A receptor (K_i_ = 890 nM), which was surprising given that previous mutagenesis studies indicated that the H-bond between the indole N–H of LSD and the 5-HT2A receptor contributed minimally to its binding affinity (18).

In sharp contrast to the tricyclic ergologs, most bicyclic ergologs (i.e., **UCD0076**, **UCD0830**, **UCD0829**) and the monocyclic ergolog (i.e **UCD0162**) exhibited minimal (K_i_ = ~2 μM) to no affinity for the psychLight2 5-HT2A receptor, which was surprising given that **UCD0162** (10 μM) had been previously shown to block the binding of LSD to synaptosomal membranes (28). The only bicyclic analogue that retained nanomolar affinity was **UCD0120** (K_i_ = 175 nM), indicating that the A-ring of the ergoline core is essential to maintain high affinity for the 5-HT2A receptor.

To better understand how ergologs interact with the 5-HT2A receptor, we first calculated their most stable conformations in aqueous solution (*SI Appendix*, Fig. S10). Next, we docked the ergologs into an active state X-ray structure of the 5-HT2A receptor bound to LSD (PDB: 9AS4) (*SI Appendix*, Fig. S11). Given that D155^3.32^ is essential for ligand binding (29), we deemed poses valid if they positioned the protonated nitrogen of the ligand within 4 Å of the carbonyl oxygen of D155^3.32^. As expected, most ergologs adopted conformations in the receptor binding pocket that were distinct from their lowest energy conformations in solution. Moreover, the poses predicted to yield the highest affinities also tended to align well with the binding pose of LSD. Despite only possessing two rings, **UCD0120** yielded 3 poses with predicted affinities ranging from −7.3 to −8.0 kcal/mol and root mean square deviations (RMSDs) from LSD ranging from 1.24 to 2.22 Å (Fig. 2*C*). The A-ring of these docked structures maintained the hydrophobic and edge-to-face π-stacking interactions with V156^3.33^ and F340^6.52^, respectively, that were observed in the crystal structure of LSD bound to the 5-HT2A receptor.

### Functional Effects of Ergologs at 5-HT2 Receptors.

**Fig. 3.**
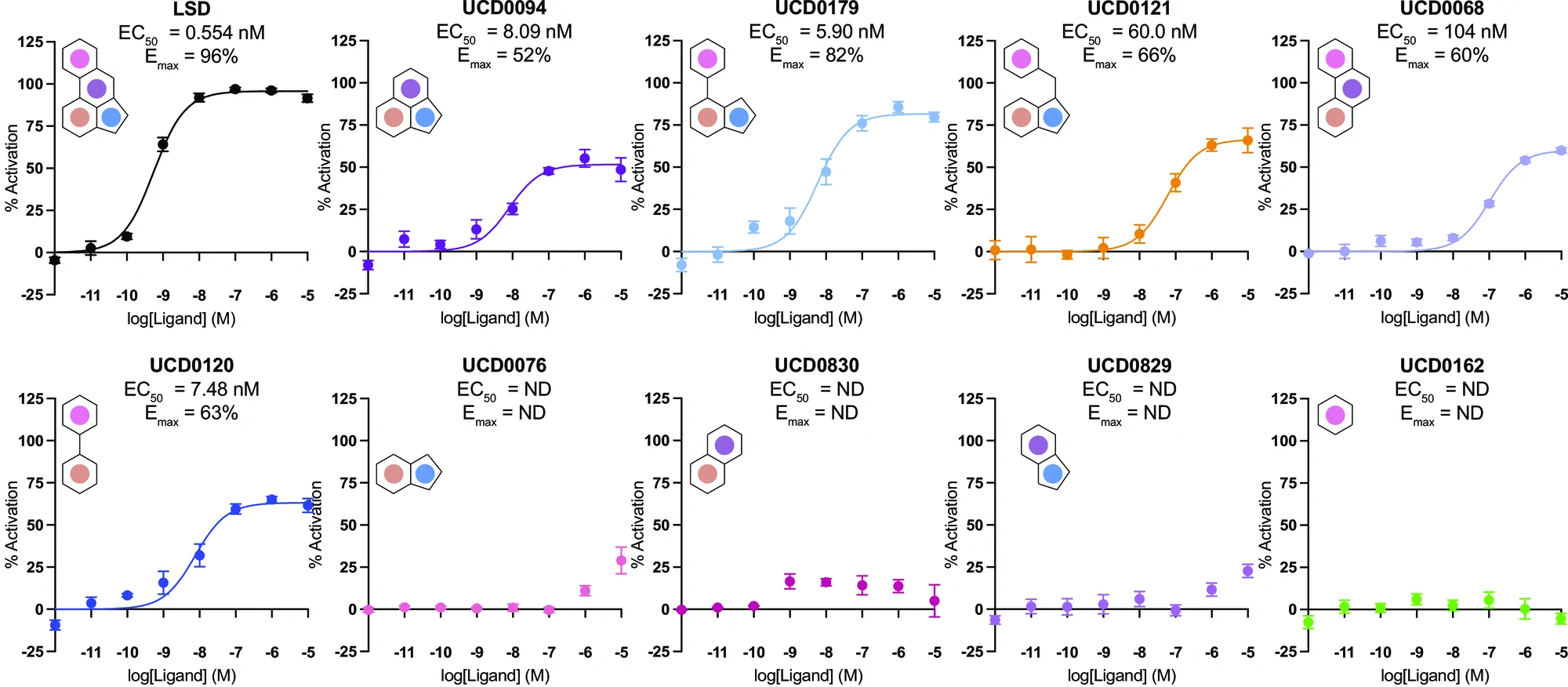
BRET-based assays of Gq activation following 5-HT2A receptor stimulation. Potency (EC_50_) and efficacy (E_max_) of Gq activation was assessed using BRET-based methods in HEK293T cells transiently expressing 5-HT2A receptors. Data represent 2 wells from 3 to 6 experimental replicates (i.e., independent cultures) and are normalized to the serotonin response (5-HT E_max_ = 100%; 5-HT EC_50_ = 3.5 nM) with the bottom of the curves constrained to 0. N = 12 for **UCD0076**, N = 6 for all other compounds. EC_50_ and E_max_ values were not determined (ND) for compounds that did not produce at least 30% activation at 10 μM. Schematics indicate structural components of the ergoline core present in each experimental compound. LSD = lysergic acid diethylamide.

Changes in binding affinity cannot predict changes in receptor activation, so we next performed concentration–response studies using a BRET-based assay of Gq activation following stimulation of the 5-HT2A receptor (5, 30) (Fig. 3 and *SI Appendix*, Table S2). The potencies of **UCD0094**, **UCD0179**, and **UCD0121** mirrored their affinities, but **UCD0068** and **UCD0120** were more potent than their affinity values would belie. With the exception of **UCD0120**, all other bicyclic and monocyclic ergologs were inactive in agonist mode assays of 5-HT2A receptor-dependent Gq activation.

Despite being relatively potent (EC_50_ = 6 to 104 nM), all ergologs exhibited substantially reduced maximal Gq-efficacy compared to LSD (E_max_ = 96%), suggesting that an intact tetracyclic ergoline core is essential for producing maximal efficacy at the 5-HT2A receptor. Notably, the D-ring appeared to be the essential component for maintaining high efficacy (i.e., E_max_ > 60%), as the E_max_ of ABC ring-containing analogue **UCD0094** was only 52%. The bicyclic analogues exhibiting μM affinity for the 5-HT2A receptor (i.e., **UCD0076** and **UCD0830**) did not display any evidence of agonism. To ensure ergologs did not exhibit strong biased agonism, we tested a subset of them in β-arrestin2 recruitment assays (*SI Appendix*, Table S3) and found that their abilities to induce β-arrestin2 recruitment mirrored their abilities to activate Gq, albeit with slightly lower potencies/efficacies.

**Fig. 4.**
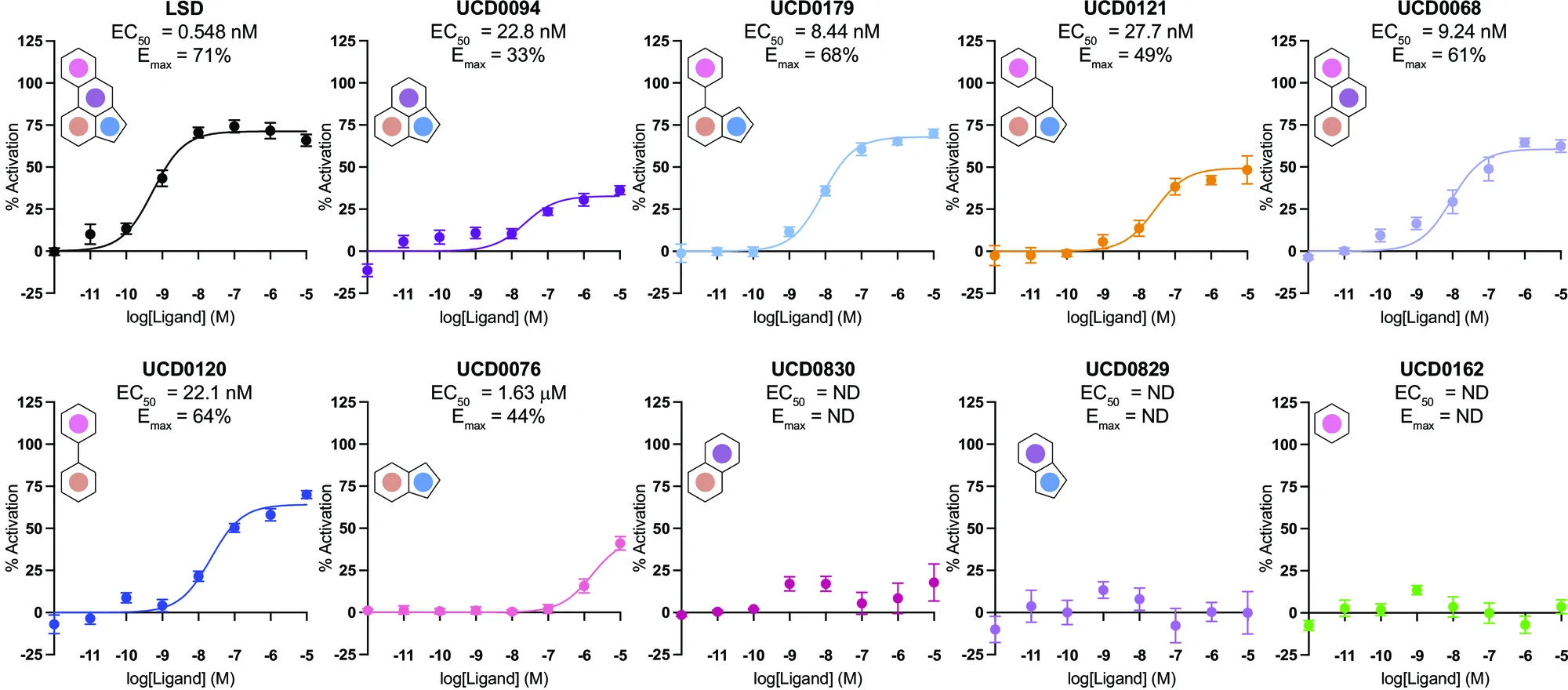
BRET-based assays of Gq activation following 5-HT2B receptor stimulation. Potency (EC_50_) and efficacy (E_max_) of Gq activation was assessed using BRET-based methods in HEK293T cells transiently expressing 5-HT2B receptors. Data represent 2 wells from 3 to 6 experimental replicates (i.e., independent cultures) and are normalized to the serotonin response (5-HT E_max_ = 100%; 5-HT EC_50_ = 0.52 nM) with the bottom of the curves constrained to 0. N = 22 for **UCD0076**, N = 10 for **UCD0121**, **UCD0094**, and **UCD0068**, N = 16 for **UCD0179**, N = 6 for all other compounds. EC_50_ and E_max_ values were not determined (ND) for compounds that did not produce at least 30% activation at 10 μM. Schematics indicate structural components of the ergoline core present in each experimental compound. LSD = lysergic acid diethylamide.

**Fig. 5.**
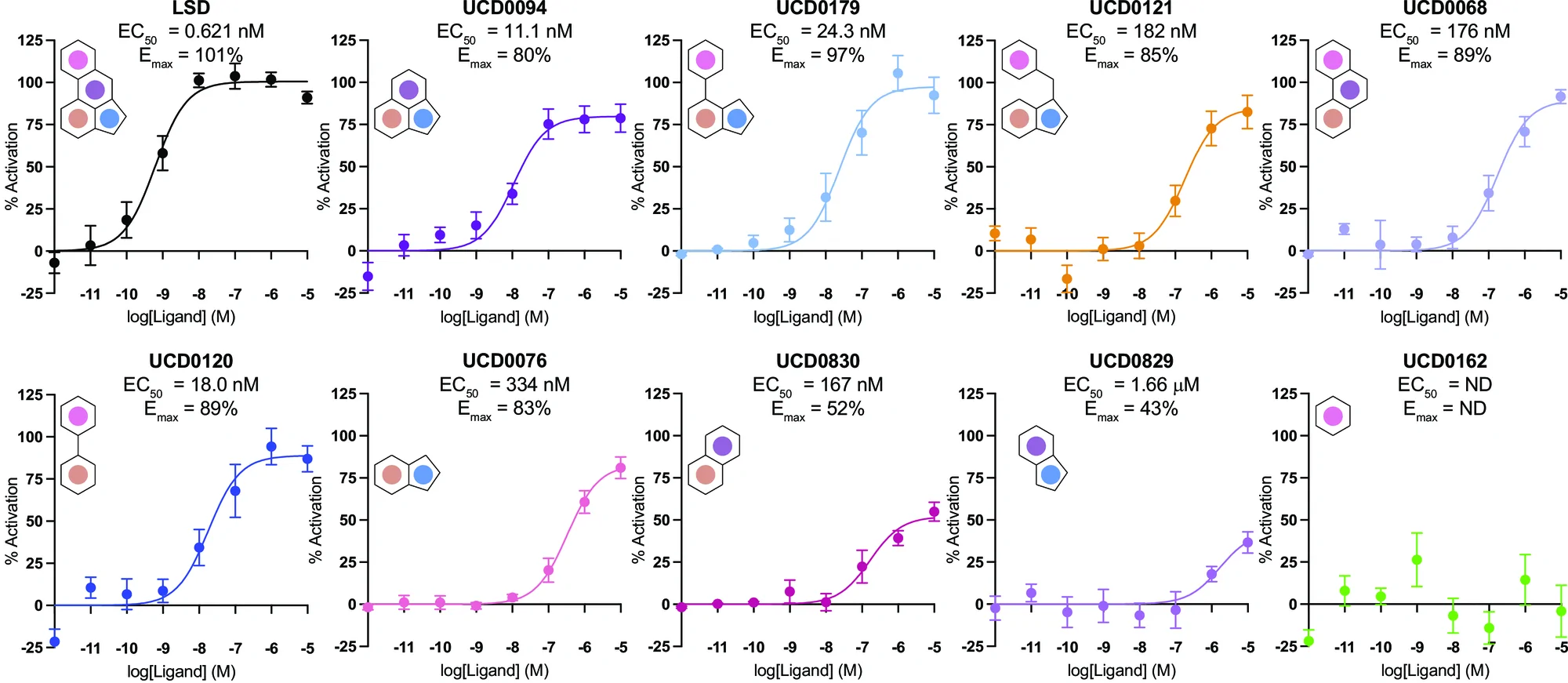
BRET-based assays of Gq activation following 5-HT2C receptor stimulation. Potency (EC_50_) and efficacy (E_max_) of Gq activation was assessed using BRET-based methods in HEK293T cells transiently expressing 5-HT2C receptors. Data represent 2 wells from 3 to 6 experimental replicates (i.e., independent cultures) and are normalized to the serotonin response (5-HT E_max_ = 100%; 5-HT EC_50_ = 0.33 nM) with the bottom of the curves constrained to 0. N = 12 for **UCD0076**, N = 6 for all other compounds. EC_50_ and E_max_ values were not determined (ND) for compounds that did not produce at least 30% activation at 10 μM. Schematics indicate structural components of the ergoline core present in each experimental compound. LSD = lysergic acid diethylamide.

Given the high homology of the 5-HT2A receptor ligand binding domain with those of the 5-HT2B and 5-HT2C receptors (31), the vast majority of compounds that bind to 5-HT2A receptors are also ligands for 5-HT2B and 5-HT2C receptors. To assess the functional effects of ergologs at the latter two receptors, we performed analogous BRET-based assays of Gq activation following their stimulation (Figs. 4 and 5 and *SI Appendix*, Table S2). We found that rigid compounds containing the AD rings (i.e., **UCD0179**, **UCD0068**, and **UCD0120**) were nearly as efficacious as LSD for activating the 5-HT2B receptor (E_max_ = 71% for LSD compared to 49 to 68% for the ergologs). In stark contrast, **UCD0094** exhibited drastically reduced maximal activation (E_max_ = 33%).

While **UCD0094** did not strongly activate 5-HT2A or 5-HT2B receptors, it did activate 5-HT2C receptors with a maximal efficacy of 80%. In fact, with the exception of the monocyclic analogue **UCD0162**, all ergologs exhibited agonist activity at the 5-HT2C receptor (Fig. 5), with many of them producing high maximal activity (E_max_ > 80%). Thus, it appears that the 5-HT2C receptor is less sensitive to ergoline core ring deletion than either the 5-HT2A or 5-HT2B receptor.

**Fig. 6.**
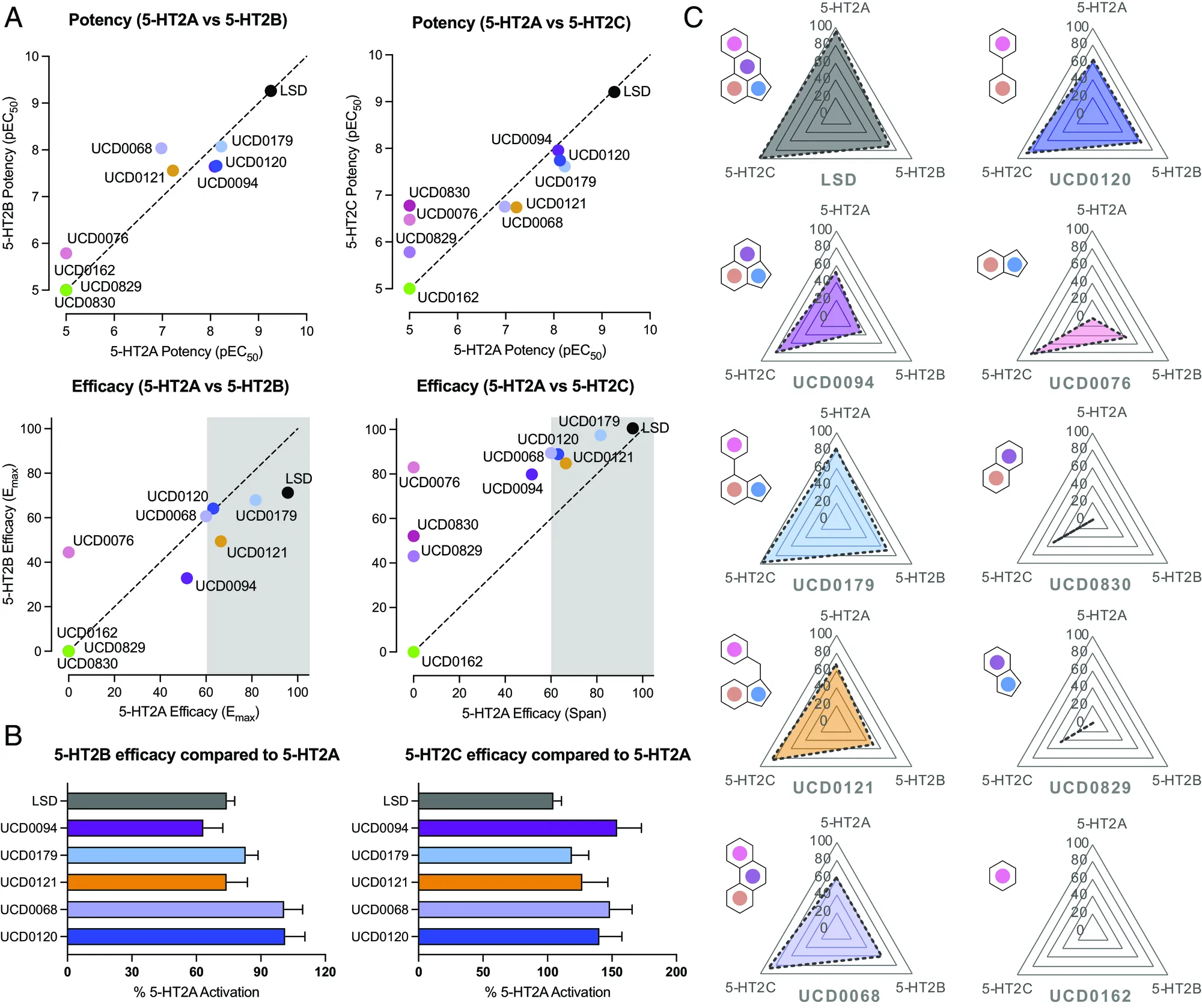
Ergologs exhibit changes in 5-HT2 receptor pharmacology relative to LSD. (*A*) Correlations between 5-HT2A receptor potencies (*Top*) and efficacies (*Bottom*) in BRET-based assays of Gq activation with analogous assays of 5-HT2B (*Left*) and 5-HT2C (*Right*) receptor activation. Data represent mean values. The dotted lines indicate unity. Approximate levels of 5-HT2A receptor activation hypothesized to be necessary for inducing hallucinogenic effects (60 to 100% E_max_) are shown in gray. Pharmacodynamic parameters that could not be determined due to a lack of a concentration–response were set to pKi = 5 or E_max_ = 0%. (*B*) The efficacy (E_max_) of each compound for activating 5-HT2B (*Left*) and 5-HT2C (*Right*) receptors was expressed as a percentage of their maximum 5-HT2A receptor efficacy. Data represent 2 wells from 3 experimental replicates (i.e., independent cultures) and are normalized to the serotonin response (5-HT E_max_ = 100%) with the bottom of the curves constrained to 0; N = 6 for all compounds. There were no statistical differences between LSD and the experimental compounds (one-way ANOVA with Dunnett’s post hoc test; *Left*, F(5, 30) = 2.232, *P* = 0.0769; *Right*, F(5, 30) = 0.9920, *P* = 0.4392). (*C*) Radar graphs indicate relative efficacy (E_max_) across 5-HT2 receptors. Data represent mean values. Schematics indicate structural components of the ergoline core present in each experimental compound. LSD = lysergic acid diethylamide.

To compare the relative impact of ergoline ring deletion on 5-HT2 receptor activation, we plotted the potency and efficacy of 5-HT2B and 5-HT2C activation as a function of 5-HT2A activation (Fig. 6*A*). In general, loss of 5-HT2A receptor potency resulted in a comparable loss in potency across 5-HT2B and 5-HT2C receptors. However, **UCD0076** and **UCD0830** maintained high potency for 5-HT2C receptor activation without activating 5-HT2A receptors.

While changes in potency seemed to be comparable across 5-HT2 receptors, changes in efficacy were not. For all compounds with agonist activity at 5-HT2A receptors, we plotted their maximal activations of 5-HT2B and 5-HT2C receptors as a percentage of their maximal 5-HT2A receptor activations (Fig. 6*B*). Though not statistically significant compared to LSD, most ergologs exhibited greater activation of 5-HT2B and 5-HT2C receptors compared to 5-HT2A. This trend was particularly obvious for 5-HT2C receptors, as all ergologs exhibited greater efficacy at 5-HT2C than 5-HT2A receptors (Fig. 6*A*). For those that agonized 5-HT2A receptors, their activities at 5-HT2C receptors were ~1.2 to 1.6-fold higher (Fig. 6*B*). This shift toward 5-HT2C receptor activation was apparent in radar plots of ergolog activity (Fig. 6*C*), and we identified **UCD0076** as selective for 5-HT2C receptor activation compared to 5-HT2A and 5-HT2B receptors.

### The Effects of UCD0094 are Stereospecific.

Previous work has demonstrated that only the (+)-enantiomer of LSD produces psychoactive effects and that *R*-stereochemistry at C5 is essential for ergoline-containing compounds to bind to 5-HT2 receptors with high affinities (32). To determine if this stereospecificity applies to simplified ergologs, we first developed an asymmetric synthesis of **UCD0094** (*SI Appendix*, Fig. S12)—the C5-containing ergolog with the greatest potency for activating 5-HT2 receptors. First, we treated epoxide **6** with (1*R*)-(+)-phenylethylamine to produce a separable mixture of the alcohol-containing diastereomers **32** and **33**. Treatment of the purified diastereomers with MsCl and base produced the respective aziridines. A one-pot hydrogenolysis, reductive ring opening, and reductive amination sequence furnished **36** and **40**, though incomplete deprotection also yielded **35** and **39**. These byproducts were separated and subjected to ammonium formate and formaldehyde under acid conditions to increase the yields of **36** and **40** prior to deprotection with *n*-BuLi to produce **37** and **41**. Absolute stereochemistry was confirmed for **37** and **41** using X-ray crystallography. Finally, oxidation of the indolines to the indoles yielded the target compounds **(+)-UCD0094** and **(–)-UCD0094**, which were judged to be enantiomerically pure based on chiral HPLC and optical rotation.

As expected, **(+)-UCD0094** exhibited an affinity for psychLigth2 that was nearly 2-orders of magnitude greater than **(–)-UCD0094** (*SI Appendix*, Fig. S13 and Table S1). This higher affinity also translated into increased potency for activating Gq following 5-HT2A receptor stimulation (*SI Appendix*, Table S2). As observed in BRET-based assays of 5-HT2A receptor activation, **(+)-UCD0094** was also a more potent 5-HT2C agonist (*SI Appendix*, Fig. S13). However, the enantiomers were equipotent in BRET-based assays of 5-HT2B receptor activation (*SI Appendix*, Fig. S13). The enantiomers exhibited comparable efficacies to each other at 5-HT2A, 5-HT2B, and 5-HT2C receptors. Thus, we conclude that the effects of the racemates at 5-HT2 receptors are likely dominated by the C5 *R*-epimers, as is the case for LSD and the indolonapthyridine analogue JRT (20, 32).

### Minimal Pharmacophore for Inducing Hallucinations.

**Fig. 7.**
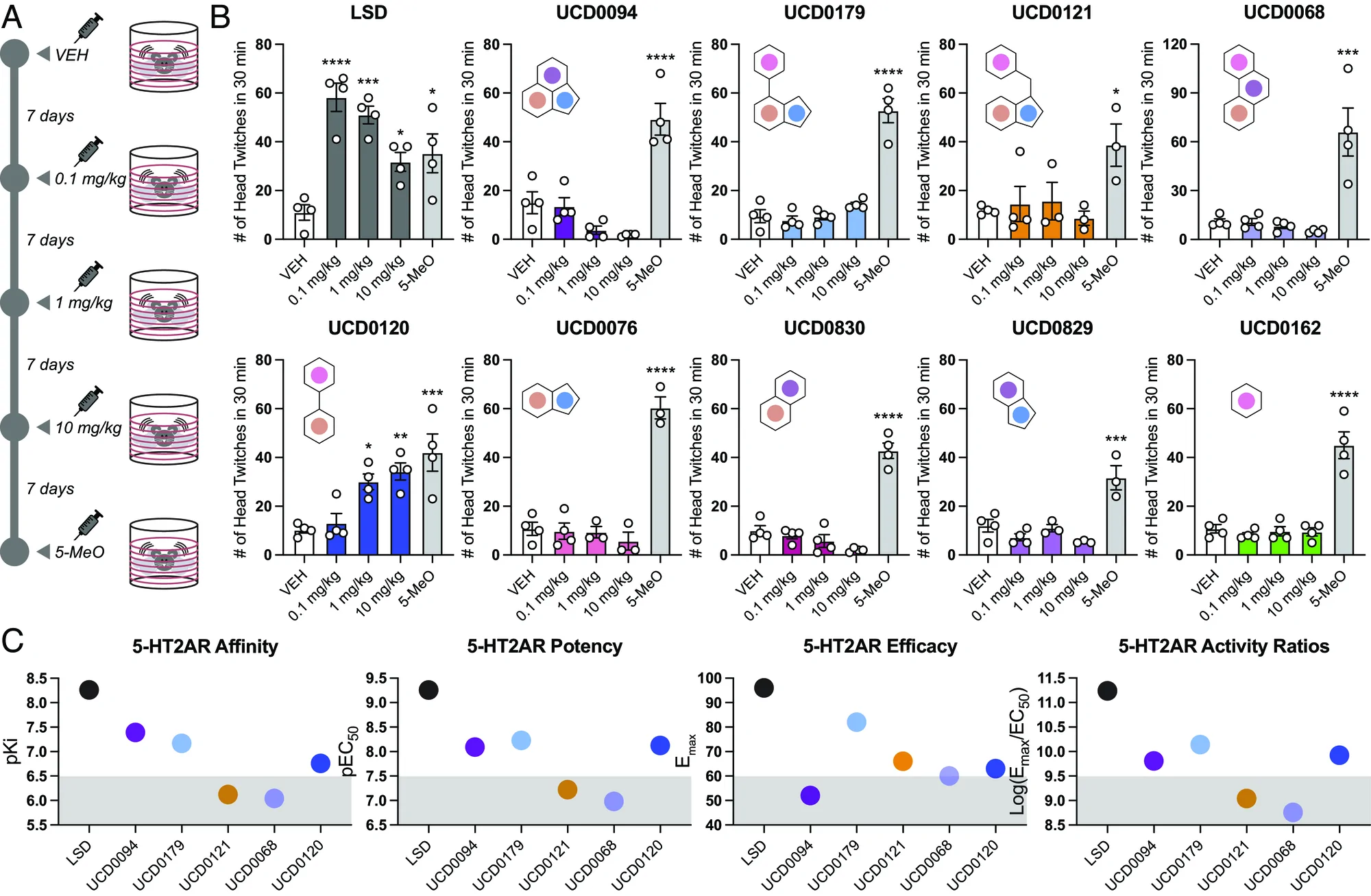
Hallucinogenic potential of ergologs. (*A*) Schematic showing experimental design. Compounds were assessed in a magnetometer-based automated HTR assay with VEH being administered during the first week and ascending doses being given subsequently spaced 1 wk apart. A week following the last dose, 5-MeO (10 mg/kg) was given to ensure that tachyphylaxis had not occurred. (*B*) Dose–response studies indicate that only LSD and **UCD0120** induce a HTR. Data represent mean ± SEM. N = 3 to 4 female mice per group. LSD: N = 4 mice per dose; **UCD0094**: N = 4 mice per dose; **UCD0179**: N = 4 mice per dose; **UCD0121**: N = 4 mice for VEH and 0.1 mg/kg dose, N = 3 for 1 mg/kg dose, 10 mg/kg dose, and 5-MeO; **UCD0068**: N = 4 mice per dose; **UCD0120**: N = 4 mice per dose; **UCD0076**: N = 4 mice for VEH and 0.1 mg/kg dose, N = 3 for 1 mg/kg dose, 10 mg/kg dose, and 5-MeO; **UCD0830**: N = 4 mice per dose; **UCD0829**: N = 4 mice for VEH and 0.1 mg/kg dose, N = 3 for 1 mg/kg dose, 10 mg/kg dose, and 5-MeO; **UCD0162**: N = 4 mice per dose. *P < 0.05, **P < 0.01, ***P < 0.001, ****P < 0.0001 compared to VEH, calculated using a one-way ANOVA with Dunnett’s post hoc test. (*C*) Comparisons of affinities, potencies, efficacies, and activity ratios at the 5-HT2A receptor. Gray shading indicates hypothesized thresholds that must be achieved to produce hallucinogenic effects. VEH = vehicle, LSD = lysergic acid diethylamide, 5-MeO = 5-methoxy-*N,N*-dimethyltryptamine.

The mouse head-twitch response (HTR) is a characteristic behavioral effect of serotonergic psychedelics (33) that correlates exceptionally well with human hallucinogenic potency (34). Previous studies have suggested that a threshold of ~60 to 70% Gq activation is necessary for 5-HT2A receptor agonists to produce a HTR (35). To test this hypothesis, we subjected all ergologs to a magnetometer-based assay capable of automatically detecting head-twitches in an unbiased manner (36, 37). Increasing doses of each compound were administered following 1-wk washout periods between doses (Fig. 7*A*). Previous studies have shown that tachyphylaxis is not typically observed in the HTR after a 1-wk washout period (34, 38–40). To confirm that this was the case, we administered 5-MeO-DMT (10 mg/kg) at the end of each dose escalation study (Fig. 7*B*).

Unlike LSD, most of the ergologs tested did not produce a HTR (Fig. 7*B*). Based on their abilities to activate Gq following 5-HT2A receptor stimulation, we expected that 4 ergologs (i.e., **UCD0179**, **UCD0121**, **UCD0068**, and **UCD0120**) would produce a HTR, as they all produced E_max_ values of ~60% or greater (Fig. 3). However, **UCD0120** was the only compound to induce a HTR (Fig. 7*B*). To better understand the source of this discrepancy, we compared their affinities (pKi), potencies (pEC_50_), efficacies (E_max_), and relative activity ratios [log(E_max_)/EC_50_)] at the 5-HT2A receptor (Fig. 7*C*), which revealed that **UCD0121** and **UCD0068** both exhibited low affinity (pK_i_ < 6.5), potency (pEC_50_ < 7.5), and relative activity ratios [log(E_max_/EC_50_) < 9.5]. Thus, we conclude that in addition to producing E_max_ values of >60 to 70%, compounds in this chemical series may need to exceed a potency threshold at the 5-HT2A receptor to induce a HTR.

Although, in vitro studies indicate that **UCD0179** is a potent (EC_50_ = 5.90 nM) and efficacious (E_max_ = 82%) agonist of 5-HT2A receptors (Fig. 3), it failed to produce a head-twitch response in mice (Fig. 7*B*). We suspected that its lack of in vivo activity might be due to pharmacokinetic factors and low brain penetrance. Unlike **UCD0120**, a compound that does produce a HTR, **UCD0179** contains a C2/C3 unsubstituted indole. These positions exhibit high chemical reactivity for both electrophiles and oxidants, and cytochrome P450s are known to catalyze the oxidation of indole at C2 and C3 (41). First, we confirmed that **UCD0179** does not readily degrade in vitro, even when stored as a DMSO stock solution at room temperature for a week (*SI Appendix*, Fig. S14*A*). Next, we attempted to block a psychedelic-induced HTR by pretreating mice with **UCD0179** (10 mg/kg) 15 min prior to administering 5-MeO (10 mg/kg). We did not observe a reduction in the 5-MeO-induced HTR (*SI Appendix*, Fig. S14*B*), leading us to hypothesize that **UCD0179** does not reach sufficient brain concentrations to impact HTR behavior. To confirm this, we first analyzed the HTR time course for **UCD0120** and **UCD0179** and found that the majority of **UCD0120**-induced head-twitches occurred within the first 15 min post administration (*SI Appendix*, Fig. S14*C*). Thus, we performed pharmacokinetic studies at 2, 5, 15, and 30 min following IP administration of **UCD0179** (10 mg/kg) or **UCD0120** (10 mg/kg). We found that the brain concentration of **UCD0120** was ~fivefold to sevenfold higher than that of **UCD0179** during the early time points (i.e., 0 to 5 min) when the **UCD0120**-induced HTR was highest (*SI Appendix*, Fig. S14*D*). In the brain, **UCD0120** reached a maximum concentration quite rapidly (within 5 min), while the concentration of **UCD0179** rose more gradually over time and was always substantially lower in comparison. Thus, it is likely that **UCD0179** does not induce a HTR because it does not rapidly achieve a sufficiently high brain concentration. Given that **UCD0120** elicits a HTR, we conclude that the A and D rings of the ergoline core structure represent the minimal pharmacophore of LSD required to induce hallucinogenic effects.

### UCD0076 Is an Ergolog with Antipsychotic Properties.

**Fig. 8.**
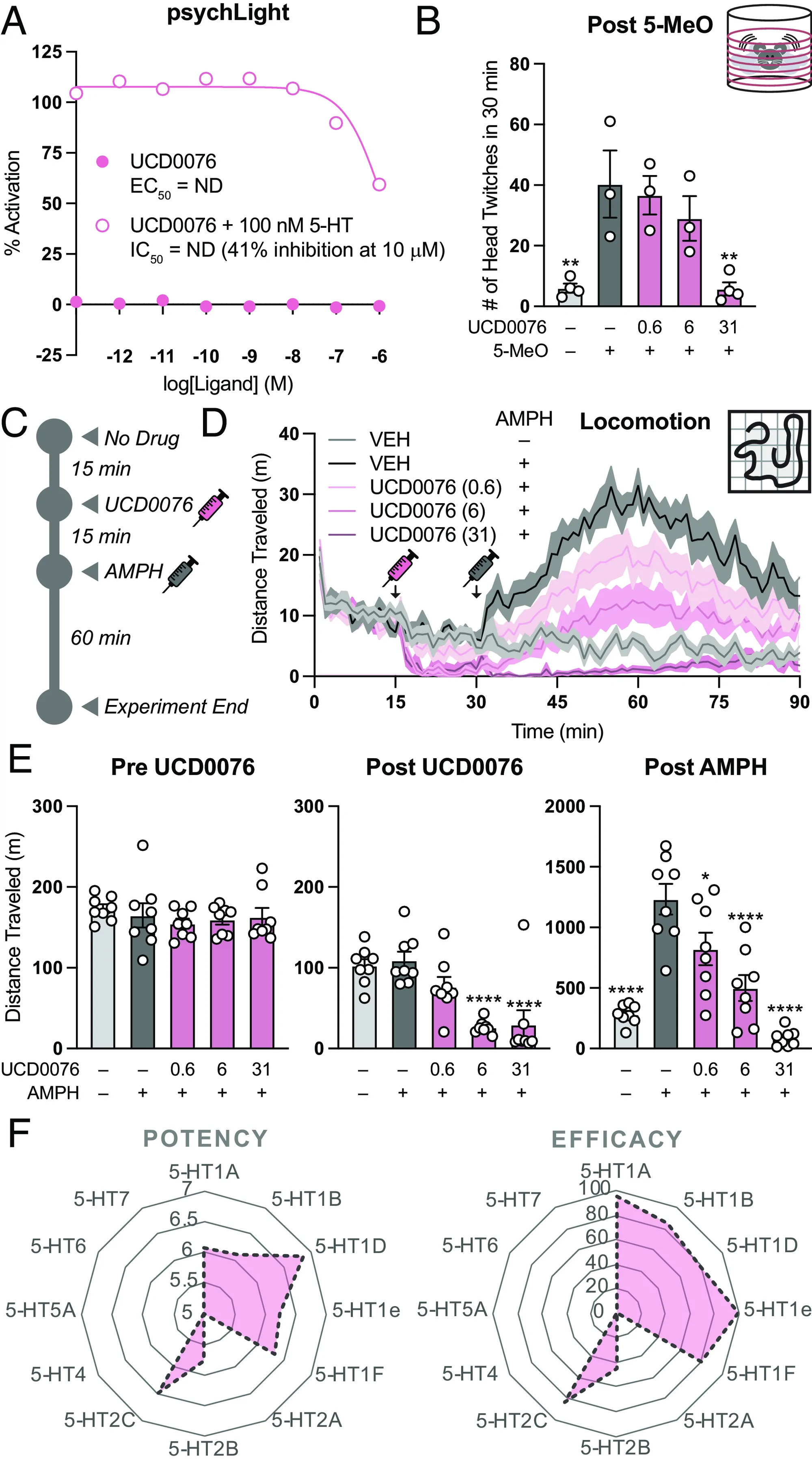
UCD0076 exhibits antipsychotic-like properties. (*A*) In psychLight2 assays, **UCD0076** does not act as an agonist and is a weak antagonist. Data represent the mean of well averages from 3 to 4 experimental replicates (i.e., independent cultures) ± SEM and are normalized to the serotonin response (5-HT E_Max_ = 100%); N = 3 for **UCD0076**, N = 4 for **UCD0076** + 100 nM 5-HT. (*B*) **UCD0076** can block a HTR induced by 5-MeO (10 mg/kg). Data represent mean ± SEM. VEH/VEH: N = 4 mice (3 male, 1 female); VEH/5-MeO: N = 3 mice (2 male, 1 female); **UCD0076** (0.6 mg/kg)/5-MeO: N = 3 mice (1 male, 2 female); **UCD0076** (6 mg/kg)/5-MeO: N = 3 mice (2 male, 1 female); **UCD0076** (31 mg/kg)/5-MeO: N = 4 mice (2 male, 2 female). **P < 0.01 compared to VEH + 5-MeO, calculated using a one-way ANOVA with Dunnett’s post hoc test. (*C*) Schematic for AMPH-induced hyperlocomotion assays. (*D*) **UCD0076** dose-dependently decreases AMPH-induced hyperlocomotion. (*E*) Locomotion was quantified before drug was given, after **UCD0076** was administered, and after the AMPH stimulus. Data represent mean ± SEM. N = 4 mice per group (2 male and 2 female mice per group). *P < 0.05, ****P < 0.0001 compared to VEH + AMPH, calculated using a one-way ANOVA with Dunnett’s post hoc test. (*F*) The selectivity of **UCD0076** was assessed by comparing potencies (pEC_50_, *Left*) and efficacies (E_max_, *Right*) of Gq activation using BRET-based methods in HEK293T cells transiently expressing 5-HT receptors. Data represent 2 wells from 3 to 6 experimental replicates (i.e., independent cultures) and are normalized to the serotonin response with the bottom of the curves constrained to 0. N = 12 for **UCD0076**, N = 6 for all other compounds. The potencies/efficacies were too low to determine accurate EC_50_ and E_max_ values for 5-HT2A, 5-HT4, 5-HT5A, 5-HT6, and 5-HT7 receptors, and thus, these values were arbitrarily set to 5 and 0%, respectively. 5-MeO = 5-methoxy-*N,N*-dimethyltryptamine; AMPH = *S*-amphetamine.

Although **UCD0076** is a potent agonist of 5-HT2C receptors (Fig. 5, EC_50_ = 334 nM, E_max_ = 83%), it exhibits very low affinity for psychLight2 5-HT2A receptors (Fig. 2, K_i_ = 1.97 μM) indicating that the pyrrole-fused phenethylamine motif of LSD is sufficient to achieve high selectivity for 5-HT2C over 5-HT2A receptors. Given that 5-HT2C receptor agonism is known to produce antipsychotic effects (42) and **UCD0076** did not elicit a HTR (Fig. 7*B*), we reasoned that it might be useful as an antipsychotic-like agent. To confirm that its hallucinogenic potential is low, we first used psychLight—a biosensor capable of predicting the hallucinogenic potential of 5-HT2A ligands (26). As expected, **UCD0076** did not activate psychLight2 and served as a weak antagonist, consistent with its low affinity for psychLight2 5-HT2A receptors (Fig. 8*A*). Next, we pretreated animals with varying concentrations of **UCD0076** before administering 5-MeO-DMT (10 mg/kg), and we found that **UCD0076** suppressed psychedelic-induced HTR at the highest dose tested (Fig. 8*B*).

Agonists of 5-HT2C receptors are also known to indirectly modulate dopamine release in the striatum (43–45), so we assessed the ability of **UCD0076** to suppress amphetamine-induced hyperlocomotion (Fig. 8 *C*–*E*). After treatment with **UCD0076**, animals were allowed to freely explore an open field arena, and we observed a dose-dependent decrease in locomotion (Fig. 8*E*). Stimulation with amphetamine (3 mg/kg) resulted in the expected increase in locomotion, which was attenuated by **UCD0076** in a dose-dependent manner (Fig. 8*E*). Given that LSD exhibits high potency/efficacy for activating dopamine receptors (6), we wanted to determine if **UCD0076** also acted as a D2 ligand. Thus, we tested **UCD0076** and 4 additional ergologs in BRET-based assays of D2 receptor activation (*SI Appendix*, Fig. S15 and Table S4). We found that **UCD0076** is a weak partial agonist of D2 receptors (EC_50_ = 588 nM, E_max_ = 43%), which may contribute to its antipsychotic-like effects.

To better understand the polypharmacology of **UCD0076**, we determined its activity across 12 serotonin receptors (Fig. 8*F* and *SI Appendix*, Table S5). These studies demonstrated that **UCD0076** is also a high efficacy (E_max_ > 80%) agonist of all 5-HT1 receptors with much lower activity at other serotonin receptor subtypes. Though the potency of **UCD0076** for activating most 5-HT1 subtypes is modest (EC_50_ ~ 500 to 800 nM), its potency at 5-HT1D receptors (EC_50_ = 134 nM) is comparable to its 5-HT2C receptor potency. Thus, activity at 5-HT1 receptors could also be contributing to the antipsychotic-like properties of **UCD0076**.

## Discussion

The complex tetracyclic core of LSD is an ideal testbed for the principles of function-oriented synthesis. In fact, others have suggested studying simplified D-ring analogues of LSD as potential therapeutics (46). By systematically deconstructing LSD into smaller fragments, we found that tricyclic and bicyclic analogues retained interesting biological activity across the 5-HT2 family of receptors, while the monocyclic analogue **UCD0162** was completely inactive. Moreover, our studies enabled us to determine that an intact tetracyclic ergoline core is essential for maintaining high efficacy and potency for Gq activation following 5-HT2A receptor stimulation.

Several studies have suggested that 5-HT2A receptor agonists, like LSD, must achieve a threshold level of Gq activation to elicit hallucinogenic behavioral effects (35, 47, 48), though the exact threshold is not well defined and is likely tied to pharmacokinetic considerations related to maximal receptor occupancy (49). Signaling through Gq appears to be more relevant to the hallucinogenic effects of psychedelics as β-arrestin2 biased agonists with low Gq efficacy do not produce a HTR (35, 50). Our studies demonstrated that the A and D rings of LSD represent the minimal pharmacophore necessary to produce hallucinogenic effects. While these rings are necessary, they are not sufficient as **UCD0121** and **UCD0068** exhibited lower relative affinities and potencies at 5-HT2A receptors and did not induce a HTR.

It is important to note that the majority of the ergologs that we synthesized and tested were produced as racemic mixtures. The absolute stereochemistry at C5 is essential for LSD to bind to 5-HT2A receptors with high affinity (32), and we confirmed that this is also the case for the tricyclic ergolog **UCD0094**. Thus, racemic compounds containing a stereogenic center at C5 such as **UCD0121**, **UCD0068**, **UCD0830**, and **UCD0829** might exhibit slightly reduced affinity/potency compared to enantiopure versions matching the stereochemistry of LSD and C5. In contrast, the absolute stereochemistry at C8 does not seem to drastically impact the affinity/potency of LSD (32) but LSD analogues with the *S*-configuration at C8 (e.g., lisuride) tend to exhibit lower efficacy (35). Therefore, it is possible that the E_max_ values reported for **UCD0120** and **UCD0179** represent a combination from opposing epimers with comparable affinities. Future studies should investigate whether the hallucinogenic effects of **UCD0120** result from the actions of a single enantiomer.

The tricyclic ergologs **(±)-UCD0094** and **(+)-UCD0094** remained relatively potent activators of 5-HT2A receptors. However, the efficacy of **UCD0094** was drastically reduced compared to LSD (~ 60% vs. 96%, respectively). Recent evidence suggests that partial agonists of 5-HT2A receptors can promote cortical neuron growth and produce therapeutic effects with minimal to no hallucinogenic effects (5, 20, 25, 51). Moreover, elimination of the D-ring reduced the efficacy of **UCD0094** compared to LSD for activating 5-HT2B receptor antitargets (31% vs. 71%), making **UCD0094** an attractive lead structure for the development of nonhallucinogenic psychoplastogens (52). Given the importance of cortical structural plasticity in treating a variety of neuropsychiatric and neurodegenerative conditions (53), nonhallucinogenic psychoplastogens have broad potential in central nervous system drug discovery.

Converting LSD into a partial 5-HT2A receptor agonist is one strategy for maximizing therapeutic benefit while minimizing risk. Alternatively, ergologs exhibiting preference for 5-HT2C receptor activation over 5-HT2A and 5-HT2B receptors could also prove useful, as 5-HT2C agonists have shown potential for treating addiction, psychosis, and various seizure disorders (54). Both **UCD0094** and **UCD0076** exhibited reduced potency and efficacy at 5-HT2A and 5-HT2B receptors compared to LSD, but **UCD0076** demonstrated a strong preference for activating 5-HT2C over 5-HT2A and 5-HT2B receptors. Despite being a potent (EC_50_ = 334 nM) nearly full agonist (E_max_ = 83%) of 5-HT2C receptors, **UCD0076** exhibits extremely low affinity for psychLight2 5-HT2A receptors (~2 μM) where it acts as a weak antagonist. Given its low affinity for psychLight2 5-HT2A receptors coupled with its ability to activate 5-HT2C and 5-HT1 receptors and serve as a D2 partial agonist, we reasoned that **UCD0076** might modulate serotonergic (HTR) and dopaminergic (AMPH-induced hyperlocomotion) behaviors relevant to psychosis.

While 5-HT2C agonism is generally believed to result in hypolocomotion (55–57), conflicting reports have emerged about the involvement of 5-HT2C receptors in the mouse HTR. Some reports have demonstrated that 5-HT2C receptor agonists decrease psychedelic-induced HTR (58), while low doses of 5-HT2C receptor antagonists potentiate the response (59). However, high doses of 5-HT2C receptor antagonists suppress the HTR, calling into question the selectivity of these agents for 5-HT2C over 5-HT2A receptors at high doses (60, 61). In contrast, others contend that high doses of the 5-HT2C receptor antagonist SB206553 antagonize psychedelic-induced HTR while maintaining selectivity for 5-HT2C over 5-HT2A receptors (62). Moreover, 5-HT2C receptor knockout mice have a blunted psychedelic-induced HTR (62)

We found that **UCD0076** antagonized both psychedelic-induced HTR and psychostimulant-induced hyperlocomotion; however, its effects in the latter assay were more potent than the former. Even the lowest dose of **UCD0076** tested (0.6 mg/kg) attenuated amphetamine-induced hyperlocomotion despite exerting negligible effects on locomotion in the absence of a psychostimulant, suggesting that 5-HT2C agonism and/or D2 partial agonism can potently modulate response to dopamine releasing agents. In stark contrast, **UCD0076** only inhibited psychedelic-induced HTR at the highest dose tested (31 mg/kg). As 5-HT1A agonism has been shown to inhibit psychedelic-induced HTR (63), we therefore conclude that the ability of **UCD0076** to suppress a psychedelic-induced HTR is likely due to its low affinity antagonism of 5-HT2A receptors or 5-HT1A agonism rather than 5-HT2C agonism, though additional off-target screening to confirm this hypothesis is warranted. Regardless, the preference of **UCD0076** for activating 5-HT2C receptors over other 5-HT2 subtypes make it an interesting lead scaffold for the development of antipsychotic, antiaddictive, and antiseizure agents.

While our studies primarily focused on the 5-HT2 family of receptors, it is important to note that LSD exhibits complex polypharmacology and activates a wide variety of serotonin, dopamine, and adrenergic receptors (6). It will be important for future studies to determine how ergologs interact with these targets. For example, we found that **UCD0094** is a relatively potent D2 agonist (EC_50_ = 29 nM, E_max_ = 78%) while **UCD0121** is not active.

Here, we found that the A and D rings of the ergoline core are essential for LSD to maximally activate 5-HT2A receptors and produce hallucinogenic behavioral effects. By removing the D ring, we created several LSD analogues with reduced hallucinogenic potential that might serve as antidepressants, antipsychotics, or antiseizure agents. Taken together, our results demonstrate the power of function-oriented synthesis and that systematic deconstruction of natural product scaffolds can reveal strategies for modulating the polypharmacological profile of structurally complex compounds like LSD.

## Methods

### Chemistry (General).

All reagents were obtained from commercial sources and were used without further purification unless noted otherwise. Reactions were performed using oven-dried glassware (120 °C) under an argon atmosphere unless noted otherwise. Air- and moisture-sensitive liquids and solutions were transferred via syringe. Organic solutions were concentrated under reduced pressure (∼5 Torr) by rotary evaporation. Chromatography was performed using Fisher Chemical™ Silica Gel Sorbent (230 to 400 Mesh, Grade 60). Compounds purified by chromatography were typically dissolved in a minimal amount of chloroform for loading. Thin layer chromatography (TLC) was performed on Merck silica gel 60 F_254_ plates. Visualization of the developed chromatogram was accomplished by fluorescence quenching or by staining with aqueous potassium permanganate, ceric ammonium molybdate (CAM), or Ehrlich’s reagent.

#### NMR.

NMR spectra were acquired on a Bruker 300 operating at 300 and 75 MHz, a Bruker 400 operating at 400 and 100 MHz, or a Varian 600 operating at 600 and 150 MHz for ^1^H and ^13^C, respectively, and are referenced internally according to residual solvent signals (CDCl_3_, CD_3_OD, acetic acid-*d*_4_, DMSO-*d*_6_). Data for ^1^H NMR are recorded as follows: chemical shift (δ, ppm), multiplicity (s, singlet; d, doublet; t, triplet; q, quartet; quint, quintet; m, multiplet; br, broad), coupling constant (Hz), and integration. Data for ^13^C NMR are reported in terms of chemical shift (δ, ppm).

#### LC–MS.

Liquid chromatography-mass spectrometry (LC–MS) was performed using two instruments: 1) a Waters Alliance 2695 HPLC with a Waters Micromass ZQ Detector; 2) a Waters UHPLC−MS with an ACQUITY Arc QDa detector.

#### HRMS.

High-resolution mass spectra (HRMS) were obtained using a Thermo Fisher Scientific Q-Exactive HF Orbitrap.

#### FT–IR.

Infrared spectra were recorded using a Thermo Nicolet iS10 Fourier transform infrared (FT–IR) spectrometer with a Smart iTX Accessory [diamond attenuated total reflection (ATR)] and are reported in the frequency of absorption (ν, cm^−1^).

#### Purity.

All compounds tested in biological assays were confirmed to be of >95% purity based on UHPLC analysis (Waters ACQUITY Arc, 2489 UV/Vis Detector) measuring absorbance at 210, 225, 254, or 280 nm. Mobile phase A consisted of 0.1% formic acid in water, and mobile phase B consisted of 0.1% formic acid in acetonitrile. All samples were injected at a volume of 1 μL (Waters ACQUITY Arc, Sample Manager FTN-R), and the column was maintained at ambient temperature. A Waters Cortecs™ C18 column (2.7 µm, 4.6 × 50 mm) was used with a flow rate of 0.6 mL/min and a gradient from 20 to 95% mobile phase B over 3 min, which was maintained for an additional 3 min. All compounds reported in this study were isolated as the fumarate, hemifumarate, tosylate, or hydrochloride salts. Peaks in ultra-high pressure liquid chromatography (UHPLC) traces corresponding to the acid were not included in calculations of purity.

#### Ratio Determination.

The amine to acid ratio was determined by ^1^H NMR utilizing either CD_3_OD or DMSO-*d*_6_ as the deuterated solvent. In the case of the hydrochloride salts, the integrated ^1^H NMR signal of the protonated amine was compared to that of a C–H signal utilizing anhydrous DMSO-*d*_6_ as the deuterated solvent. Addition of D_2_O suppressed the ^1^H NMR signals of the exchangeable acidic protons. Detailed synthetic methods and characterization data are provided in *SI Appendix*.

### Pharmacology (General).

Salt forms of all compounds were used for biological testing (see supporting information for details). For in vitro pharmacological assays, all compounds were dissolved in DMSO and stored as 10 mM stock solutions in the dark at −20 °C. For in vivo studies, solutions of the compounds in 0.9% saline were prepared fresh immediately prior to performing the experiment. The 5-MeO used in the HTR experiments was prepared as described previously, (64) while *S*-amphetamine hemisulfate (Sigma-Aldrich 1180004) and 5-HT·HCl (Millipore Sigma, cat. # H9523) were purchased. Treatments were randomized, and in vivo data were analyzed by experimenters blinded to treatment conditions. Statistical analyses were performed using GraphPad Prism (version 10.5.0). All comparisons were planned prior to performing each experiment. Data are represented as mean ± SEM, unless noted otherwise, with asterisks indicating *P < 0.05, **P < 0.01, ***P < 0.001, and ****P < 0.0001. Detailed materials and methods for each pharmacology experiment are provided in *SI Appendix*.

## Disclosure

D.E.O. is a cofounder of Delix Therapeutics, Inc., serves as the Chief Innovation Officer and Head of the Scientific Advisory Board, and has sponsored research agreements with Delix Therapeutics. Delix Therapeutics has licensed LSD-related technology from the University of California, Davis. The sponsors of this research were not involved in the conceptualization, design, decision to publish, or preparation of the manuscript.

## Supplementary Material

Appendix 01 (PDF)

## Data Availability

Custom scripts for psychLight analysis are available at https://github.com/moagonzalez/OlsonLab/blob/main/PsychLightV3_Clean.txt (65). Data are available at the following link https://figshare.com/s/5011fc52b804a5e08c39 (66). All X-ray were deposited in the Cambridge Crystallographic Data Centre under deposition numbers 2322609 and 2322610.
