# Supplementary material for: Deconstruction of lysergic acid diethylamide
Source: Proc Natl Acad Sci U S A. 2026 Sep 8;123(38):e2603412123. doi: 10.1073/pnas.2603412123 (PMC13598156; doi:10.1073/pnas.2603412123)
Supplement: Supplementary file 1 [file pnas.2603412123.sapp.pdf]

Supporting Information for
Deconstruction of Lysergic Acid Diethylamide

Andrian G. Basargin,† Andras Domokos,† Joseph J. Hennessey, Isak K. Aarrestad, Rohini Sambyal, Yara A. Khatib, Johanna Krüger, Lee E. Dunlap, Samuel J. Carter, Isabella A. Rebek, John L. McKee, Serena S. Schalk, Min Liu, James C. Fettinger, Monica A., Gonzalez, Abhay Potluri, Dean J. Tantillo, Oliver Fiehn, John D. McCorvy, David E. Olson*

David E. Olson
Email: deolson@ucdavis.edu

This PDF file includes:

Supporting text
Figures S1 to S15
Tables S1 to S5
SI References

Table of Contents

1. *Supplemental Figures and Tables (Page S2 – S22)*
2. *Materials and Methods (Page S23 – S27)*
3. *General Synthetic Procedures (Page S28)*
4. *Detailed Synthetic Procedures and Experimental Data for all Compounds (Page S28 – S59)*
5. *Chiral LC-MS Traces (Page S59 – S62)*
6. *UHPLC-MS Purity Traces (Page S63 – S71)*
7. *Additional characterization of **UCD0068** (Page S72 – S76)*
8. *^{1}H and ^{13}C NMR Spectra (Page S77 – S121)*
9. *X-Ray Crystallography (Pages S122 – S139)*
10. *Pharmacokinetic studies of **UCD0120** and **UCD0179** (Page S140 – S141)*
11. *Supplemental References (Page S142 – S143)*

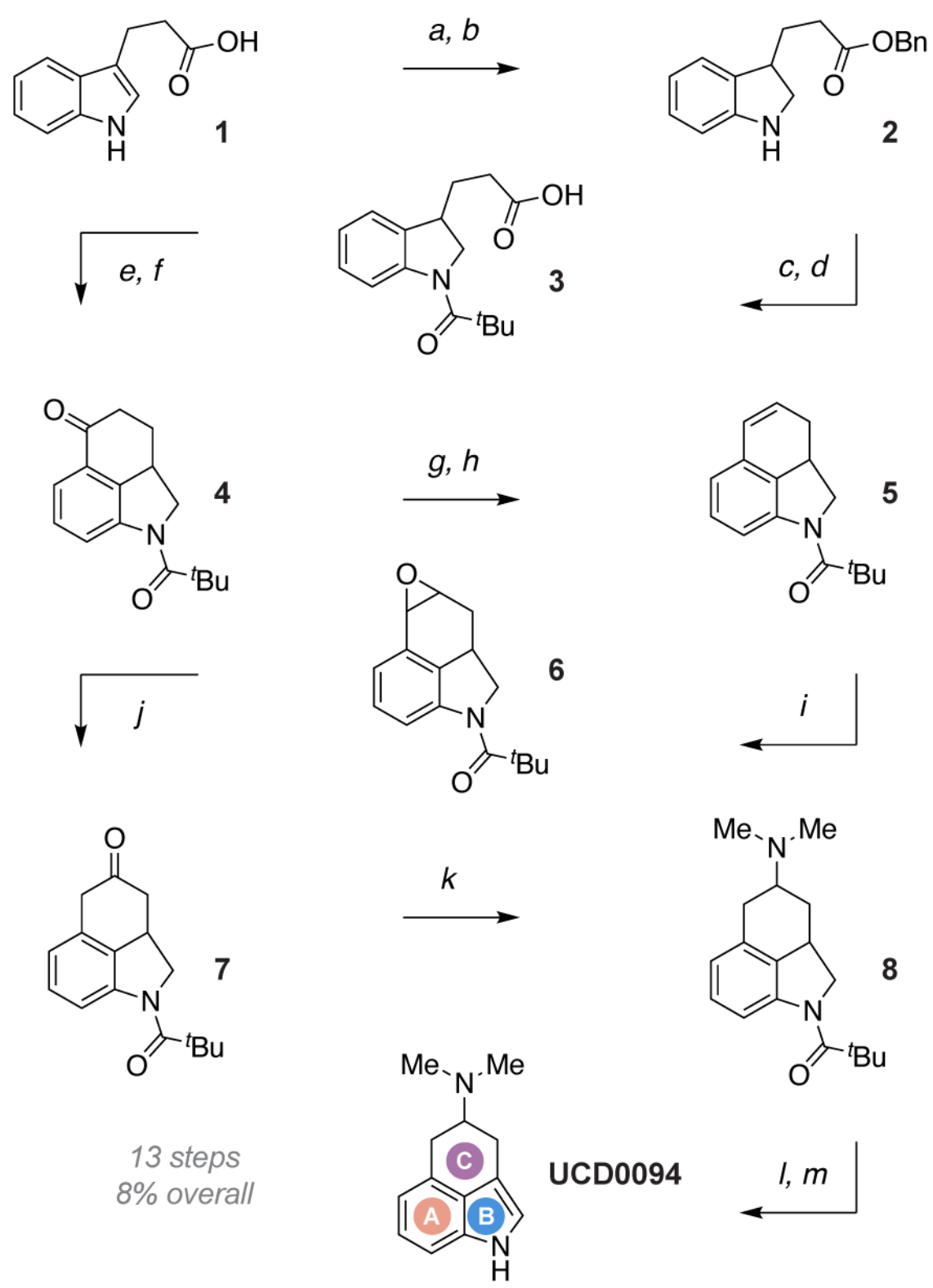

Figure S1. Synthesis of UCD0094. (a) DCC, DMAP, BnOH, DCM (b) $NaCNBH_3$, AcOH, 59% (2 steps) (c) C_6H_5N, PivCl, $CHCl_3$ (d) H_2, Pd/C, EtOH, 84% (2 steps) (e) $SOCl_2$, DCM (f) $AlCl_3$, DCM, 66% (2 steps) (g) $NaBH_4$, EtOH (h) amberlyst, DCE, 93% (2 steps) (i) mCPBA, $CHCl_3$, 88% (j) ZnI_2, C_6H_6, 95% (k) Me_2NH, $NaCNBH_3$, MeOH, AcOH, 53% (l) ^{n}BuLi, THF (m) DDQ, THF, 61% (2 steps).

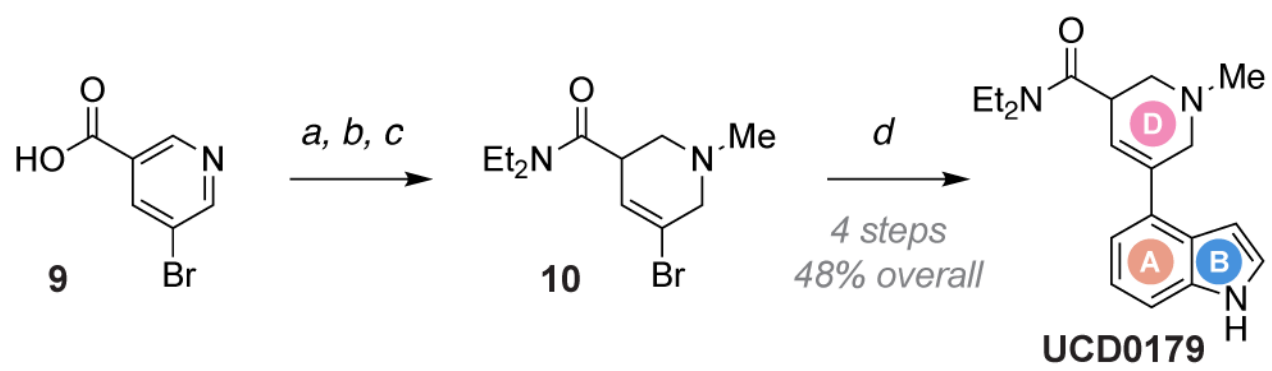

Figure S2. Synthesis of UCD0179. (a) $(COCl)_2$, DMF, DCM then Et_2NH, DCM (b) MeI, EtOAc, 70ºC, 94% (2 steps) (c) $NaCNBH_3$, AcOH, MeOH, 94% (d) indole-4-boronic acid pinacol ester, $Pd(PPh_3)_4$, K_2CO_3, H_2O, PhMe, 80ºC, 54%.

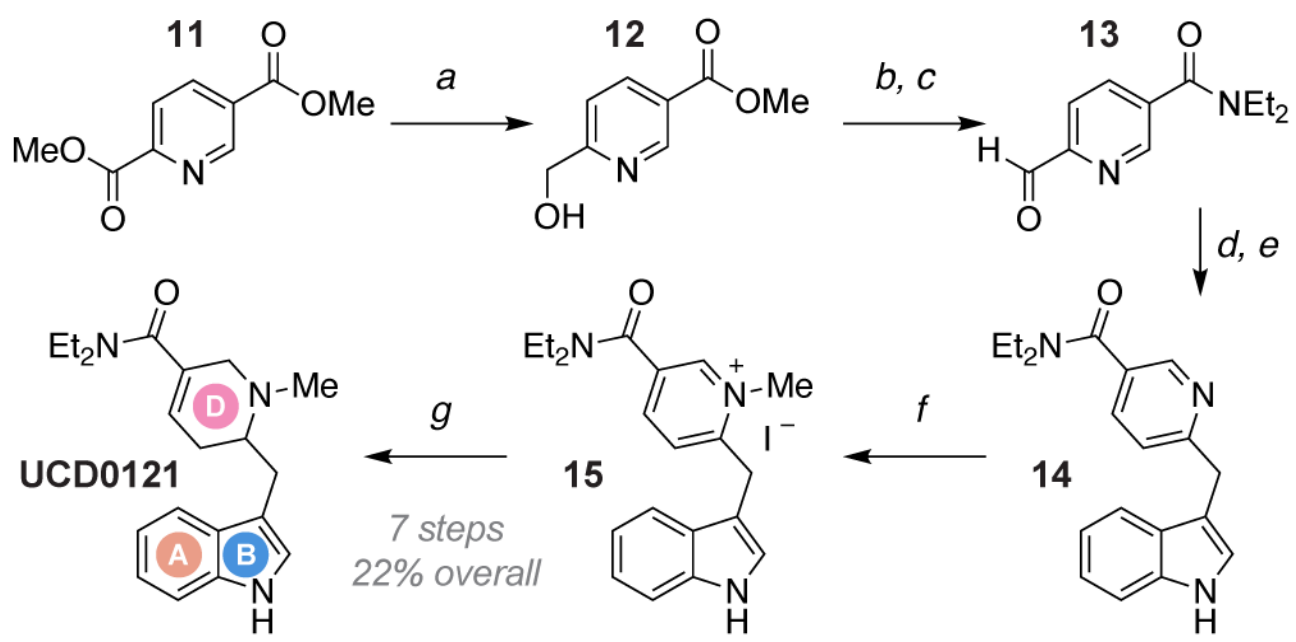

Figure S3. Synthesis of UCD0121. (a) $NaBH_4$, $CaCl_2$, THF, MeOH, 0ºC, 71% (b) ^{n}BuLi, Et_2NH, THF, -78ºC, 79% (c) $(COCl)_2$, DMSO, Et_3N, DCM, -78ºC, 71% (d) indole, NaOH, MeOH, 0ºC, 77% (e) Hantzsch ester, TsOH, $CHCl_3$, 95% (f) MeI, CH_3CN, 70ºC (g) $NaBH_4$, MeOH, 74% (2 steps).

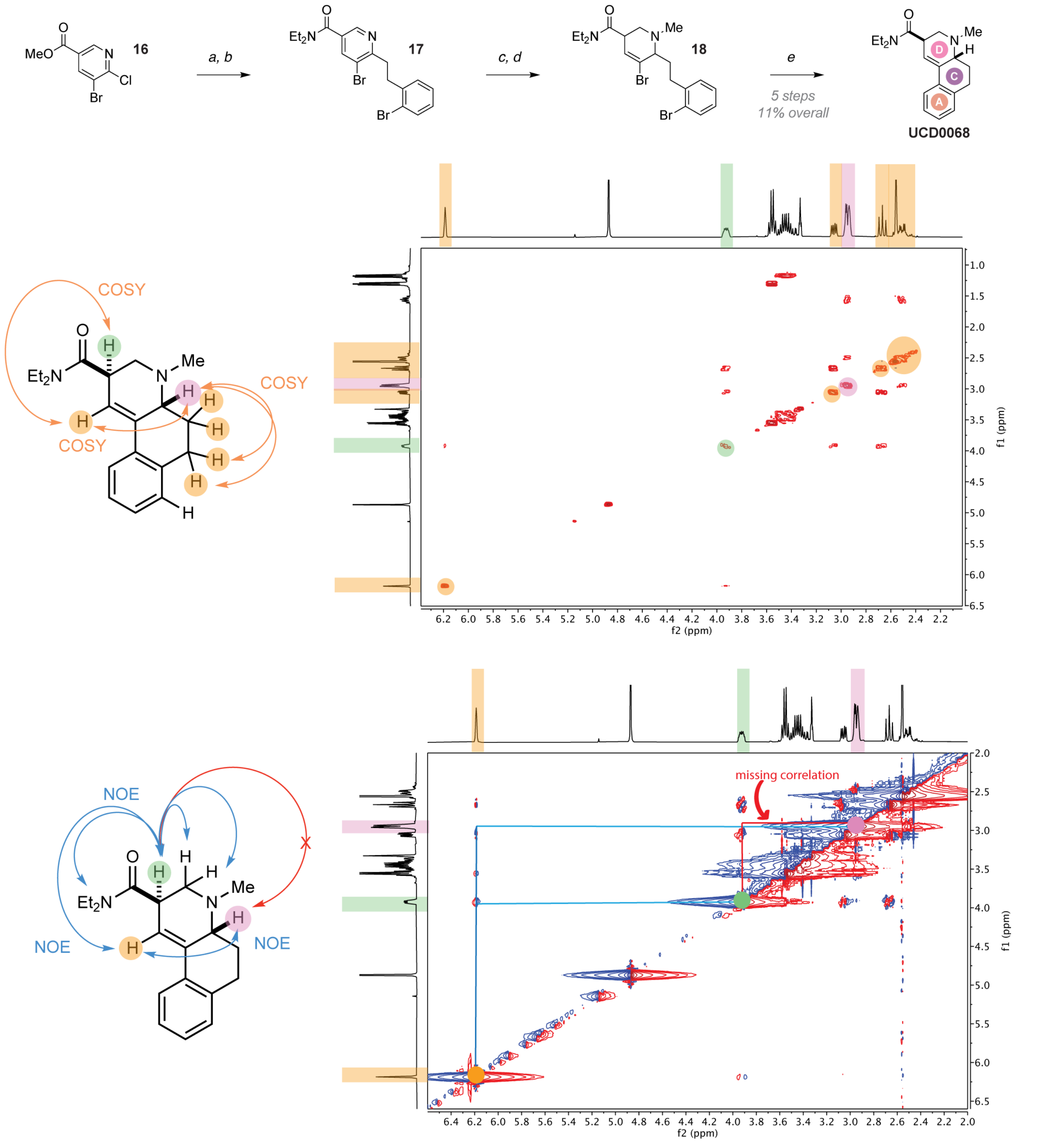

Figure S4. Synthesis of UCD00068. (a) $CH_2(CO_2Me)_2$, K_2CO_3, DMF, then 2-bromobenzyl bromide, then NaOH, 80ºC, then H_2SO_4, 80ºC, 58% (b) $(COCl)_2$, DMF, DCM, then Et_2NH, 83% (c) MeI, CH_3CN, 70ºC, 97% (d) $NaBH_4$ then $NaCNBH_3$, MeOH, AcOH, 65% (e) Pd_2dba_3, Xphos, K_3PO_4, B_2Pin_2, 1,4-dioxane, 100ºC, 37%. COSY data indicate which ^1H atoms are coupled. NOESY data indicate a trans relationship between the substituents on the 8- and 5-positions (ergoline numbering). See supporting information for additional information.

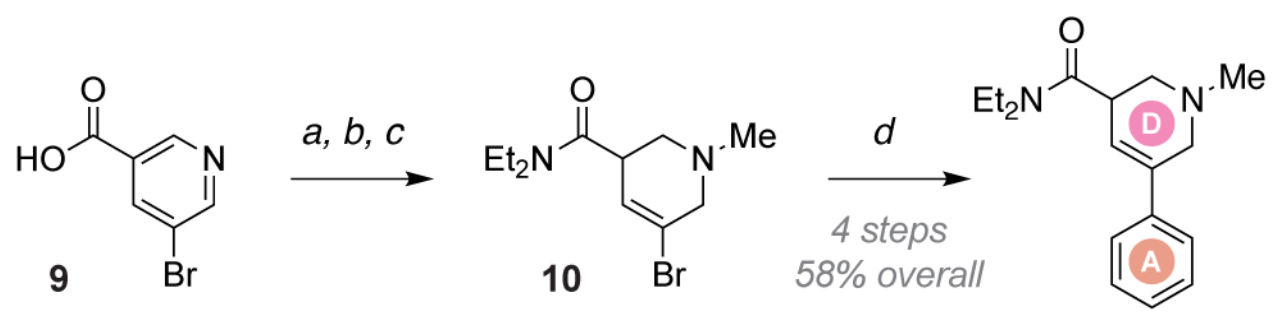

Figure S5. Synthesis of UCD0120. (a) $(COCl)_2$, DMF, DCM then Et_2NH, DCM (b) MeI, EtOAc, 70ºC 94% (2 steps) (c) $NaCNBH_3$, AcOH, MeOH, 94% (d) phenylboronic acid, $Pd(PPh_3)_4$, K_2CO_3, H_2O, PhMe, 80ºC, 66%.

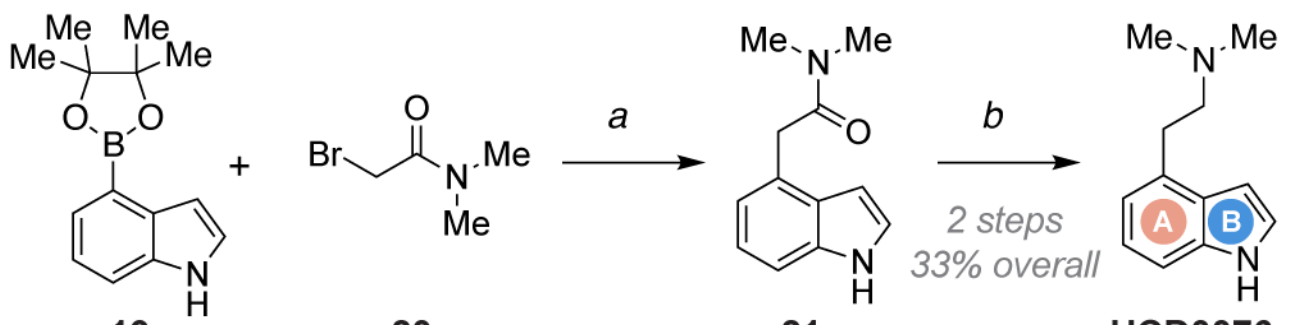

Figure S6. Synthesis of UCD0076. (a) $Pd(PPh_3)_4$, K_2CO_3, H_2O, MeCN, 80ºC (b) $LiAlH_4$, THF, 0ºC, 33% (2 steps).

22

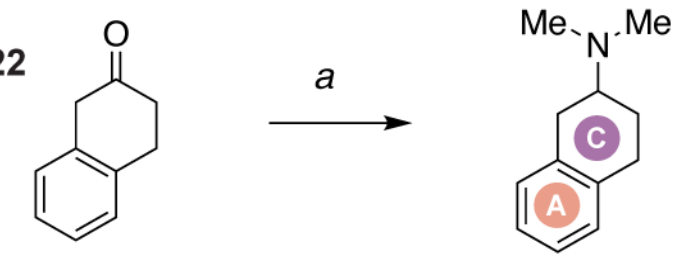

UCD0830

1 step
38% overall

Figure S7. Synthesis of UCD0830. (a) Me_2NH, $NaCNBH_3$, AcOH, MeOH, 38%.

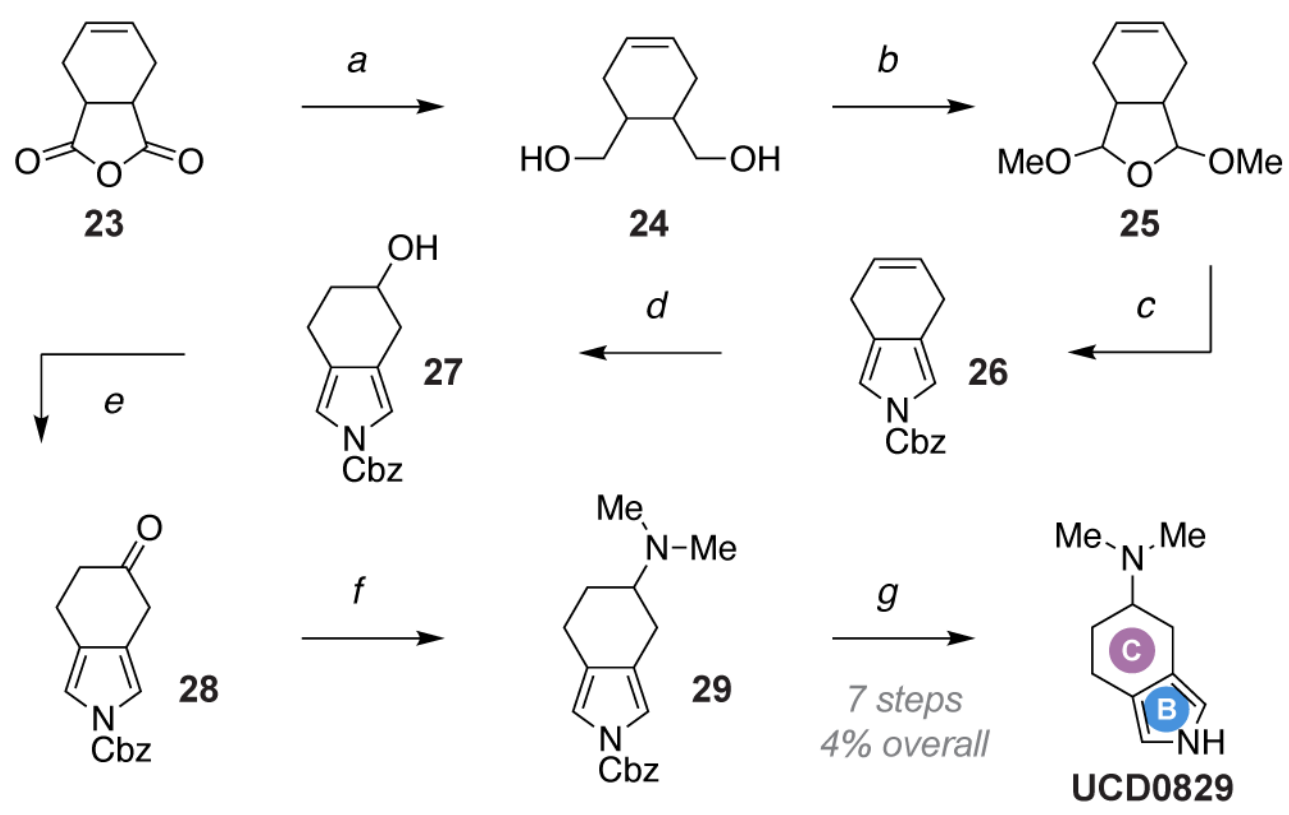

Figure S8. Synthesis of UCD0829. (a) $LiAlH_4$, THF, 0ºC, 99% (b) $(COCl)_2$, DMSO, Et_3N, DCM, -78ºC then TsOH, MeOH, 67% (c) $CBzNH_2$, P_2O_5, PhMe, 100ºC, 51% (d) BH_3, THF then NaOH, H_2O_2, 86% (e) $(COCl)_2$, DMSO, Et_3N, DCM, -78ºC, 52% (f) Me_2NH, $NaCNBH_3$, AcOH, MeOH, 52% (g) H_2, Pd/C, MeOH, 53%.

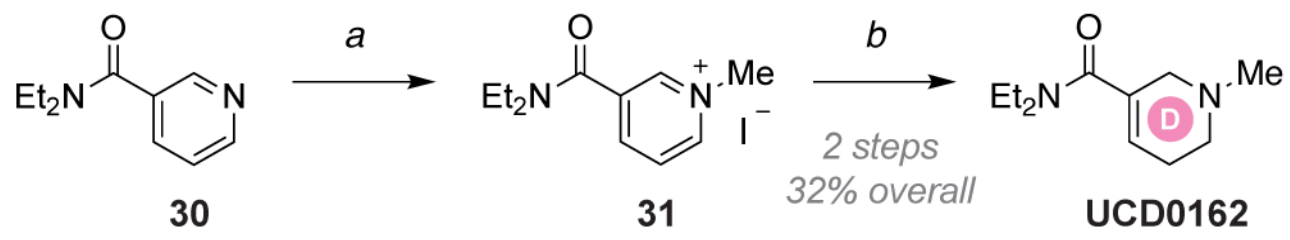

Figure S9. Synthesis of UCD0162. (a) MeI, MeCN, 70ºC, 88% (b) $NaCNBH_3$, AcOH, MeOH, 36%.

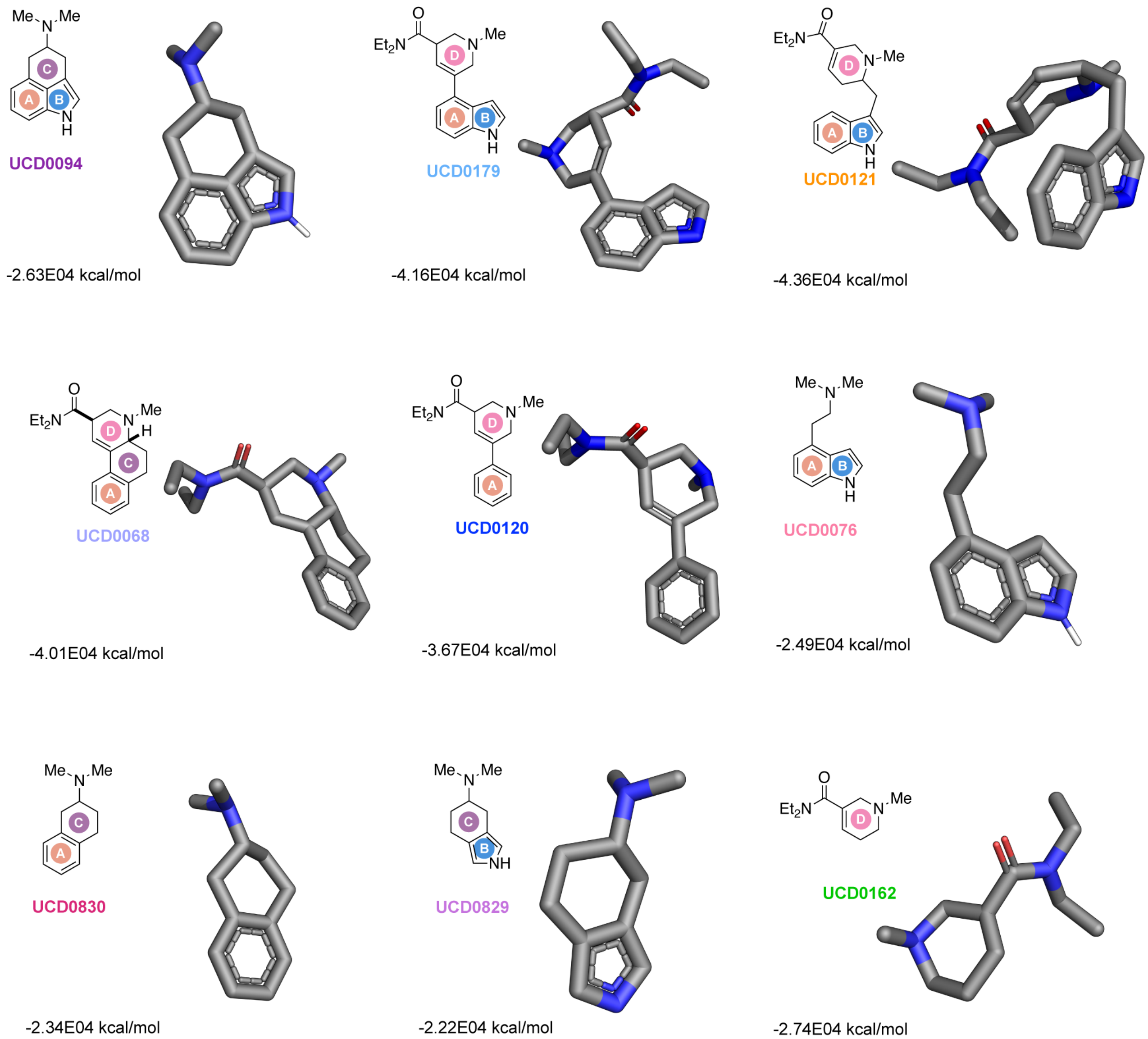

Figure S10. Minimum energy conformations of ergologs. Optimizations of ergolog structures in water were performed using eXtended Tight-Binding (xTB) without considering absolute stereochemistry and conformer sampling was conducted with Conformer-Rotamer Ensemble Sampling Tool (CREST) with GNF2-xTB calculations. Anywhere between 4 and 292 conformers were generated for each of the 9 compounds. The lowest energy conformers were selected and shown here, along with their respective energy in kcal/mol.

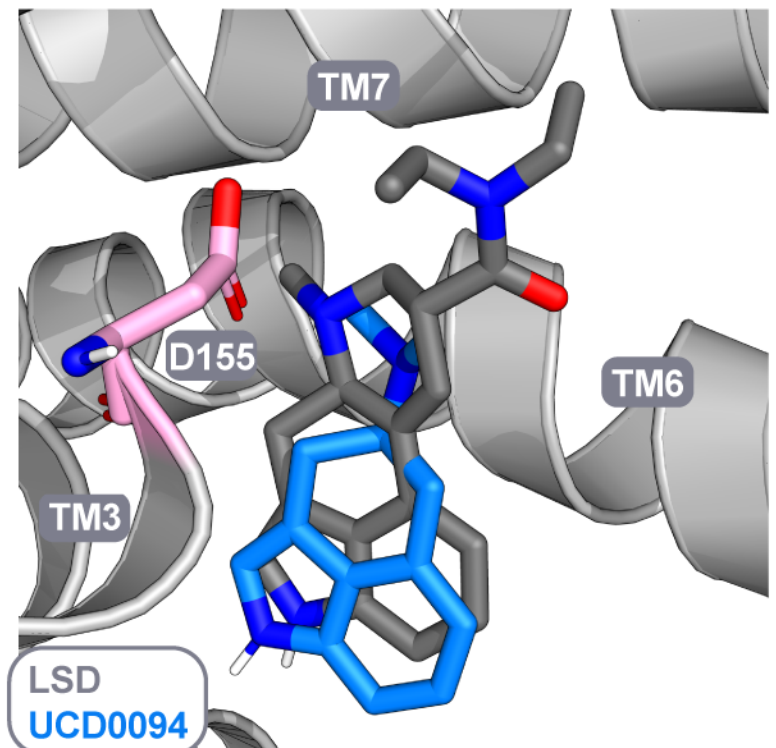

Conformer/ Pose	Affinity (kcal/mol)	Distance from N to D155 (Å)	RMSD (Å)
**1/1**	**-7.1**	**3.6**	**1.51**
2/1	-6.9	3.4	4.58
3/1	-7.1	3.7	1.36
4/2	-6.2	3.6	4.42

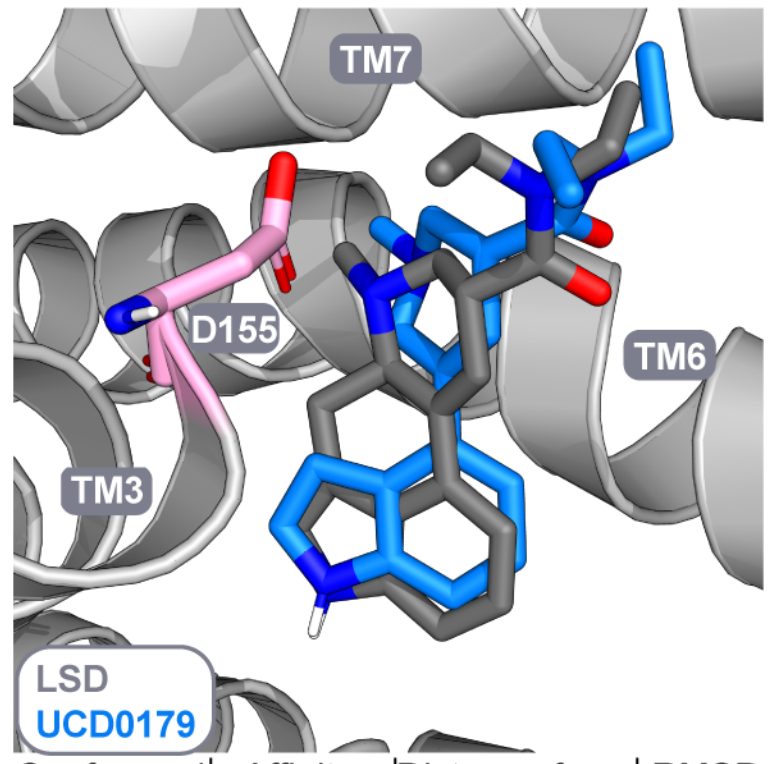

Conformer/ Pose	Affinity (kcal/mol)	Distance from N to D155 (Å)	RMSD (Å)
1/4	-7.4	3.5	1.73
28/4	-7.5	3.6	1.94
**65/6**	**-8.1**	**3.7**	**1.09**
68/5	-8.1	3.7	1.11

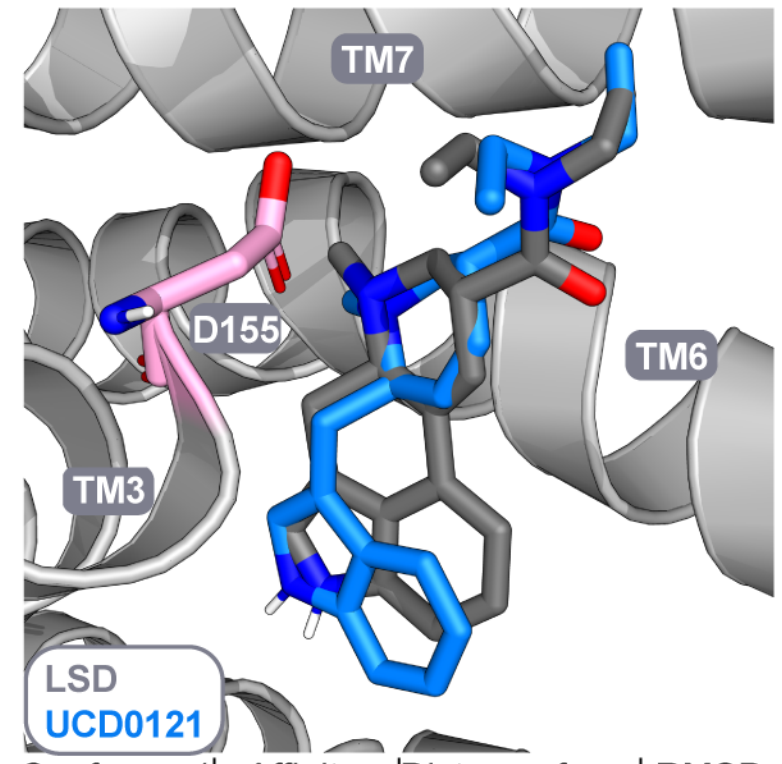

Conformer/ Pose	Affinity (kcal/mol)	Distance from N to D155 (Å)	RMSD (Å)
1/1	-8.6	2.9	1.07
146/8	-7.3	3.9	2.78
**221/1**	**-8.4**	**2.9**	**1.05**
292/4	-8.0	2.8	1.18

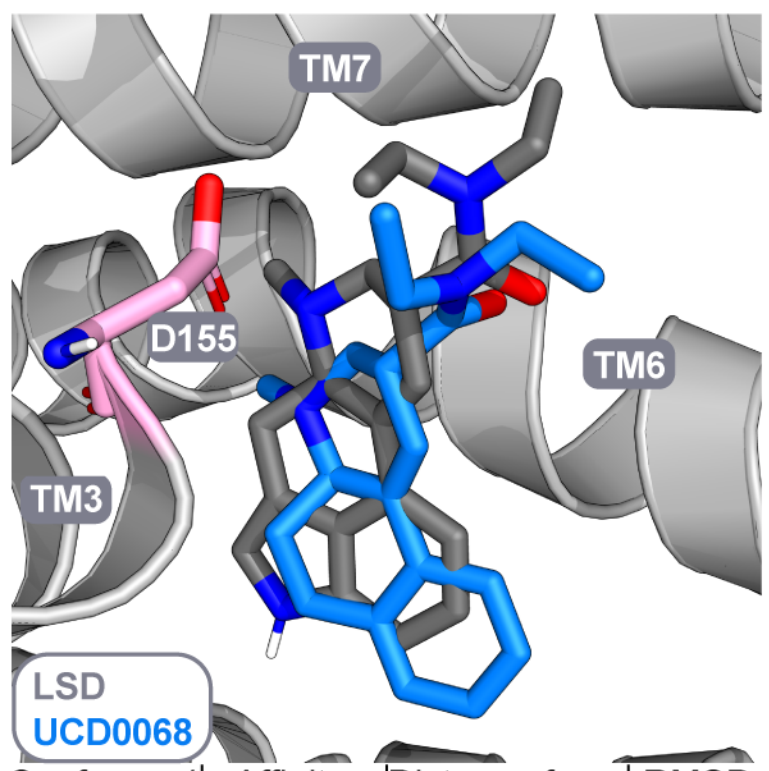

Conformer/ Pose	Affinity (kcal/mol)	Distance from N to D155 (Å)	RMSD (Å)
**1/1**	**-8.8**	**3.3**	**1.85**
34/1	-8.3	3.5	2.07
37/1	-8.7	3.4	2.11
48/3	-7.6	3.4	2.93

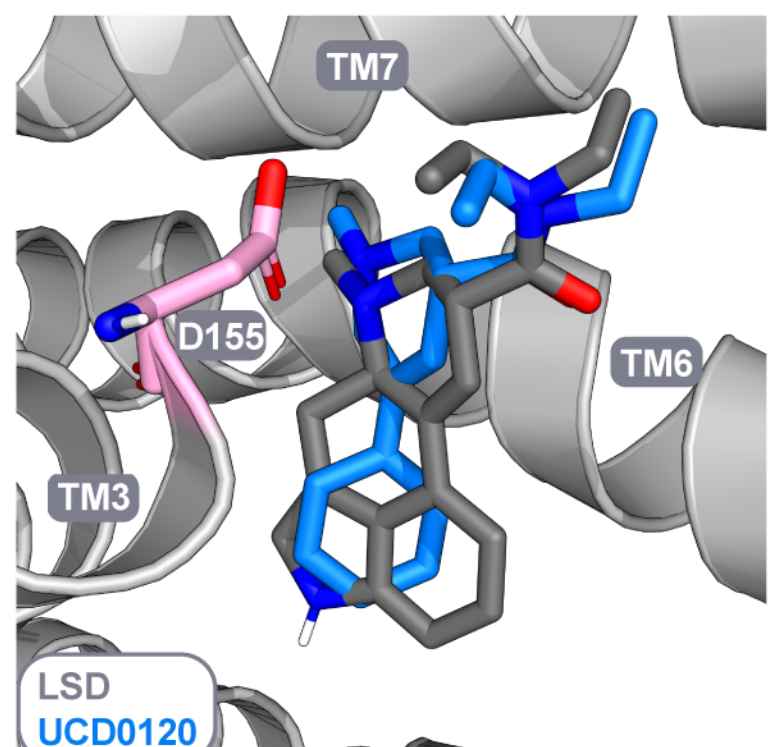

Conformer/ Pose	Affinity (kcal/mol)	Distance from N to D155 (Å)	RMSD (Å)
1/2	-7.3	3.8	2.08
33/8	-6.5	3.3	2.71
**54/2**	**-7.5**	**3.1**	1.24
123/3	-7.3	3.6	2.53

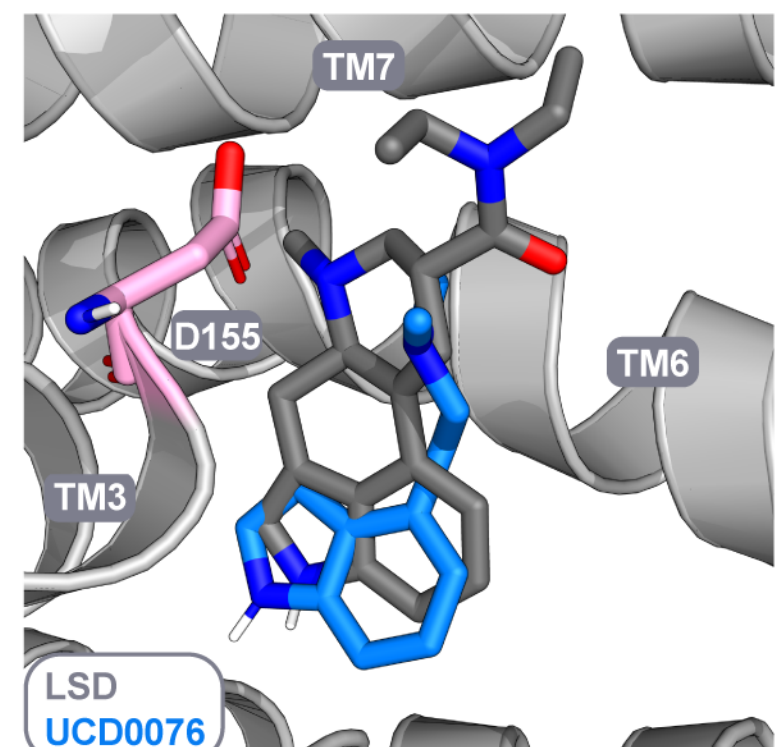

Conformer/ Pose	Affinity (kcal/mol)	Distance from N to D155 (Å)	RMSD (Å)
1/1	-6.3	3.4	1.88
**3/1**	**-6.4**	**3.4**	**1.90**
9/1	-6.4	3.5	2.03
10/1	-5.8	3.4	2.55

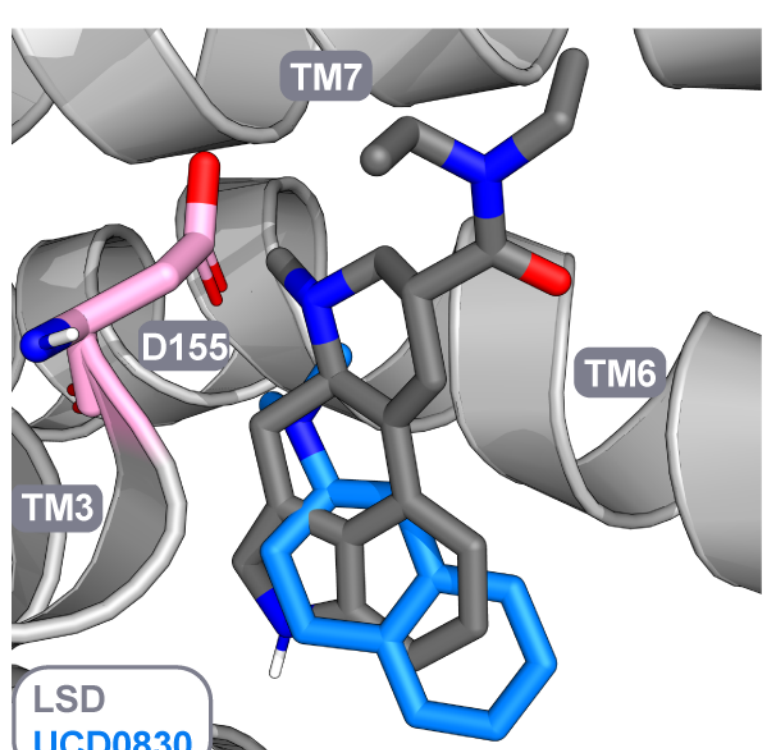

Conformer/ Pose	Affinity (kcal/mol)	Distance from N to D155 (Å)	RMSD (Å)
**1/6**	**-6.4**	**3.8**	**2.01**
2/3	-6.1	3.7	4.52
5/1	-6.2	3.8	5.20
6/4	-6.2	3.6	2.32

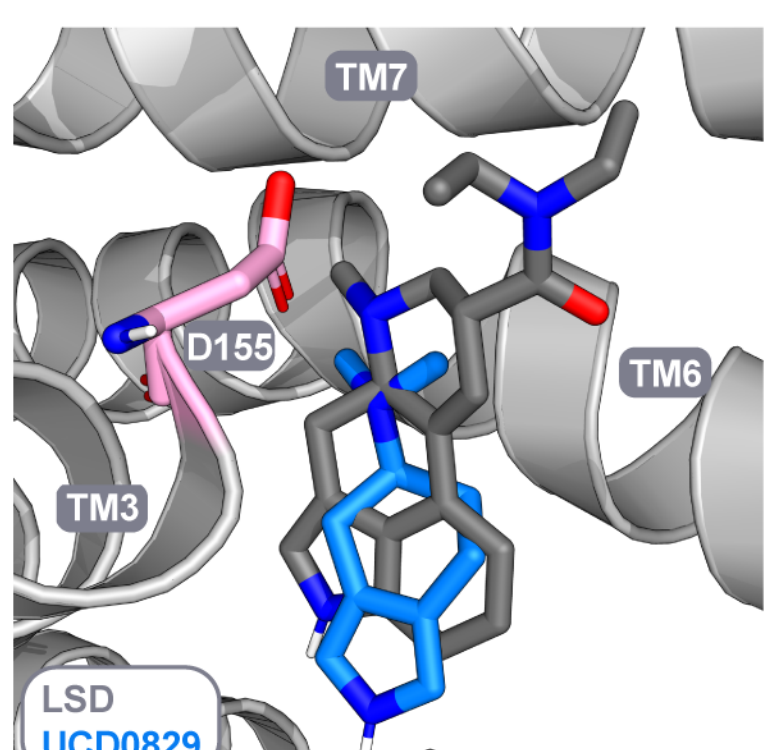

Conformer/ Pose	Affinity (kcal/mol)	Distance from N to D155 (Å)	RMSD (Å)
1/4	-5.6	3.1	2.85
6/6	-5.5	3.7	2.74
9/3	-5.5	3.8	2.36
**13/2**	**-5.7**	**3.4**	**2.11**

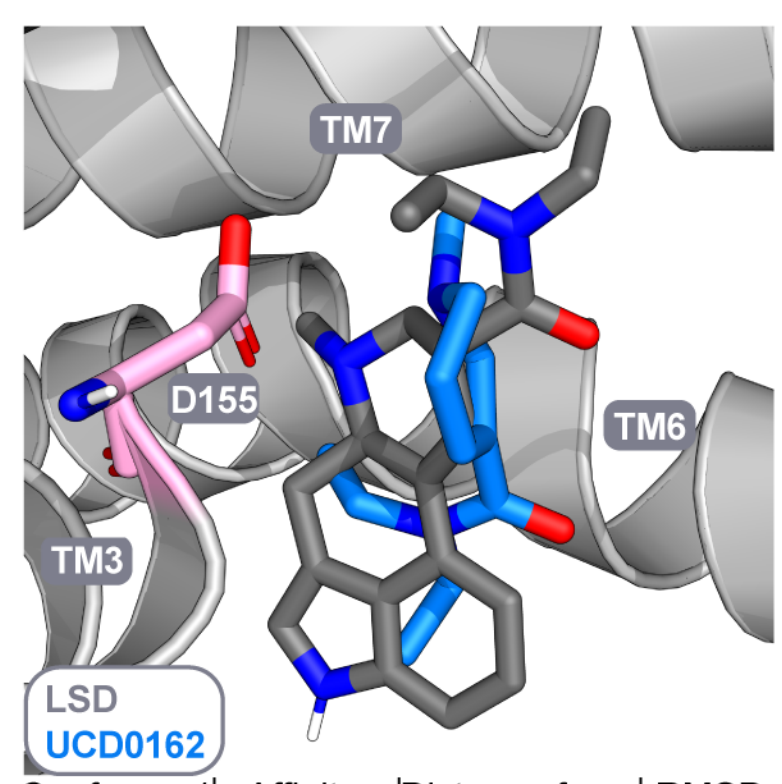

Conformer/ Pose	Affinity (kcal/mol)	Distance from N to D155 (Å)	RMSD (Å)
1/5	-5.3	3.5	6.50
4/8	-5.2	3.5	6.58
**7/1**	**-5.7**	**3.5**	**5.22**
8/1	-5.7	3.5	5.24

Figure S11. Docking of ergologs to the 5-HT2A receptor. From the library of conformers generated through CREST/xTB, the minimum energy conformation and three randomly selected conformers (n = 4) were chosen for docking using a crystal structure of LSD bound to an active-state 5-HT2A receptor in complex with mini-Gq/scFv16 (PDB: 9AS4). The conformers were prepared for docking in AutoDock Tools and docked to the crystal structure with the ligand removed using AutoDock Vina. Only the poses that had the basic nitrogen within 4 Å of the D155 residue were considered. The highest affinity pose for each conformer is shown along with its root mean squared

deviations (RMSDs) relative to LSD. The best pose based on highest calculated affinity (bolded) was visualized in PyMol and depicted in the figure (ergolog in light blue, and LSD in gray). Portions of TM3 were hidden for clarity.

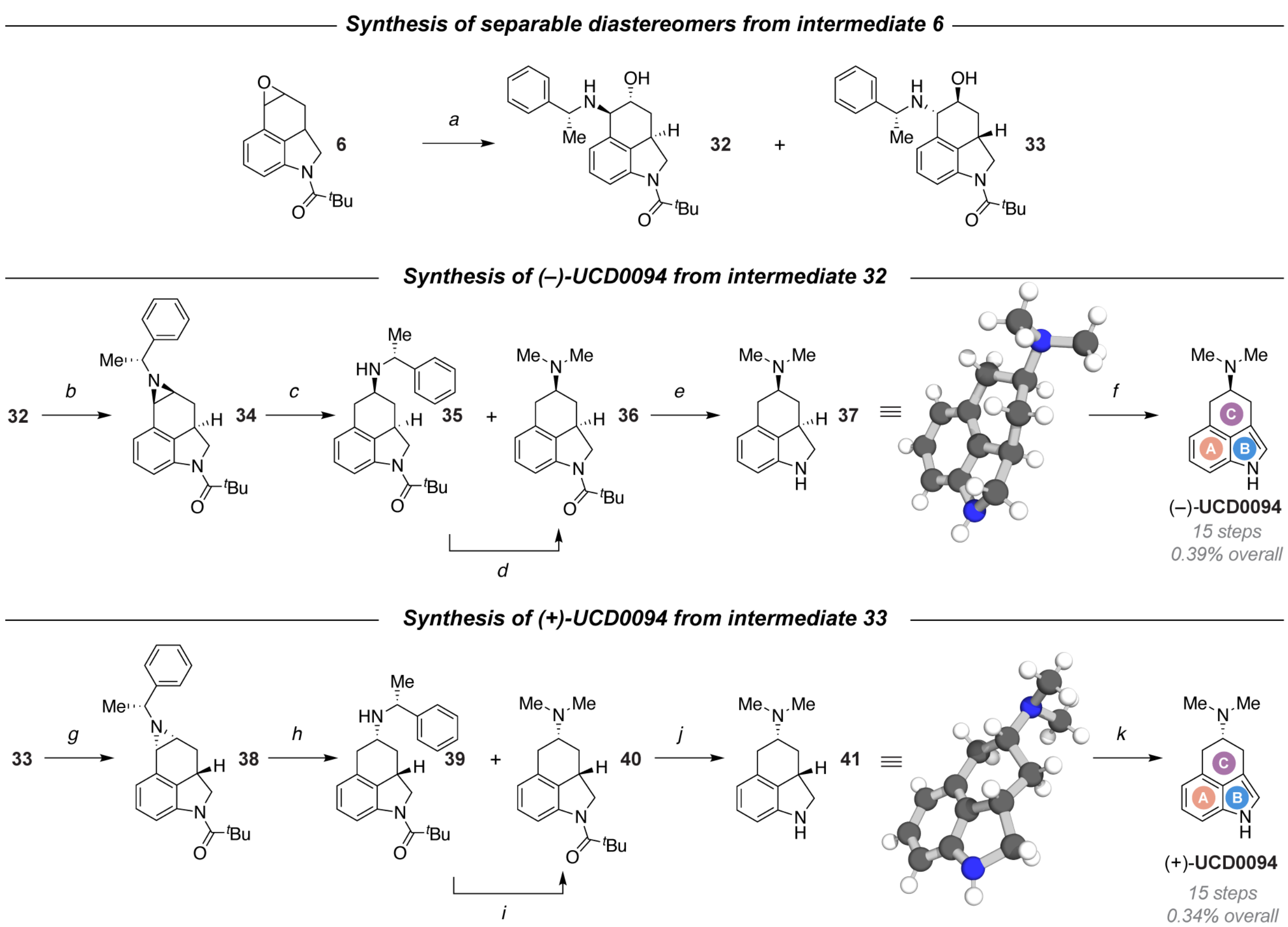

Figure S12. Asymmetric syntheses of (–)-UCD0094 and (+)-UCD0094. (a) (1*R*)-(+)-phenylethylamine, BuOH, 90ºC, 27% (**32**) and 34% (**33**) (b) MsCl, NEt_3, DCM, 89% (c) Pd/C, H_2, H_2SO_4/THF *then* ammonium formate, *then* CHO, AcOH, 35% (**35**) and 30% (**36**) (d) ammonium formate *then* CHO, AcOH, 29% (e) *n*-BuLi, THF, 93%, XRD crystal structure obtained to confirm stereochemistry of **37.** (f) DDQ, THF (75%) (g) MsCl, NEt_3, DCM, 93% (h) Pd/C, H_2, H_2SO_4/THF *then* ammonium formate *then* CHO, AcOH, 35% (**39**) and 32% (**40**) (i) ammonium formate *then* CHO, AcOH, 27% (j) *n*-BuLi, THF, 77%, XRD crystal structure obtained to confirm stereochemistry of **41**. (k) DDQ, THF, 60%.

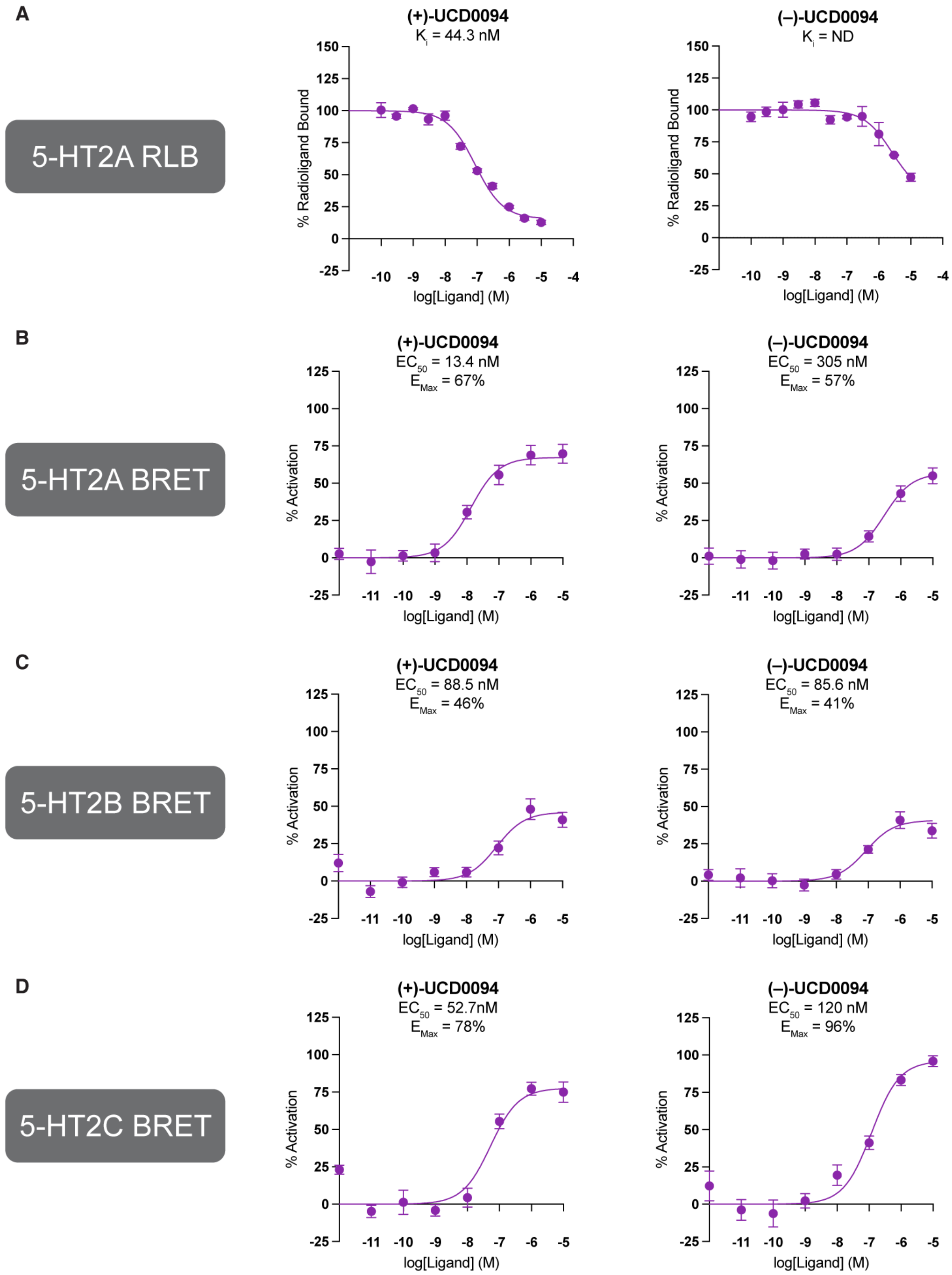

Figure S13. Biological data for (–)-UCD0094 and (+)-UCD0094. (A) Radioligand competition binding assays using membrane preparations from PSYLI2 cells (i.e., HEK293T cells stably expressing the 5-HT2A-based biosensor psychLight2) were performed to determine K_i values. Data represent the mean of well averages from 2–3 experimental replicates (i.e., independent batches of membrane preparations) ± SEM; N = 3 for experimental compounds. K_i values were not determined (ND) for compounds that did not produce at least 75% inhibition at 10 μM. (B–D) Potency (EC_{50}) and efficacy (E_{max}) of Gq activation was assessed using BRET-based methods in HEK293T cells transiently expressing 5-HT2A (B), 5-HT2B (C), or 5-HT2C (D) receptors. Data represent 2 wells from 3–6 experimental replicates (i.e., independent cultures) and are normalized to the serotonin response with the bottom of the curves constrained to 0. N = 7 for all compounds.

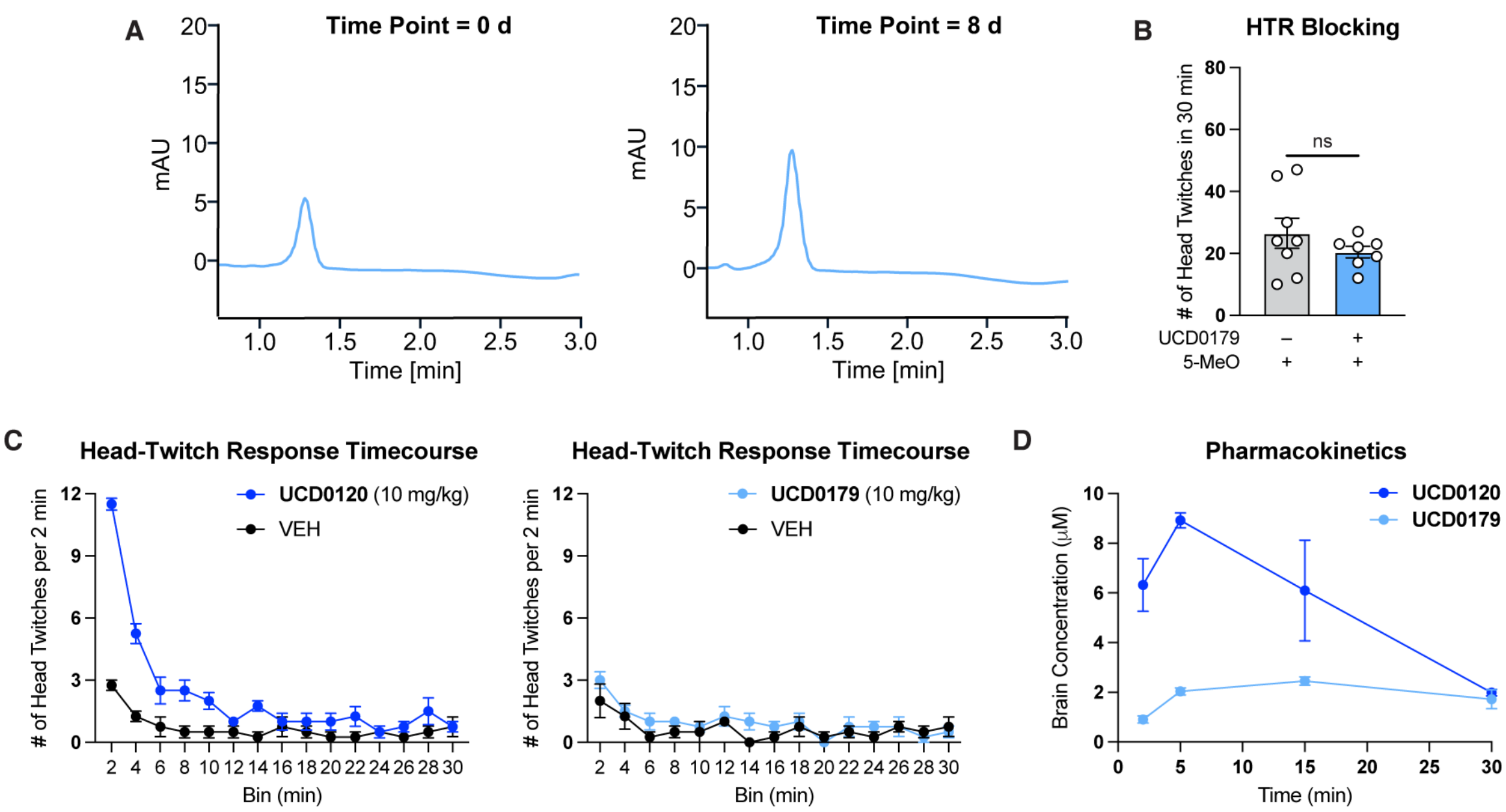

Figure S14. UCD00179 is not as brain penetrant as UCD0120. (A) In vitro studies demonstrate that **UCD0179** does not readily degrade when stored at room temperature as a 10 mM stock solution in DMSO. Purity was assessed using HPLC measuring absorbance at 260 nm immediately following preparation of the stock solution (Time Point = 0 d) and 8 days later (Time Point = 8 d). The chromatogram is shown starting from after solvent front artifacts (~0.75 min). No new peaks were observed after 8 days. (B) A magnetometer-based automated HTR assay was used to demonstrate that a 15 min pretreatment with **UCD0179** (10 mg/kg) could not block a HTR induced by 5-MeO (10 mg/kg). Data represent mean ± SEM. VEH/5-MeO: N = 8 mice (4 male, 4 female); **UCD0179**/5-MeO: N = 7 mice (4 male, 3 female). (C) Analyzing the HTR in 2 min bins revealed that the majority of head-twitches induced by **UCD0120** occur in the first 15 min following IP administration, while **UCD0179** does not produce head-twitches at any time point. (D) Pharmacokinetic studies measured brain concentrations of the compounds at 2, 5, 15, and 30 min after administration (10 mg/kg, IP). Concentrations in ng/mg were converted to molarities using 1.04 g/mL as the density of the mouse brain. Data represent mean ± SEM. N = 4 mice (2 male, 2 female) per group. ns = not significant, calculated using a Welch's t test. 5-MeO = 5-methoxy-*N,N*-dimethyltryptamine

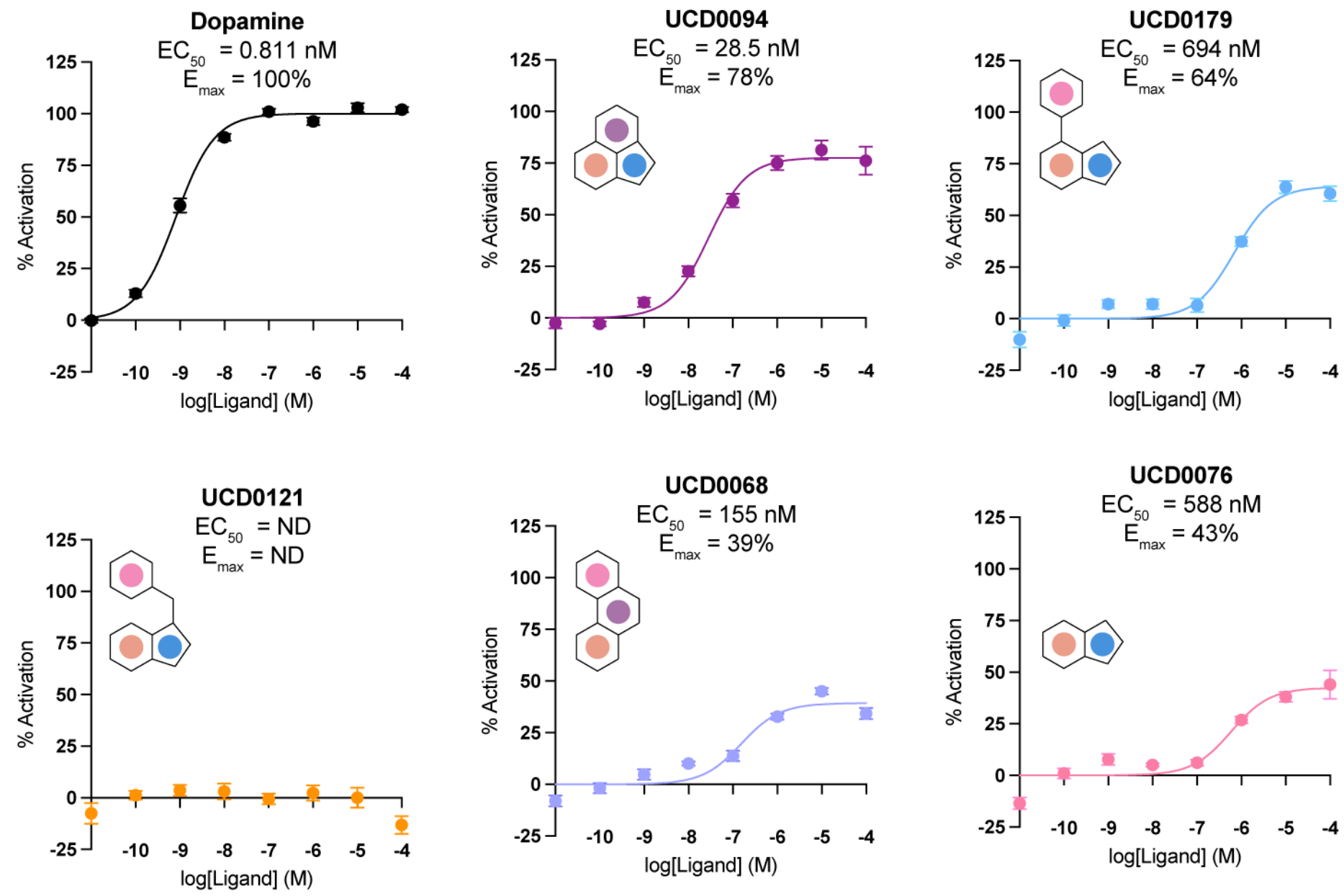

Figure S15. BRET-Based Assays of GoB Activation Following D2 Receptor Stimulation. Potency (EC_{50}) and efficacy (E_{max}) of GoB activation was assessed using BRET-based methods in HEK293T cells transiently expressing D2 receptors. Data represent 2 wells from 3–6 experimental replicates (i.e., independent cultures) and are normalized to the dopamine response (DA E_{max} = 100%; DA EC_{50} = 0.811 nM) with the bottom of the curves constrained to 0. N = 6 for all compounds. EC_{50} and E_{max} values were not determined (ND) for compounds that did not produce at least 30% activation at 10 μM. Schematics indicate structural components of the ergoline core present in each experimental compound.

	K_i (M)	pK_i	± SEM
**UCD0121**	7.53E-07	6.123	0.1107
**UCD0829**	ND	ND	ND
**UCD0094**	4.06E-08	7.391	0.09708
**UCD0076**	1.97E-06	5.706	0.1737
**UCD0830**	2.29E-06	5.64	0.1724
**UCD0179**	6.80E-08	7.167	0.1256
**UCD0120**	1.75E-07	6.757	0.0824
**UCD0068**	9.07E-07	6.042	0.1137
**UCD0162**	ND	ND	ND
**LSD**	5.48E-09	8.261	0.1082
**5-HT**	1.03E-08	7.988	0.08785
**(–)-UCD0094**	ND	ND	ND
**(+)-UCD0094**	4.43E-08	7.354	0.06412

Table S1. Binding Affinity for the psychLight2 5-HT2A receptor. (A) Radioligand competition binding assays using membrane preparations from PSYLI2 cells (i.e., HEK293T cells stably expressing the 5-HT2A-based biosensor psychLight2) were performed to determine K_i values. K_i values were not determined (ND) for compounds that did not produce at least 75% inhibition at 10 μM.

	5-HT2A					5-HT2B					5-HT2C				
	EC_{50}	pEC_{50}	± SEM	E_{max}	± SEM	EC50	pEC_{50}	± SEM	E_{max}	± SEM	EC50	pEC_{50}	± SEM	E_{max}	± SEM
**UCD0121**	6.00E-08	7.222	0.1529	66.48	4.183	2.77E-08	7.558	0.183	49.44	3.139	1.82E-07	6.739	0.1913	84.79	7.46
**UCD0829**	ND	ND	ND	ND	ND	ND	ND	ND	ND	ND	1.66E-06	5.78	0.4428	43.05	12.63
**UCD0094**	8.09E-09	8.092	0.1612	51.65	2.969	2.28E-08	7.642	0.2195	32.8	2.663	1.11E-08	7.957	0.1667	79.82	4.867
**UCD0076**	ND	ND	ND	ND	ND	1.63E-06	5.788	0.1361	44.49	3.657	3.34E-07	6.476	0.1219	82.96	4.89
**UCD0830**	ND	ND	ND	ND	ND	ND	ND	ND	ND	ND	1.67E-07	6.778	0.1934	52.13	4.619
**UCD0179**	5.90E-09	8.229	0.1197	81.63	3.345	8.44E-09	8.073	0.07291	67.95	1.677	2.43E-08	7.614	0.1739	97.45	6.276
**UCD0120**	7.48E-09	8.126	0.1288	63.18	2.876	2.21E-08	7.656	0.114	64.17	2.704	1.80E-08	7.744	0.2056	88.89	6.747
**UCD0068**	1.04E-07	6.982	0.07722	60.04	2.061	9.25E-09	8.034	0.1328	60.69	2.913	1.76E-07	6.755	0.1699	89.42	6.975
**UCD0162**	ND	ND	ND	ND	ND	ND	ND	ND	ND	ND	ND	ND	ND	ND	ND
**LSD**	5.54E-10	9.257	0.05079	95.73	1.447	5.48E-10	9.261	0.1028	71.28	2.178	6.21E-10	9.207	0.134	100.5	4.066
**(–)-UCD0094**	3.05E-07	6.516	0.06364	56.78	1.481	8.56E-08	7.068	0.1953	40.74	3.072	1.20E-07	6.922	0.1147	96.05	4.371
**(+)-UCD0094**	1.34E-08	7.873	0.06667	67.27	1.512	8.85E-08	7.053	0.1859	46.05	3.317	5.27E-08	7.279	0.1432	77.61	4.011

Table S2. BRET-Based Assays of Gq Activation Following 5-HT2 Receptor Stimulation. Potency (EC_{50}) and efficacy (E_{max}) of Gq activation was assessed using BRET-based methods in HEK293T cells transiently expressing 5-HT2 receptors. EC_{50} and E_{max} values were not determined (ND) for compounds that did not produce at least 30% activation at 10 μM.

	EC_{50}	pEC_{50}	± SEM	E_{max}	± SEM
**UCD0179**	1.01E-07	6.994	0.07806	72.82	2.069
**UCD0121**	2.58E-07	6.588	0.1077	44.3	1.77
**UCD0094**	4.94E-08	7.306	0.139	36.82	1.71
**UCD0076**	ND	ND	ND	ND	ND
**UCD0068**	8.30E-08	7.081	0.1765	44.88	2.833
**5-HT**	1.51E-08	7.822	0.06043	100	NA

Table S3. BRET-Based Assays of β-Arrestin2 Recruitment Following 5-HT2A Receptor Stimulation. Potency (EC_{50}) and efficacy (E_{max}) of Gq activation was assessed using BRET-based methods in HEK293T cells transiently expressing 5-HT2 receptors. EC_{50} and E_{max} values were not determined (ND) for compounds that did not produce at least 30% activation at 10 μM. NA = not applicable

	EC_{50}	pEC_{50}	± SEM	E_{max}	± SEM
**UCD0179**	6.94E-07	6.159	0.1035	64.09	2.796
**UCD0121**	ND	ND	ND	ND	ND
**UCD0094**	2.85E-08	7.545	0.08726	77.63	2.149
**UCD0076**	5.88E-07	6.231	0.1722	42.51	3.002
**UCD0068**	1.55E-07	6.81	0.1374	39.3	1.993
**DA**	8.11E-10	9.091	0.03294	100	NA

Table S4. BRET-Based Assays of GoB Activation Following D2 Receptor Stimulation. Potency (EC_{50}) and efficacy (E_{max}) of Gq activation was assessed using BRET-based methods in HEK293T cells transiently expressing 5-HT2 receptors. EC_{50} and E_{max} values were not determined (ND) for compounds that did not produce at least 30% activation at 10 μM. NA = not applicable

	EC_{50}	pEC_{50}	± SEM	E_{max}	± SEM
**5-HT1A**	8.29E-07	6.081	0.07057	95.71	3.97
**5-HT1B**	7.87E-07	6.104	0.08795	84.23	4.304
**5-HT1D**	1.34E-07	6.872	0.1089	78.96	3.898
**5-HT1e**	6.06E-07	6.217	0.08151	99.34	4.428
**5-HT1F**	4.88E-07	6.312	0.09803	80.08	4.081
**5-HT4**	ND	ND	ND	ND	ND
**5-HT5A**	ND	ND	ND	ND	ND
**5-HT6**	ND	ND	ND	ND	ND
**5-HT7**	ND	ND	ND	ND	ND

Table S5. BRET-Based Assays of Gα Activation Following 5-HT Receptor Stimulation. Potency (EC_{50}) and efficacy (E_{max}) of Gq activation was assessed using BRET-based methods in HEK293T cells transiently expressing 5-HT receptors. EC_{50} and E_{max} values were not determined (ND) for compounds that did not produce at least 30% activation at 10 μM.

1. Materials and Methods

Conformer Optimization and Receptor Docking. All structures were converted from 2D SMILES into 3D structures through Avogadro (v.1.2.0)[1] and initial MMFF92[2] geometry optimization was performed in Avogadro. The structures were then further optimized using eXtended Tight-Binding (xTB) (v6.7.1).[3] The structures were then exported as plain text (.xyz) files and submitted to Conformer–Rotamer Ensemble Sampling Tool (CREST) (v3.0.2)[4] for conformer sampling using GNF2-xTB[3] calculations in an implicit water solvent model.[5] Anywhere between 4 and 292 conformers were generated and optimized for each of the 9 ergologs. The minimum energy conformers were visualized in PyMol (v3.1.6.1).[6] From each library, the minimum energy conformer and three randomly selected conformers (n = 4) were loaded into AutoDock Tools (v1.5.6)[7], where polar hydrogens were added, non-polar hydrogens were merged, torsion trees and rotatable bonds were identified, and Gasteiger chargers were assigned. Each structure was then exported as a PDBQT file. These compounds were then docked through AutoDock Vina (v.1.1.2)[8] to the 5-HT2A protein (PDB: 9AS4)[9] with the ligand removed. The binding site of the protein was centered at x=138.161, y=143.440, x=171.142 and was defined by a 14 x 14 x 14 grid with a spacing of 1.000 Å. An exhaustiveness factor of 32 was used for docking. AutoDock Vina generated 9 binding poses for each conformer, for a total of 36 binding poses for each of the ergologs. For each of these poses, the distance between the basic amine (N) and aspartic acid 155 (D155) was measured. As a salt bridge with D155 is required for activation of the receptor, only the binding poses where the basic nitrogen was within 4 Å of D155 were considered valid. Root mean square deviation (RMSD) was calculated for the top 4 poses of each compound – these being identified as the binding poses with the lowest energies/highest affinities after considering the proximity of the basic nitrogen to D155. RMSD was calculated by noting the common atoms between the ergologs bound to the pocket and comparing these to the same atoms of (+)-LSD in the crystal structure binding pocket. The closer the atoms were located to one another in space, the lower the RMSD score, with 0 being perfect overlap of atoms. The highest affinity binding poses were visualized in PyMol. The values associated with the top 4 poses of each compound (conformer number/binding pose, binding affinity, distance between the basic nitrogen and D155, and RMSD) are reported.

Radioligand Binding Assays. Competition radioligand binding experiments were conducted as described previously.[10] Briefly, membrane preparations were generated from PSYLI2 cells (HEK293T cell line stably expressing psychLight2). Millipore MultiScreen HTS FB filter plates were pre-soaked with 0.3% polyethylenimine$_{(aq)}$ (PEI) for at least 30 min. All compounds and radioligands were diluted in binding buffer (50 mM Tris HCl, 10 mM $MgCl_2$, and 0.1 mM EDTA disodium dihydrate in autoclaved deionized water). Competition radioligand binding experiments were conducted at 25ºC and initiated by the addition of [^{3}H]-LSD (5 nM final concentration; K_d = 5.2 ± 2.6 nM; B_{max} = 2.2 ± 0.26 fmol/µg), membrane preparations (10 µg/well), and unlabeled compounds (0 – 10 µM final concentration). The assays were terminated after 60 min by rapid vacuum filtration followed by three rapid washes with 200 µL of ice-cold binding buffer. Dried filter screens from each well were then transferred to 7 mL scintillation vials containing 1 mL PerkinElmer Ultima Gold liquid scintillation cocktail. Data from two individual wells were excluded due to technical issues during transfer of the filters to the scintillation cocktail. Amount of radioactivity was determined using a Beckman LS 6000 liquid scintillation counter. Data were analyzed using the One site – Fit K_i nonlinear regression in GraphPad Prism. The 0.1% DMSO vehicle condition was set to an arbitrarily dilute concentration of 10^{-25} M to be included on the logarithmic scale of the X-axis. Data were normalized to VEH (100%) and 10 µM 5-HT (0%) plate controls followed by baseline correction to set the top of the curve to 100% radioligand bound.

PsychLight2 Experiments. The inner 60 wells of glass bottom 96-well plates (P96-1.5H-N, CellVis) were coated with 50 μg/mL of poly-D-lysine (Sigma, P7886-500MG) in UltraPure distilled water (DNAse, RNAse, free, Life Technologies, 10977-015) overnight at ambient temperature. The outer 36 wells were filled with Dulbecco's PBS (ThermoFisher, 14190-136). Plates were washed twice with UltarPure distilled water (DNAse, RNAse, free, Life Technologies, 10977-015) and once with Dulbecco's PBS (ThermoFisher, 14190-136). PSYLI2 cells were suspended in DMEM (Fisher, 11995065) containing 10% FBS (Fisher, 10438-026) and plated at a density of 80,000 cells/well in the inner 60-wells of the 96-well plates 24 h prior to each experiment. The cells were then gently washed with imaging media three times, and the wells were filled with an appropriate volume of imaging media for the respective experiment (vide infra). The imaging media consisted of Gibco™ HBSS containing 100 mg/L $MgCl_2$ and 140 mg/L $CaCl_2$ (Fisher 14025-076) and buffered with 5% 1M HEPES (ThermoFisher, 15630-080) and 1M NaOH. Prior to experiments, stock solutions of drugs in DMSO (10 mM) were serially diluted in imaging media to prepare the 8-point concentrations of the concentration-response.

PsychLight Agonist Mode Experiments. For agonist mode experiments, 180 μL of imaging media were added to each of the inner 60 wells of the assay plate. Wells were then imaged either on a ThermoScientific CELLINSIGHT CX7 LZR Pro HCS platform or CX7 LED Pro platform at 20x with 9 fields of interest (FOI) taken per well using a spiral 9 FOI pattern for each well with no bias to location, no overlap of the FOIs, and a distance of at least 1 field in between each imaged FOI (exposure = 0.05s, LED power = 80%). Next, 20 μL from the treatment plate was transferred to the assay plate containing a 1:1000 dilution of drug (final concentration response consisted of the following final concentrations in imaging media with 0.1% DMSO: 1 μM, 100 nM, 10 nM, 1nM, 100 pM, 10 pM, 1 pM, 100 fM). The highest treatment concentration of 1 μM was chosen to avoid potential confounding fluorescence interference often observed with compounds at 10 μM and above. As positive and vehicle controls, 5-HT•HCl (10 μM), and DMSO (0.1%) in imaging media were used, respectively. Treatments were allowed to incubate for 5 min, then the same sites were re-imaged using the same settings. Once imaging was complete, the images were exported as TIFF files and processed using a custom Python script to obtain %ΔF/F values for each ROI from each well (https://github.com/moagonzalez/OlsonLab/blob/main/PsychLightV3_Clean.txt). After analysis, outliers were removed by performing a ROUT test (Q = 1%) in GraphPad Prism and the responses were averaged to obtain the average %ΔF/F for each well. On each assay plate, experiments were performed in duplicate (technical replicates that were averaged), and a total of 3 plates prepared using independent PSYLI2 cultures were utilized (3 biological replicates). The average response per treatment was normalized so that the positive control (10 μM 5-HT) was set as 100% and the vehicle (0.1% DMSO) as 0%. Data for two replicates were obtained using the ThermosScientific CellInsight CX7 LED Platform, and data for one replicate were obtained using the ThermoScientific CellInsight CX7 LZR Pro HCS Platform.

PsychLight Antagonist Mode Experiments. For antagonist mode experiments, 160 μL of imaging media was added to the inner 60 wells of the assay plate. Wells were then imaged on ThermoScientific CELLINSIGHT CX7 LZR Pro HCS platform or CX7 LED Pro platform at 20x with 9 fields of interest (FOI) taken per well using a spiral 9 FOI pattern for each well with no bias to location, no overlap of the FOIs, and a distance of at least 1 field in between each imaged FOI (exposure = 0.05s, LED power = 80%). As positive and vehicle controls, 5-HT (100 nM), and DMSO (0.1%) in imaging media were used, respectively. To prepare the positive control, 10 mM 5-HT•HCl stock solution in DMSO was diluted 1:10,000 in imaging media. Treatments were prepared in concentration response from 10 mM stocks in DMSO by diluting into imaging media. Final concentration response consisted of the following final concentrations in imaging media (0.1% DMSO): 10 μM, 1 μM, 100 nM, 10 nM, 1nM, 100 pM, 10 pM, 1 pM. Wells containing these treatments were prepared with both 100 nM 5-HT and the treatment. To accomplish this, 20 μL

of the 5-HT solution was concomitantly added to the assay plate with 20 μL of the appropriate treatment for a final concentration of 100 nM 5-HT (0.1% DMSO) and treatment. The same 9 FOIs were imaged after 5 min of incubation. Once imaging was complete, the images were exported as TIFF files and analyzed using a custom python script (https://github.com/moagonzalez/OlsonLab/blob/main/PsychLightV3_Clean.txt) to obtain %ΔF/F values for each ROI from each well. After analysis, outliers were removed by performing a ROUT test (Q = 1%) in GraphPad Prism and the responses were averaged to obtain the average %ΔF/F for each well. On each assay plate, experiments were performed in duplicate (technical replicates that were averaged), and a total of 4 plates prepared using independent PSYLI2 cultures were utilized (4 biological replicates). The average response per treatment was normalized so that the positive control (10 μM 5-HT) was set as 100% and the vehicle (0.1% DMSO) as 0% and plotted. Data for two replicates were obtained using the ThermoScientific CellInsight CX7 LED Pro Platform, and data for two replicates were obtained using the ThermoScientific CellInsight CX7 LZR Pro HCS Platform.

5-HT2 Gq dissociation BRET Assays. To measure 5-HT2 receptor-mediated Gq activation via Gq/γ9 dissociation as measured by $BRET^2$, experiments were performed as described previously[11] with slight modifications. First, HEK293T cells were subcultured in DMEM supplemented with 10% dialyzed FBS and were co-transfected using TransiT-2020 in either a 1:1:1:1 ratio (for 5-HT_{2A}) or 1:2:2:2 ratio (for 5-HT_{2B} and 5-HT_{2C} INI) with receptor:*R*Luc8-fused human Gαq (Gαq- *R*Luc8):human Gβ3:GFP^2-fused to the C-terminus of human Gγ9 (GFP^2-Gγ9). Cells were transfected using a reverse transfection method, where transfection mixtures were added to cells resuspended in 1% dialyzed FBS (dFBS) and plated at 25-40,000 cells/well (200 μL) in poly-L-lysine-coated 96-well white assay plates (Grenier Bio-One). Approximately 48 hours after plating, media was decanted from plates and drug buffer (1 x HBSS, 20 mM HEPES, pH 7.4) was added via a Multidrop (60 μL/well; ThermoFisher Scientific). Drug dilutions for all compounds were prepared in McCorvy buffer (1 x HBSS, 20 mM HEPES, pH 7.4, supplemented with 0.3% BSA fatty acid free (GoldBio), and 0.03% ascorbic acid). Following a 15 min equilibration at 37ºC in a humidified incubator, plates received drug stimulation (30 μL/well). Plates were then incubated at 37ºC in a humidified incubator for total of 60 min of drug incubation time. Coelenterazine 400a (5 μM final concentration; Nanolight Technology) was added to plates (10 μL/well) approximately 15 min prior to reading on a PheraStar FSX or ClarioStar Plus (BMG Lab Tech). Plates were read at 410 nm (*R*Luc8) and 515 nm (GFP^2) emission filters for 1.0 second per well. BRET ratios of GFP^2/*R*Luc8 luminescence were calculated per well and plotted as a function of drug concentration using GraphPad Prism 10 (GraphPad Software Inc., San Diego, CA). Data were normalized to the serotonin response (5-HT E_{max} = 100%) with the bottom of the curves constrained to 0 and analyzed using nonlinear regression "log(agonist) vs. response (fold, span)" to yield E_{max} and EC_{50} parameter estimates. The expression of 5-HT2 receptors using these transfection conditions were comparable as determined by saturation binding assays utilizing [^{3}H]LSD (5-HT2A B_{max} = 1,500 fmol/mg protein; 5-HT2B B_{max} = 1,200 fmol/mg protein; 5-HT2C B_{max} = 1,900 fmol/mg protein).

β-Arrestin2 Recruitment BRET Assays. β-arrestin recruitment BRET assays were conducted using $BRET^2$ with 5-HT2A fused to *R*luc8 at the C-terminus and GFP^2 fused to the N-terminus of human β-arrestin2 in pcDNA3.1 vectors. Constructs were expressed in in a 1:15 ratio (5-HT2A-*R*luc8: GFP^2-hβ-arrestin2) in HEK293T cells (ATCC CRL-11268; mycoplasma-free), which were subcultured in high-glucose DMEM (VWR) supplemented with 10% FBS (Life Technologies). Approximately 24 hours after expression assays, cells were plated at an approximate density of 40-50,000 cells per well into poly-L-lysine-coated 96-well white assay plates (Grenier Bio-One). On the day of the assay, plates were decanted and 60 μL of drug buffer per well (HBSS, 20 mM HEPES, pH = 7.4) was added using a Multidrop (ThermoFisher Scientific), and plates were

allowed to equilibrate at 37°C in a humidified incubator before receiving drug stimulation. Drug dilutions (3X) of all compounds were performed in McCorvy buffer (HBSS, 20 mM HEPES, pH = 7.4, supplemented with 0.3% BSA fatty acid free (GoldBio), and 0.03% ascorbic acid). Next, plates were incubated at 37°C in a humidified incubator for total time of 60 min, except 15 min before reading, plates were taken out and coelenterazine 400a (5 μM final concentration; Nanolight Technology) was added. Immediately after 60 min incubation, plates were read at 400 nm Rluc and 510 nm emission filters for using a ClarioStarPlus (BMG Lab Tech, Cary NC). The BRET ratios of 510/400 luminescence were calculated per well and were plotted as a function of drug concentration using Graphpad Prism 11 (Graphpad Software Inc., San Diego, CA). Data were normalized to % 5-HT stimulation and analyzed using nonlinear regression “log(agonist) vs. response” to yield E_{max} and EC_{50} parameter estimates.

Automated HTR. Female C57BL/6J mice 9 weeks of age (4 per treatment group) were induced in a chamber using 5% isoflurane, then transferred to a stereotaxic apparatus (RWD) where the head was fixed using ear bars under constant 2% isoflurane anesthesia. Following aseptic technique and institutional IACUC approved procedures, an incision was made to expose the cranium. After thoroughly cleaning the surface, a small 5x2 mm neodymium magnet (TRYMAG) was affixed to the skull using a dental cement headcap (Parkell). Mice were allowed 7 days to recover before beginning experiments. Attrition occurred for some cohorts when magnet implants fell out (N = 1, **UCD0829**), or when incorrect treatments were mistakenly given (N =1, **UCD0121** and **UCD0076**). Data from these mice were excluded from analysis in the following weeks. Magnetometers and subsequent analysis steps were constructed similarly to a previous report.[12] In brief, a small glass chamber was encircled with insulated magnetic wire to make a coil. Each wire end of the coil was connected to a Powerlab/16SP (ADInstruments) acquisition device through copper HiFi speaker cables (AutCreation). Mice were placed in the chamber with a plastic lid covering the top for 30 min of recording. On the first week of experiments, mice were administered a negative control of 5 mL/kg saline. In each subsequent week, escalating salt-corrected doses of the described compounds were given to the mice (one compound per mouse) with a minimum washout period of 6 days. Finally, each mouse was given a 10 mg/kg salt-corrected dose of 5-MeO-DMT (i.e., the dose of the freebase was equal to 10 mg/kg). Labchart software (v7.3.8, ADInstruments) was used to record the magnetometer traces of 6 mice simultaneously. These traces were then analyzed using a waveform analysis MATLAB (r2023a) script published previously.[12] The script extracts waveform features using a convolutional neural network, then classifies events using a support vector machine algorithm with accuracy parallel to human classification.[12] The code produced the number of HTR during each 30 minute trial, which we used to compare incidence of the HTR across treatments groups.

Automated HTR Blocking Studies. These studies were conducted as above with the exception that male and female mice were used and VEH or salt corrected doses of **UCD0076** or **UCD0179** (i.e, dose based on the weight of the freebase) were administered 15 min prior to 5-MeO (10 mg/kg, salt corrected). Data were recorded for 30 min following the administration of 5-MeO.

Amphetamine-Induced Locomotion. An equal number of ~10-week-old male and female C57BL/6J mice (4 male, 4 female, N = 8 per group) were used for these experiments. A basal reading of locomotor activity was obtained by first placing the animals in the test arena (40 cm x 40 cm) for 15 min. Animals were then administered **UCD0076** (1, 10, and 50 mg/kg uncorrected = 0.6, 6, 31 mg/kg salt corrected) or vehicle (0.9% saline, 5 mL/kg, i.p) and placed back in the test arena for 15 min. Next, the animals were administered *S*-amphetamine hemisulfate (3 mg/kg) or vehicle (5 mL/kg) and placed back in the test arena for 60 min. Locomotion during the entire experiment was quantified using ANYmaze Video Tracking System, version 7.10 (Stoelting Co.).

Stability studies on DMSO stock solutions of UCD0179. Degradation of **UCD0179** was monitored using an Agilent 1260 III HPLC. Samples were run on an InfinityLab Poroshell 120 EC-C18 column (3.0 x 100 mm, 2.7 μm) heated to 40ºC. Flow rate was set to 0.6 mL/min. The solvent system consisted of a linear gradient of H_2O:MeCN (80:20) from 0 to 0.5 min, followed by gradient elution utilizing H_2O:MeCN (80:20 to 5:95) from 0.5 to 3 min, then a linear gradient of H_2O:MeCN (5:95) from 3 to 6 min, and returning to 80:20 H_2O:MeCN from 6 to 7 min. All solvents contained 0.1% (v/v) formic acid. All wavelengths were collected, and 260 nm was selected for analysis due to the largest response. Data processing was performed on OpenLab CDS.

Pharmacokinetics. Samples were prepared as describe previously.[13] Briefly, mice (4M, 4F) aged 9–24 weeks old were administered 10 mg/kg (salt-corrected) of either **UCD0120**, or **UCD0179**. Fifteen minutes after administration, each mouse underwent live cervical dislocation followed by rapid decapitation. Their brains were removed and and transferred to 2 mL microcentrifuge tubes before being flash frozen in liquid nitrogen. Samples were stored in -80ºC prior to analysis. Tissue samples (around 200 mg) were added to a 2 mL amber Eppendorf tube containing 1.5 mL of ice-cold acetonitrile/methanol/water (2:2:1, v/v/v) with 37.5 µL of 0.1 M ascorbic acid containing 1 ng of DMT-D4 (Supelco) spiked into each sample as internal standard. Samples were homogenized at -20°C with three 3 mm metal beads using a GenoGrinder 2010 (SPEX SamplePrep). The tissue was homogenized three times for 20 s at 1500 rpm, with a 30 s interval between homogenizations. Homogenates were then extracted and sonicated in an ice bath for 15 min, followed by centrifugation for 2 min at 14000*g* to precipitate proteins. The resulting supernatant was transferred and evaporated to dryness using a CentriVap centrifugal vacuum concentrator (Labconco). The dried extracts were reconstituted in 140 µL of methanol/water (1:1, v/v). After centrifugation for 2 min at 14,000*g*, the samples were transferred to a high-performance liquid chromatography (HPLC) vial with an insert ready for analysis. Instrumental analysis was performed using a Vanquish UHPLC system coupled to a SCIEX QTRAP 6500+ LC–MS/MS system (SCIEX). A Thermo Scientific Accucore Biphenyl Reverse Phase HPLC column (particle size 2.6 µm, i.d. 2.1 × length 100 mm) coupled with an Accucore Defender Guard Column (particle size 2.6 µm, i.d. 2.1 × 10 mm). For **UCD0179** and **UCD0120**, a gradient was used at a flow rate of 0.3 mL/min with a column temperature set to 40°C. The linear gradient was set as follows: 0–1.5 min: 4% B; 1.5 to 2.5 min: 4% B to 20% B; 2.5 to 5.5 min: 20% B to 40% B; 5.5 to 7.5 min: 40% B to 60% B; 7.5 to 8.5 min: 60% B to 90% B; 9.5 to 9.6 min, 90% B to 4% B; 9.6 to 11 min: 4% B (hold), with 2.0 min used as a postrun equilibrium. The mobile phase was comprised of 5 mM of ammonium acetate in HPLC water (A) and 100% methanol (B). Other parameters were set as follows: temperature 500 °C, curtain gas 35 psi, ion spray voltage 5500 V, nebulizer gas: 50 psi, and heater gas: 40 psi. The MS/MS was operated in the Scheduled MRM Pro Algorithm in positive polarity mode. Parameters were optimized by infusion of individual analytes as shown in **Table S6** including representative traces. PBS was used as the procedural blanks. A QC sample was prepared by pooling all of the treated and control samples. QC analysis was conducted once for every six injections of biological samples and a blank sample (methanol:water, 1:1, v/v). A seven-point calibration curve was prepared by spiking 1 ng of DMT-D4 and varying amounts of native molecules: **UCD0179** and **UCD0120** 0.02, 1.5, 2.5, 5, 10, 20, and 30 ng. Peak picking and integration were performed by using SCIEX MultiQuant 3.0 software. Linearity was evaluated by plotting analyte-to-internal standard peak area ratios against nominal concentrations, and the instrumental detection limits are summarized in **Table S7**.

2. General Synthetic Procedures

General Procedure A: Preparation of Amine Salts

One of three acids was utilized for salting: fumaric acid, p-toluenesulfonic acid hydrate, or hydrochloric acid. Facilitated by heating and sonication, acid (0.9 equiv.) was dissolved in a minimal amount of solvent. The solution was then allowed to cool to ambient temperature. Basic amine (1.0 equiv.) was dissolved in a minimal amount of solvent then added dropwise into a solution of dissolved acid. The mixture was stored at ambient temperature for 30 min then at -22 °C for 14 h. The mixture was filtered and the solid was washed with cold solvent. The solid was collected and dried under high vacuum at 50 °C for 18 h. Integration of the ^{1}H NMR spectrum was utilized to verify the salting ratio and removal of residual solvent. General Procedure B was followed if solvent was observed in the crystallized solids.

General Procedure B: Removal of Trapped Solvent

If residual solvent was observed in the crystallized solid by ^{1}H NMR, the solid was redissolved in a minimal amount of isopropanol (IPA) and HPLC grade water (50:50). Then, the solution was concentrated under reduced pressure at 50 °C until all solvent was removed. Additional IPA was added as needed, and the azeotrope was removed under reduced pressure. Next, pentane was added, and the mixture was sonicated before being concentrated under reduced pressure. The solid was stored under high vacuum at 50 °C for 18 h. ^{1}H NMR was then utilized to verify that residual solvent had been removed.

3. Detailed Synthesis Procedures and Experimental Data for all Compounds

Experimental Procedures for the Synthesis of UCD0094

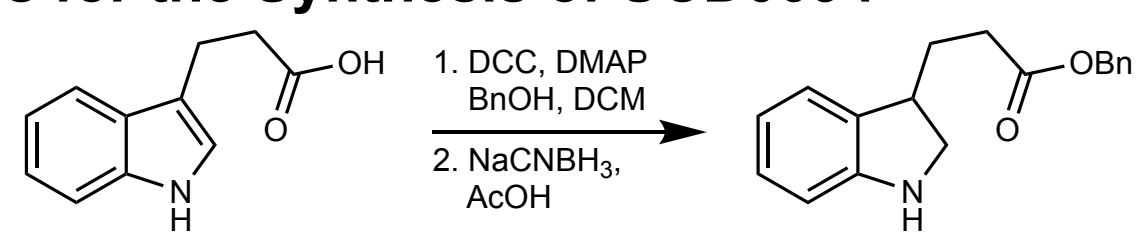

1 **2**

Benzyl 3-(indolin-3-yl) propanoate (2)

To a stirred solution of **indole-3-propanoic acid** (**1**) (50.0 g, 265 mmol, 1.00 equiv.), DCC (57.2 g, 277 mmol, 1.05 equiv.), and DMAP (16.1 g, 132 mmol, 0.500 equiv.) in DCM (529 mL) was added benzyl alcohol (28.9 mL, 277 mmol, 1.05 equiv.). The solution was stirred for 22 h before being filtered. The filtrate was collected and washed sequentially with 2 M HCl (175 mL), 1 M NaOH (175 mL), and brine (100 mL). The organic layer was dried over Na_2SO_4, filtered, and the filtrate was concentrated under reduced pressure to yield a white solid, which was used without further purification. The solid was split into two equal batches, and each batch was subjected to the following conditions, then combined. To a solution of unpurified white solid in acetic acid (640 mL) was added $NaCNBH_3$ (38.8 g, 537 mmol, 4.0 equiv.) over three equivalent potions. The reaction mixture was stirred for 3 h before being poured into chilled H_2O (1.50 L). The solution was extracted with DCM (3 x 200 mL). The combined organic extracts were washed with saturated aqueous $NaHCO_3$ (2 x 300 mL) and brine (300 mL). The organic layer was dried over Na_2SO_4, filtered, and then concentrated under reduced pressure to yield a crude oil. The oil was purified via chromatography on silica gel (gradient elution: 90:10 hexanes/EtOAc → 80:20 hexanes/EtOAc) to afford compound **2** (41.6 g, 59% over 2 steps) as a clear, pale-yellow oil.

R_f = 0.25 (75:25 hexanes/EtOAc)

^{1}H NMR (400 MHz, $CDCl_3$) δ (ppm) = 7.48 – 7.32 (m, 5H), 7.15 (d, *J* = 7.3z Hz, 1H), 7.09 (t, *J* = 7.6 Hz, 1H), 6.77 (t, *J* = 7.3 Hz, 1H), 6.68 (d, *J* = 7.6 Hz, 1H), 5.18 (s, 2H), 3.71 (t, *J* = 8.7 Hz, 1H), 3.63 (br s, 1H), 3.40 – 3.30 (m, 1H), 3.25 (dd, *J* = 8.6, 6.8 Hz, 1H), 2.57 – 2.42 (m, 2H), 2.27 – 2.15 (m, 1H), 2.02 – 1.90 (m, 8.4 Hz, 1H)
^{13}C NMR (101 MHz, CD_3OD) δ (ppm) = 173.52, 151.35, 136.26, 132.49, 128.21, 127.90, 127.86, 127.30, 123.57, 118.77, 110.17, 65.92, 52.37, 41.24, 31.44, 29.06
LRMS (ES$^+$) = *m/z* $[M + H]^+$ calcd. for $C_{18}H_{20}NO_2^+$, 282.15; found, 282.17
IR (Smart iTX Diamond) = ν 3377, 3030, 2925, 2855, 1725, 1606 cm-1

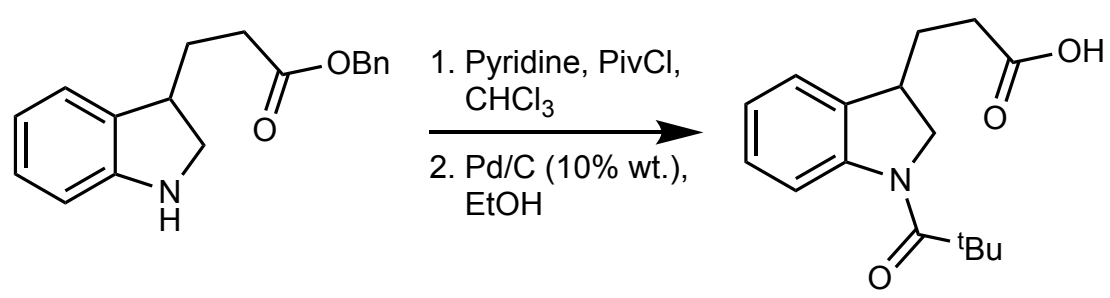

2 3

Benzyl 3-(1-pivaloylindolin-3-yl) propanoate (3)

To a stirred solution of compound **2** (20.8 g, 77.8 mmol, 1.0 equiv.) in chloroform (164 mL) at 0 °C was added pyridine (11.9 mL, 147 mmol, 1.89 equiv.) then pivaloyl chloride (10.3 mL, 83.6 mmol, 1.07 equiv.). The resulting solution was warmed to ambient temperature and stirred for 1 h before being quenched with 2 M HCl (75.0 mL). The organic layer was collected, washed with saturated aqueous $NaHCO_3$ (75 mL), then dried over Na_2SO_4. The mixture was filtered, and then concentrated under reduced pressure to yield oil. The oil (30.9 g) was used in the next step without further purification. To a solution of reduced 10 wt % Pd/C (3.00 g, 2.82 mmol, 0.0362 equiv. of Pd) in EtOH (50.0 mL) was added the unpurified oil (30.9 g, 73.9 mmol, 1.00 equiv.) in EtOH (380 mL). The solution was sparged with hydrogen gas for 10 min then stirred under a hydrogen atmosphere (1.00 atm) for 18.0 h. The mixture was filtered through celite, then concentrated under reduced pressure. The residue was purified via chromatography on silica gel (75:25 hexanes/Acetone) to afford compound **3** (18.0 g, 84% over 2 steps) as a white solid.

Note: The Pd/C catalyst was acquired from Acros Organics. If necessary, further purification may be achieved via recrystallization with Hexane/EtOAc (85:15).

R_f = 0.28 (75:25 hexanes/Acetone)
^{1}H NMR (600 MHz, $CDCl_3$) δ (ppm) = 8.21 (d, *J* = 8.3 Hz, 1H), 7.26 – 7.16 (m, 2H), 7.03 (t, *J* = 7.3 Hz, 1H), 4.28 (t, *J* = 9.4 Hz, 1H), 3.94 (dd, *J* = 10.1, 5.0 Hz, 1H), 3.38 – 3.29 (m, 1H), 2.44 (t, *J* = 7.7 Hz, 2H), 2.12 – 2.02 (m, 1H), 1.91 (m, 1H), 1.36 (s, 9H)
^{13}C NMR (150 MHz, $CDCl_3$) δ (ppm) = 178.60, 176.58, 144.32, 133.39, 128.09, 123.80, 123.74, 118.60, 55.33, 40.18, 40.16, 31.20, 29.17, 27.67
LRMS (ES$^+$) = *m/z* $[M + H]^+$ calcd. for $C_{16}H_{22}NO_3^+$, 276.16; found, 276.23
IR (Smart iTX Diamond) = ν 3100, 2971, 1732, 1708, 1643, 1619, 1588 cm^{-1}

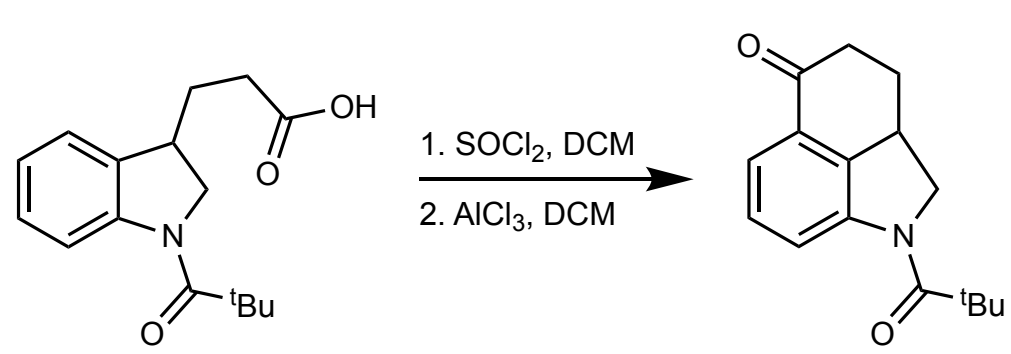

3 4

1-pivaloyl-2,2a,3,4-tetrahydrobenzo[cd]indol-5(1*H*)-one (4)

To a stirred solution of compound **3** (16.6 g, 60.3 mmol, 1.00 equiv.) in DCM (30.0 mL) at 0 °C was added thionyl chloride (8.70 mL, 120 mmol, 2.00 equiv.). The mixture was stirred for 50 min at 0 °C before being concentrated under reduced pressure. The residue was dissolved in DCM

(15.0 mL), and the solution was split into three equal volumes and added dropwise via addition funnel to three round bottom flasks containing granular $AlCl_3$ (8.10 g, 60.7 mmol, 3.03 equiv.) in DCM (60.0 mL) at 0 °C. The three solutions were stirred 18 h before being poured into a brine ice bath solution (200 mL). The aqueous layer was extracted with DCM (3 x 100 mL). Organic extracts were combined, washed sequentially with saturated aqueous $NaHCO_3$ (2 x 100 mL) and brine (150 mL). The organic layer was then dried over Na_2SO_4, filtered, and the filtrate was concentrated under reduced pressure. The residue was purified by chromatography on silica gel (gradient elution: 80:20 hexanes/EtOAc → 65:35 hexanes/EtOAc) to afford compound **4** (10.3 g, 66%) as a yellow solid.

R_f = 0.33 (50:50 hexanes/EtOAc)
^{1}H NMR (600 MHz, $CDCl_3$) δ (ppm) = 8.18 (d, *J* = 7.9 Hz, 1H), 7.47 (d, *J* = 7.9 Hz, 1H), 7.24 (t, *J* = 7.9 Hz, 1H), 4.64 (t, *J* = 9.0 Hz, 1H), 3.65 (t, *J* = 10.7 Hz, 1H), 3.59 – 3.50 (m, 1H), 2.72 (d, *J* = 17.6 Hz, 1H), 2.58 – 2.48 (m, 1H), 2.40 – 2.33 (m, 1H), 1.89 (qd, *J* = 13.0, 4.0 Hz, 1H), 1.33 (s, 9H)
^{13}C NMR (150 MHz, $CDCl_3$) δ (ppm) = 196.51, 176.56, 143.78, 140.22, 129.21, 128.79, 122.73, 120.05, 57.04, 39.88, 38.34, 37.75, 28.32, 27.53
LRMS (ES$^+$) = *m/z* $[M + H]^+$ calcd. for $C_{16}H_{20}NO_2^+$, 258.15; found, 258.21
IR (Smart iTX Diamond) = ν 2969, 2873, 2246, 1682, 1641, 1589 cm^{-1}

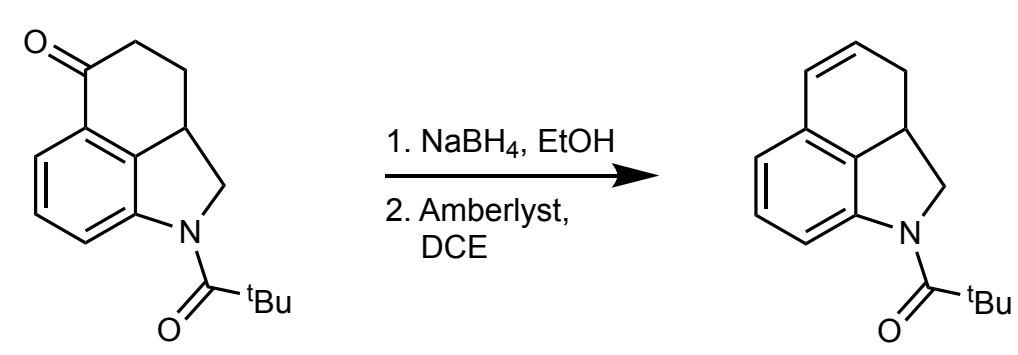

4 5

1-(2a,3-dihydrobenzo[cd]indol-1(2*H*)-yl)-2,2-dimethylpropan-1-one (**5**)
To a stirred solution of compound **4** (10.2 g, 39.6 mmol, 1.00 equiv.) in EtOH (200 mL) at a 0 °C was added $NaBH_4$ (1.50 g, 39.6 mmol, 1.00 equiv.). The solution was warmed to ambient temperature after 15 min and stirred for 2 h before being concentrated under reduced pressure. Deionized water (100 mL) was added to the residue. The aqueous layer was extracted with DCM (3 x 50 mL). The organic extracts were combined, washed with brine (100 mL), dried over Na_2SO_4, filtered, and then concentrated under reduced pressure. The residue was used without further purification. To a solution of the residue (10.2 g, 39.2 mmol, 1.00 equiv.) in DCE (390 mL) was added Amberlyst15(H), wet, ion exchange resin catalyst (10.0 g). The mixture was heated to 65 °C and stirred for 3 h. The mixture was filtered, then concentrated under reduced pressure. The residue was purified by chromatography on silica gel (75:25 hexanes/EtOAc) to afford compound **5** (8.87 g, 93% over two steps) as a lime green solid.

R_f = 0.50 (75:25 hexanes/EtOAc)
^{1}H NMR (600 MHz, $CDCl_3$) δ (ppm) = 7.92 (d, *J* = 8.1 Hz, 1H), 7.14 (t, *J* = 7.8 Hz, 1H), 6.77 (d, *J* = 7.5 Hz, 1H), 6.53 (dd, *J* = 9.7, 1.9 Hz, 1H), 6.01 – 5.95 (m, 1H), 4.68 (t, *J* = 9.0 Hz, 1H), 3.70 (t, *J* = 10.4 1H), 3.55 – 3.45 (m, 1H), 2.58 (dt, *J* = 16.0, 7.2 1H), 2.13 (t, *J* = 16.0 Hz, 1H)
^{13}C NMR (150 MHz, $CDCl_3$) δ (ppm) = 176.50, 142.76, 130.75, 129.28, 128.46, 127.68, 126.58, 119.59, 117.13, 58.31, 39.87, 35.12, 27.99, 27.64
LRMS (ES$^+$) = *m/z* $[M + H]^+$ calcd. for $C_{16}H_{20}NO^+$, 242.15; found, 242.31
IR (Smart iTX Diamond) = ν 3031, 2969, 2931, 2872, 2243, 1641, 1624, 1569 cm^{-1}

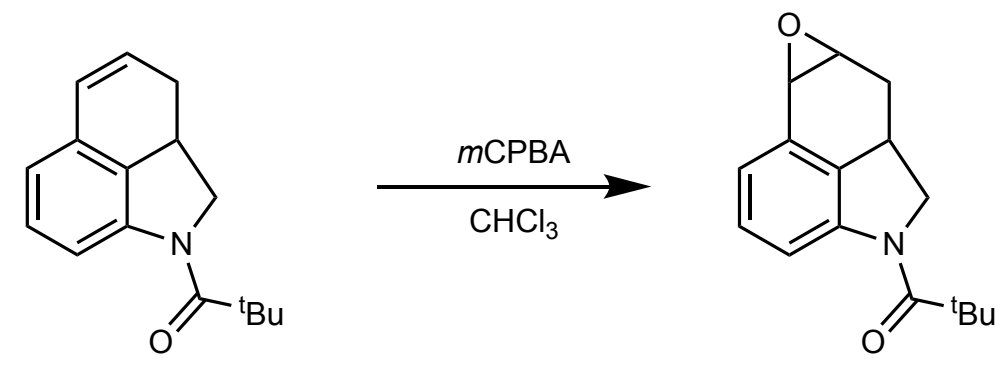

5 6

2,2-dimethyl-1-(5a,6,6a,7a-tetrahydrooxireno[2',3':4,5]benzo[1,2,3-cd]indol-4(5*H*)-yl)propan-1-one (**6**)

To a stirred solution of compound **5** (8.87 g, 36.8 mmol, 1.0 equiv.) in $CHCl_3$ (292 mL) at 0 °C was added 70–75% m-CPBA (9.07 g, 40.5 mmol, 1.20 equiv.). The solution was stirred for 3 h at ambient temperature before being quenched with NaOH (100 mL). The organic layer was collected, and the aqueous layer was further extracted with DCM (2 x 60 mL). The organic extracts were combined, washed with brine (80 mL), dried over Na_2SO_4, filtered, and then concentrated under reduced pressure. The residue was purified by chromatography on silica gel (75:25 hexanes/EtOAc) to afford compound **6** (8.29 g, 88%) as a white solid.

R_f = 0.25 (75:25 hexanes/EtOAc)
^{1}H NMR (600 MHz, $CDCl_3$) δ (ppm) = 8.05 (d, *J* = 8.1 Hz, 1H), 7.19 (dd, *J* = 8.1, 7.5 Hz, 1H), 7.13 (d, *J* = 7.5 Hz, 1H), 4.63 (t, *J* = 9.3 Hz, 1H), 3.85 (s, 1H), 3.72 (s, 1H), 3.62 (t, *J* = 11.0 Hz, 1H), 3.40 – 3.32 (m, 1H), 2.76 – 2.68 (m, 1H), 1.48 (t, *J* = 13.2 Hz, 1H), 1.35 (s, 9H)
^{13}C NMR (150 MHz, $CDCl_3$) δ (ppm) = 176.34, 143.57 129.95, 129.62, 128.19, 122.98, 118.47, 57.13, 55.09, 50.38, 39.90, 32.77, 27.59, 26.17
LRMS (ES$^+$) = *m/z* $[M + H]^+$ calcd. for $C_{16}H_{20}NO_2^+$, 258.15; found, 258.21
IR (Smart iTX Diamond) = ν 2972, 2874, 2245, 1639, 1594 cm^{-1}

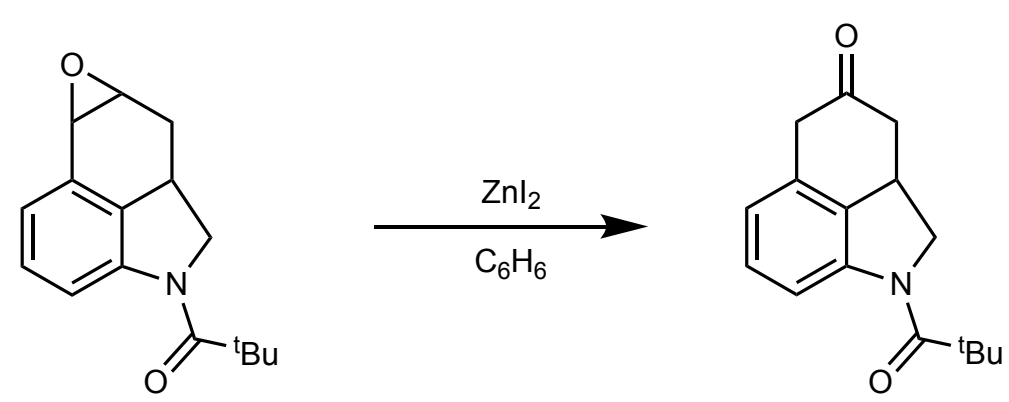

6 7

1-pivaloyl-1,2,2a,5-tetrahydrobenzo[cd]indol-4(3H)-one (**7**)

To a stirred solution of compound **6** (1.64 g, 6.37 mmol, 1.00 equiv.) in benzene (44.0 mL) was added ZnI_2 (0.612 g, 1.92 mmol, 0.300 equiv.). The solution was stirred at reflux for 2 h. The solution was cooled to ambient temperature then diluted with water (20 mL). The biphasic layers were separated. The aqueous layer was further extracted with EtOAc (3 x 20 mL). The combined organic extracts were washed with brine (40 mL), dried over Na_2SO_4, filtered, and then concentrated under reduced pressure. The residue was purified by chromatography on silica gel (75:25 hexanes/EtOAc) to afford compound **7** (1.56 g, 95%) as a yellow solid.

R_f = 0.25 (75:25 hexanes/EtOAc)
^{1}H NMR (600 MHz, $CDCl_3$) δ (ppm) = 7.98 (d, *J* = 8.0 Hz, 1H), 7.21 (t, *J* = 7.9 Hz, 1H), 6.86 (d, *J* = 7.6 Hz, 1H), 4.74 (t, *J* = 8.5 Hz, 1H), 3.82 – 3.68 (m, 2H), 3.54 (s, 2H), 2.97 (dd, *J* = 16.2, 4.7 Hz, 1H), 2.32 (dd, *J* = 15.6, 11.6 Hz, 1H), 1.38 (s, 9H)
^{13}C NMR (101 MHz, CD_3OD) δ (ppm) = 209.49, 177.24, 142.79, 130.83, 130.48, 128.25, 122.50, 116.15, 57.44, 42.93, 41.30, 39.65, 35.75, 26.57
LRMS (ES$^+$) = *m/z* $[M + H]^+$ calcd. for $C_{16}H_{20}NO_2^+$, 258.15; found, 258.21
IR (Smart iTX Diamond) = ν 2970, 2874, 2245, 1710, 1640, 1623, 1593 cm^{-1}

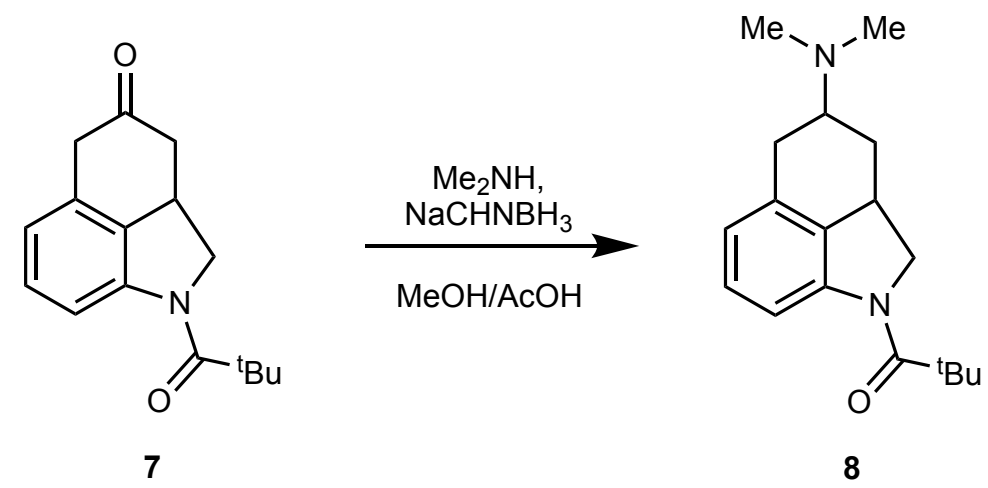

1-(4-(dimethylamino)-2a,3,4,5-tetrahydrobenzo[cd]indol-1(2*H*)-yl)-2,2-dimethylpropan-1-one (**8**) (mixture of diastereomers)

To a stirred solution of compound **7** (1.31 g, 5.10 mmol, 1.00 equiv.) and AcOH (0.870 mL, 15.2 mmol, 3.00 equiv.) in MeOH (34.0 mL) at 0 °C was added 40% wt. dimethylamine in H_2O (3.45 g, 30.6 mmol, 6.00 equiv.) and $NaCNBH_3$ (0.576 g, 9.17 mmol, 1.80 equiv.). The solution was warmed to ambient temperature and stirred for 24 h before being quenched with 1 M NaOH (30 mL). The mixture was extracted with DCM (3 x 20 mL). The organic extracts were combined, dried over Na_2SO_4, filtered, and then concentrated under reduced pressure. The residue was purified by chromatography on silica gel (90:10:1 DCM/MeOH/$NH_4OH_{(aq)}$) to afford an inseparable mixture of diastereomers **8** (780 mg, 53%, 3:1 dr) as a red solid. The diastereomeric ratio was determined by ^{1}H NMR analysis.

R_f (mixture of diastereomers) = 0.50 (90:10:1 DCM/MeOH/$NH_4OH_{(aq)}$)
^{1}H NMR (600 MHz, $CDCl_3$, mixture of diastereomers) δ (ppm) = 7.91 – 7.80 (m, 1.16H), 7.15 – 7.07 (m, 1.33H), 6.86 – 6.79 (m, 1.29H), 4.61* (t, *J* = 8.64 Hz, 0.38H), 4.56† (t, *J* = 8.64 Hz, 1.00H), 3.57 (t, *J* = 10.8 Hz, 1.38H), 3.51 – 3.41* (m, 0.38H), 3.36 – 3.25 (m, 1.03H), 3.01 – 2.91 (m, 2.07H), 2.84 – 2.64 (m, 2.23H), 2.38† (s, 6.09H), 2.34* (s, 2.25H), 2.33 – 3.28 (m, 1.44H), 1.56 – 1.49* (m, 0.41H), 1.42 – 1.32 (m, 13.9H)
^{13}C NMR (150 MHz, $CDCl_3$, mixture of diastereomers) δ (ppm) = 176.28, 176.24, 143.17, 143.11, 132.91, 132.54, 131.56, 131.14, 128.15, 127.86, 123.25, 122.60, 115.76, 115.46, 61.04, 60.56, 58.36, 57.61, 42.78, 41.20, 39.82, 39.80, 38.47, 33.79, 29.41, 28.91, 28.16, 27.63
LRMS (ES$^+$, mixture of diastereomers) = *m/z* [M + H]$^+$ calcd. for $C_{18}H_{27}N_2O^+$, 287.21; found, 287.45
IR (**Smart iTX Diamond, mixture of diastereomers) =** ν 2931, 2869, 2821, 2771, 1643, 1592 cm^{-1}

†denotes ^{1}H NMR signal arising exclusively from the major diastereomer; *denotes ^{1}H NMR signal arising exclusively from the minor diastereomer; undesignated signals arise from a mixture of both.

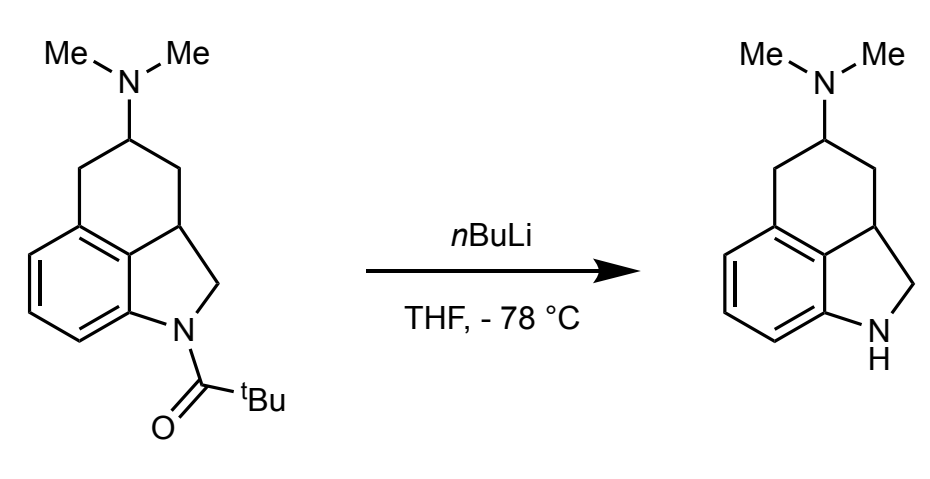

***N*,*N*-dimethyl-1,2,2a,3,4,5-hexahydrobenzo[cd]indol-4-amine** (**S1**) (mixture of diastereomers)

To a stirred solution of **8** (3:1 mixture of diastereomers) (270 mg, 0.943 mmol, 1.00 equiv.) in THF (6.25 mL) at -78 °C was added 2.5 M n-BuLi in hexanes (1.13 mL, 2.82 mmol, 3.00 equiv.) dropwise. The mixture was stirred at -78 °C for 40 min before being quenched with saturated aqueous NH_4Cl (1.0 mL), followed by 1 M NaOH (5.0 mL). The solution was warmed to ambient temperature and was then extracted with EtOAc (3 x 2.0 mL). The combined organic extracts

were dried over Na_2SO_4, filtered, and then concentrated under reduced pressure. The residue was purified by chromatography on silica gel (gradient elution: 100:0:0 DCM/MeOH/$NH_4OH_{(aq)}$ → 90:10:1 DCM/MeOH/$NH_4OH_{(aq)}$) to afford an inseparable mixture of diastereomers **S1** (147 mg, 77%, 3:1 dr) as a dark green solid. The diastereomeric ratios were determined by ^1H NMR analysis.

Note: On occasion a small amount of white precipitate crashed out when starting material **8** was dissolved in THF. In these instances, the product was filtered, concentrated and redissolved in THF.

R_f (mixture of diastereomers) = 0.28 (90:10:1 DCM/MeOH/$NH_4OH_{(aq)}$)
^1H NMR (600 MHz, $CDCl_3$, mixture of diastereomers) δ (ppm) = 7.02 – 6.92 (m, 1.24H), 6.55 – 6.52 (m, 1.21H), 6.52 – 6.45 (m, 1.17H), 3.85 – 3.52 (m, 2.83H), 3.35 – 3.26* (m, 0.44H), 3.22 – 3.07 (m, 2.76H), 3.04 – 2.91 (m, 2.19H), 2.82 – 2.70 (m, 1.33H), 2.69 – 2.62 (m, 1.08H), 2.39† (s, 6H), 2.36* (s, 2.54H), 2.31 – 2.23 (m, 1.41H), 1.57 – 1.51* (m, 0.43H), 1.42 – 1.34† (m, 1.16H)
^{13}C NMR (150 MHz, $CDCl_3$, mixture of diastereomers) δ (ppm) = 150.27, 133.50, 133.09, 130.60, 130.42, 127.82, 127.49, 118.09, 117.65, 106.71, 106.31, 61.72, 61.24, 57.13, 56.21, 42.82, 41.25, 38.98, 34.26, 29.66, 29.41, 29.29, 28.28
LRMS (ES^+, mixture of diastereomers) = *m/z* $[M + H]^+$ calcd. for $C_{13}H_{19}N_2^+$, 203.15; found, 203.34
IR (Smart iTX Diamond) = ν 3274, 2927, 2858, 2823, 2773, 1621, 1602 cm^{-1}

†denotes ^1H NMR signal arising exclusively from the major diastereomer; *denotes ^1H NMR signal arising exclusively from the minor diastereomer; undesignated signals arise from a mixture of both.

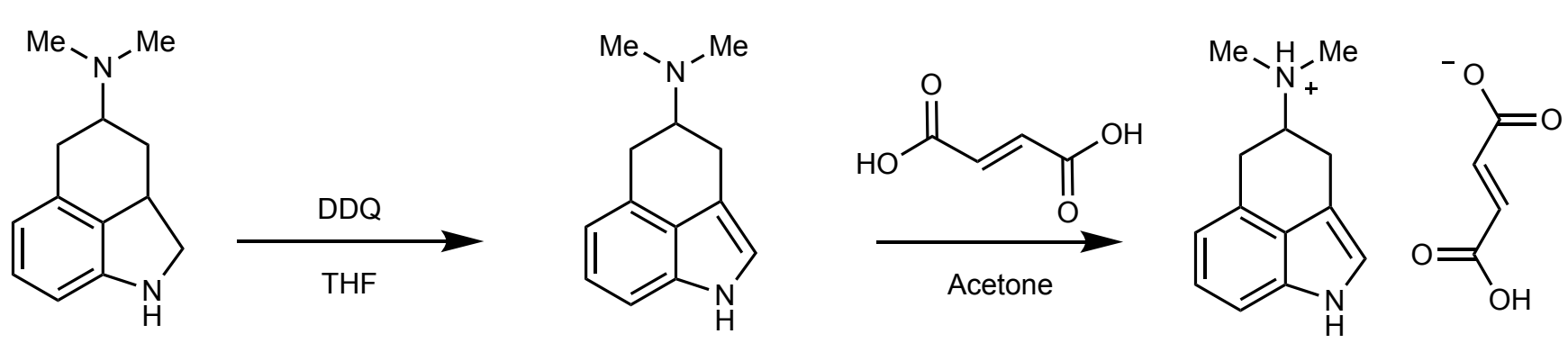

***N,N*-dimethyl-1,3,4,5-tetrahydrobenzo[cd]indol-4-aminium fumarate** (**UCD0094 fumarate**)
To a stirred solution of **S1** (3:1 mixture of diastereomers) (104 mg, 0.514 mmol, 1.00 equiv.) in THF (13.0 mL) was added DDQ (128 mg, 0.564 mmol, 1.10 equiv.). The mixture was stirred for 2 h before being quenched with 1 M NaOH (5.00 mL). The solution was then extracted with ether (3 x 10 mL) and the organic extracts were combined, dried over Na_2SO_4, filtered, and then concentrated under reduced pressure. The residue was purified by chromatography on silica gel (gradient elution: 95:10:0.5 DCM/MeOH/$NH_4OH_{(aq)}$ → 90:10:1 DCM/MeOH/$NH_4OH_{(aq)}$) to afford ***N,N*-dimethyl-1,3,4,5-tetrahydrobenzo[cd]indol-4-amine** (**UCD0094 freebase**) (81.0 mg, 79%) as a brown oil.

UCD0094 freebase
R_f = 0.35 (90:10:1 DCM/MeOH/$NH_4OH_{(aq)}$)
^1H NMR (600 MHz, $CDCl_3$) δ (ppm) = 8.09 (br, 1H), 7.17 – 7.09 (m, 2H), 6.85 (d, *J* = 7.5 Hz, 2H), 3.15 – 3.09 (m, 2H), 3.08 – 2.98 (m, 2H), 2.89 – 2.80 (m, 1H), 2.48 (s, 6H)

The fumarate salt was prepared according to **General Procedure A** using fumaric acid (42.0 mg, 0.362 mmol, 0.900 equiv.) and **UCD0094 freebase** (81.0 mg, 0.404 mmol, 1.00 equiv.) with

acetone as the solvent. The product **UCD0094 fumarate** (30 mg, 23%) was obtained as a pink salt.

UCD0094 fumarate
^{1}H NMR (600 MHz, CD_3OD) δ (ppm) = 7.20 (d, *J* = 8.2 Hz, 1H), 7.08 (t, *J* = 7.1 1H), 7.03 (s, 1H), 6.84 (d, *J* = 7.0 Hz, 1H), 6.70 (s, 2H), 3.87 – 3.77 (m, 1H), 3.37 (dd, *J* = 14.2, 4.6 Hz, 2H), 3.28 (d, *J* = 9.0 Hz, 1H), 3.20 (dd, *J* = 15.0, 9.0 Hz, 1H), 2.93 (s, 6H)
^{13}C NMR (150 MHz, CD_3OD) δ (ppm) = 169.73, 134.72, 133.91, 125.88, 125.21, 122.54, 119.21, 115.66, 109.21, 106.80, 63.48, 39.79, 28.18, 23.04
LRMS (ES$^+$) = *m/z* $[M]^+$ calcd. for $C_{13}H_{19}N_2^+$, 201.14; found, 201.30
HRMS (ES$^+$) = *m/z* $[M]^+$ calcd. for $C_{13}H_{19}N_2^+$, 201.1386; found, 201.1387
IR (Smart iTX Diamond) = ν 3209, 3039, 2941, 2462, 1905, 1699, 1567 cm^{-1}
Ratio of Amine to Acid = 1:1 (Ratio Determined by ^{1}H NMR)
Purity = >99% (Diode Array, 210 nm)

Experimental Procedures for the Synthesis of UCD0179 and UCD0120

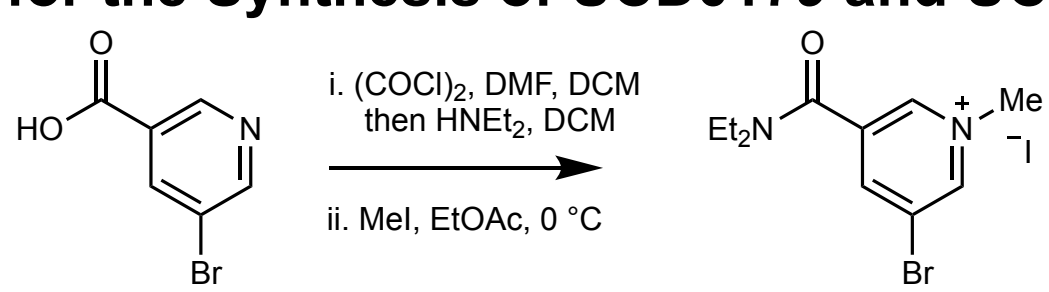

9 **S2**

3-bromo-5-(diethylcarbamoyl)-1-methylpyridin-1-ium Iodide (S2)
To a stirred solution of **5-bromonicotinic acid** (**9**) (2.02 g, 10.0 mmol, 1.00 equiv.) and DMF (0.100 mL) in DCM (20.0 mL) at 0 °C was added $(COCl)_2$ (2.14 mL, 25.0 mmol, 2.50 equiv.) dropwise. After $(COCl)_2$ addition, the solution was warmed to ambient temperature and stirred for 2 h before being concentrated under reduced pressure. The residue was dissolved in DCM (20.0 mL) and cooled to 0 °C. A solution of diethylamine (5.16 mL, 50.0 mmol, 5.00 equiv.) in DCM (20.0 mL) was then added to the stirred solution at 0 °C via addition funnel over 30 min. Saturated aqueous $NaHCO_3$ (50 mL) was then added to mixture. The biphasic layers were separated. The aqueous layer was further extracted with DCM (3 x 20 mL). The organic extracts were combined, washed with saturated brine (50 mL), dried over Na_2SO_4, filtered, and concentrated under reduced pressure to afford an oil. The oil was dissolved in anhydrous EtOAc (12.0 mL), then MeI (5.00 mL, 80.3 mmol, 8.03 equiv.) was added to the solution. The vessel was sealed and heated to 70 °C for 22 h before being cooled to ambient temperature. The mixture was further diluted with EtOAc (12 mL), then filtered. The filter cake was washed with EtOAc (3 x 5 mL) giving compound **S2** (3.74 g, 94%) as yellow solid. The solid was used without further purification.

^{1}H NMR (400 MHz, CD_3OD) δ (ppm) = 9.41 (s, 1H), 9.18 (s, 1H), 8.92 (s, 1H), 4.49 (s, 3H), 3.61 (q, *J* = 7.2 Hz, 2H), 3.41 (q, *J* = 7.2 Hz, 2H), 1.30 (t, *J* = 7.2 Hz, 3H), 1.21 (t, *J* = 7.2 Hz, 3H)
^{13}C NMR (101 MHz, CD_3OD) δ (ppm) = 163.28, 147.68, 145.12, 142.33, 137.49, 122.62, 122.48, 43.96, 40.01, 13.00, 11.56
LRMS (ES$^+$) = *m/z* $[M]^+$ calcd. for $C_{11}H_{16}BrN_2O^+$, 271.04; found, 271.11/273.11
IR (Smart iTX Diamond) = ν 2973, 2260, 1625, 1440, 1382, 1284, 1209, 1166, 1016, 796 cm^{-1}

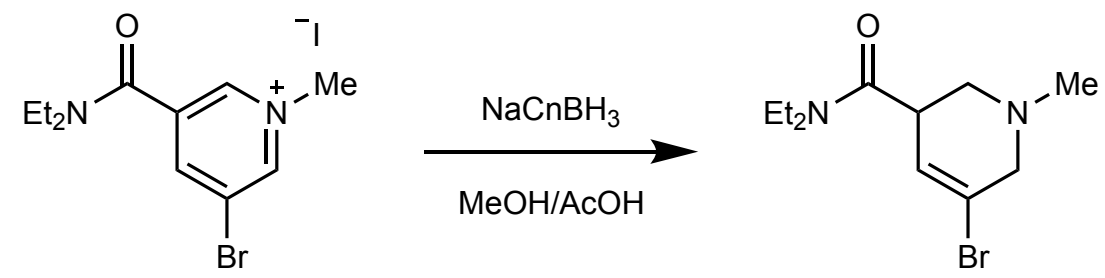

S2 10

5-bromo-*N*,*N*-diethyl-1-methyl-1,2,3,6-tetrahydropyridine-3-carboxamide (**10**)

To a stirred solution of compound **S2** (2.00 g, 5.01 mmol, 1.00 equiv.) and acetic acid (1.72 mL, 30.0 mmol, 6.00 equiv.) in MeOH (58.3 mL) at 0 °C was added $NaCNBH_3$ (0.944 g, 15.0 mmol, 3.00 equiv.). The solution was then stirred for 21 h at ambient temperature before being concentrated under reduced pressure. The material was diluted with 1 M NaOH (15 mL), and the aqueous mixture was extracted with DCM (3 x 10 mL). The combined organic extracts were washed with brine (20 mL), dried over Na_2SO_4, filtered, and then concentrated under reduced pressure. The residue was purified by chromatography on silica gel (95:5:0.5, DCM/MeOH/$NH_4OH_{(aq)}$) to afford compound **10** (1.30 g, 94%) as a yellow oil.

R_f = 0.50 (90:10:1 DCM/MeOH/$NH_4OH_{(aq)}$)
^{1}H NMR (400 MHz, $CDCl_3$) δ (ppm) = 5.98 (s, 1H), 3.68 – 3.58 (m, 1H), 3.41 – 3.27 (m, 5H), 3.07 (ddd, *J* = 16.3, 3.5, 2.4 Hz, 1H), 2.87 (dd, *J* = 11.6, 5.4 Hz, 1H), 2.63 (dd, *J* = 11.6, 9.7 Hz, 1H), 2.39 (s, 3H), 1.20 (t, *J* = 7.1 Hz, 3H), 1.11 (t, *J* = 7.1 Hz, 3H)
^{13}C NMR (101 MHz, $CDCl_3$) δ (ppm) = 170.24, 125.54, 120.66, 60.74, 53.49, 44.76, 42.03, 41.44, 40.38, 14.88, 13.05
LRMS (ES^+) = *m/z* $[M + H]^+$ calcd. for $C_{11}H_{20}BrN_2O^+$, 275.08; found, 275.15/277.16
IR (Smart iTX Diamond) = ν 2969, 2933, 2780, 1633, 1429, 1376, 1265, 1232, 1216, 1130, 991 cm^{-1}

General Procedure C: Suzuki Coupling

To a stirred solution of compound **10** (275 mg, 1.00 mmol, 1.00 equiv.), boronic acid/ester (1.00 mmol, 1.00 equiv.), and potassium carbonate (276, mg, 2.00 mmol 2.00 equiv.) in toluene (10.0 mL), EtOH (1.0 mL) and H_2O (1.00 mL) was added $Pd(PPh_3)_4$ (57.0 mg, 0.05 mmol, 0.05 equiv.). The solution was heated to 80 °C for 48 h, unless noted otherwise. The solution was then cooled to ambient temperature and washed with brine (10 mL). The organic layer was concentrated under reduced pressure then purified by chromatography on silica gel (90:10:1, DCM/MeOH/$NH_4OH_{(aq)}$) unless noted otherwise.

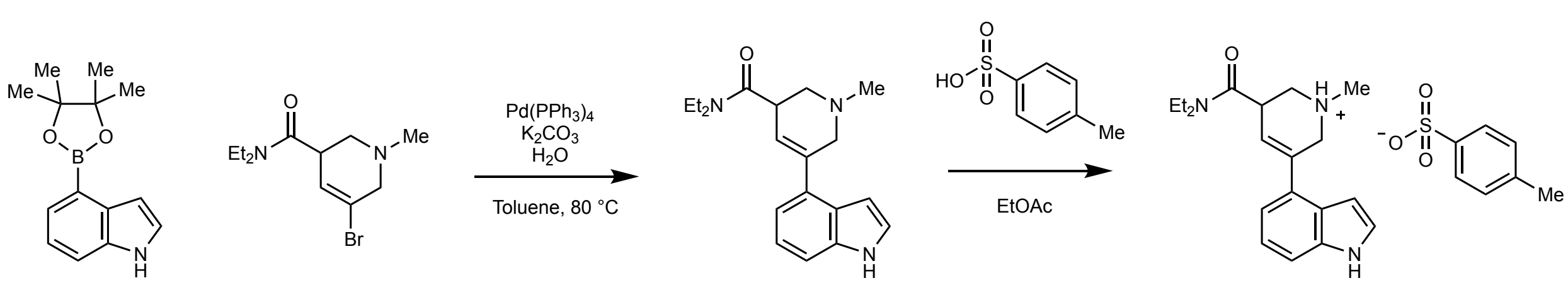

19 10 UCD0179 (freebase) UCD0179 (tosylate)

3-(diethylcarbamoyl)-5-(1*H*-indol-4-yl)-1-methyl-1,2,3,6-tetrahydropyridin-1-ium 4-methylbenzenesulfonate (**UCD0179 tosylate**)

UCD0179 was prepared from compound **10**, **indole-4-boronic acid pinacol ester (19(** (243 mg, 1.00 mmol, 1.00 equiv.), potassium carbonate, and $Pd(PPh_3)_4$ in toluene, EtOH and H_2O following **General Procedure C**. The residue was purified by chromatography on silica gel (90:10:1, DCM/MeOH/$NH_4OH_{(aq)}$) to afford **UCD0179 freebase** (168 mg, 54%) as a light red/brown oil.

UCD0179 freebase

R_f = 0.25 (90:10:1 DCM/MeOH/$NH_4OH_{(aq)}$)

^{1}H NMR (400 MHz, CD_3OD) δ (ppm) = 7.34 (d, *J* = 8.2 Hz, 1H), 7.25 (d, *J* = 3.2 Hz, 1H), 7.09 (t, *J* = 7.54 Hz, 1H), 6.95 (dd, *J* = 7.4, 1.0 Hz, 1H), 6.65 (dd, *J* = 3.2, 1.0 Hz, 1H), 5.97 (s, 1H), 4.03 – 3.93 (m, 1H), 3.72 (d, *J* = 16.1 Hz, 1H), 3.56 (q, *J* = 7.2 Hz, 2H), 3.44 (q, *J* = 7.2 Hz, 2H), 3.24 (dt, *J* = 16.1, 3.4 Hz, 1H), 3.08 (dd, *J* = 11.3, 5.7 Hz, 1H), 2.70 (t, *J* = 10.7 Hz, 1H), 2.53 (s, 3H), 1.30 (t, *J* = 7.2 Hz, 3H), 1.17 (t, *J* = 7.1 Hz, 3H)

The tosylate salt was prepared according to **General Procedure A** using *p*-toluenesulfonic acid hydrate (36.1 mg, 0.190 mmol, 1.00 equiv.) and **UCD0179 freebase** (59.1 mg, 0.190 mmol, 1.00 equiv.) with anhydrous EtOAc as the solvent. The product **UCD0179 tosylate** (45.6 mg, 48%) was obtained as a light orange solid.

UCD0179 tosylate
^{1}H NMR (300 MHz, CD_3OD with saturated K_2CO_3) δ (ppm) = 7.73 (d, *J* = 8.3 Hz, 2H), 7.34 (d, *J* = 8.1 Hz, 1H), 7.31 – 7.19 (m, 3H), 7.09 (t, *J* = 7.5 Hz, 1H), 6.95 (d, *J* = 8.3 Hz, 1H), 6.66 (d, *J* = 4.2 Hz, 1H), 5.97 (s, 1H), 3.97 (s, 1H), 3.71 (d, *J* = 16.4 Hz, 1H), 3.56 (q, *J* = 7.2 Hz, 2H), 3.44 (q, *J* = 7.1 Hz, 2H), 3.22 (d, *J* = 18.6 Hz, 1H), 3.12 – 3.00 (m, 1H), 2.77 – 2.61 (m, 1H), 2.52 (d, *J* = 4.9 Hz, 3H), 2.39 (s, 3H), 1.30 (t, *J* = 7.1 Hz, 3H), 1.17 (t, *J* = 7.1 Hz, 3H)
^{13}C NMR (101 MHz, CD_3OD) δ (ppm) = 172.63, 142.12, 140.27, 137.82, 136.72, 131.73, 128.41, 125.76, 125.57, 124.32, 121.61, 120.72, 116.56, 110.49, 100.56, 56.59, 53.83, 44.18, 42.28, 40.46, 39.64, 19.91, 13.74, 11.89
LRMS (ES$^+$) = *m/z* $[M]^+$ calcd. for $C_{19}H_{26}N_3O^+$, 312.21; found, 312.26
HRMS (ES$^+$) = *m/z* $[M]^+$ calcd. for $C_{19}H_{26}N_3O^+$, 312.2070; found, 312.2082
IR (Smart iTX Diamond) = ν 3253, 2975, 2931, 1625, 1461, 1382, 1355, 1213, 1182, 1122, 1033 cm^{-1}
Ratio of Amine to Acid = 1:1 (Ratio Determined by ^{1}H NMR)
Purity = >99% (Diode Array, 210 nm)

Note: Additional ^{1}H NMR signals are observed in CD_3OD for **UCD0179-Salt** (presumably due to atropisomerism), but may be resolved upon addition of anhydrous K_2CO_3.

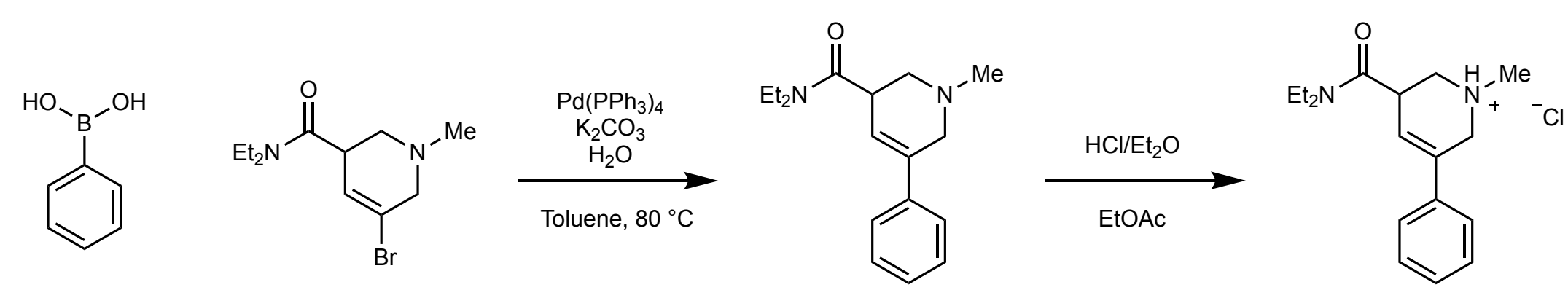

Phenylboronic acid (S3) 10 UCD0120 (freebase) UCD0120 (hydrocloride)

3-(diethylcarbamoyl)-1-methyl-5-phenyl-1,2,3,6-tetrahydropyridin-1-ium hydrochloride (**UCD0120 hydrochloride**)
UCD0120 was prepared from compound **10** and **phenylboronic acid** (**S3**) (122 mg, 1.00 mmol, 1.00 equiv.), potassium carbonate, and $Pd(PPh_3)_4$ in toluene, EtOH and H_2O following **General Procedure C**. The residue was purified by chromatography on silica gel (90:10:1, DCM/MeOH/$NH_4OH_{(aq)}$) to afford ***N,N*-diethyl-1-methyl-5-phenyl-1,2,3,6-tetrahydropyridine-3-carboxamide** (**UCD0120 freebase**) (179 mg, 66%) as a light orange oil.

UCD0120 freebase
R_f = 0.24 (95:5:0.5 DCM/MeOH/$NH_4OH_{(aq)}$)
^{1}H NMR (300 MHz, $CDCl_3$) δ (ppm) = 7.73 – 7.60 (m, 2H), 7.59 – 7.50 (m, 1H), 7.50 – 7.41 (m, 2H), 5.98 (s, 1H), 3.69 – 3.60 (m, 1H), 3.41 – 3.30 (m, 5H), 3.07 (ddd, *J* = 16.3, 3.9, 2.3 Hz, 1H),

2.88 (dd, *J* = 11.5, 5.3 Hz, 1H), 2.63 (dd, *J* = 11.5, 9.7 Hz, 1H), 2.39 (s, 3H), 1.20 (t, *J* = 7.1 Hz, 3H), 1.11 (t, *J* = 7.1 Hz, 3H)

The hydrochloride salt was prepared according to **General Procedure A** using ethereal HCl (0.460 mL, 1.00 M, 1.00 equiv.) and **UCD0120 freebase** (122 mg, 0.449 mmol, 1.00 equiv.) with anhydrous EtOAc as the solvent. The product **UCD0120 hydrochloride** (68.2 mg, 49%) was obtained as a white powder.

UCD0120 hydrochloride
^{1}H NMR (300 MHz, CD_3OD) δ (ppm) = 7.51 – 7.30 (m, 5H), 6.32 (s, 1H), 4.34 (d, *J* = 16.2 Hz, 1H), 4.10 (d, *J* = 16.7 Hz, 2H), 3.85 – 3.65 (m, 2H), 3.55 (dt, *J* = 14.9, 7.2 Hz, 2H), 3.45 – 3.34 (d, *J* = 13.6 Hz, 2H), 3.13 (s, 3H), 1.33 (t, *J* = 7.2 Hz, 3H), 1.17 (t, *J* = 7.1 Hz, 3H)
^{13}C NMR (101 MHz, CD_3OD) δ (ppm) = 171.41, 136.55, 134.35, 128.62, 125.21, 118.22, 52.99, 52.75, 42.48, 42.22, 40.33, 35.98, 13.59, 11.72
LRMS (ES$^+$) = *m/z* $[M]^+$ calcd. for $C_{17}H_{25}N_2O^+$, 273.20; found, 273.25
HRMS (ES$^+$) = *m/z* $[M]^+$ calcd. for $C_{17}H_{25}N_2O^+$, 273.1961; found, 273.1962
IR (Smart iTX Diamond) = ν 3450, 2969, 2470, 2260, 1625, 1440, 1382, 1284, 1209, 1166, 1016, 796 cm^{-1}
Ratio of Amine to Acid = 1:1 (Ratio Determined by ^{1}H NMR utilizing anhydrous DMSO as the deuterated solvent. The exchangeable NH peak was confirmed by adding D_2O)
Purity = >99% (Diode Array, 210 nm)

Experimental Procedures for the Synthesis of UCD0121

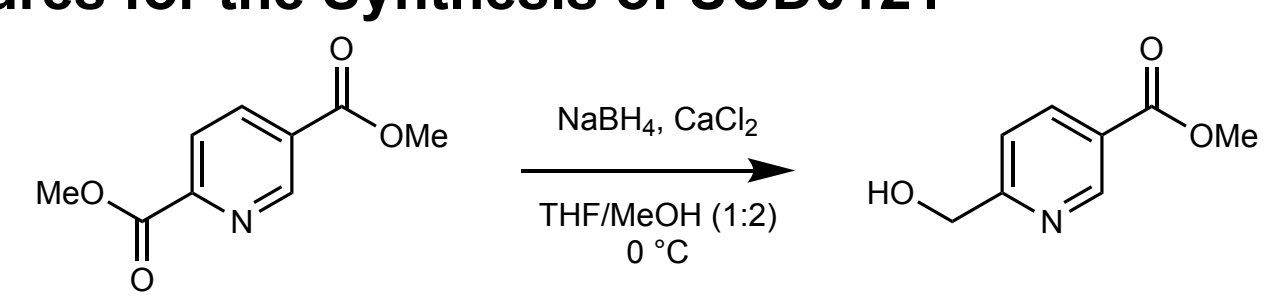

11 12

methyl 6-(hydroxymethyl)nicotinate (**12**)
Compound **12** was synthesized using a modified literature procedure.[14] To a stirred solution of **dimethyl pyridine-2,5-dicarboxylate** (**11**) (10.0 g, 51.2 mmol, 1.00 equiv.) and $CaCl_2$ (22.4 g, 202 mmol, 4.00 equiv.) in THF/MeOH (1:2, 318 mL) at 0 °C was added $NaBH_4$ (4.82 g, 127 mmol, 2.50 equiv.) in three equivalent portions. The mixture was stirred at 0 °C for 1.5 h before being quenched with a dropwise addition of H_2O (256 mL) at 0 °C. The resulting mixture was concentrated under reduced pressure, then extracted with $CHCl_3$ (3 x 80 mL). The organic extracts were combined, washed with brine (200 mL), dried over Na_2SO_4, filtered, and then concentrated under reduced pressure to afford compound **12** (6.06 g, 71%) as a white solid, which was used in the next step without further purification.

^{1}H NMR (400 MHz, $CDCl_3$) δ (ppm) = 9.13 (d, *J* = 2.1 Hz, 1H), 8.27 (dd, *J* = 8.2, 2.1 Hz, 1H), 7.37 (d, *J* = 8.2 Hz, 1H), 4.81 (s, 2H), 3.94 (s, 3H)
^{13}C NMR (101 MHz, $CDCl_3$) δ (ppm) = 165.62, 163.74, 149.94, 137.78, 124.89, 120.04, 64.32, 52.41
LRMS (ES$^+$) = *m/z* $[M + H]^+$ calcd. for $C_8H_{10}NO_3^+$, 168.07; found, 168.14
IR (Smart iTX Diamond) = ν 3336, 3097, 2966, 2879, 1724, 1596, 1430, 1384, 1280, 1114, 1024 cm^{-1}

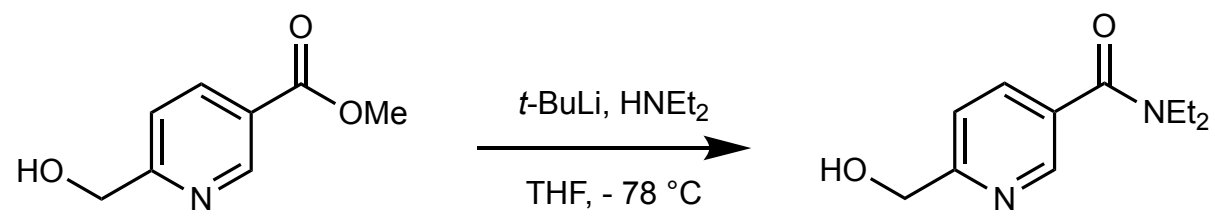

12 S4

***N*,*N*-diethyl-6-(hydroxymethyl)nicotinamide** (**S4**)
To a stirred solution of diethylamine (6.16 mL, 59.5 mmol, 2.60 equiv.) in THF (5.50 mL) at -78 °C was added t-BuLi (1.7 M) in hexanes (35.0 mL, 59.5 mmol, 2.60 equiv.) dropwise. The mixture was stirred at -78 °C for 30 min before being cannulated into a stirred solution of compound **12** (3.83 g, 22.9 mmol, 1.00 equiv.) in THF (76.4 mL) at -78 °C. The resulting solution was then stirred at –78 °C for 1 h before being slowly quenched with a 1:5 MeOH/THF mixture (6.00 mL). A saturated solution of NH_4Cl (50 mL) was added to the reaction before warming it to ambient temperature. The mixture was extracted with EtOAc (3 x 40 mL). The organic extracts were combined, washed with saturated brine (50.0 mL), dried over Na_2SO_4, filtered, and then concentrated under reduced pressure. The residue was purified by chromatography on silica gel (100% EtOAc, then 90:10:1 DCM/MeOH/$NH_4OH_{(aq)}$) to afford compound **S4** (3.75 g, 79%) as a yellow oil.

R_f = 0.46 (90:10:1 DCM/MeOH/$NH_4OH_{(aq)}$)
^{1}H NMR (400 MHz, $CDCl_3$) δ (ppm) = 8.43 (d, *J* = 2.2 Hz, 1H), 7.58 (dd, *J* = 8.0, 2.2 Hz, 1H), 7.30 (d, *J* = 8.0 Hz, 1H), 4.70 (br, 1H), 4.65 (s, 2H), 3.45 (br, 2H), 3.17 (br, 2H), 1.16 (br, 3H), 1.04 (br, 3H)
^{13}C NMR (101 MHz, $CDCl_3$) δ (ppm) = 168.72, 161.49, 145.97, 134.95, 131.17, 120.05, 64.33, 43.48, 39.64, 14.23, 12.78
LRMS (ES$^+$) = *m/z* $[M + H]^+$ calcd. for $C_{11}H_{17}N_2O_2^+$, 209.13; found, 209.20
IR (Smart iTX Diamond) = ν 3346, 2973, 2935, 1596, 1432, 1375, 1288, 1101, 1068, 1025, 754 cm^{-1}

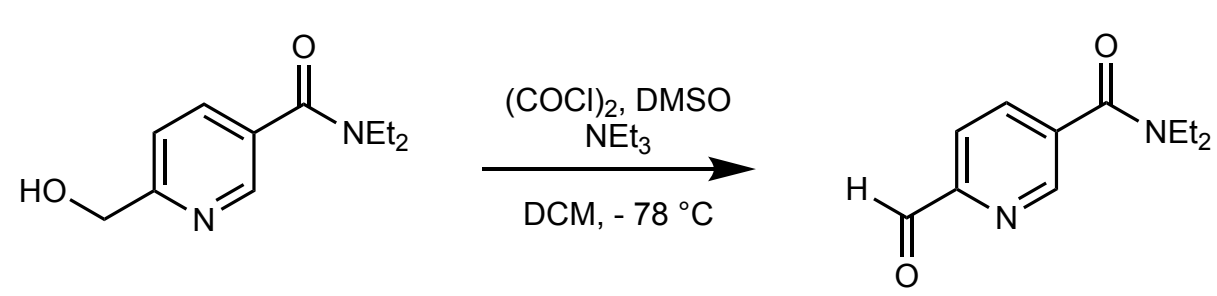

S4 13

***N*,*N*-diethyl-6-formylnicotinamide** (**13**)
To a stirred solution of $(COCl)_2$ (2.93 mL, 34.1 mmol, 1.50 equiv.) in DCM (41.7 mL) -78 °C was added DMSO (4.97 mL, 70.0 mmol, 3.0 equiv.) dropwise. The solution was then stirred for 30 min at -78 °C before compound **S4** (4.86 g, 23.3 mmol, 1.00 equiv.) in DCM (58.4 mL) was added to the solution. The mixture was stirred for an additional 1 h at -78 °C before NEt_3 (11.7 mL, 83.9 mmol, 3.60 equiv.) was added. The mixture was stirred for an additional 45 min at -78 °C before being quenched with saturated aqueous NH_4Cl (50 mL). The aqueous solution was then extracted with DCM (3 x 50 mL). The combined organic extracts were washed with saturated aqueous $NaHCO_3$ (50 mL), brine (50 mL), dried over Na_2SO_4, filtered, and then concentrated under reduced pressure. The residue was purified by chromatography on silica gel (gradient elution: 50:50 EtOAc/hexanes → 100:00 EtOAc/hexanes) to afford compound **13** (3.42 g, 71%) as a lime-green oil.

Note: Gas pressure build up was observed upon addition of DMSO.

R_f = 0.2 (50:50 EtOAc/hexanes)

^{1}H NMR (400 MHz, CDCl$_3$) δ (ppm) = 10.03 (s, 1H), 8.73 (d, *J* = 2.2 Hz, 1H), 7.95 (d, *J* = 7.9 Hz, 1H), 7.83 (dd, *J* = 7.9, 2.2 Hz, 1H), 3.52 (q, *J* = 7.2 Hz, 2H), 3.19 (q, *J* = 7.2 Hz, 2H), 1.25 – 1.17 (m, 3H), 1.13 – 1.03 (m, 3H) ppm
^{13}C NMR (101 MHz, CDCl$_3$) δ (ppm) = 192.53, 167.44, 152.69, 147.64, 136.76, 135.21, 121.42, 43.38, 39.65, 14.27, 12.82 ppm
LRMS (ES$^+$) = *m/z* [M + H]$^+$ calcd. for $C_{11}H_{15}N_2O_2^+$, 207.11; found, 207.25
IR (Smart iTX Diamond) = ν 2973, 2935, 1712, 1625, 1429, 1363, 1286, 1218, 1097, 829, 730 cm^{-1}

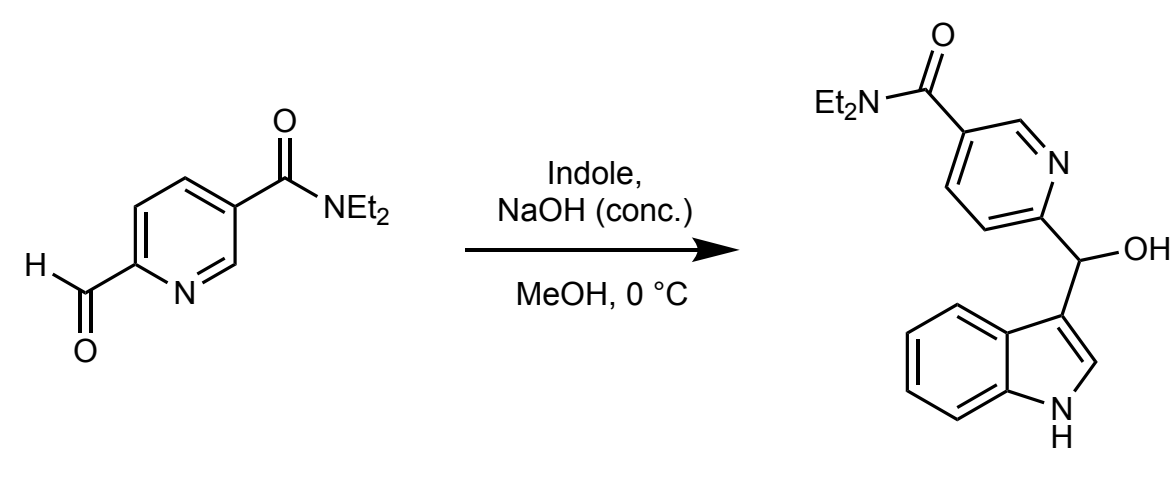

13 **S5**

***N,N*-diethyl-6-(hydroxy(1*H*-indol-3-yl)methyl)nicotinamide** (**S5**)
To a stirred solution of **indole** (2.17 g, 18.5 mmol, 1.30 equiv.) and conc. NaOH (1.42 mL) in MeOH (14.0 mL) at 0 °C was added compound **13** (2.94 g, 14.3 mmol, 1.00 equiv.) in MeOH (5.00 mL). The solution was warmed to ambient temperature and stirred for 6.5 h before being diluted with H_2O (40 mL) and extracted with DCM (3 x 40.mL). The combined organic extracts were washed with brine, dried over Na_2SO_4, filtered, and then concentrated under reduced pressure. The residue was purified by chromatography on silica gel (gradient elution: 25:75–75:25 acetone/hexanes) to afford compound **S5** (3.55 g, 77%) as a fluffy white solid.

R_f = 0.18 (50:50 acetone/hexanes)
^{1}H NMR (400 MHz, CDCl$_3$) δ (ppm) = 9.18 (s, 1H), 8.55 (d, *J* = 2.4 Hz, 1H), 7.55 (dd, *J* = 8.0, 2.4 Hz, 1H), 7.45 (d, *J* = 8.0 Hz, 1H), 7.28 (d, *J* = 8.1 Hz, 1H), 7.19 (d, *J* = 8.1 Hz, 1H), 7.08 (t, *J* = 7.5 Hz, 1H), 6.97 (t, *J* = 7.5 Hz, 1H), 6.69 (d, *J* = 2.4 Hz, 1H), 6.03 (s, 1H), 5.09 (s, 1H), 3.53 (br, 2H), 3.22 (br, 2H), 1.24 (br, 3H), 1.09 (br, 3H)
^{13}C NMR (101 MHz, CDCl$_3$) δ (ppm) = 168.80, 162.79, 145.49, 136.79, 135.11, 131.34, 125.70, 123.80, 122.00, 121.00, 119.54, 119.45, 117.11, 111.57, 69.60, 43.57, 39.74, 14.31, 12.89
LRMS (ES$^+$) = *m/z* [M + H]$^+$ calcd. for $C_{19}H_{22}N_3O_2^+$, 324.17; found, 324.33
IR (Smart iTX Diamond) = ν 3272, 2972, 2935, 1596, 1456, 1432, 1313, 1290, 1101, 906, 727 cm^{-1}

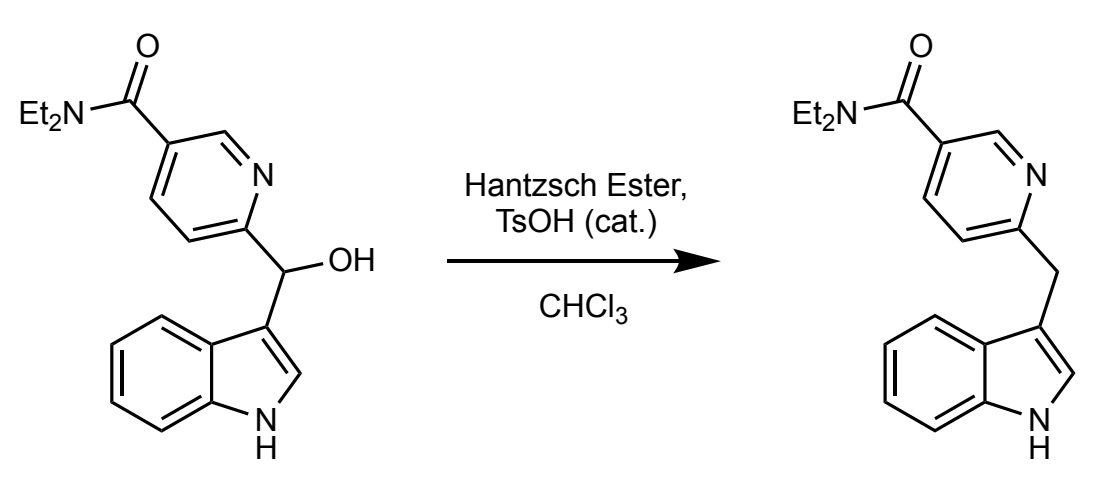

S5 **14**

6-((1*H*-indol-3-yl)methyl)-*N,N*-diethylnicotinamide (**14**)
To a stirred solution of compound **S5** (2.49 g, 7.70 mmol, 1.00 equiv.) and toluenesulfonic acid hydrate(146 mg, 0.768 mmol, 0.100 equiv.) in $CHCl_3$ (11.0 mL) was added Hantzsch's ester (1.56 g, 6.16 mmol, 0.800 equiv.). The solution was stirred at ambient temperature for 1 h before being concentrated under reduced pressure. The residue was purified by chromatography on silica gel

(25:75 acetone/hexanes → 50:50 acetone/hexanes) to afford compound **14** (2.24 g, 95%) as a yellow oil.

R_f = 0.35 (50:50 acetone/hexanes)
^{1}H NMR (300 MHz, CD_3OD) δ (ppm) = 8.50 (dd, *J* = 2.2, 0.9 Hz, 1H), 7.66 (dd, *J* = 8.1, 2.2 Hz, 1H), 7.40 (dt, *J* = 7.9, 1.0 Hz, 1H), 7.35 (dt, *J* = 8.0, 1.0, 2H), 7.13 (s, 1H), 7.09 (ddd, *J* = 8.2, 7.0, 1.1 Hz, 1H), 6.96 (ddd, *J* = 8.0, 7.0, 1.1 Hz, 1H), 4.30 (s, 2H), 3.54 (q, *J* = 6.9 Hz, 2H), 3.25 (q, *J* = 6.9 Hz, 2H), 1.24 (t, *J* = 7.6 Hz, 3H), 1.11 (t, *J* = 7.3 Hz, 3H)
8.80 (s, 1H), 8.61 (dd, *J* = 2.3, 0.9 Hz, 1H), 7.56 (dd, *J* = 8.0, 2.3 Hz, 1H), 7.48 (d, *J* = 7.9, 0.9 Hz, 1H), 7.30 (d, *J* = 8.1 Hz, 1H), 7.20 – 7.12 (m, 2H), 7.05 (td, *J* = 8.1, 1.1 Hz, 1H), 6.98 (d, *J* = 2.3 Hz, 1H), 4.32 (s, 2H), 3.55 (br, 2H), 3.27 (br, 2H), 1.24 (br, 3H), 1.12 (br, 3H)
^{13}C NMR (101 MHz, CD_3OD) δ (ppm) = 169.36, 162.93, 145.60, 136.91, 135.13, 130.43, 127.08, 123.04, 122.89, 121.16, 118.47, 118.05, 111.56, 110.98, 43.64, 39.72, 33.50, 13.00, 11.66
LRMS (ES$^+$) = *m/z* $[M + H]^+$ calcd, For $C_{19}H_{22}N_3O^+$, 308.18; found, 308.02
IR (Smart iTX Diamond) = ν 3253, 2973, 2931, 1610, 1596, 1430, 1288, 1214, 1099, 740 cm^{-1}

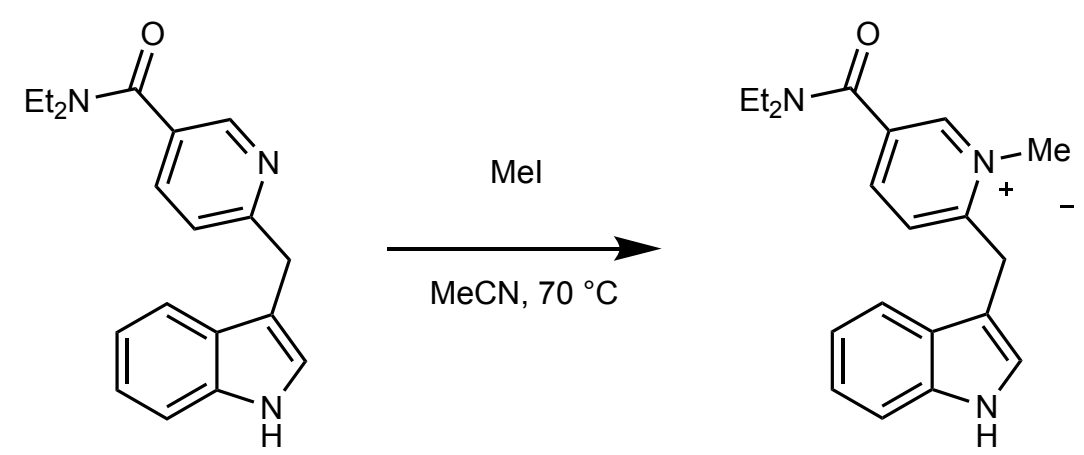

14 15

2-((1H-indol-3-yl)methyl)-5-(diethylcarbamoyl)-1-methylpyridin-1-ium (**15**)
To a stirred solution of compound **14** (2.24 g, 7.29 mmol, 1.00 equiv.) in acetonitrile (9.20 mL) was added MeI (6.70 mL, 108 mmol, 15.0 equiv.). The vial was sealed and heated to 70 °C for 26.0 h, before being cooled to ambient temperature and concentrated under reduced pressure. The residue was redissolved in acetonitrile (2.00 mL), then diluted with EtOAc (10.0 mL) before being filtered. The filter cake was isolated affording compound **15** as a yellow solid salt (2.53 g, 77%). The solid was used without further purification.

^{1}H NMR (400 MHz, DMSO-d_6) δ (ppm) = 11.25 (s, 1H), 9.21 (d, *J* = 1.9 Hz, 1H), 8.43 (dd, *J* = 8.2, 1.9 Hz, 1H), 7.63 (d, *J* = 8.2 Hz, 1H), 7.53 (d, *J* = 7.8 Hz, 1H), 7.46 (d, *J* = 8.2 Hz, 1H), 7.38 (d, *J* = 2.6 Hz, 1H), 7.16 (ddd, *J* = 8.2, 7.1, 1.2 Hz, 1H), 7.03 (ddd, *J* = 8.0, 7.1, 1.1 Hz, 1H), 4.61 (s, 2H), 4.41 (s, 3H), 3.48 (q, *J* = 7.2 Hz, 2H), 3.24 (q, *J* = 7.1 Hz, 2H), 1.18 (t, *J* = 7.1 Hz, 3H), 1.08 (t, *J* = 7.1 Hz, 3H)
^{13}C NMR (101 MHz, DMSO-d_6) δ (ppm) = 164.33, 158.98, 145.14, 142.71, 136.95, 134.24, 128.07, 127.08, 125.87, 122.17, 119.52, 118.92, 112.35, 106.46, 49.07, 46.08, 43.54, 29.23, 14.40, 13.12
LRMS (ES$^+$) = *m/z* $[M]^+$ calcd. for $C_{20}H_{24}N_3O^+$, 322.19; found 322.26
IR (Smart iTX Diamond) = ν 2973, 2933, 2404, 1621, 1454, 1116, 831, 744 cm^{-1}

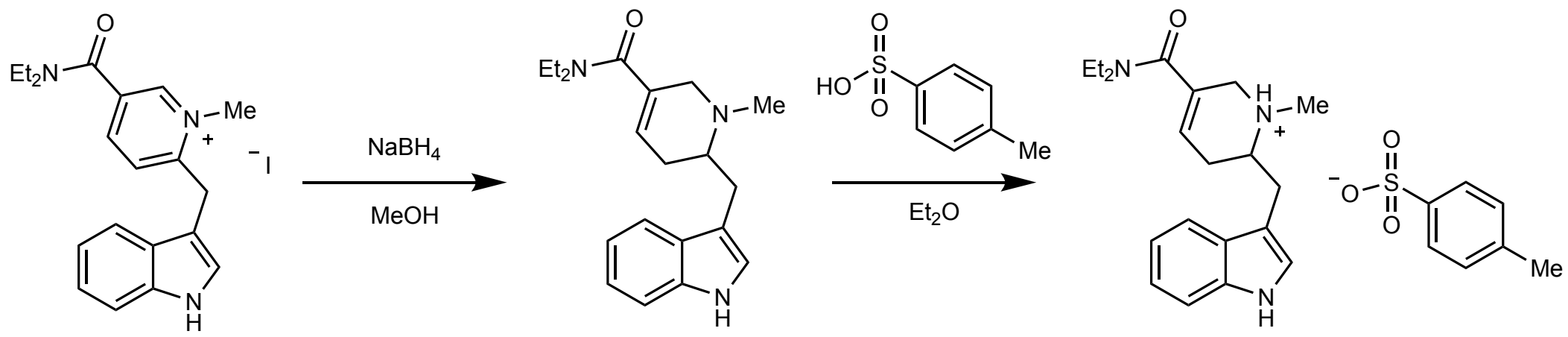

15 **UCD0121 (freebase)** **UCD0121 (tosylate)**

2-((1*H*-indol-3-yl)methyl)-5-(diethylcarbamoyl)-1-methyl-1,2,3,6-tetrahydropyridin-1-ium 4-methylbenzenesulfonate (**UCD0121 tosylate**)

To a stirred solution of compound **15** (1.60 g, 3.56 mmol, 1.00 equiv.) in MeOH (36.0 mL) at 0 °C was added $NaBH_4$ (408 mg, 10.8 mmol, 3.00 equiv.). The mixture was warmed to ambient temperature and stirred for 1 h before being quenched with 1 M NaOH (20 mL). The product was then extracted with DCM (3 x 20 mL). The combined organic extracts were washed with brine (50 mL), dried over Na_2SO_4, filtered, and then concentrated under reduced pressure. The residue was purified by chromatography on silica gel (100% acetone then 90:10:1; DCM/MeOH/$NH_4OH_{(aq)}$) to afford **6-((1*H*-indol-3-yl)methyl)-*N*,*N*-diethyl-1-methyl-1,2,5,6-tetrahydropyridine-3-carboxamide** (**UCD0121 freebase**) (1.11 g, 96%) as an orange oil.

UCD0121 freebase

R_f = 0.35 (acetone)

H NMR (400 MHz, CD_3OD) δ (ppm) = 7.55 (d, *J* = 8.0 Hz, 1H), 7.35 (d, *J* = 8.0 Hz, 1H), 7.14 – 7.06 (m, 2H), 7.02 (td, *J* = 7.5, 1.2 Hz, 1H), 5.88 – 5.80 (m, 1H), 3.53 – 3.38 (m, 5H), 3.31 – 3.15 (m, 2H), 3.02 (td, *J* = 9.7, 5.7 Hz, 1H), 2.73 (dd, *J* = 13.8, 10.5 Hz, 1H), 2.58 (s, 3H), 2.20 – 2.05 (m, 2H), 1.18 (t, *J* = 6.4 Hz, 6H)

The tosylate salt was prepared according to **General Procedure A** using p-toluenesulfonic acid hydrate (87.6 mg, 0.461 mmol, 0.85 equiv.) and **UCD0121 freebase** (176 mg, 0.542 mmol, 1.0 equiv.) with ether as the solvent. The product **UCD0121 tosylate** (111 mg, 41%) was obtained as a light-yellow solid.

Note: DCM/MeOH/$NH_4OH_{(aq)}$ solvent conditions are not necessary but do help elute the product quicker, after the less polar impurities have been removed by the acetone mobile phase.

UCD0121 tosylate

^{1}H NMR (400 MHz, DMSO-d_6) δ (ppm) = 11.07 (s, 1H), 10.02 (d, *J* = 53.2 Hz, 1H), 7.61 (d, *J* = 8.1 Hz, 1H), 7.55 – 7.45 (m, 2H), 7.39 (d, *J* = 8.1 Hz, 1H), 7.31 (dd, *J* = 4.6, 2.4 Hz, 1H), 7.22 – 7.09 (m, 3H), 7.04 (t, *J* = 7.5 Hz, 1H), 5.97 (d, *J* = 12.3 Hz, 1H), 4.12 (t, *J* = 16.2 Hz, 1H), 3.98 – 3.63 (m, 2H), 3.38 (d, *J* = 14.4 Hz, 4H), 3.21 (dd, *J* = 14.1, 4.2 Hz, 1H), 2.97 (dd, *J* = 41.2, 4.8 Hz, 3H), 2.90 – 2.79 (m, 1H), 2.47 – 2.32 (m, 2H), 2.29 (s, 3H), 1.21 – 1.00 (m, *J* = 5.8 Hz, 6H)

^{13}C NMR (101 MHz, CD_3OD, with saturated K2CO3) δ (ppm) = 169.09, 142.13, 140.39, 136.84, 128.51, 128.09, 127.13, 125.58, 124.59, 123.53, 121.34, 118.73, 117.76, 111.23, 108.43, 58.95, 51.24, 43.29, 39.55, 37.86, 25.80, 25.30, 19.95, 14.09, 11.60

LRMS (ES^+) = *m/z* $[M]^+$ calcd. for $C_{20}H_{28}N_3O^+$, 326.22; found, 326.13

HRMS (ES^+) = *m/z* $[M]^+$ calcd. for $C_{20}H_{28}N_3O^+$, 326.2227; found, 326.2228

IR (Smart iTX Diamond) = ν 3266, 3010, 1596, 1457, 1432, 1286, 1213, 1159, 1118, 1031, 1006 cm^{-1}

Ratio of Amine to Acid = 1:1 (Ratio Determined by ^{1}H NMR)

Purity = >99% (Diode Array, 210 nm)

Experimental Procedures for the Synthesis of UCD0068

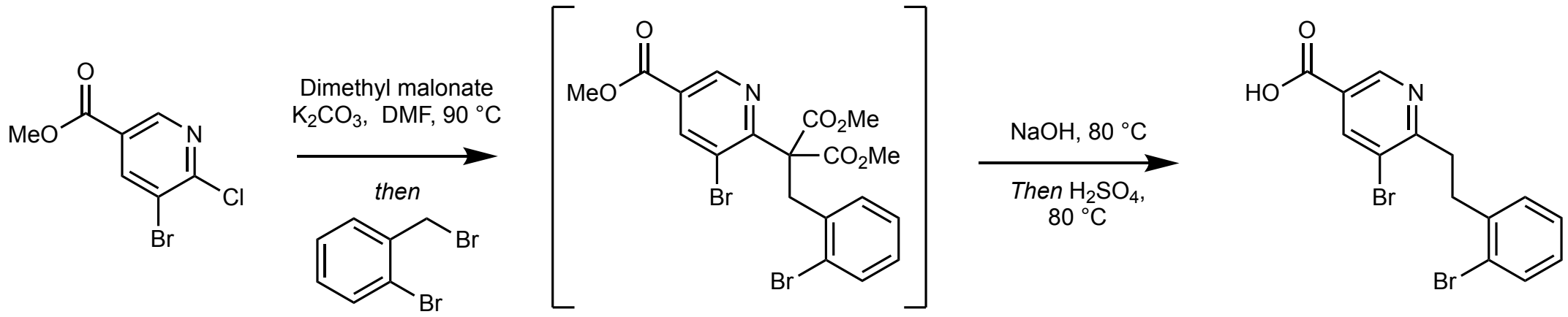

16 S6

5-bromo-6-(2-bromophenethyl)nicotinic acid (S6)

To a stirred mixture of **5-bromo-6-chloronicotinic methyl ester (16)** (5.00 g, 20.0 mmol, 1.00 equiv.) and potassium carbonate (11.0 g, 80.0 mmol, 4.00 equiv.) in DMF (10.0 mL) was added dimethyl malonate (2.39 mL, 20.8 mmol, 1.05 equiv.). The stirring mixture was then heated to 90 °C for 1.5 h before 2-bromobenzylbromide (5.23 g, 20.9 mmol, 1.05 equiv.) was added. The mixture was then stirred for an additional 2 h before being cooled to ambient temperature. The reaction mixture was filtered, and the residue was washed with EtOH (3 x 15.0 mL), then further diluted with toluene (50 mL). The solution was concentrated under reduced pressure at 50 °C. Additional toluene was added as needed to facilitate removal of DMF during concentration. Upon removal of DMF, the residue was diluted with EtOH (40 mL) and H_2O (10 mL). Sodium hydroxide (3.20 g, 80.0 mmol, 4.00 equiv.) was added to the mixture before heating to 80 °C for 2 h. The solution was then cooled to ambient temperature and acidified to pH 3 with concentrated H_2SO_4 (~3.00 mL). The mixture was again heated to 80 °C for 1 h. The solution was cooled to ambient temperature and the pH was adjusted to 3.0. The solution was then cooled to 0 °C to initiate the precipitation of the product. The mixture was filtered and the filter cake was washed with cold water (3 x 10 mL) and cold EtOH (1 x 10 mL) to yield compound **S6** (4.50 g, 58%) as a lime-green solid.

R_f = 0.38 (95:5:1 DCM/MeOH/AcOH)
^{1}H NMR (400 MHz, DMSO-*d*$_6$) δ (ppm) = 13.68 (br, 1H), 8.98 (d, *J* = 1.8 Hz, 1H), 8.36 (d, *J* = 1.8 Hz, 1H), 7.60 (d, *J* = 8.0 Hz, 1H), 7.30(d, *J* = 4.0 Hz, 2H), 7.24 – 7.13 (m, 1H), 3.29 – 3.20 (m, 2H), 3.20 – 3.11 (m, 2H)
^{13}C NMR (101 MHz, DMSO-*d*$_6$) δ (ppm) = 165.49, 162.76, 148.91, 140.92, 140.28, 133.04, 131.21, 128.92, 128.43, 126.77, 124.26, 121.13, 37.49, 34.18
LRMS (ES$^+$) = *m/z* $[M + H]^+$ calcd. for $C_{14}H_{12}Br_2NO_2^+$, 383.92; found, 383.85/386/.12/388.13
IR (Smart iTX Diamond) = ν 3072, 2414, 1888, 1699, 1587, 1459, 1375, 1288, 1234, 1051, 956, 840 cm^{-1}

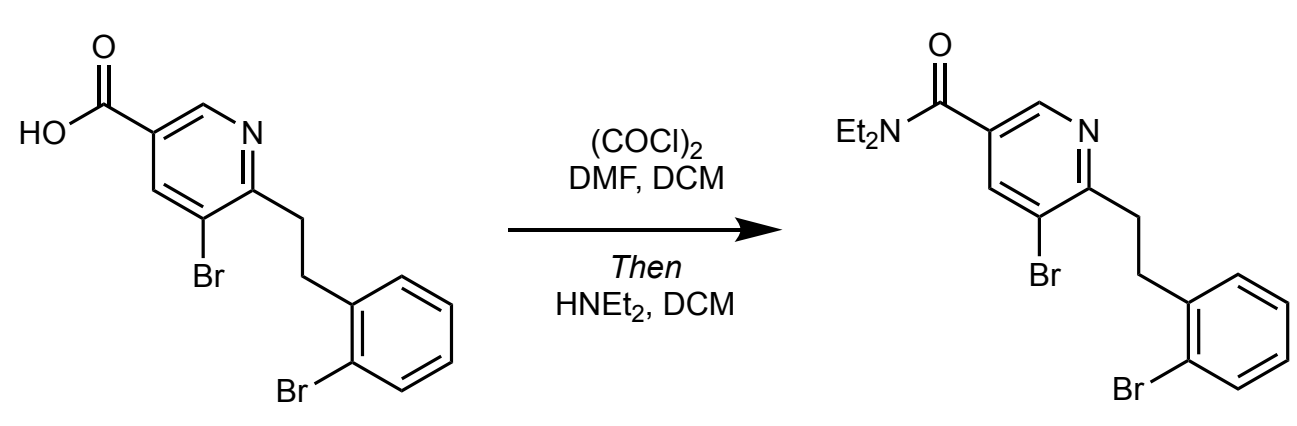

S6 17

5-bromo-6-(2-bromophenethyl)-*N,N*-diethylnicotinamide (17)

To a stirred solution of compound **S6** (4.50 g, 11.7 mmol, 1.00 equiv.) and DMF (0.110 mL) in DCM (30.0 mL) at 0 °C was added $(COCl)_2$ (1.50 mL, 17.5 mmol, 1.50 equiv.) dropwise via addition funnel. The solution was warmed to ambient temperature and stirred for 1 h before being concentrated under reduced pressure. The residue was dissolved with DCM (20.0 mL) and cooled to 0 °C. A solution of diethylamine (3.72 mL, 26.0 mmol, 2.40 equiv.) in DCM (6.00 mL) was added

to the cooled solution via addition funnel over 30.0 min. Saturated aqueous $NaHCO_3$ (20 mL) was added, and the mixture was extracted with DCM (3 x 20 mL). The combined organic extracts were washed with brine (40 mL), dried over Na_2SO_4, filtered, and then concentrated under reduced pressure. The residue was purified by chromatography on silica gel (gradient elution: 10:90 acetone/hexanes → 20:80, acetone/hexanes) to afford compound **17** (4.25 g, 83%) as a lime clear oil.

R_f = 0.40 (25:75 acetone/hexanes)
^{1}H NMR (400 MHz, CDCl$_3$) δ (ppm) = 8.51 (d, *J* = 1.9 Hz, 1H), 7.86 (d, *J* = 1.9 Hz, 1H), 7.53 (d, *J* = 7.9 Hz, 1H), 7.24 – 7.16 (m, 2H), 7.05 (ddd, *J* = 7.9, 6.5, 2.5 Hz, 1H), 3.54 (br, 2H), 3.35 – 3.22 (m, 4H), 3.21 – 3.14 (m, 2H), 1.24 (br, 3H), 1.18 (br, 3H)
^{13}C NMR (101 MHz, CDCl$_3$) δ (ppm) = 167.04, 160.10, 145.12, 140.40, 138.49, 132.85, 132.00, 130.57, 127.93, 127.52, 124.57, 121.28, 43.51, 39.71, 37.46, 34.66, 14.38, 12.85
LRMS (ES$^+$) = *m/z* $[M + H]^+$ calcd. for $C_{18}H_{21}Br_2N_2O^+$, 439.00; found, 438.96/440.91/443.18
IR (Smart iTX Diamond) = ν 2971, 2935, 2242, 1625, 1585, 1425, 1336, 1284, 1216, 1099, 1047 cm^{-1}

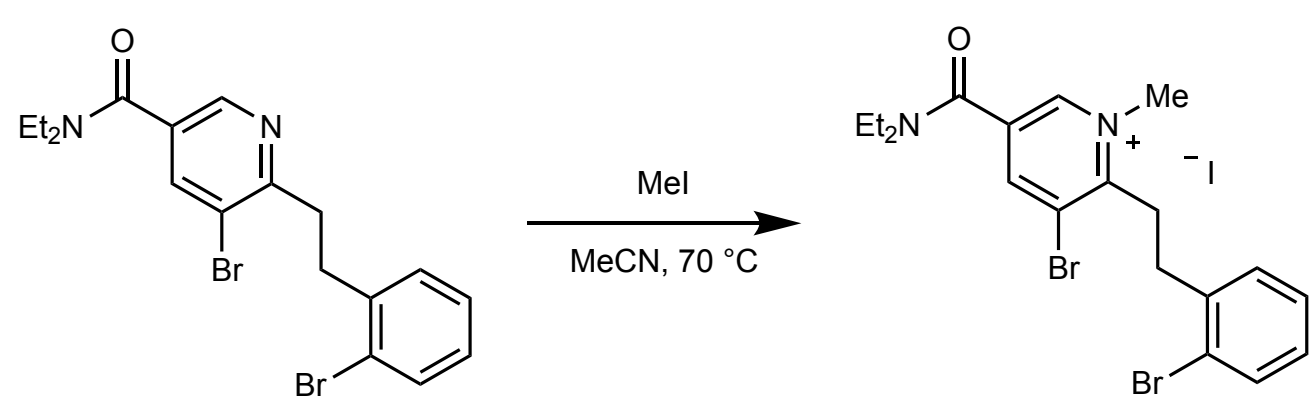

17 **S7**

3-bromo-2-(2-bromophenethyl)-5-(diethylcarbamoyl)-1-methylpyridin-1-ium Iodide (**S7**)
To a stirred solution of compound **17** (900 mg, 2.04 mmol, 1.00 equiv.) in acetonitrile (8.00 mL) was added MeI (0.800 mL, 12.9 mmol, 6.00 equiv.). The vessel was sealed and heated to 70 °C for 17 h before being cooled to ambient temperature. The mixture was concentrated under reduced pressure, diluted with EtOAc (10 mL), and filtered. The filter cake was washed with EtOAc (3 x 5.00 mL) giving compound **S7** (1.15 g, 97%) as a yellow solid, which was used in the next step without further purification.

^{1}H NMR (400 MHz, CD$_3$OD) δ (ppm) = 9.16 (d, *J* = 1.8 Hz, 1H), 8.92 (d, *J* = 1.8 Hz, 1H), 7.63 (dd, *J* = 7.9, 1.2 Hz, 1H), 7.50 (dd, *J* = 7.6, 1.7 Hz, 1H), 7.39 (td, *J* = 7.6, 1.2 Hz, 1H), 7.24 (td, *J* = 7.9, 1.7 Hz, 1H), 4.52 (s, 3H), 3.72 – 3.57 (m, 4H), 3.43 (q, *J* = 7.1 Hz, 2H), 3.33 – 3.27 (m, 2H), 1.31 (t, *J* = 7.1 Hz, 3H), 1.22 (t, *J* = 7.1 Hz, 3H)
^{13}C NMR (101 MHz, CD$_3$OD) δ (ppm) = 163.22, 157.39, 146.54, 144.43, 138.04, 135.24, 132.84, 130.85, 129.01, 128.20, 126.04, 123.77, 47.73, 43.97, 40.04, 33.68, 31.91, 13.02, 11.54
LRMS (ES$^+$) = *m/z* $[M]^+$ calcd. for $C_{19}H_{23}Br_2N_2O^+$, 453.02; found, 452.94/455.02/457.22
IR (Smart iTX Diamond) = ν 2975, 2512, 2067, 1621, 1456, 1440, 1313, 1270, 1106, 1022 cm^{-1}

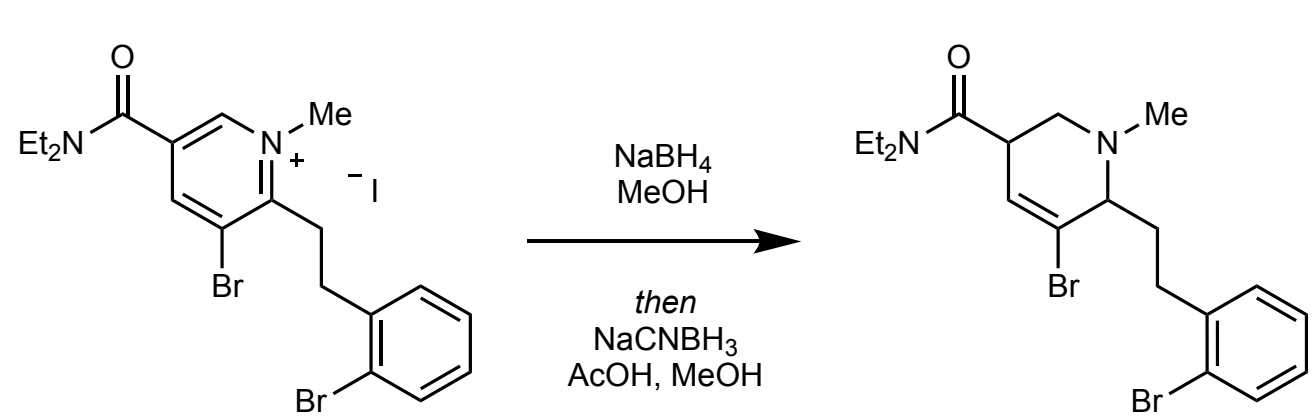

S7 **18**

5-bromo-6-(2-bromophenethyl)-*N*,*N*-diethyl-1-methyl-1,2,3,6-tetrahydropyridine-3-carboxamide (**18**)

To a stirred solution of compound **S7** (0.707 g, 1.21 mmol, 1.00 equiv.) in MeOH (12.0 mL) at 0 °C was added $NaBH_4$ (55.2 mg, 1.46 mmol, 1.20 equiv.). The resulting solution was warmed to ambient temperature and stirred for 1h. Acetic acid (0.700 mL, 12.2 mmol, 10.0 equiv.) and $NaCNBH_3$ (152 mg, 2.42 mmol, 2.00 equiv.) were added to the solution sequentially, and the mixture was stirred for an additional 16 h. The solution was quenched with 1 M NaOH (5.00 mL), and the mixture was extracted with DCM (3 x 10 mL). Next, the organic extracts were combined, washed with brine (20 mL), dried over Na_2SO_4, filtered, and then concentrated under reduced pressure. The residue was purified by chromatography on silica gel (15:85, acetone/hexanes) to afford an inseparable mixture of diastereomers **18** (360 mg, 65%) as a clear oil. Diastereomeric ratio = 1:1, as determined by ^{1}H NMR analysis.

R_f (mixture of diastereomers) = 0.42 (25:75 acetone/hexanes)
^{1}H NMR (400 MHz, $CDCl_3$, mixture of diastereomers) δ (ppm) = 7.50 (d, *J* = 8.0 Hz, 1H), 7.31 – 7.16 (m, 2H), 7.08 – 6.98 (m, 1H), 6.17 (s, 0.5H), 6.00 (s, 0.5H), 3.78 – 3.64 (m, 0.5H), 3.64 – 3.53 (m, 0.50H), 3.43 – 3.30 (m, 4.5H), 3.09 – 2.73 (m, 4H), 2.64 – 2.56 (m, 0.5H), 2.54 (s, 1.5H), 2.48 (s, 1.5H), 2.28 – 2.16 (m, 0.5H), 2.15 – 2.03 (m, 1H), 1.96 – 1.80 (m, 0.5H), 1.29 – 1.17 (m, 3H), 1.17 – 1.04 (m, 3H)
^{13}C NMR (101 MHz, $CDCl_3$, mixture of diastereomers) δ (ppm) = 170.55, 170.46, 142.03, 141.34, 132.75, 132.69, 130.71, 130.57, 129.12, 127.56, 127.53, 127.51, 127.33, 125.56, 124.59, 124.38, 66.55, 65.84, 54.42, 46.26, 43.66, 42.38, 42.01, 41.50, 40.46, 40.26, 36.57, 33.50, 31.63, 30.69, 30.24, 15.03, 14.86, 13.12
LRMS (ES^+, mixture of diastereomers) = *m/z* $[M + H]^+$ calcd. for $C_{19}H_{27}Br_2N_2O^+$, 457.05; found, 457.03/459.05/461.19
IR (Smart iTX Diamond mixture of diastereomers) = ν 2968, 2933, 2796, 1633, 1429, 1378, 1241, 1022, 964, 906, 748, 730 cm^{-1}

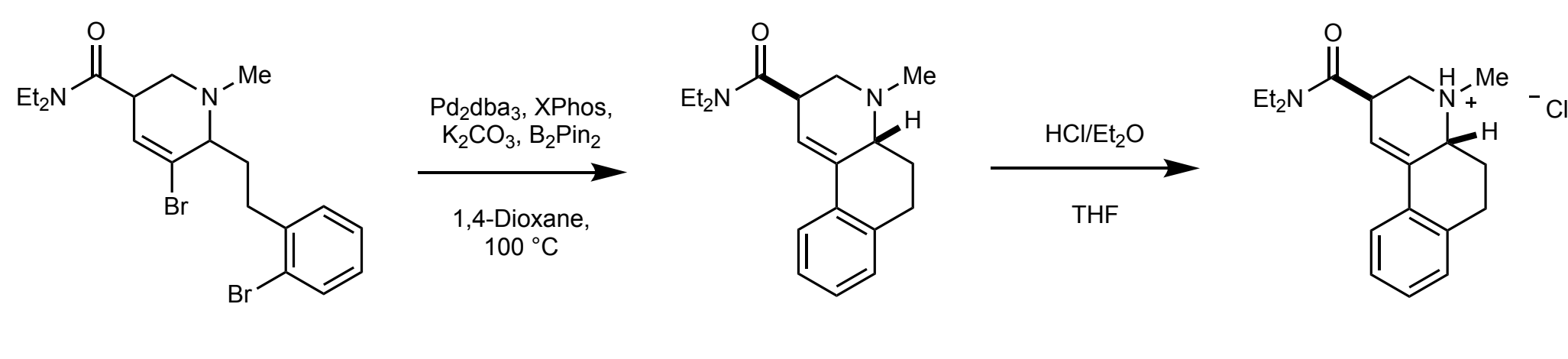

18 UCD0068 (freebase) UCD0068 (hydrochloride)

2-(diethylcarbamoyl)-4-methyl-2,3,4,4a,5,6-hexahydrobenzo[f]quinolin-4-ium hydrochloride (UCD0068 hydrochloride)

To a mixture of B_2Pin_2 (194 mg, 0.764 mmol, 1.00 equiv.), tripotassium phosphate (648 mg, 3.05 mmol, 4.00 equiv.), Xphos (72.8 mg, 0.153 mmol, 0.200 equiv.) and Pd_2dba_3 (70.0 mg, 0.0764 mmol, 0.100 equiv.) in dioxane (7.64 mL) was added compound **18** (350 mg, 0.764 mmol, 1.00 equiv.) as a 1:1 mixture of diastereomers). The vessel was sealed under argon and stirred at 100 °C for 18 h before being concentrated under reduced pressure. The residue was diluted with EtOAc (10 mL) and then extracted with 1 M HCl (3 x 2 mL). Next, the aqueous HCl extracts were combined, basified with 1 M NaOH (pH >10), and extracted with DCM (3 x 5 mL). The DCM extracts were combined, dried over Na_2SO_4, filtered, and then concentrated under reduced pressure. The oil was then purified by chromatography on silica gel (gradient elution: 90:10 EtOAc/MeOH → 75:25 EtOAc/MeOH) to afford ***N*,*N*-diethyl-4-methyl-2,3,4,4a,5,6-hexahydrobenzo[f]quinoline-2-carboxamide** (**UCD0068 freebase**) (85.0 mg, 37%, single diastereomer) as a dark red oil. The *trans* stereochemistry was assigned based on 2D NMR and

NOE analysis. Moreover, the minor diastereomer isomerizes to the more thermodynamically stable *trans* isomer.

UCD0068 freebase
R_f = 0.42 (90:10:1 DCM/MeOH/$NH_4OH_{(aq)}$)
^{1}H NMR (400 MHz, CD_3OD) δ (ppm) = 7.62 (dd, *J* = 6.8, 2.6 Hz, 1H), 7.21 – 7.10 (m, 3H), 6.19 (s, 1H), 3.97 – 3.89 (m, 1H), 3.56 (q, *J* = 7.1 Hz, 2H), 3.50 – 3.40 (m, 2H), 3.06 (ddd, *J* = 11.3, 5.2, 1.3 Hz, 1H), 3.00 – 2.91 (m, 3H), 2.68 (t, *J* = 11.0 Hz, 1H), 2.57 (s, 3H), 2.51 (dt, *J* = 12.5, 3.6 Hz, 1H), 1.62 – 1.51 (m, 1H), 1.31 (t, *J* = 7.1 Hz, 3H), 1.18 (t, *J* = 7.1 Hz, 3H)
^{13}C NMR (101 MHz, CD_3OD) δ (ppm) = 172.35, 136.82, 135.50, 132.58, 128.48, 127.43, 125.87, 124.02, 116.63, 62.74, 54.37, 42.36, 41.73, 40.57, 39.24, 28.13, 27.90, 13.77, 11.92

The hydrochloride salt was prepared according to **General Procedure A** using 2 M ethereal HCl (0.100 mL, 0.200 mmol, 0.900 equiv.) and **UCD0068 freebase** (66.0 mg, 0.221 mmol, 1.00 equiv.) with anhydrous THF as the solvent. Initially, the salt did not precipitate. However, additional anhydrous diethyl ether (~1–2 mL) was added until a precipitate was observed. The mixture was cooled to -22 ºC, filtered, and the filter cake was washed with cold diethyl ether before it was dried under vacuum to yield **UCD0068 hydrochloride** (55.0 mg, 74%) as a yellow powder. The relative configuration was assigned using a combination of DEPT-135, HSQC, 2D NOESY, and 1D NOESY experiments. The data supports an anti-configuration and are in agreement with data previously reported on a related structure. [15]

UCD0068 hydrochloride
^{1}H NMR (400 MHz, CD_3OD with saturated K_2CO_3) δ (ppm) = 7.62 (dd, *J* = 7.4, 1.8 Hz, 1H), 7.22 – 7.06 (m, 3H), 6.18 (s, 1H), 3.96 – 3.87 (m, 1H), 3.56 (q, *J* = 7.1 Hz, 2H), 3.44 (tt, *J* = 13.4, 7.1 Hz, 2H), 3.04 (ddd, *J* = 11.0, 5.3, 1.3 Hz, 1H), 3.00 – 2.85 (m, 3H), 2.64 (t, *J* = 11.0 Hz, 1H), 2.54 (s, 3H), 2.53 – 2.45 (m, 1H), 1.61 – 1.49 (m, 1H), 1.31 (t, *J* = 7.1 Hz, 3H), 1.18 (t, *J* = 7.1 Hz, 3H)
^{13}C NMR (101 MHz, DMSO-*d*$_6$) δ (ppm) = 168.59, 135.30, 132.65, 131.44, 129.39, 128.81, 126.99, 124.86, 118.45, 61.68, 52.64, 42.31, 40.84, 37.03, 27.80, 25.03, 15.18, 13.46 ppm
LRMS (ES$^+$) = *m/z* [M]$^+$ calcd. for $C_{19}H_{27}N_2O^+$, 299.21; found, 299.25
HRMS (ES$^+$) = *m/z* [M]$^+$ calcd. for $C_{19}H_{27}N_2O^+$, 299.2118; found, 299.2122
IR (Smart iTX Diamond mixture of diastereomers) = ν 3706, 3392, 2971, 2865, 2844, 1625, 1454, 1346, 1213, 1132, 1132 cm^{-1}
Ratio of Amine to Acid = 1:1 (Ratio Determined by ^{1}H NMR)
Purity = >99% (Diode Array, 254 nm)

Experimental Procedures for the Synthesis of UCD0076

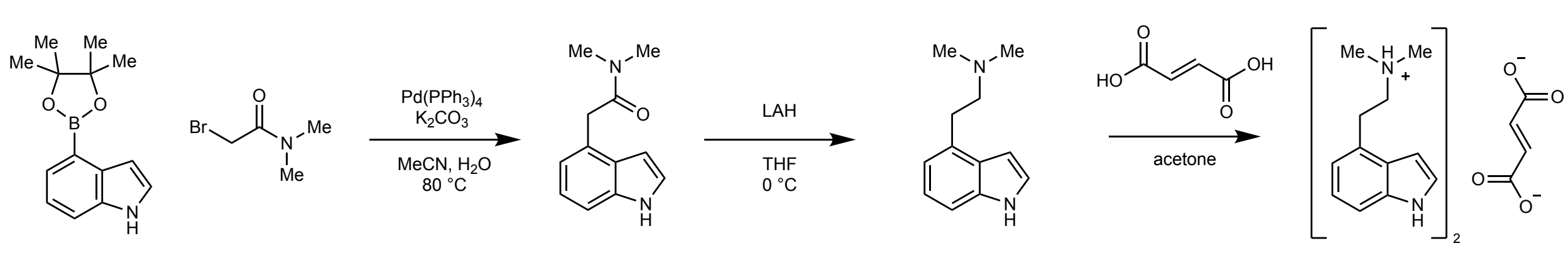

2-(1*H*-indol-4-yl)-*N*,*N*-dimethylethan-1-aminium hemifumarate (**UCD0076 hemifumarate**)
To a stirred solution of **2-bromo-*N*,*N*-dimethylacetamide** (**20**) (830 mg, 5.00 mmol, 1.00 equiv.), K_2CO_3 (1.38 g, 10.0 mmol, 2.00 equiv.), and **4-(4,4,5,5-tetramethyl-1,3,2-dioxaborolan-2-yl)-1*H*-indole** (**19**) (1.22 g, 5.00 mmol, 1.00 equiv.) in acetonitrile (45.0 mL) and H_2O (5.00 mL) was added $Pd(PPh_3)_4$ (57.8 mg, 0.0500 mmol, 0.0500 equiv.). The mixture was heated to 80 °C for 15

h before being filtered through celite. The filtrate was then diluted with saturated aqueous NH_4Cl (30 mL) and extracted with EtOAc (3 x 15 mL). The organic extracts were combined, dried over Na_2SO_4, filtered, and then concentrated under reduced pressure to yield **2-(1*H*-indol-4-yl)-*N*,*N*-dimethylacetamide** (**21**) as a green oil, which was used without further purification We assumed quantitative conversion for the Suzuki reaction. To a stirred solution of unpurified oil (5.00 mmol, 1.00 equiv.) in THF (20.0 mL) at 0 °C was slowly added 2 M lithium aluminum hydride in THF (6.50 mL, 13.0 mmol, 2.60 equiv.). The mixture was then heated to 80 °C and stirred for 5 h. The solution was then cooled to 0 °C and quenched with a dropwise addition of MeOH (3 mL) under an inert atmosphere. The solution was then then further diluted with 2 M NaOH (10 mL) and EtOAc (15 mL) before being filtered through celite. The biphasic layers were separated, and the organic layer was extracted with 2 M HCl (3 x 5.0 mL). The combined aqueous HCl extracts were basified with 2 M NaOH (20 mL) to a to pH >10, before being extracted with DCM (3 x 15 mL). The combined DCM extracts were washed with brine (40 mL), dried over Na_2SO_4, and then concentrated under reduced pressure. The residue was purified by chromatography on silica gel (90:10:1, DCM/MeOH/$NH_4OH_{(aq)}$) to afford **2-(1*H*-indol-4-yl)-*N*,*N*-dimethylethan-1-amine** (**UCD0076 freebase**) (313 mg, 33%) as a white solid.

Note: For safety reasons, it is essential to add lithium aluminum hydride dropwise, as an extreme exotherm is generated upon addition. Additionally, **2-bromo-*N*,*N*-dimethylacetamide** (**20**) could be purchased from SIGMA or prepared following a known procedure.[16]

UCD0076 freebase
R_f = 0.30 (90:10:1 DCM/MeOH/$NH_4OH_{(aq)}$)
^{1}H NMR (300 MHz, $CDCl_3$) δ (ppm) = 8.25 (br, 1H), 7.28 (d, *J* = 8.1 Hz, 1H), 7.21 (t, *J* = 2.9, 1H), 7.13 (dd, J = 7.3, 1.0 Hz, 1H), 6.95 (d, *J* = 8.0 Hz, 1H), 6.61 (td, *J* = 2.1, 1.0 Hz, 1H), 3.16 – 3.05 (m, 2H), 2.76 – 2.65 (m, 2H), 2.37 (s, 6H)

The hemifumarate salt was prepared according to **General Procedure A** using fumaric acid (104 mg, 0.894 mmol, 1.00 equiv.) and **UCD0076 freebase** (168 mg, 0.894 mmol, 1.00 equiv.) with acetone as the solvent. The product **UCD0076 hemifumarate** (106 mg, 43%) was obtained as white powder.

UCD0076 hemifumarate
^{1}H NMR (400 MHz, DMSO-*d*$_6$) δ (ppm) = 11.12 (br, 1H), 7.33 (t, *J* = 2.8 Hz, 1H), 7.26 (d, *J* = 8.2 Hz, 1H), 7.00 (t, *J* = 7.4 Hz, 1H), 6.84 (d, *J* = 7.1 Hz, 1H), 6.54 (s, 1H), 6.53 – 6.48 (m, 1H), 3.14 – 3.00 (m, 2H), 2.93 – 2.79 (m, 2H), 2.47 (s, 6H)
^{13}C NMR (101 MHz, DMSO-*d*$_6$) δ (ppm) = 168.55, 136.27, 135.56, 130.70, 127.58, 125.32, 121.47, 118.98, 110.18, 99.78, 59.01, 44.06, 30.24
LRMS (ES$^+$) = *m/z* $[M]^+$ calcd. for $C_{12}H_{17}N_2^+$, 189.14; found, 189.23
HRMS (ES$^+$) = *m/z* $[M]^+$ calcd. for $C_{12}H_{17}N_2^+$, 189.1386; found, 189.1387
IR (Smart iTX Diamond) = ν 3187, 2925, 1575, 1357, 1249, 1159, 1087, 983, 898, 759, 667 cm^{-1}
Ratio of Amine to Acid = 2:1 (Ratio Determined by ^{1}H NMR)
Purity = >99% (Diode Array, 210 nm)

Experimental Procedures for the Synthesis of UCD0830

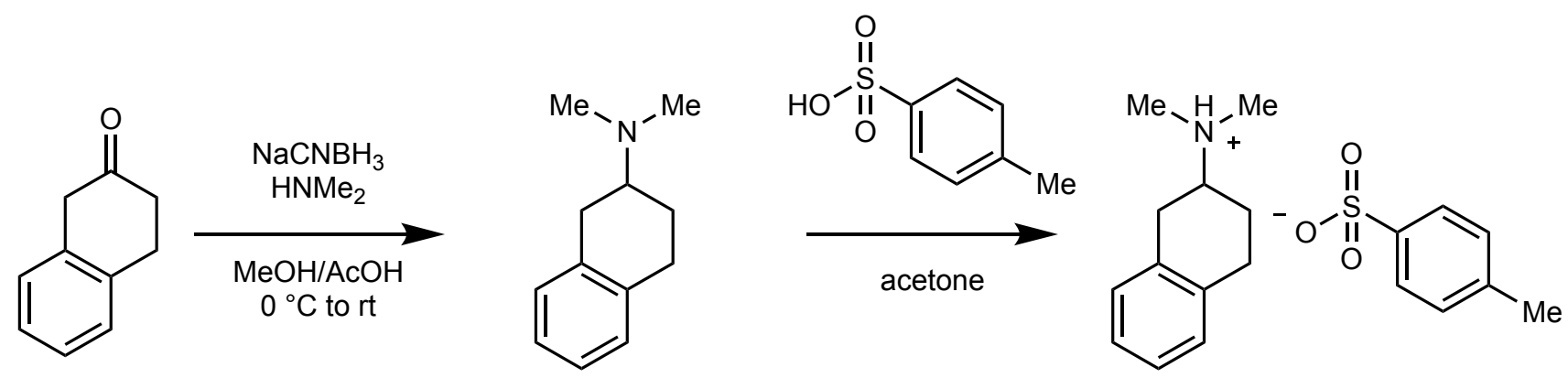

22 UCD0830 (freebase) UCD0830 (tosylate)

***N,N*-dimethyl-1,2,3,4-tetrahydronaphthalen-2-aminium 4-methylbenzenesulfonate** (**UCD0830 tosylate**)

To a stirred solution of **3,4-dihydronaphthalen-2(1*H*)-one** (**22**) (730 mg, 4.99 mmol, 1.00 equiv.), 40% wt. dimethylamine in H_2O (1.13 g, 25.0 mmol, 5.00 equiv.), and acetic acid (0.857 mL, 15.0 mmol, 3.0 equiv.) in MeOH (25.0 mL) at 0 °C was added $NaCNBH_3$ (0.345 g, 5.49 mmol, 1.10 equiv.). The resulting solution was stirred at ambient temperature for 18.5 h before being diluted with saturated aqueous $NaHCO_3$ (20 mL). The aqueous mixture was extracted with DCM (3 x 20 mL). The organic extracts were combined, washed with brine (40 mL), dried over Na_2SO_4, filtered, and then concentrated under reduced pressure. The residue was purified by chromatography on silica gel (90:10:1, DCM/MeOH/$NH_4OH_{(aq)}$) to afford ***N,N*-dimethyl-1,2,3,4-tetrahydronaphthalen-2-amine** (**UCD0830 freebase**) (0.330 mg, 38%) as a red oil.

UCD0830 freebase
R_f (free amine) = 0.33 (90:10:1 DCM/MeOH/$NH_4OH_{(aq)}$)
^{1}H NMR (600 MHz, $CDCl_3$) δ (ppm) = 7.20 – 6.98 (m, 4H), 2.97 – 2.90 (m, 2H), 2.84 (dt, *J* = 11.4, 5.4 Hz, 1H), 2.77 (dd, *J* = 16.6, 11.3 Hz, 1H), 2.64 – 2.58 (m, 1H), 2.37 (s, 6H), 2.15 – 2.07 (m, 1H), 1.62 (qd, *J* = 11.8, 5.5 Hz, 1H)

The tosylate salt was prepared according to **General Procedure A** using *p*-toluenesulfonic acid hydrate (149 mg, 0.781 mmol, 1.00 equiv.) and **UCD0830 freebase** (136 mg, 0.781 mmol, 1.00 equiv.) with acetone as the solvent. The product **UCD0830 tosylate** (229 mg, 84%) was obtained as a white powder.

UCD0830 tosylate
^{1}H NMR (400 MHz, CD_3OD) δ (ppm) = 7.76 – 7.66 (m, 2H), 7.24 (d, *J* = 7.8 Hz, 2H), 7.20 – 7.08 (m, 4H), 3.69 – 3.57 (m, 1H), 3.21 (ddd, *J* = 15.7, 5.5, 2.2 Hz, 2H), 3.10 – 2.97 (m, 3H), 2.96 (s, 6H), 2.38 (s, 3H), 2.37 – 2.28 (m, 1H), 1.97 – 1.83 (m, 1H)
^{13}C NMR (101 MHz, CD_3OD) δ (ppm) = 142.15, 140.29, 134.45, 131.81, 128.95, 128.42, 128.21, 126.47, 126.06, 125.55, 62.65, 39.10, 29.24, 27.55, 23.83, 19.89 ppm
LRMS (ES$^+$) = *m/z* [M]$^+$ calcd. for $C_{12}H_{18}N^+$, 176.14; found, 176.30
HRMS (ES$^+$) = *m/z* [M]$^+$ calcd. for $C_{12}H_{18}N^+$, 176.1434; found, 176.1435
IR (Smart iTX Diamond) = ν 3403, 3043, 2763, 1494, 1214, 1172, 1124, 1035, 1010, 811, 738 cm^{-1}
Ratio of Amine to Acid = 1:1 (Ratio Determined by ^{1}H NMR)
Purity = >99% (ES+)

Experimental Procedures for the Synthesis of UCD0829

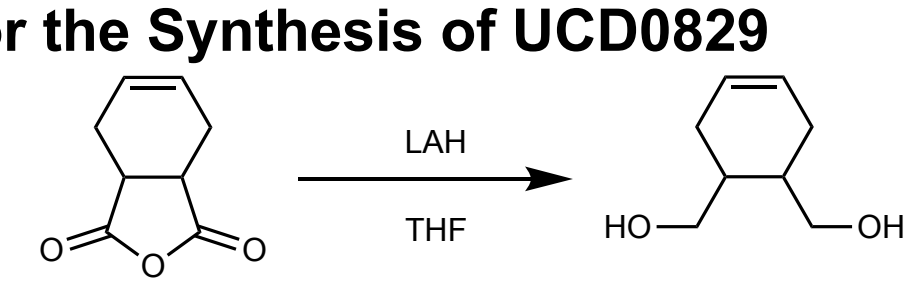

23 24

cyclohex-4-ene-1,2-diyldimethanol (24)
Compound **24** was synthesized following a modified version of a literature procedure.[17] To a stirred solution of **cis-4-cyclohexene-1,2-dicarboxylic anhydride (23)** (7.61 g, 50.0 mmol, 1.00 equiv.) in THF (125 mL) at 0 °C was added 2 M lithium aluminum hydride in THF (50.0 mL, 100 mmol, 2.00 equiv.) dropwise. The solution was warmed to ambient temperature and stirred for 2 h before again being cooled to 0 °C. The cooled solution was quenched with a dropwise addition of THF/H_2O (6:1, 35.0 mL). Deionized H_2O (10.0 mL) and 1 M NaOH (10.0 mL) were sequentially added to the solution. The mixture was stirred for 10 min before filtering through celite. The filtrate was collected and extracted with EtOAc (3 x 40 mL). The organic extracts were combined, washed with brine (100 mL), dried over Na_2SO_4, filtered, and then concentrated under reduced pressure to afford compound **24** (7.50 g, 99%) as a clear oil. The oil was used without further purification.

Note: For safety reasons, it is essential to add lithium aluminum hydride dropwise, as an extreme exotherm is generated upon addition.

R_f = 0.5 (EtOAc)
^{1}H NMR (400 MHz, $CDCl_3$) δ (ppm) = 5.62 (s, 2H), 3.74 (dd, *J* = 11.0, 6.9 Hz, 2H), 3.60 (dd, *J* = 11.0, 3.9 Hz, 2H), 2.72 – 2.49 (m, 2H), 2.20 – 1.94 (m, 6H)
Spectral data are in agreement with the literature.[17]

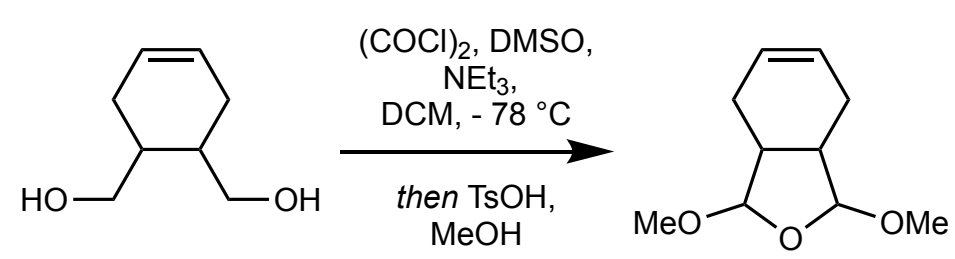

24 25

1,3-dimethoxy-1,3,3a,4,7,7a-hexahydroisobenzofuran (25)
Compound **25** was synthesized following a modified version of a literature procedure.[17] To a stirred solution of $(COCl)_2$ (25.1 mL, 293 mmol, 3.00 equiv.) in DCM (175 mL) at –78 °C was added DMSO (41.9 mL, 590 mmol, 6.00 equiv.) dropwise. The mixture was stirred for 30 min at –78 °C before compound **24** (14.0 g, 98.5 mmol, 1.00 equiv.) in DCM (248 mL) was added to the solution via cannulation. The resulting solution was stirred for 1.5 h at –78 °C before the addition of NEt_3 (102 mL, 732 mmol, 7.50 equiv.). The mixture was warmed to ambient temperature and stirred for an additional 1 h. The mixture was then cooled to 0 °C and diluted with p-toluenesulfonic acid hydrate (67.0 g, 352 mmol, 3.50 equiv.) in MeOH (148 mL). The resulting solution was then warmed to ambient temperature and stirred 12 h. The reaction was quenched with saturated aqueous NH_4Cl (300 mL) and extracted with DCM (3 x 200 mL). The organic extracts were combined, concentrated under reduced pressure, and then diluted with EtOAc (400 mL). The solution was washed with brine (3 x 150 mL), dried over Na_2SO_4, filtered, and then concentrated under reduced pressure. The residue was purified by chromatography on silica gel (1:15 ether/pentane) to afford compound **25** (12.1 g, 67%) as a clear yellow oil.

Note: Gas pressure build up was observed upon addition of DMSO.

R_f = 0.16 (1:15 ether/pentane)
^{1}H NMR (400 MHz, $CDCl_3$) δ (ppm) = 5.68 – 5.55 (m, 2H), 4.63 (d, *J* = 2.9 Hz, 2H), 3.34 (s, 6H), 2.44 – 2.27 (m, 2H), 2.24 – 2.07 (m, 2H), 1.94 – 1.80 (m, 2H)
Spectral Data are in agreement with the literature.[17]

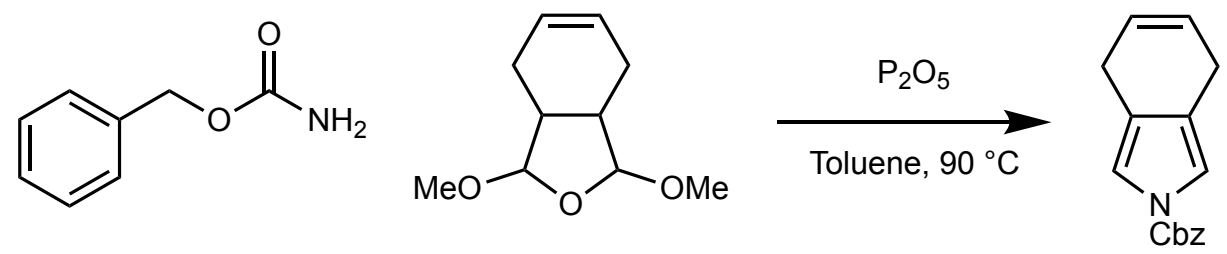

S8 25 26

benzyl 4,7-dihydro-2*H*-isoindole-2-carboxylate (26)

To a stirring solution of P_2O_5 (8.85 g, 62.4 mmol, 3.50 equiv.), **benzyl carbamate** (**S8**) (2.71 g, 17.9 mmol, 1.00 equiv.) in toluene (80.0 mL) was added a solution of compound **25** (3.30 g, 17.9 mmol, 1.00 equiv.) in toluene (10.0 mL). The solution was heated to 90 °C for 2 h, before being cooled to ambient temperature and filtered. The organic mixture was washed with brine (20 mL), then concentrated under reduced pressure. The residue was purified by chromatography on silica gel (1:15 ether/pentane) to afford compound **26** (2.30 g, 51%) as a lime green solid.

R_f = 0.40 (1:15 ether/pentane)
^{1}H NMR (400 MHz, $CDCl_3$) δ (ppm) = 7.54 – 7.33 (m, 5H), 7.11 (s, 2H), 5.91 (s, 2H), 5.39 (s, 2H), 3.23 (s, 4H)
^{13}C NMR (101 MHz, $CDCl_3$) δ (ppm) = 150.53, 135.24, 128.72, 128.64, 128.34, 124.15, 121.54, 115.00, 68.61, 22.20
LRMS (ES$^+$) = *m/z* $[M + H]^+$ calcd. for $C_{16}H_{16}NO_2^+$, 254.12; found, 254.24
IR (Smart iTX Diamond) = ν 3029, 2888, 2825, 1735, 1409, 1359, 1294, 1224, 977, 750, 732 cm^{-1}

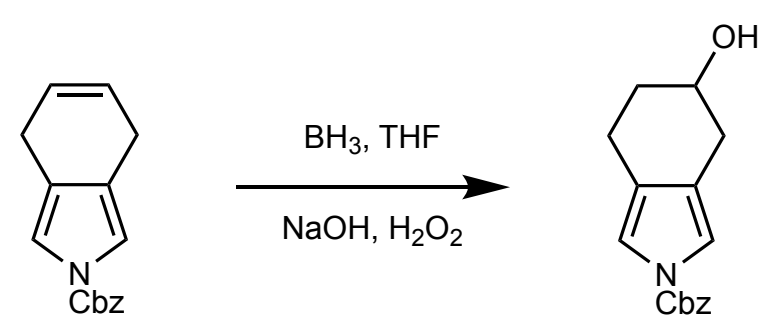

26 27

benzyl 5-hydroxy-4,5,6,7-tetrahydro-2*H*-isoindole-2-carboxylate (27)

To a stirred solution of compound **26** (2.14 g, 8.45 mmol, 1.00 equiv.) in THF (11.3 mL) at 0 °C was added 1.00 M borane in THF (9.09 mL, 9.09 mmol, 1.08 equiv.) dropwise. The mixture was warmed to ambient temperature and stirred for 4 h. The mixture was cooled to 0 °C before 1 M NaOH (8.63 mL, 8.63 mmol, 1.02 equiv.) and 30 wt% aqueous H_2O_2 (5.00 mL) were added sequentially. The mixture was warmed to ambient temperature and stirred for 12 h. The solution was then transferred to a separatory funnel and was extracted with ether (3 x 10 mL). The organic extracts were washed with brine (30 mL), dried over Na_2SO_4, filtered, and then concentrated under reduced pressure. The residue was purified by chromatography on silica gel (50:50 EtOAc/Hexanes) to afford compound **27** (1.98 g, 86%) as a clear oil.

R_f = 0.40 (50:50 EtOAc/hexanes)
^{1}H NMR (400 MHz, $CDCl_3$) δ (ppm) = 7.54 – 7.30 (m, 5H), 7.00 (d, *J* = 6.2 Hz, 2H), 5.33 (s, 2H), 4.16 – 4.02 (m, 1H), 2.88 (dd, *J* = 15.8, 4.8 Hz, 1H), 2.74 (dt, , *J* = 16.4, 5.9 1H), 2.65 – 2.45 (m, 2H), 2.03 – 1.89 (m, 1H), 1.87 – 1.73 (m, 2H)
^{13}C NMR (101 MHz, $CDCl_3$) δ (ppm) = 150.31, 135.15, 128.70, 128.64, 128.31, 122.83, 122.26, 115.93, 115.12, 68.61, 67.79, 31.58, 31.22, 18.86
LRMS (ES$^+$) = *m/z* $[M + H]^+$ calcd. for $C_{16}H_{18}NO_3^+$, 272.13; found, 272.19
IR (Smart iTX Diamond) = ν 3365, 2927, 1735, 1413, 1346, 1232, 971, 750, 696 cm^{-1}

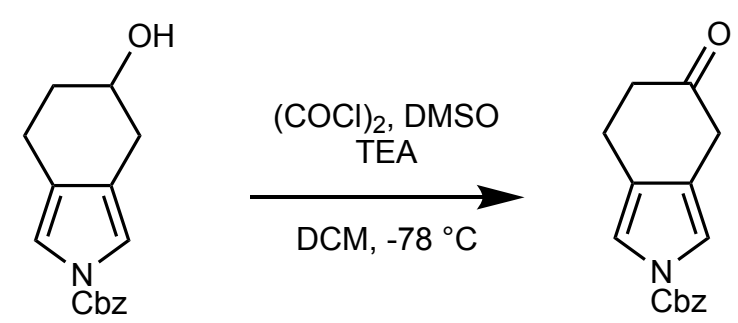

27 28

benzyl 5-oxo-4,5,6,7-tetrahydro-2*H*-isoindole-2-carboxylate (28)
To a stirred solution of $(COCl)_2$ (0.876 mL, 10.3 mmol, 1.50 equiv.) in DCM (12.3 mL) at -78 °C was added DMSO (1.46 mL, 20.6 mmol, 3.00 equiv.) dropwise. The solution was stirred for 30 min at -78 °C before compound **27** (1.85 g, 6.82 mmol, 1.00 equiv.) in DCM (17.2 mL) was added. The mixture was stirred for 1 h at -78 °C, then NEt_3 (3.27 mL, 23.5 mmol, 3.50 equiv.) was added. The solution was warmed to ambient temperature and stirred for 1 h before being quenched with saturated aqueous NH_4Cl (60.0 mL). The solution was extracted with DCM (3 x 30 mL). The organic extracts were combined and washed with saturated aqueous $NaHCO_3$ (40 mL), brine (40 mL), dried over Na_2SO_4, filtered, and then concentrated under reduced pressure. The residue was purified by chromatography on silica gel (gradient elution: 12.5:87.5 EtOAc/hexanes → 25:75 EtOAc/hexanes) to afford compound **28** (0.951 g, 52%) as a yellow oil.

Note: Gas pressure build up was observed upon addition of DMSO.

R_f = 0.43 (25:75 EtOAc/hexanes)
^{1}H NMR (400 MHz, $CDCl_3$) δ (ppm) = 7.47 – 7.32 (m, 5H), 7.09 (d, *J* = 7.2 Hz, 2H), 5.35 (s, 2H), 3.42 (dd, *J* = 1.0, 2H), 2.87 (dd, *J* = 7.2, 1.0 Hz, 2H), 2.56 (dd, *J* = 7.2, 5.6 Hz, 2H)
^{13}C NMR (101 MHz, $CDCl_3$) δ (ppm) = 209.88, 150.20, 134.93, 128.77, 128.75, 128.41, 122.93, 121.33, 116.01, 115.02, 68.89, 39.71, 37.39, 20.54
LRMS (ES$^+$) = *m/z* $[M + H]^+$ calcd. for $C_{16}H_{16}NO_3^+$, 270.11; found, 270.17
IR (Smart iTX Diamond) = ν 2958, 1735, 1708, 1419, 1359, 1324, 1230, 981, 763, 698 cm^{-1}

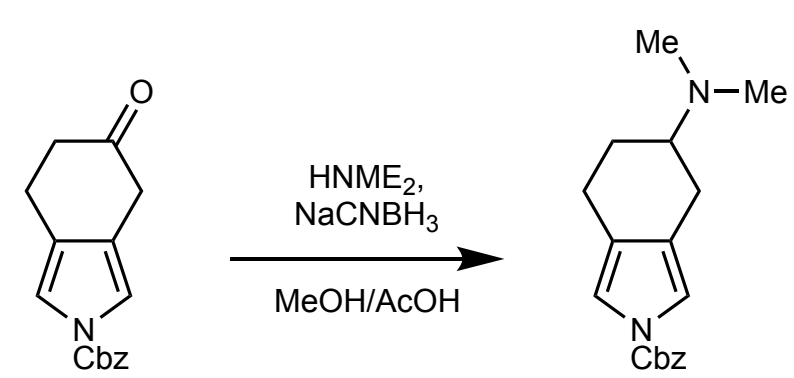

28 29

benzyl 5-(dimethylamino)-4,5,6,7-tetrahydro-2*H*-isoindole-2-carboxylate (29)
To a stirred solution of compound **28** (289 mg, 1.07 mmol, 1.00 equiv.), acetic acid (0.328 mL, 0.328 mmol, 5.75 equiv.) and dimethylamine (2.00 M in THF, 2.30 mL, 4.60 mmol, 4.00 equiv.) in MeOH (5.75 mL) at 0 °C was added $NaCNBH_3$ (108 mg, 1.72 mmol, 1.40 equiv.). The mixture was warmed to ambient temperature and stirred for 3 h. The solution was then concentrated under reduced pressure and diluted with saturated aqueous $NaHCO_3$ (3.00 mL). The mixture was extracted with DCM (3 x 4.0 mL). The organic extracts were combined, washed with brine (10 mL), dried over Na_2SO_4, filtered, and then concentrated under reduced pressure. The residue was purified by chromatography on silica gel (90:10:1 DCM/MeOH/$NH_4OH_{(aq)}$) to afford compound **29** (0.166 mg, 52%) as a brown oil.

R_f = 0.35 (90:10:1 DCM/MeOH/$NH_4OH_{(aq)}$)
^{1}H NMR (600 MHz, $CDCl_3$) δ (ppm) = 7.46 – 7.36 (m, 5H), 7.03 (d, *J* = 16.1 Hz, 2H), 5.36 (s, 2H), 3.06 – 2.92 (m, 2H), 2.92 – 2.77 (m, 1H), 2.74 – 2.51 (m, 8H), 2.25 (d, *J* = 11.2 Hz, 1H), 1.76 – 1.63 (m, 1H)

^{13}C NMR (150 MHz, CDCl$_3$) δ (ppm) = 150.17, 135.02, 128.72, 128.33, 122.38, 121.52, 116.04, 115.26, 68.75, 62.32, 40.59, 29.71, 25.66, 23.44, 20.93
LRMS (ES$^+$) = *m/z* [M + H]$^+$ calcd. for $C_{18}H_{23}N_2O_2^+$, 299.18; found, 299.25
IR (Smart iTX Diamond) = ν 2923, 2852, 2773, 1739, 1456, 1415, 1353, 1332, 1236, 975 cm^{-1}

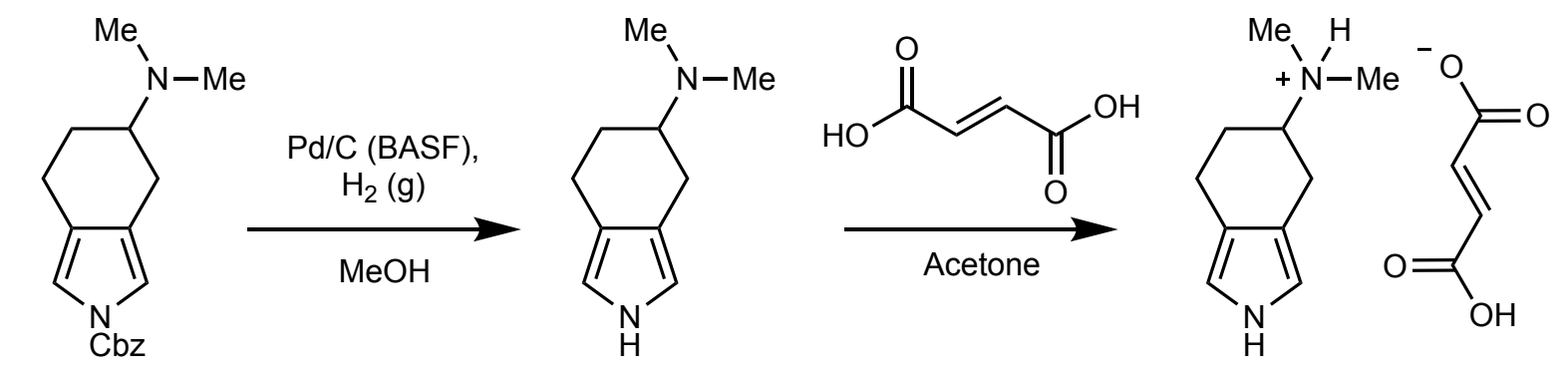

***N*,*N*-dimethyl-4,5,6,7-tetrahydro-2*H*-isoindol-5-aminium fumarate** (**UCD0829 fumarate**)
Under inert atmosphere, to a stirring solution of unreduced 10 wt % Pd/C catalyst (16.5 mg, 0.155 mmol, 0.279 equiv.) in MeOH (5.60 mL) was added compound **29** (155 mg, 0.519 mmol, 1.00 equiv.) dissolved in MeOH (3.0 mL). The mixture was continually sparged with H_2 (1.00 atm) for 10 min then placed under a hydrogen atmosphere. After 4 h, the mixture was filtered, the filtrate was basified with 1 M NaOH (4.00 mL), and then extracted with DCM (3 x 3.0 mL). The organic extracts were combined and dried over Na_2SO_4, filtered, and then concentrated under reduced pressure. The residue was purified by chromatography on silica gel (90:10:1 DCM/MeOH/$NH_4OH_{(aq)}$) to afford ***N*,*N*-dimethyl-4,5,6,7-tetrahydro-2*H*-isoindol-5-amine** (**UCD0829 freebase**) (45.5 mg, 53%) as a brown solid.

Note: The source of the Pd/C catalyst had a dramatic impact on reaction efficiency. The Pd/C catalyst was purchased from BASF.

UCD0829 freebase
R_f = 0.17 (90:10:1 DCM/MeOH/$NH_4OH_{(aq)}$)
^{1}H NMR (400 MHz, CD$_3$OD) δ (ppm) = 6.53 (S, 1H), 6.47 (s, 1H), 2.95 – 2.86 (m, 1H), 2.84 – 2.69 (m, 2H), 2.65 – 2.53 (m, 2H), 2.45 (s, 6H), 2.15 – 2.08 (m, 1H), 1.66 – 1.55 (m, 1H)

The fumarate salt was prepared according to **General Procedure A** using fumaric acid (26.4 mg, 0.227 mmol, 0.820 equiv.) and **UCD0829 freebase** (45.5 mg, 0.277 mmol, 1.00 equiv.) with acetone as the solvent. The product **UCD0829 fumarate** (36.0 mg, 46%) was obtained as a pink solid.

UCD0829 fumarate
^{1}H NMR (400 MHz, CD$_3$OD) δ (ppm) = 6.71 (s, 2H), 6.53 (s, 1H), 6.47 (s, 1H), 3.59 – 3.45 (m, 1H), 3.15 – 3.06 (m, 1H), 2.92 (s, 6H), 2.89 – 2.78 (m, 2H), 2.78 – 2.64 (m, 1H), 2.33 – 2.20 (m, 1H), 1.95 – 1.80 (m, 1H)
^{13}C NMR (101 MHz, CD$_3$OD) δ (ppm) = 170.02, 134.81, 115.71, 113.61, 113.54, 112.82, 64.31, 38.99, 25.34, 22.75, 20.31
LRMS (ES$^+$) = *m/z* [M]$^+$ calcd. for $C_{10}H_{17}N_2^+$, 165.14; found, 165.25
HRMS (ES$^+$) = *m/z* [M]$^+$ calcd. for $C_{10}H_{17}N_2^+$, 165.1386; found, 165.1390
IR (Smart iTX Diamond) = ν 3268, 3033, 2931, 2856, 2478, 1700, 1665, 1371, 1255, 1174, 975 cm^{-1}
Ratio of Amine to Acid = 1:1 (Ratio Determined by ^{1}H NMR)
Purity = >99% (ES +)

Experimental Procedures for the Synthesis of UCD0162

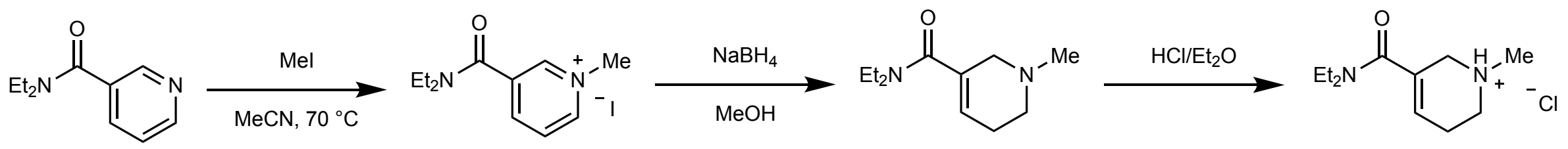

30 31 UCD0162 (Freebase) UCD0162 (Hydrochloride)

3-(diethylcarbamoyl)-1-methyl-1,2,5,6-tetrahydropyridin-1-ium-2-ylium hydrochloride (UCD0162 hydrochloride)

To a stirred mixture of ***N,N*-diethylnicotinamide** (**30**) (1.23 g, 6.90 mmol, 1.00 equiv.) in acetonitrile (13.0 mL) was added MeI (6.50 mL, 104 mmol, 15.1 equiv.). The vessel was sealed and heated to 70 °C and stirred for 22 h. The mixture was then cooled to ambient temperature, and concentrated under reduced pressure. The residue was diluted with EtOAc, the mixture was filtered, and the filter cake was washed with EtOAc (3 x 5.00 mL) yielding **3-(diethylcarbamoyl)-1-methylpyridin-1-ium iodide** (**31**) (1.94 g, 88%) as a yellow crystalline solid, which was used without further purification. To a stirred solution of compound **31** (1.86 g, 5.83 mmol, 1.00 equiv.) in MeOH (58.3 mL) at 0 °C was added $NaBH_4$ (0.662 g, 17.5 mmol, 3.00 equiv.). The solution was stirred for 21 h at ambient temperature before being concentrated under reduced pressure. The residue was diluted with 1 M NaOH (15 mL) and then extracted with DCM (3 x 10 mL). The combined organic extracts were washed with brine (20 mL), dried over Na_2SO_4, filtered, and then concentrated under reduced pressure. The residue was purified by chromatography on silica gel (95:5:1 DCM/MeOH/$NH_4OH_{(aq)}$ → 90:10:1 DCM/MeOH/$NH_4OH_{(aq)}$) to afford ***N,N*-diethyl-1-methyl-1,2,5,6-tetrahydropyridine-3-carboxamide** (**UCD0162 freebase**) (0.416 mg, 36%) as a red oil.

Note: Addition of $NaBH_4$ into mixture results in a violent exothermic reaction.

UCD0162 freebase

R_f = 0.23 (90:10:1 DCM/MeOH/$NH_4OH_{(aq)}$)

^{1}H NMR (400 MHz, CD_3OD) δ (ppm) = 5.91 (dp, *J* = 3.8, 1.9 Hz, 1H), 3.45 (d, *J* = 6.6 Hz, 4H), 3.10 (q, *J* = 2.7 Hz, 2H), 2.62 (t, *J* = 5.8 Hz, 2H), 2.41 (s, 3H), 2.33 (tq, *J* = 5.8, 2.7 Hz, 2H), 1.18 (t, *J* = 7.2 Hz, 6H)

The hydrochloride salt was prepared according to **General Procedure A** using ethereal 1 M HCl (0.430 mL, 1.00 equiv.) and **UCD0162 freebase** (85.0 mg, 0.434 mmol, 1.00 equiv.) with ether as the solvent. The product **UCD0162 hydrochloride** (49.1 mg, 49%) was obtained as a white solid

UCD0162 hydrochloride

^{1}H NMR (400 MHz, DMSO-d_6) δ (ppm) = 11.26 (br, 1H), 6.00 (s, 1H), 3.86 – 3.65 (m, 2H), 3.62 – 3.18 (m, 5H), 3.16 – 3.01 (m, 1H), 3.0 – 2.73 (m, 3H), 2.70 – 2.53 (m, 1H), 2.47 – 2.27 (d, *J* = 18.7Hz 1H), 1.08 (t, *J* = 6.7 Hz, 6H)

^{13}C NMR (101 MHz, Acetic Acid-d_4) δ (ppm) = 169.03, 127.31, 125.41, 51.45, 49.62, 43.71, 42.21, 39.63, 21.97, 13.50, 11.67

LRMS (ES^+) = *m/z* $[M]^+$ calcd. for $C_{11}H_{21}N_2O^+$, 197.16; found, 197.23

HRMS (ES^+) = *m/z* $[M]^+$ calcd. for $C_{11}H_{21}N_2O^+$, 197.1648; found, 197.1649

IR (Smart iTX Diamond) = ν 3400, 2935, 2483, 1604, 1432, 1382, 1284, 1216, 1097, 1010, 985 cm^{-1}

Ratio of Amine to Acid = 1:1 (Ratio Determined by ^{1}H NMR)

Purity = 98.7% (Diode Array, 210 nm)

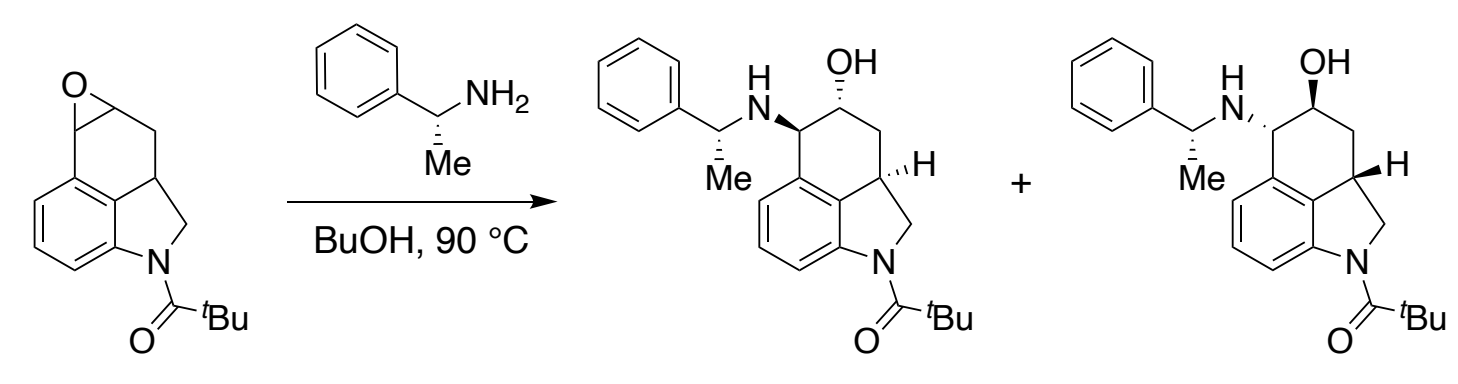

1-((2aR,4R,5R)-4-hydroxy-5-(((R)-1-phenylethyl)amino)-2a,3,4,5-tetrahydrobenzo[cd]indol-1(2H)-yl)-2,2-dimethylpropan-1-one (**32**)
1-((2aS,4S,5S)-4-hydroxy-5-(((R)-1-phenylethyl)amino)-2a,3,4,5-tetrahydrobenzo[cd]indol-1(2H)-yl)-2,2-dimethylpropan-1-one (**33**)
To a solution of **6** (8.29 g, 32.1 mmol, 1.00 equiv.) in butanol (107 mL) was added (1*R*)-(+)-phenylethylamine (22.0 mL, 171 mmol, 5.30 equiv.). The mixture was then heated to 90 °C for 24 h. Toluene (100 mL) was added to the mixture at ambient temperature. The mixture was then concentrated under reduced pressure to remove excess unreacted amine. This process was repeated two more times. The residue was taken up in EtOAc (20 mL), and the mixture was stored at –22 ºC for 18 h. The mixture was then filtered and the solid (diastereomerically enriched **33**) was collected. The filtrate was concentrated and purified by chromatography on silica gel (gradient elution: 75:25 hexanes/EtOAc to 50:50 hexanes/EtOAc) to yield diastereomerically enriched **32**. A subsequent recrystallization of **32** with EtOAc/Hexanes yielded **32** as a white solid with >95:5 d.r. (3.23 g, 27%). Recrystallization of **33** with EtOAc/EtOH yielded a white solid with >95:5 d.r. (4.18 g, 34%)

The absolute stereochemistry was determined retrospectively based on X-ray crystal structures of **37** and **38**.

32:
R_f = 0.30 (hexanes/EtOAc, 50:50 v/v)
^{1}H NMR (600 MHz, CDCl3) δ (ppm) = 7.91 (d, J = 7.9 Hz, 1H), 7.44 – 7.35 (m, 4H), 7.36 – 7.27 (m, 1H), 7.14 (t, J = 8.4 Hz, 1H), 6.80 (d, J = 7.6 Hz, 1H), 4.61 (t, J = 8.7 Hz, 1H), 4.11 – 4.00 (m, 2H), 3.60 (dd, J = 12.0, 9.3 Hz, 1H), 3.56 – 3.48 (m, 1H), 3.47 (d, J = 3.8 Hz, 1H), 2.24 – 2.15 (m, 1H), 1.87 – 1.81 (m, 1H), 1.37 (d, J = 6.7 Hz, 3H), 1.36 (s, 9H)
^{13}C NMR (150 MHz, CDCl3) δ (ppm) = 176.43, 145.47, 142.96, 134.49, 131.22, 128.77, 128.44, 127.41,126.70, 121.62, 116.57, 69.65, 57.94, 57.91, 56.74, 39.86, 32.39, 29.45, 27.63, 24.38
LRMS (ES+) = *m/z* $[M]^+$ calcd for $C_{24}H_{31}N_2O^+$ = 379.24; Found 379.47
IR (Smart iTX Diamond) = ν 3432, 2968, 2927, 2871, 1619, 1591 cm^{-1}

33:
R_f = 0.30 (hexanes/EtOAc, 50:50 v/v)
^{1}H NMR (600 MHz, CDCl3) δ (ppm) = 7.98 (d, J = 7.9 Hz, 1H), 7.38 (d, J = 7.1 Hz, 2H), 7.34 (t, J = 7.2 Hz, 2H), 7.28 – 7.23 (m, 2H), 7.03 (d, J = 7.4 Hz, 1H), 4.62 (t, J = 8.9 Hz, 1H), 4.27 – 4.20 (m, 1H), 3.65 – 3.57 (m, 2H), 3.44 (q, J = 9.4, 9.0 Hz, 1H), 3.39 (d, J = 5.7 Hz, 1H), 2.74 (br, 1H), 2.08 – 1.98 (m, 1H), 1.85 – 1.79 (m, 1H), 1.62 (br, 1H), 1.48 (d, J = 6.1 Hz, 3H), 1.36 (s, 9H)
^{13}C NMR (150 MHz, CDCl3) δ (ppm) = 176.49, 145.36, 143.17, 135.57, 131.01, 128.63, 128.46, 127.33, 126.97, 119.65, 116.73, 71.22, 59.46, 58.27, 57.64, 39.90, 32.69, 30.53, 27.61, 25.03
LRMS (ES+) = *m/z* $[M]^+$ calcd for $C_{24}H_{31}N_2O^+$ = 379.24; Found 379.42
IR (Smart iTX Diamond) = ν 3436, 2966, 2927, 2871, 1619, 1589 cm^{-1}

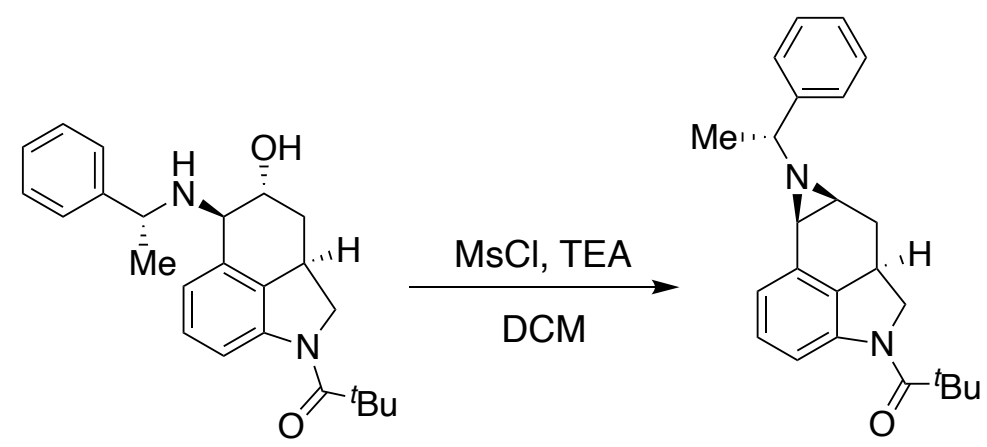

32 34

2,2-dimethyl-1-((5aR,6aS,7aR)-7-((R)-1-phenylethyl)-5,5a,6,6a,7,7a-hexahydro-4H-azirino[2',3':4,5]benzo[1,2,3-cd]indol-4-yl)propan-1-one (34)

To a 0 °C cooled solution of **32** (3.24 g, 8.56 mmol,1.00 equiv.) in DCM (80.0 mL) were added triethylamine (3.72 mL, 26.5 mmol, 3.10 equiv.) and methane sulfonyl chloride (0.990 mL, 12.9 mmol, 1.50 equiv.). The reaction was warmed to ambient temperature and stirred for 2 h before being quenched with H_2O (16 mL). The solution was then extracted with DCM (3 x 20 mL) and the combined organic extracts were dried over Na_2SO_4. The organic solution was then concentrated under reduced pressure. The residue was purified by chromatography on silica gel (90:10 hexanes/EtOAc) then recrystallized in hexanes to afford **34** (2.74g, 89%, >95:5 d.r. **34**:**38**) as a white solid.

Note: Slight heating may be required to fully dissolve the starting material prior to the addition of triethylamine or methane sulfonyl chloride.

R_f = 0.42 (hexanes/EtOAc, 75:25 v/v)
^{1}H NMR (600 MHz, CDCl3) δ (ppm) = 7.95 (d, J = 7.6 Hz, 1H), 7.41 – 7.32 (m, 4H), 7.30 – 7.25 (m, 1H), 7.10 (t, J = 7.6 Hz, 1H), 6.73 (d, J = 7.6 Hz, 1H), 4.54 (t, J = 8.6 Hz, 1H), 3.51 (t, J = 11.0 Hz, 1H), 3.48 – 3.40 (m, 1H), 2.82 – 2.71 (m, 2H), 2.32 (d, J = 5.2 Hz, 1H), 2.21 (s, 1H), 1.71 (t, J = 12.5 Hz, 1H), 1.48 (d, J = 5.5 Hz, 3H), 1.35 (s, 9H)
^{13}C NMR (150 MHz, CDCl3) δ (ppm) = 176.29, 144.37, 143.29, 132.22, 132.16, 128.37, 128.28, 127.01, 126.92, 123.86, 117.08, 69.97, 57.39, 39.87, 39.10, 37.19, 35.41, 31.48, 27.67, 23.38
LRMS (ES+) = *m/z* $[M]^+$ calcd. for $C_{24}H_{29}N_2O^+$ = 361.23; Found 361.36
IR (Smart iTX Diamond) = ν 2969, 2923, 2869, 1641, 1619, 1589 cm^{-1}

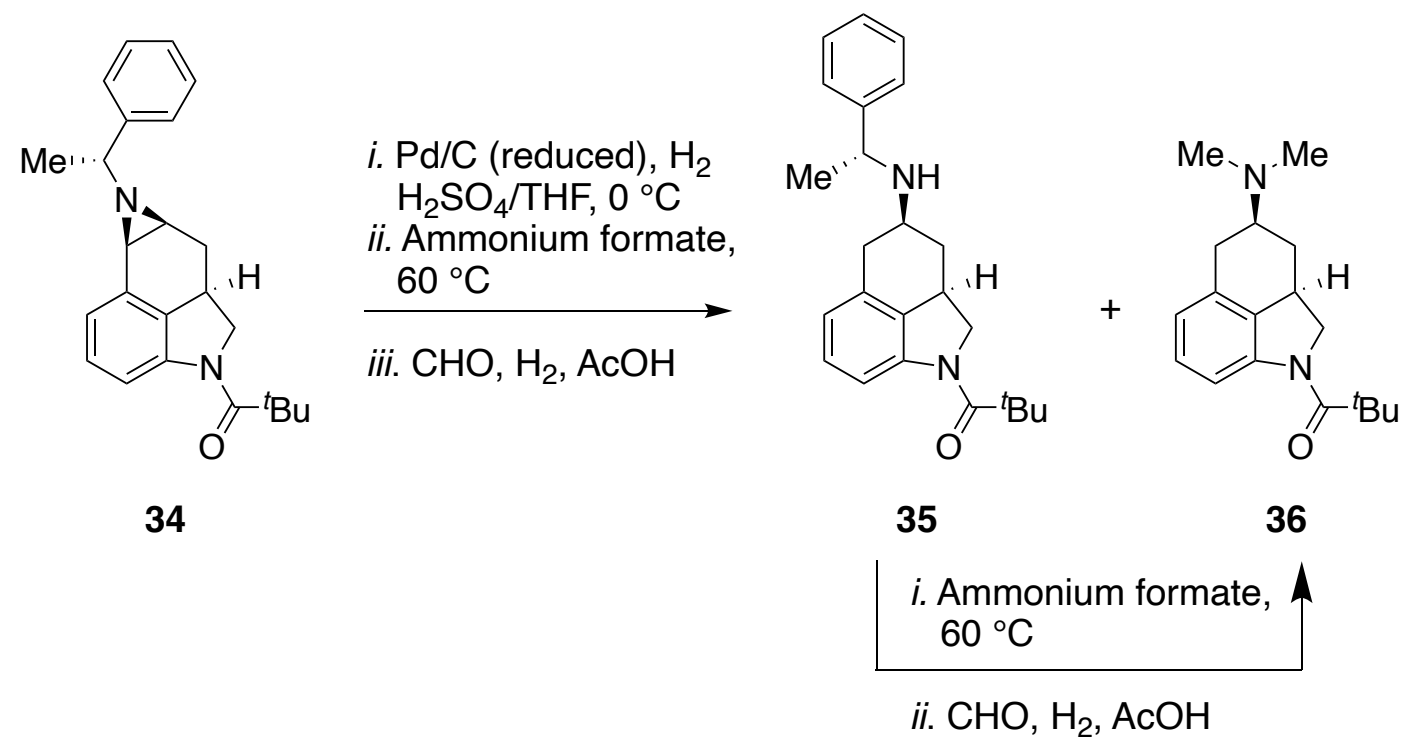

1-((2aR,4S)-4-(dimethylamino)-2a,3,4,5-tetrahydrobenzo[cd]indol-1(2H)-yl)-2,2-dimethylpropan-1-one (36)

To a 0 °C mixture of reduced 10 wt. % Pd/C catalyst (0.380 g, 0.357 mmol, 0.128 equiv.) and 1 M H_2SO_4 (2.78 mL) in THF (20.0 mL) under an argon atmosphere was added a solution of **34** (1.00 g, 2.78 mmol, 1.00 equiv.) dissolved in THF (8.00 mL). The mixture was sparged with hydrogen gas for 10 min and then stirred under a hydrogen atmosphere at 0 °C for 7.5 h. Upon addition of ammonia formate (1.80 g, 28.6 mmol, 10.3 equiv.) and EtOH (8.00 mL) the mixture was heated to 60 °C for 14 h. The mixture was then cooled to 0 °C and glacial acetic acid (1.50

mL, 26.2 mmol, 9.42 equiv.) and 37 wt. % formaldehyde (2.00 mL, 26.6 mmol, 9.57 equiv.) were added to the mixture. The mixture was sparged with hydrogen gas for 10 min and then stirred for 24 h under a hydrogen atmosphere. The solution was filtered, concentrated, and then basified to pH >10 with 1 M NaOH before being extracted with DCM (3 x 15 mL). The combined DCM extracts were dried over Na_2SO_4 and then concentrated under reduced pressure. The residue was purified by chromatography on silica gel (gradient elution: 75:25 hexanes/EtOAc to 50:50 hexanes/EtOAc, then 90:10:1 DCM/MeOH/NH4$OH_{(aq)}$) to afford **36** (468 mg, 59%) as a yellow solid over two cycles.

Note: Intermediate **35** (isolated as 35% initially) was then subjected to a second cycle of refluxing with ammonium formate and a subsequent reductive amination condition with formaldehyde, $H_{2(g)}$, and glacial acetic acid to give **36**. Each step was monitored by LCMS and allowed to run to completion before proceeding to the next step.

R_f = 0.40 (DCM/MeOH/NH4$OH_{(aq)}$, 90:10:1 v/v/v)
^{1}H NMR (600 MHz, CDCl3) δ (ppm) = 7.85 (d, J = 7.9 Hz, 1H), 7.13 (t, J = 7.9 Hz, 1H), 6.84 (d, J = 7.6 Hz, 1H), 4.58 (t, J = 8.9 Hz, 1H), 3.59 (t, J = 10.5 Hz, 1H), 3.36 – 3.28 (m, 1H), 3.03 – 2.96 (m, 2H), 2.75 – 2.66 (m, 1H), 2.40 (s, 6H), 2.34 (d, J = 11.7 Hz, 1H), 1.39 (d, J = 11.0 Hz, 1H), 1.37 (s, 9H)
^{13}C NMR (150 MHz, CDCl3) δ (ppm) = 176.30, 143.19, 132.41, 131.09, 128.20, 123.24, 115.51, 61.10, 57.59, 41.16, 39.80, 38.45, 28.92, 28.07, 27.63 ppm
LRMS (ES+) = *m/z* $[M]^+$ calcd. for $C_{18}H_{27}N_2O^+$ = 287.21; Found 287.35
IR (Smart iTX Diamond) = ν 2971, 2931, 2869, 2777, 1643, 1619, 1591 cm^{-1}

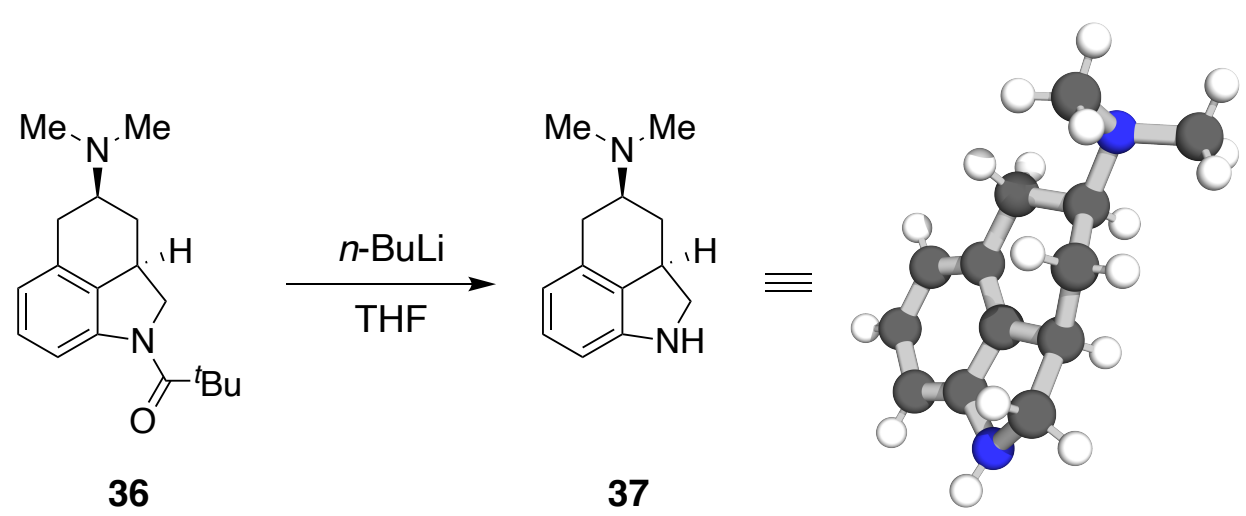

36 37

(2aR,4S)-N,N-dimethyl-1,2,2a,3,4,5-hexahydrobenzo[cd]indol-4-amine (37)
To a solution of **36** (154 mg, 0.538 mmol, 1.00 equiv.) in THF (3.60 mL) at -78 °C was added 2.5 M *n*-BuLi in hexanes (0.645 mL, 1.61 mmol, 3.00 equiv.). The mixture was stirred for 40 min at -78 °C before being quenched with saturated aqueous NH_4Cl (0.5 mL) and then basified to pH >10 with 1 M NaOH (1.5 mL). The solution was warmed to ambient temperature and then extracted with EtOAc (3 x 2 mL). The combined organic extracts were dried over Na_2SO_4, and then concentrated under reduced pressure. The residue was purified by chromatography on silica gel (gradient elution: 95:5:0.5 DCM/MeOH/NH4$OH_{(aq)}$ to 90:10:1 DCM/MeOH/$NH_4OH_{(aq)}$) to afford **37** (92.0 mg, 93%) as a yellow solid.

Note: Autoxidation of the indoline to the indole may occur during deprotection under an N_2 atmosphere. This issue can be avoided by running the reaction under an Ar atmosphere.

Recrystallization Method for XRD
Solid **37** was dissolved in THF, then the solution was allowed to slowly evaporate over 2 days.
R_f = 0.25 (DCM/MeOH/NH4$OH_{(aq)}$, 90:10:1 v/v/v)
^{1}H NMR (600 MHz, CDCl3) δ (ppm) = 6.97 (t, J = 7.6 Hz, 1H), 6.55 (d, J = 7.6 Hz, 1H), 6.47 (d, J = 7.6 Hz, 1H), 3.80 – 3.62 (m, 2H), 3.23 – 3.09 (m, 2H), 3.04 – 2.91 (m, 2H), 2.66 (q, J = 11.4, Hz, 1H), 2.38 (s, 6H), 2.28 (d, J = 11.9 Hz, 1H), 1.38 (q, J = 11.4 Hz, 1H)

^{13}C NMR (150 MHz, CDCl3) δ (ppm) = 150.27, 133.12, 130.44, 127.82, 118.09, 106.30, 61.72, 56.21, 41.27, 39.00, 29.31, 28.31 ppm
LRMS (ES+) = *m/z* $[M]^+$ calcd. for $C_{13}H_{19}N_2^+$ = 203.15; Found 203.34
IR (Smart iTX Diamond) = ν 3220, 3035, 2921, 2823, 2773, 1621, 1602 cm^{-1}

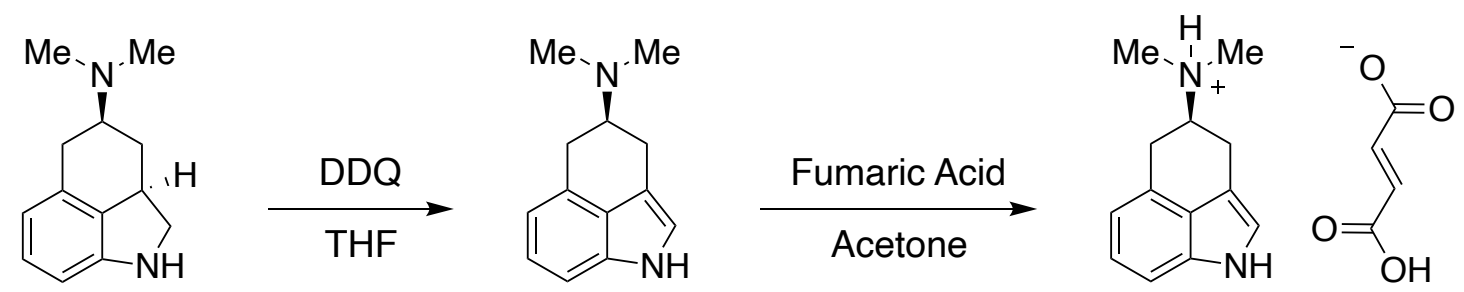

37 (-)-UCD0094 (-)-UCD0094 fumarate

(–)-UCD0094 fumarate
To a solution of **(37)** (96.0 mg, 0.475 mmol, 1.00 equiv.) in THF (8.00 mL) was added 2,3-dichloro-5,6-dicyano- 1,4-benzoquinone (107 mg, 0.475 mmol, 1.00 equiv.). The reaction was stirred for 2 h before being quenched by 1 M NaOH (1 mL). The mixture was extracted with Et_2O (3 x 5 mL). The combined organic extracts were dried over Na_2SO_4 and then concentrated under reduced pressure. The residue was purified by chromatography on silica gel (95:05:0.5 DCM/MeOH/$NH_4OH_{(aq)}$) to afford **(-)-UCD0094** (71.0 mg, 75%) as a red-brown oil. The corresponding salt was prepared according to **General Procedure A** using fumaric acid (37.0 mg, 0.319 mmol, 0.900 equiv.) and **(-)-UCD0094** (71.0 mg, 0.356 mmol, 1.00 equiv.) with acetone as the solvent. The product **(-)-UCD0094 fumarate** (50.1 mg, 44%) was obtained as an orange solid. Spectral data are in agreement with the racemate.

e.e. Analysis = Chiral-V column, 0.6 mL/min, sample run 10 min, 100:0.2 MeOH/ammonium formate (w/w)
e.r. = 98:2
e.e. = 96%
Optical Rotation $[\alpha]_D^{25}$ **=** -8.000 (c = 0.0025 M in EtOH)
Purity = >99% (Diode Array 254 nm)
Ratio of Amine to Acid = 1:1 (Ratio Determined by ^1H NMR)

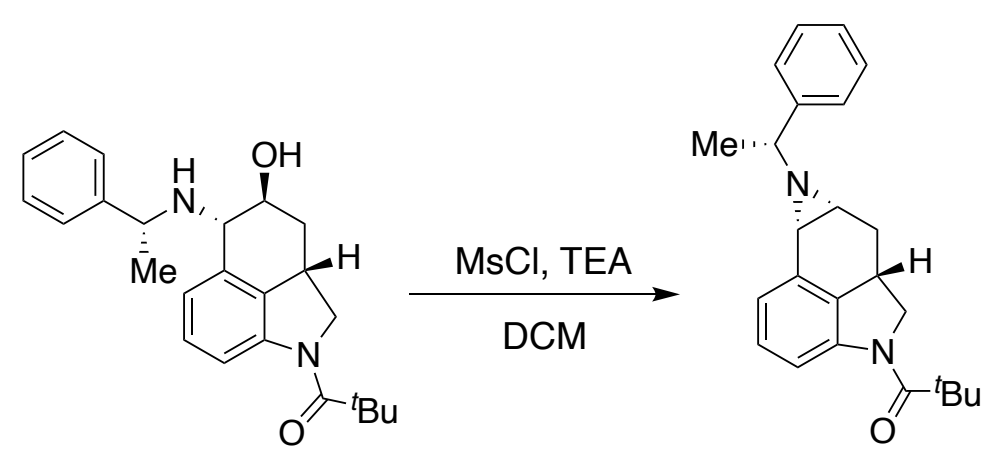

33 38

2,2-dimethyl-1-((5aS,6aR,7aS)-7-((R)-1-phenylethyl)-5,5a,6,6a,7,7a-hexahydro-4H azirino[2',3':4,5]benzo[1,2,3-cd]indol-4-yl)propan-1-one (38)
To a 0 °C cooled solution of **33** (4.18 g, 11.0 mmol, 1.00 equiv.) in DCM (120 mL) were added triethylamine (4.77 mL, 34.2 mmol, 3.10 equiv.) and methane sulfonyl chloride (1.28 mL, 16.6 mmol, 1.50 equiv.). The reaction was warmed to ambient temperature and stirred for 2 h before being quenched with H_2O (22 mL). The solution was extracted with DCM (3 x 25 mL). The combined organic extracts were dried over Na_2SO_4 and then concentrated under reduced pressure. The residue was purified by chromatography on silica gel (90:10 hexanes/EtOAc) then recrystallized in hexanes to afford **38** (3.69 g, 93%, > 95:5 d.r. **38**:**34**) as a white crystalline solid.

Note: Slight heating may be required to fully dissolve the starting material prior to the addition of triethylamine or methane sulfonyl chloride.

R_f = 0.45 (hexanes/EtOAc, 75:25 v/v)
^{1}H NMR (600 MHz, CDCl3) δ (ppm) = 7.98 (d, J = 7.7 Hz, 1H), 7.41 (d, J = 7.7 Hz, 2H), 7.33 (t, J = 7.7 Hz, 2H), 7.26 (t, J = 7.4 Hz, 1H), 7.23 (t, J = 7.9 Hz, 1H), 7.19 (d, J = 7.0 Hz, 1H), 4.52 (t, J = 8.5 Hz, 1H), 3.50 – 3.45 (m, 1H), 3.45 – 3.37 (m, 1H), 2.76 (q, J = 6.5 Hz, 1H), 2.59 (dt, J = 13.4, 6.7 Hz, 1H), 2.50 (d, J = 6.3 Hz, 1H), 2.09 (td, J = 7.5, 3.5 Hz, 1H), 1.64 – 1.59 (m, 1H), 1.54 (d, J = 6.6 Hz, 3H), 1.35 (s, 9H)
^{13}C NMR (150 MHz, CDCl3) δ (ppm) = 176.33, 144.56, 143.42, 132.39, 131.97, 128.68, 128.32, 126.94, 126.78, 123.79, 117.38, 69.93, 57.49, 39.86, 38.76, 37.48, 35.40, 31.36, 27.64, 23.67 ppm

LRMS (ES+) = *m/z* $[M]^+$ calcd. for $C_{24}H_{29}N_2O^+$ = 361.23; Found 361.36
IR (Smart iTX Diamond) = ν 2969, 2925, 2871, 1645, 1619, 1591 cm^{-1}

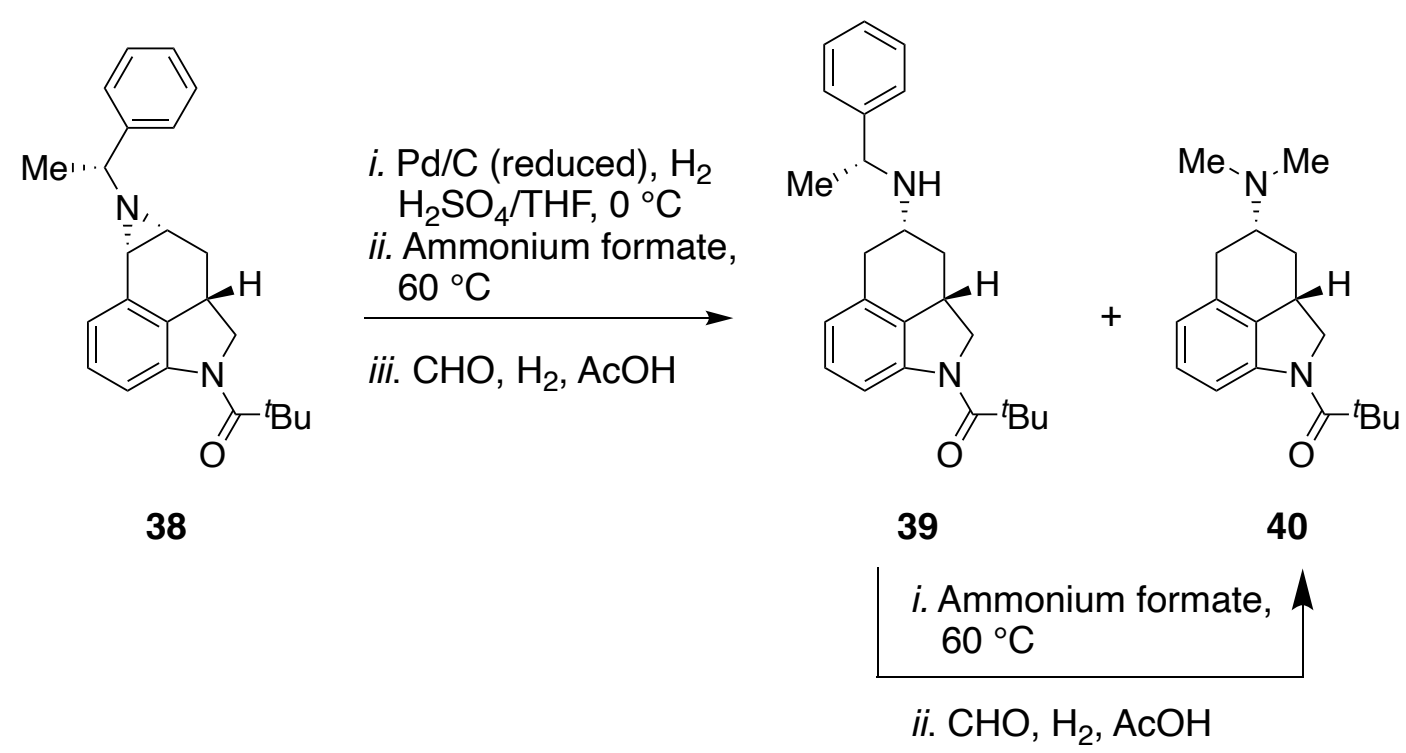

1-((2aS,4R)-4-(dimethylamino)-2a,3,4,5-tetrahydrobenzo[cd]indol-1(2H)-yl)-2,2-dimethylpropan-1-one (40)

To a 0 °C solution of reduced 10 wt. % Pd/C catalyst (0.380 g, 0.357 mmol, 0.128 equiv.) in THF (20.0 mL) and 1 M H2SO4 (2.78 mL) under an argon was added a solution **38** (1.00 g, 2.78 mmol, 1.00 equiv.) dissolved in THF (8.00 mL). The mixture was sparged with hydrogen gas for 10 min and then stirred under a hydrogen atmosphere at 0 °C for 7.5 h. Then ammonia formate (1.80 g, 28.6 mmol, 10.3 equiv.) and EtOH (8.00 mL) were added to the mixture and the reaction was heated to 60 °C for 14 h. After the mixture was cooled to 0 °C, glacial acetic acid (1.50 mL, 26.2 mmol, 9.42 equiv.) and 37 wt. % formaldehyde (2.00 mL, 26.6 mmol, 9.57 equiv.) were added to the mixture. The mixture was sparged with hydrogen gas for 10 min and then stirred for 24 h under hydrogen atmosphere. The solution was filtered, concentrated, and then basified to pH >10 with 1 M NaOH before being extracted with DCM (3 X 15 mL). The combined DCM extracts were dried over Na_2SO_4 and then concentrated under reduced pressure. The residue was purified by chromatography on silica gel (gradient elution: 75:25 hexanes/EtOAc to 50:50 hexanes/EtOAc then 90:10:1 DCM/MeOH/$NH_4OH_{(aq)}$) to afford **40** (469 mg, 60%) as a yellow solid over two cycles.

Note: Intermediate **39** (isolated as 35% initially) was then subjected to a second cycle of refluxing with ammonium formate and a subsequent reductive amination condition with formaldehyde, $H_{2\,(g)}$, and glacial acetic acid to give **40**. Each step was monitored by LCMS and allowed to run to completion before proceeding to the next step.

R_f = 0.40 (DCM/MeOH/$NH_4OH_{(aq)}$, 90:10:1 v/v/v)
^{1}H NMR (400 MHz, CDCl3) δ (ppm) = 7.85 (d, J = 7.9 Hz, 1H), 7.12 (t, J = 7.9 Hz, 1H), 6.83 (d, J = 7.9 Hz, 1H), 4.57 (dd, J = 9.6, 8.0 Hz, 1H), 3.59 (dd, J = 11.9, 9.7 Hz, 1H), 3.34 – 3.25 (m,

1H), 3.03 – 2.94 (m, 2H), 2.78 – 2.64 (m, 1H), 2.39 (s, 6H), 2.35 (d, J = 8.2 Hz, 1H), 1.41 – 1.37 (m, 1H), 1.36 (s, 9H)
^{13}C NMR (100 MHz, CDCl3) δ (ppm) = 176.31, 143.22, 132.54, 131.16, 128.19, 123.27, 115.52, 61.12, 57.64, 41.24, 39.83, 38.50, 28.96, 28.20, 27.67
LRMS (ES+) = *m/z* $[M]^+$ calcd. for $C_{18}H_{27}N_2O^+$ = 287.21; Found 287.46
IR (Smart iTX Diamond) = ν 2966, 2921, 2869, 1639, 1619, 1591 cm^{-1}

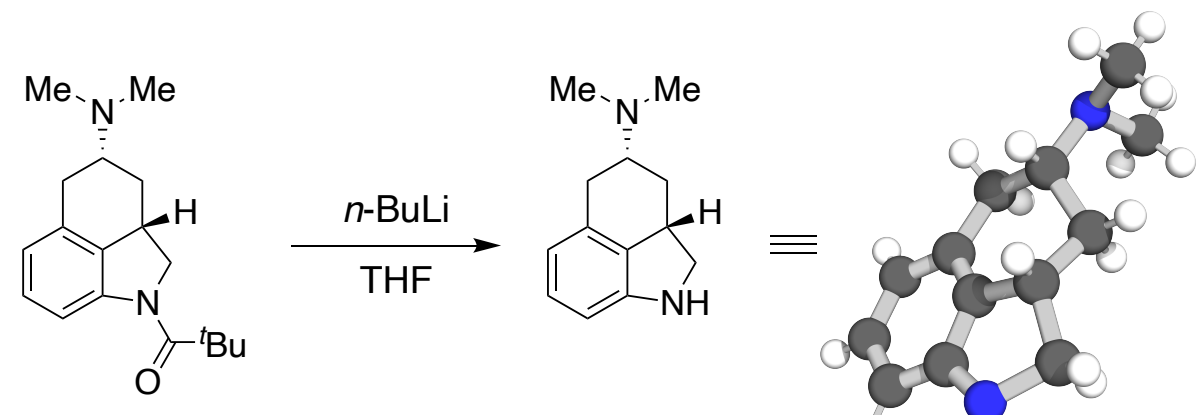

40 41

(2aS,4R)-N,N-dimethyl-1,2,2a,3,4,5-hexahydrobenzo[cd]indol-4-amine (41)
To a –78 °C solution of **40** (480 mg, 1.678 mmol, 1.0 equiv.) in THF (12.0 mL) was added 2.5 M n-BuLi in hexanes (2.0 mL, 5.03mmol, 3.0 equiv.). The mixture was stirred for 40 min at –78 °C before being quenched with saturated aqueous NH_4Cl (1.0 mL) and then basified to pH >10 with 1 M NaOH (5 mL). The solution was warmed to ambient temperature and then extracted with EtOAc (3 x 5 mL). The combined organic extracts were dried over Na_2SO_4, and then concentrated under reduced pressure. The residue was purified by chromatography on silica gel (100% EtOAc then 95:5:0.5 DCM/MeOH/$NH_4OH_{(aq)}$ to 90:10:1 DCM/MeOH/$NH_4OH_{(aq)}$) to afford **41** (250 mg, 77%) as a yellow solid.

Note: Autoxidation of the indoline to the indole may occur during deprotection under an N_2 atmosphere. This issue can be avoided by running the reaction under an Ar atmosphere.

Recrystallization Method for XRD
Solid **41** was dissolved in EtOAc, then the solution was allowed to slowly evaporate over 2 days.

R_f = 0.25 (DCM/MeOH/$NH_4OH_{(aq)}$, 90:10:1 v/v/v)
^{1}H NMR (600 MHz, CDCl3) δ (ppm) = 6.97 (t, J = 7.6 Hz, 1H), 6.55 (d, J = 7.6 Hz, 1H), 6.48 (d, J = 7.6 Hz, 1H), 3.80 – 3.60 (m, 2H), 3.25 – 3.10 (m, 2H), 3.03 – 2.91 (m, 2H), 2.66 (q, J = 11.1, 1H), 2.38 (s,6H), 2.28 d, J = 11.1 Hz, 1H), 1.38 (q, J = 11.1 Hz, 1H)
^{13}C NMR (150 MHz, CDCl3) δ (ppm) = 150.25, 133.13, 130.43, 127.82, 118.11, 106.31, 61.72, 56.21, 41.26, 38.99, 29.29, 28.29
LRMS (ES+) = *m/z* $[M]^+$ calcd. for $C_{13}H_{19}N^+$ = 203.15; Found 203.34
IR (Smart iTX Diamond) = ν 3241, 3043, 2925, 2856, 2821, 2773, 1621, 1602 cm^{-1}

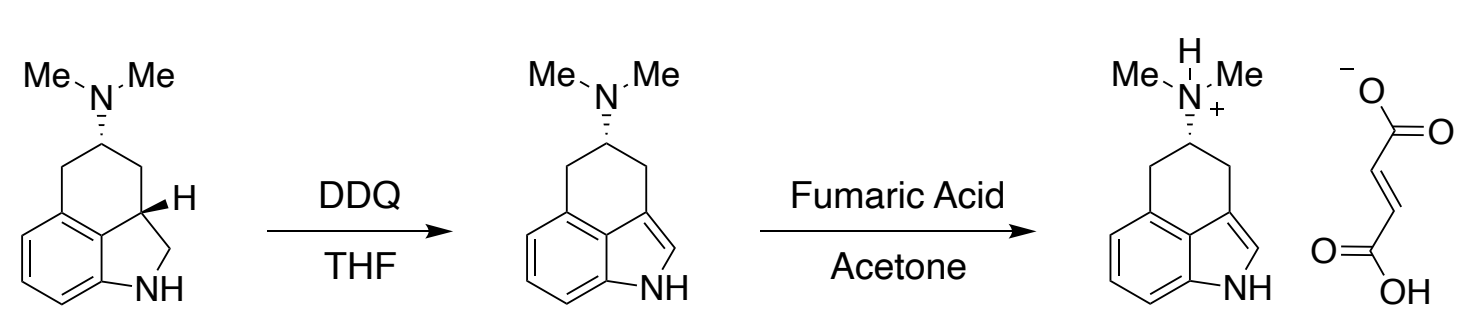

41 (+)-UCD0094 (+)-UCD0094 fumarate

(+)-(S)-N,N-dimethyl-1,3,4,5-tetrahydrobenzo[cd]indol-4-aminium fumarate ((+)-UCD0094 fumarate)
To a solution of **41** (220 mg, 1.09 mmol, 1.00 equiv.) in THF (18.0 mL) was added 2,3-dichloro-5,6-dicyano- 1,4-benzoquinone (247 mg, 1.09 mmol, 1.00 equiv.). The reaction was stirred for 2

h before being quenched by 1 M NaOH (6.0 mL). The mixture was extracted with ether (3 x 10 mL). The combined organic extracts were dried over Na_2SO_4 and then concentrated under reduced pressure. The residue was purified by chromatography on silica gel (95:05:0.5 DCM/MeOH/$NH_4OH_{(aq)}$) to afford **(+)-UCD0094** (130 mg, 60%) as a green solid. The corresponding salt was prepared according to **General Procedure A** using fumaric acid (63.0 mg, 0.540 mmol, 0.900 equiv.) and **(+)-UCD0094** (129 mg, 0.600 mmol) with acetone as the solvent. The product **(+)-UCD0094 fumarate** (130 mg, 63%) was obtained as a yellow solid. Spectral data are in agreement with the racemate.

e.e. Analysis = Chiral-V column, 0.6 mL/min, sample run 10 min, 100:0.2 MeOH/ammonium formate (w/w)
e.r. = >99:1
e.e. = >99%
Optical Rotation = $[\alpha]_D^{25}$= +8.000 (c = 0.0025 M in EtOH)
Purity = >99% (Diode Array, 254 nm)
Ratio of Amine to Acid = 1:1 (Ratio Determined by ^{1}H NMR)

4. Chiral LC-MS Traces

Enantiomeric Excess = Enantiomer Excess (ee) was determined by chiral Waters ultra-high-performance liquid chromatography (UHPLC). This method utilized an InfinityLab Poroshell 120 Chiral-V 4.6 X 100 mm, 2.7 μm column, with an injection volume of 1 μL, a flow rate of 0.6 mL/min maintained for 10 min at ambient temperature, using a solvent system of 100:0.2 MeOH/ammonium formate (w/w).

UCD0094 fumarate (racemate) (Diode Array, 254 nm)

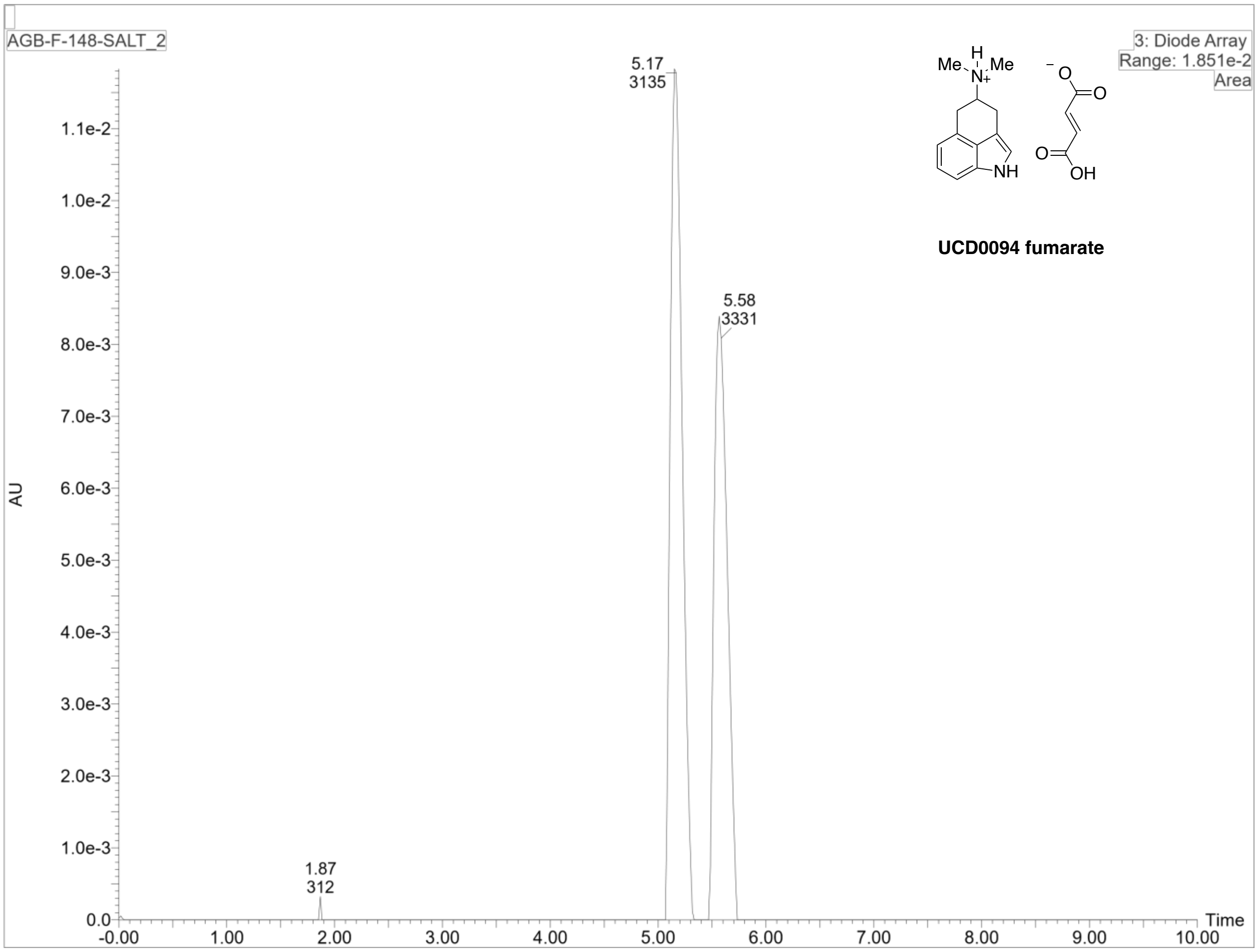

Peaks #	Ret. Time	Area	Height	Area %	Height %
1	5.17	3135	13551	48.5	53.8
2	5.58	3331	11626	51.5	46.2
Total	-	6466	25177	100	100

Note: For **UCD0094 fumarate**, the peak at 1.87 min corresponds to the fumarate.

(-)-UCD0094 (Diode Array, 254 nm)

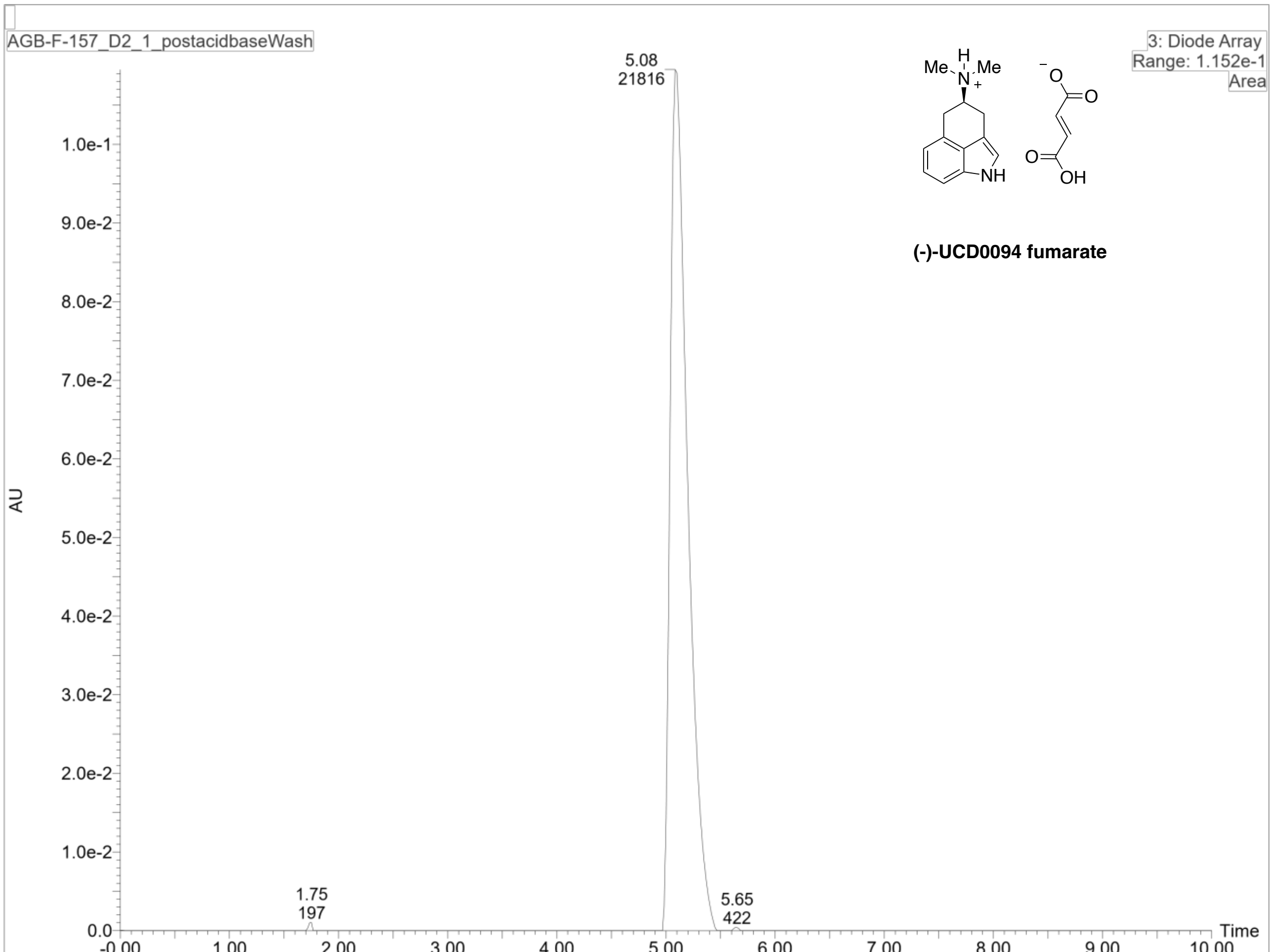

Peaks #	Ret. Time	Area	Height	Area %	Height %
1	5.08	21816	112986	98.1	98.1
2	5.65	422	2197	1.9	1.9
Total	-	22238	115183	100	100

Note: For **(-)-UCD0094 fumarate**, the peak at 1.75 min corresponds to the fumarate.

(+)-UCD0094 (Diode Array, 254 nm)

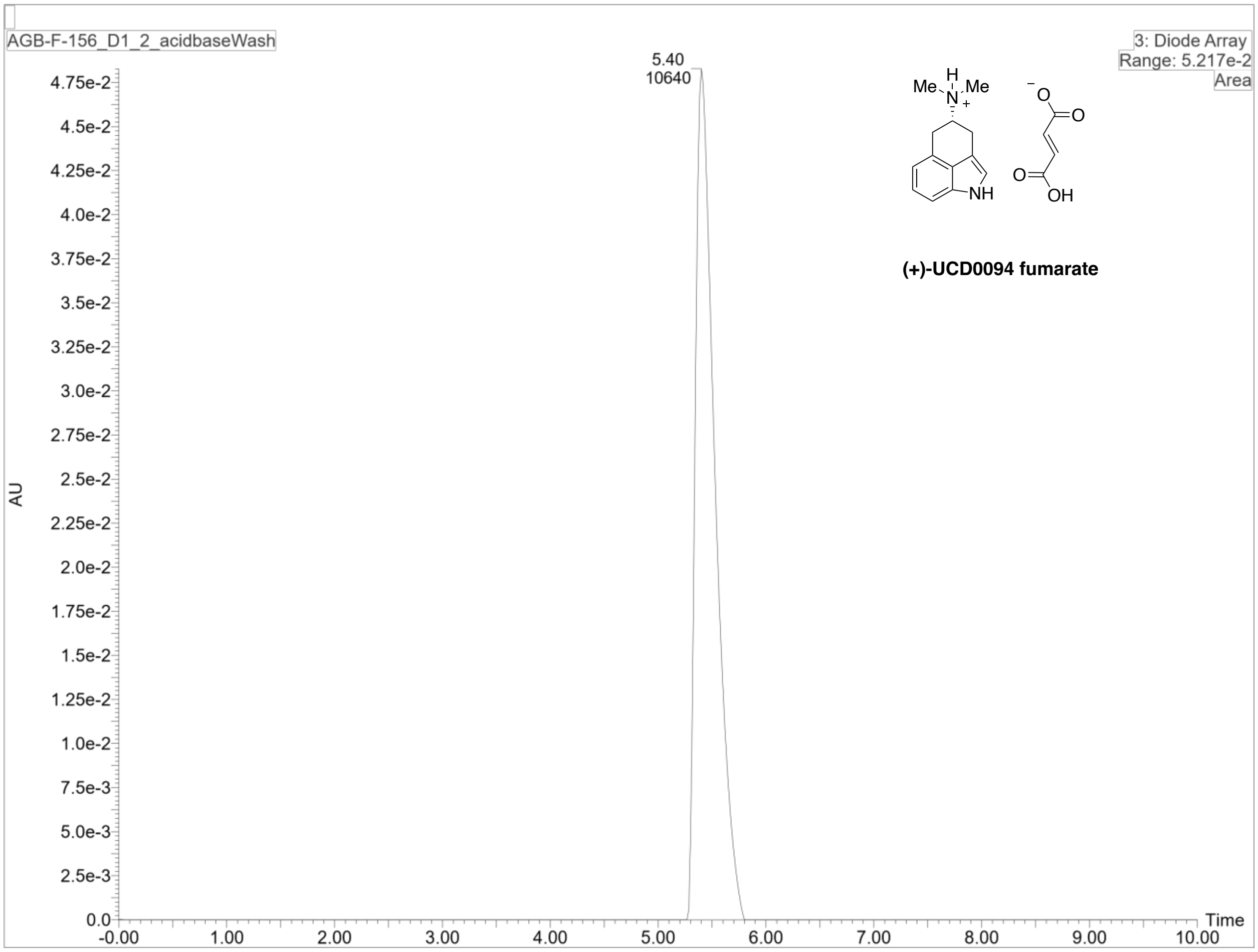

Peaks #	Ret. Time	Area	Height	Area %	Height %
1	-	-	-	-	-
2	5.40	10640	50178	>99	>99
Total	-	10640	46886	100	100

5. UHPLC-MS Purity Traces

UCD0094 fumarate (Diode Array, 210 nm)

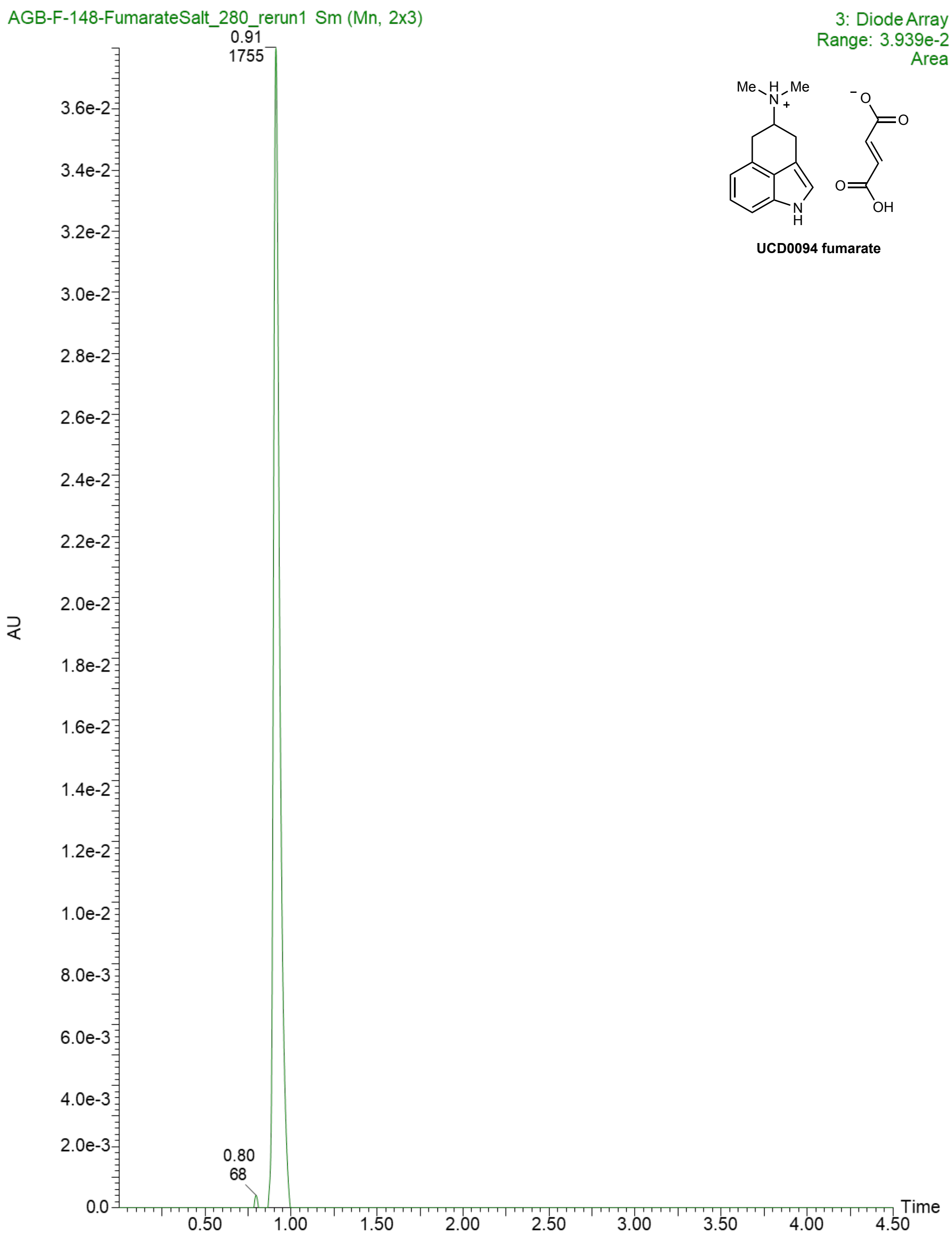

Note: For **UCD0094 fumarate**, the UV-active peak at 0.80 min corresponds to the fumarate.

UCD0179 tosylate (Diode Array, 210 nm)

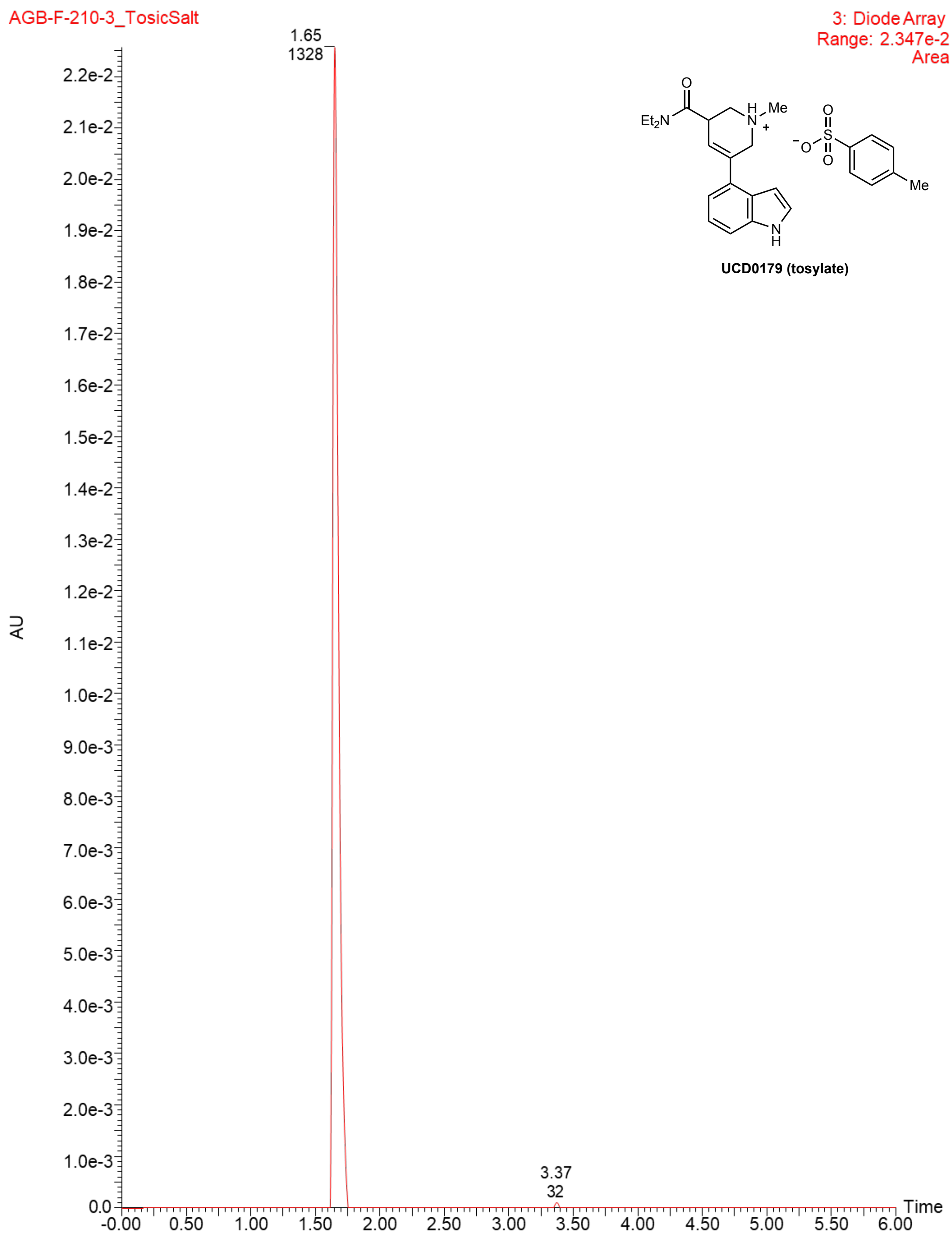

UCD0120 hydrochloride (Diode Array, 210 nm)

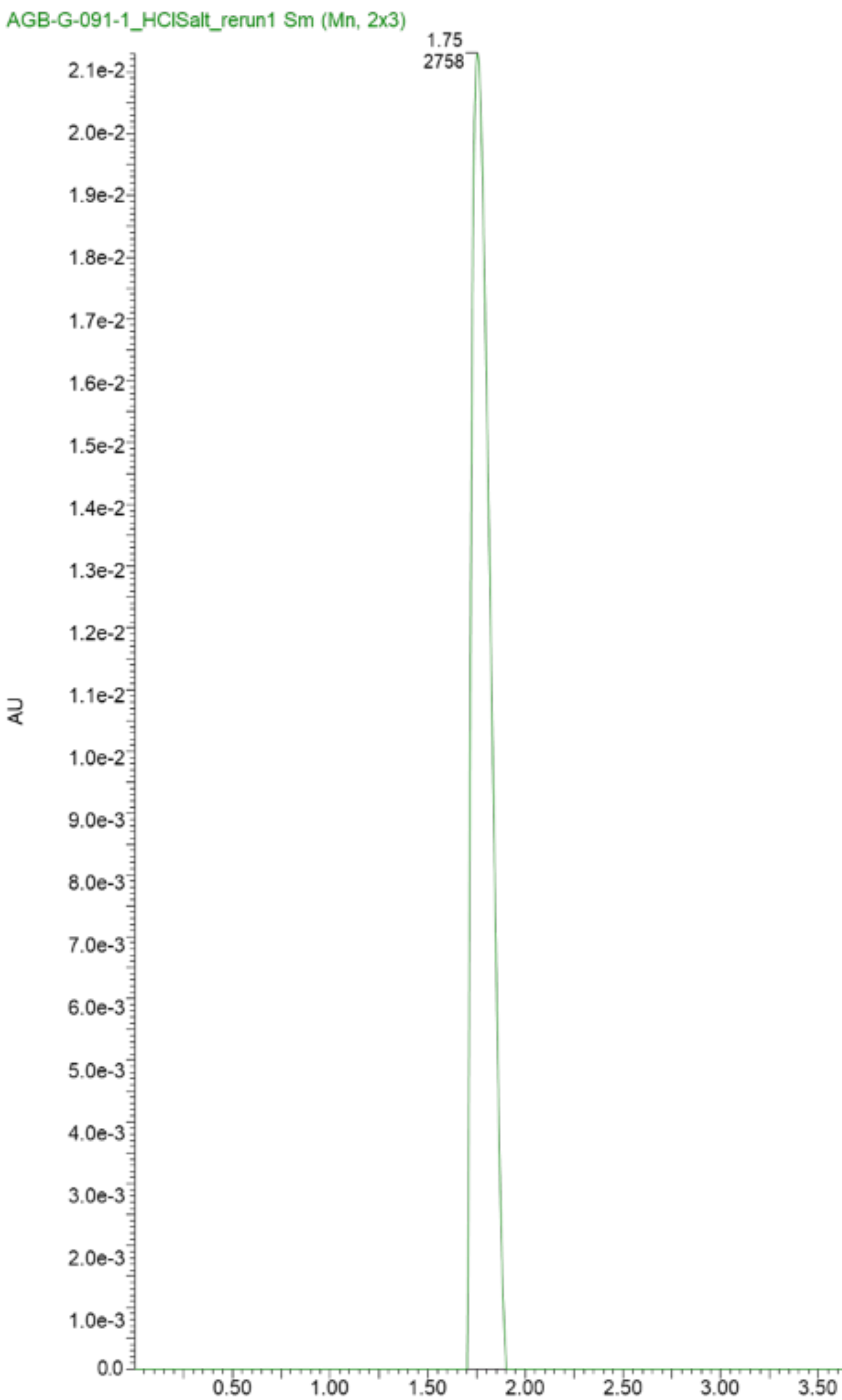

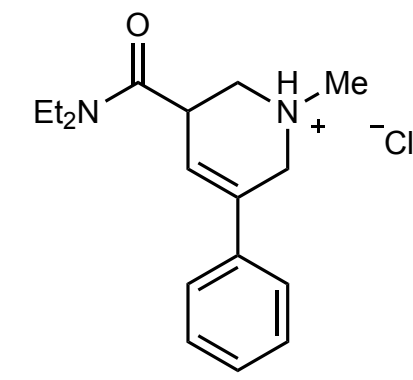

UCD0120 (hydrocloride)

UCD0121 tosylate (Diode Array, 210 nm)

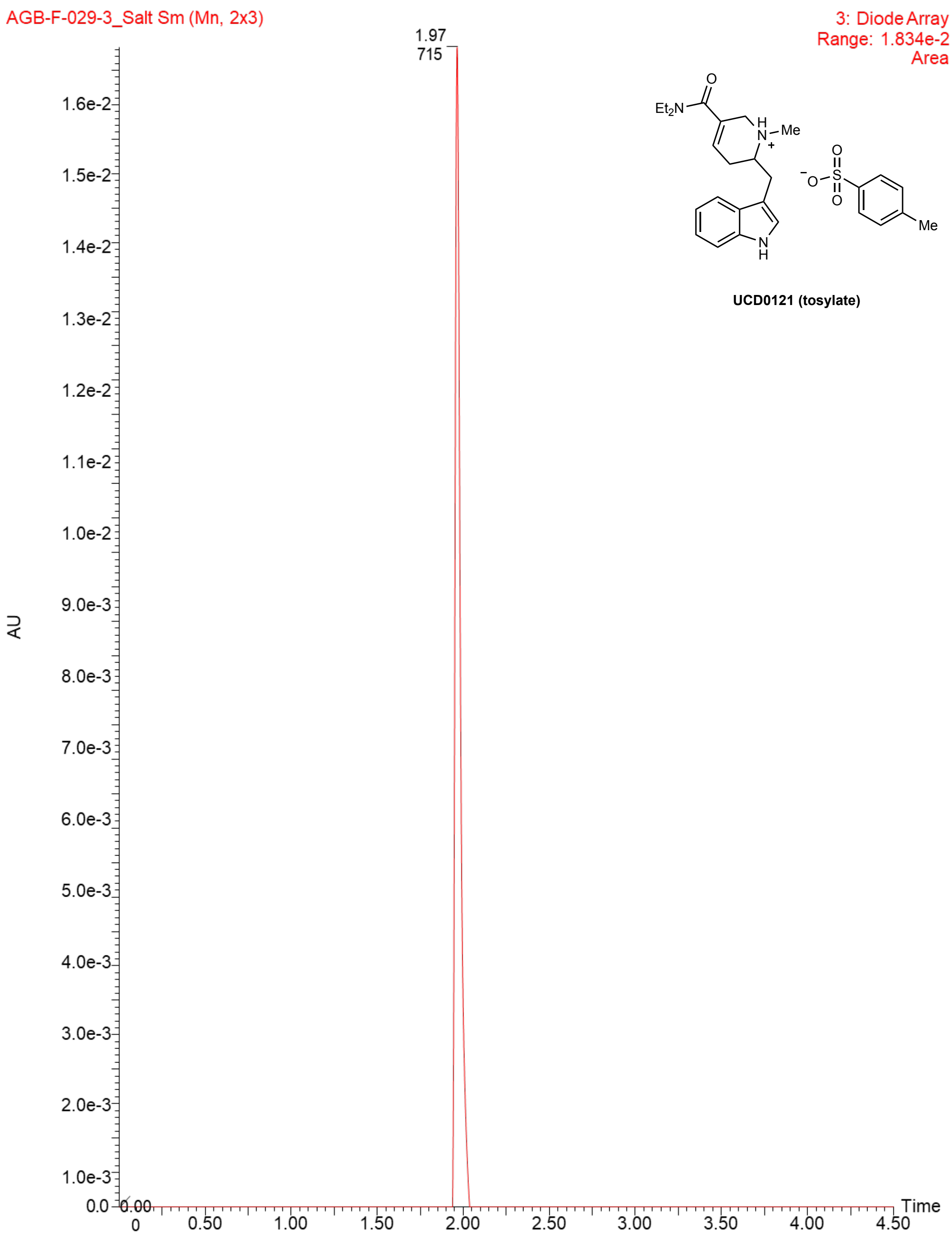

UCD0068 hydrochloride (Diode Array, 254 nm)

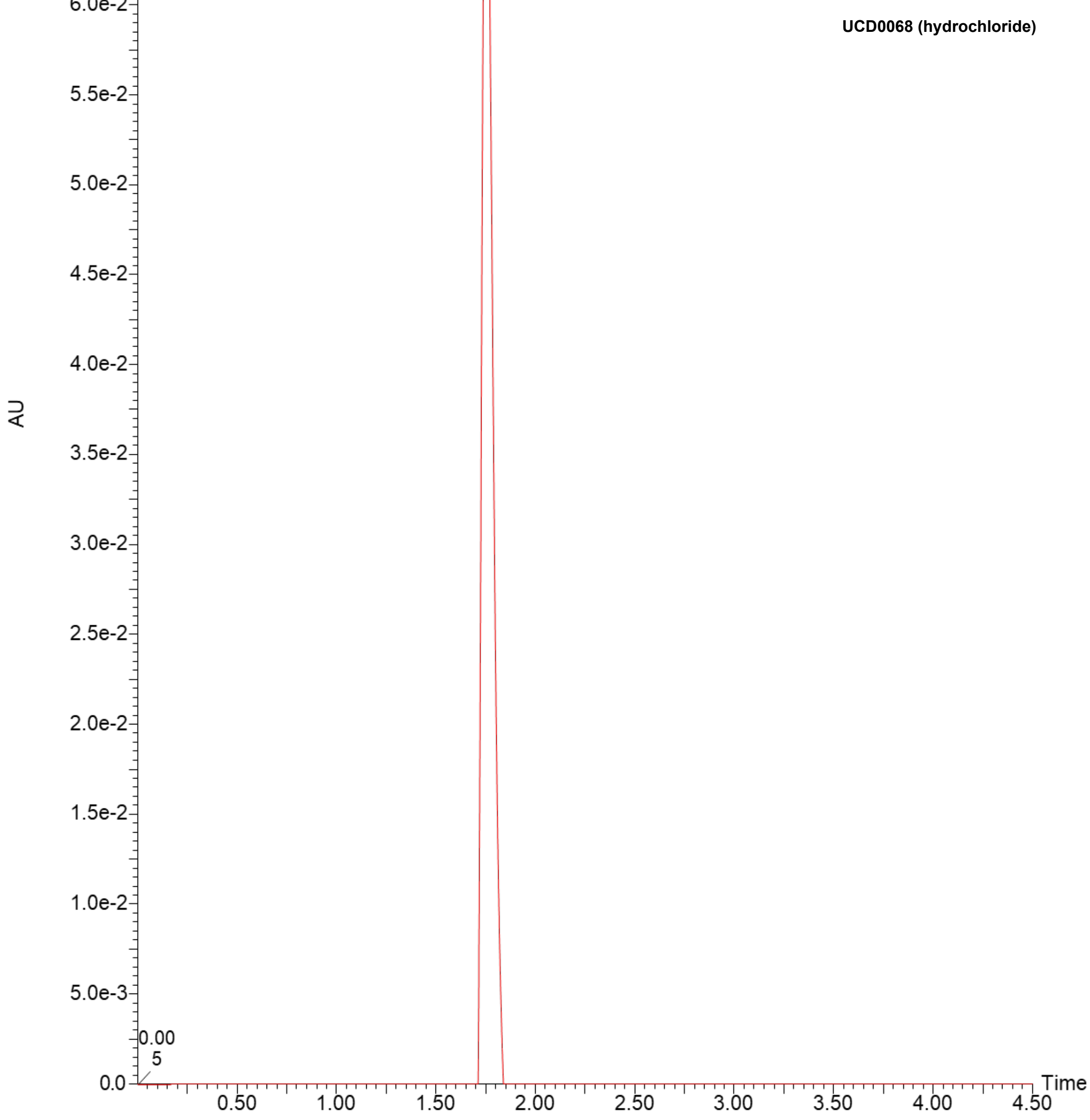

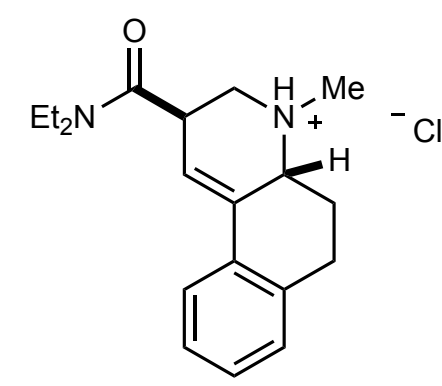

UCD0068 (hydrochloride)

UCD0076 hemifumarate (Diode Array, 210 nm)

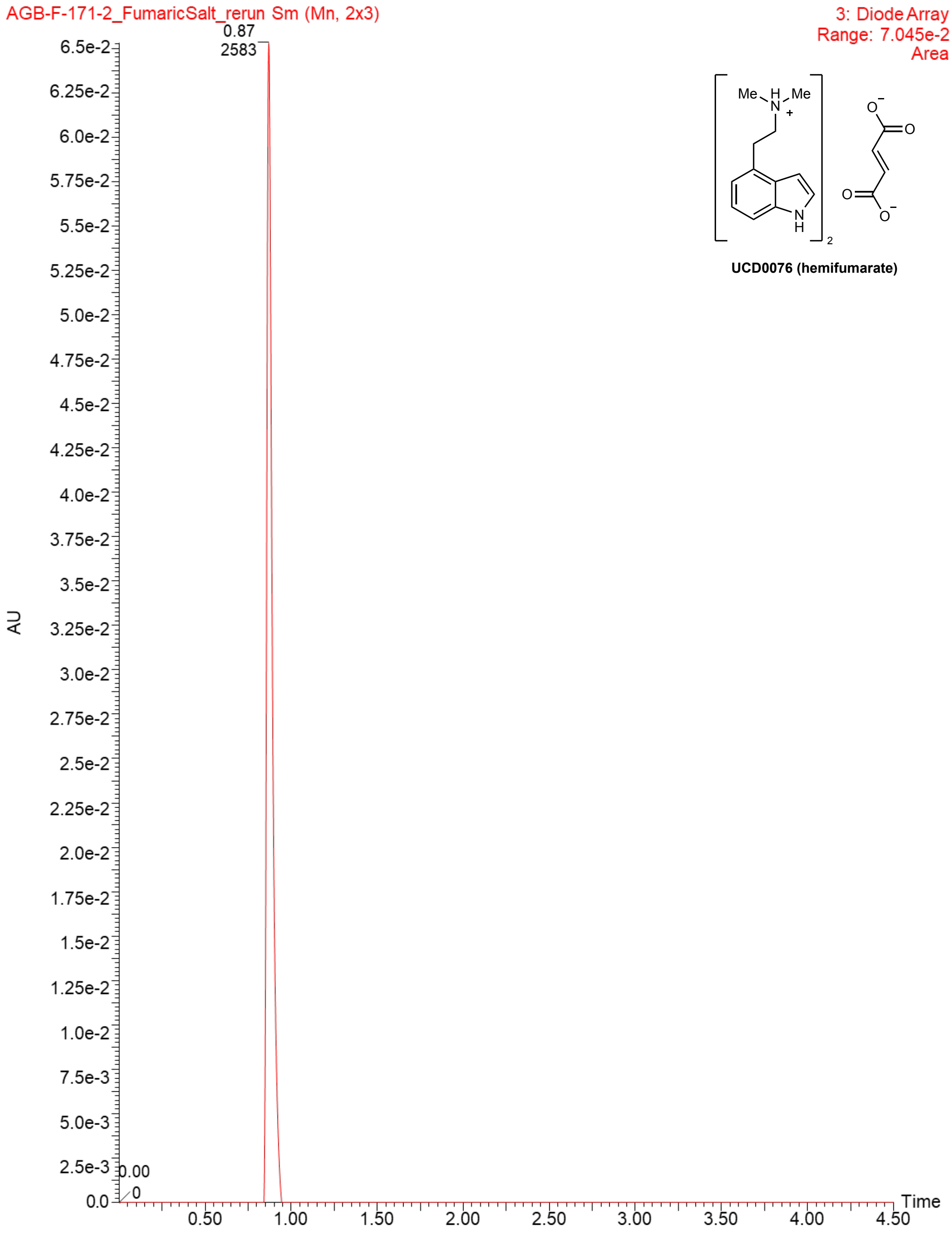

UCD0830 tosylate (ES+)

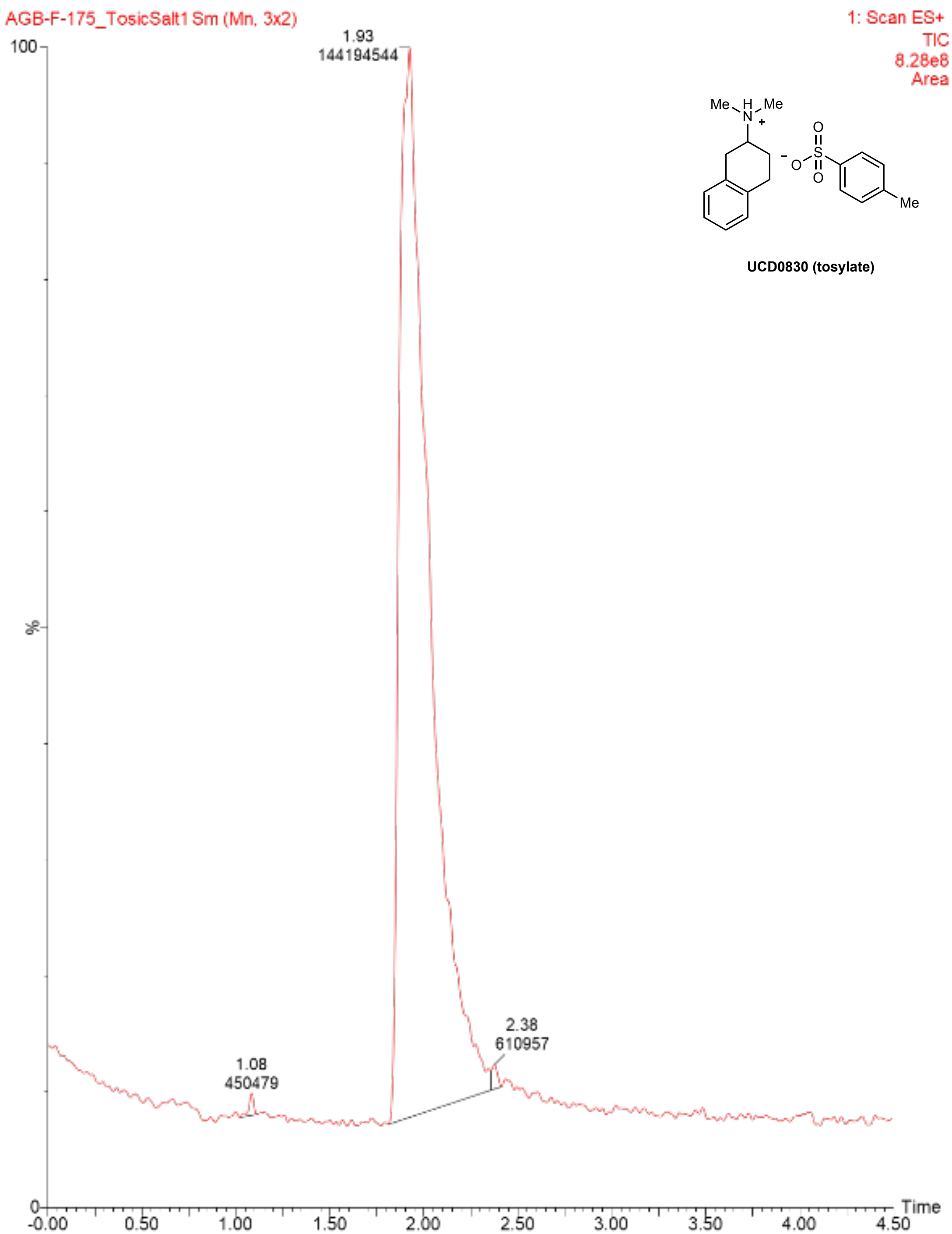

Note: UCD0830 tosylate was UV-inactive at 210, 225, 254, and 280 nm. MS spectra was utilized as the analytical method for purity analysis.

UCD0829 fumarate (ES +)

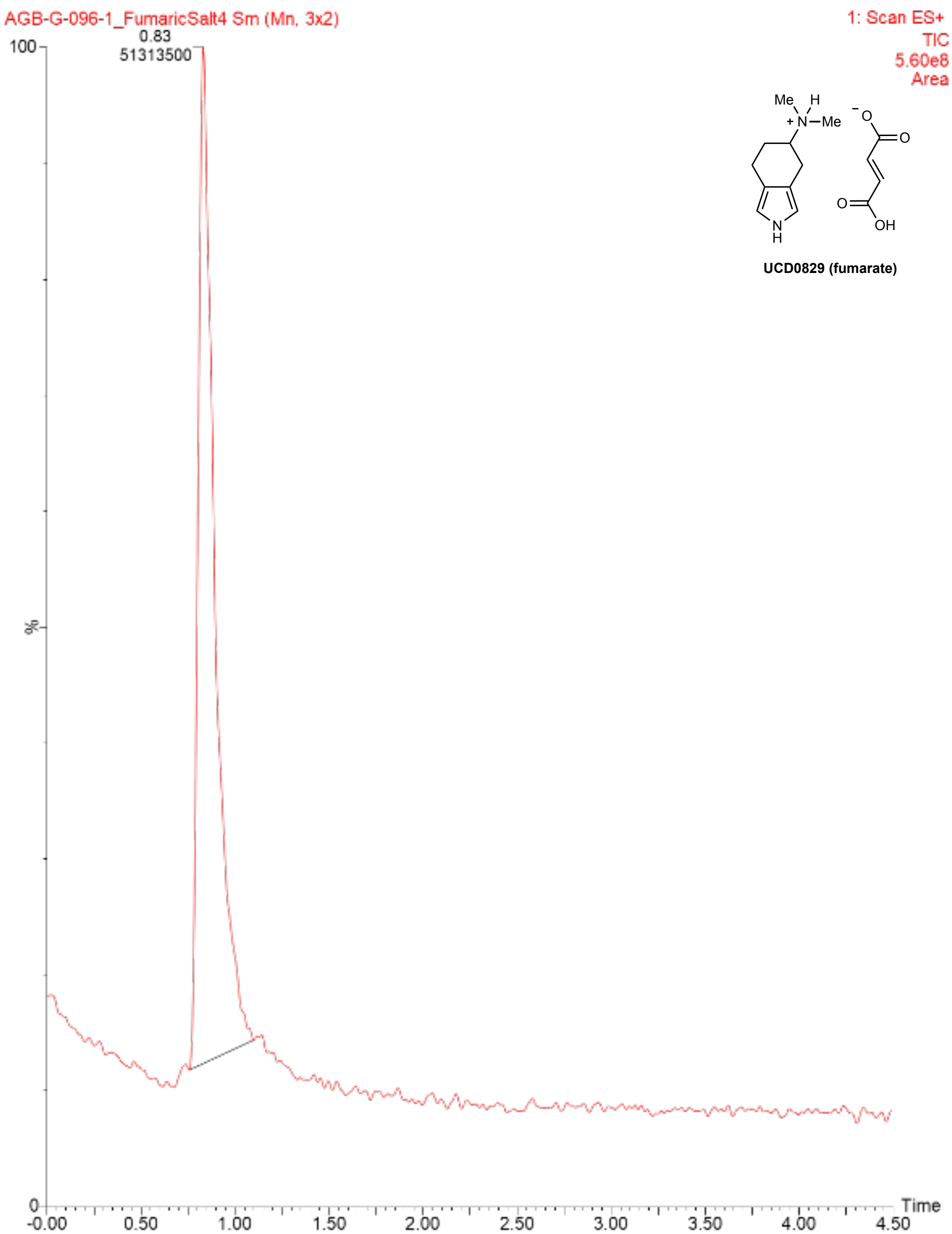

Note: UCD0829 fumarate was UV-inactive at 225, 254, and 280 nm. MS spectra was utilized as the analytical method for purity analysis.

UCD0162 hydrochloride (Diode Array, 210 nm)

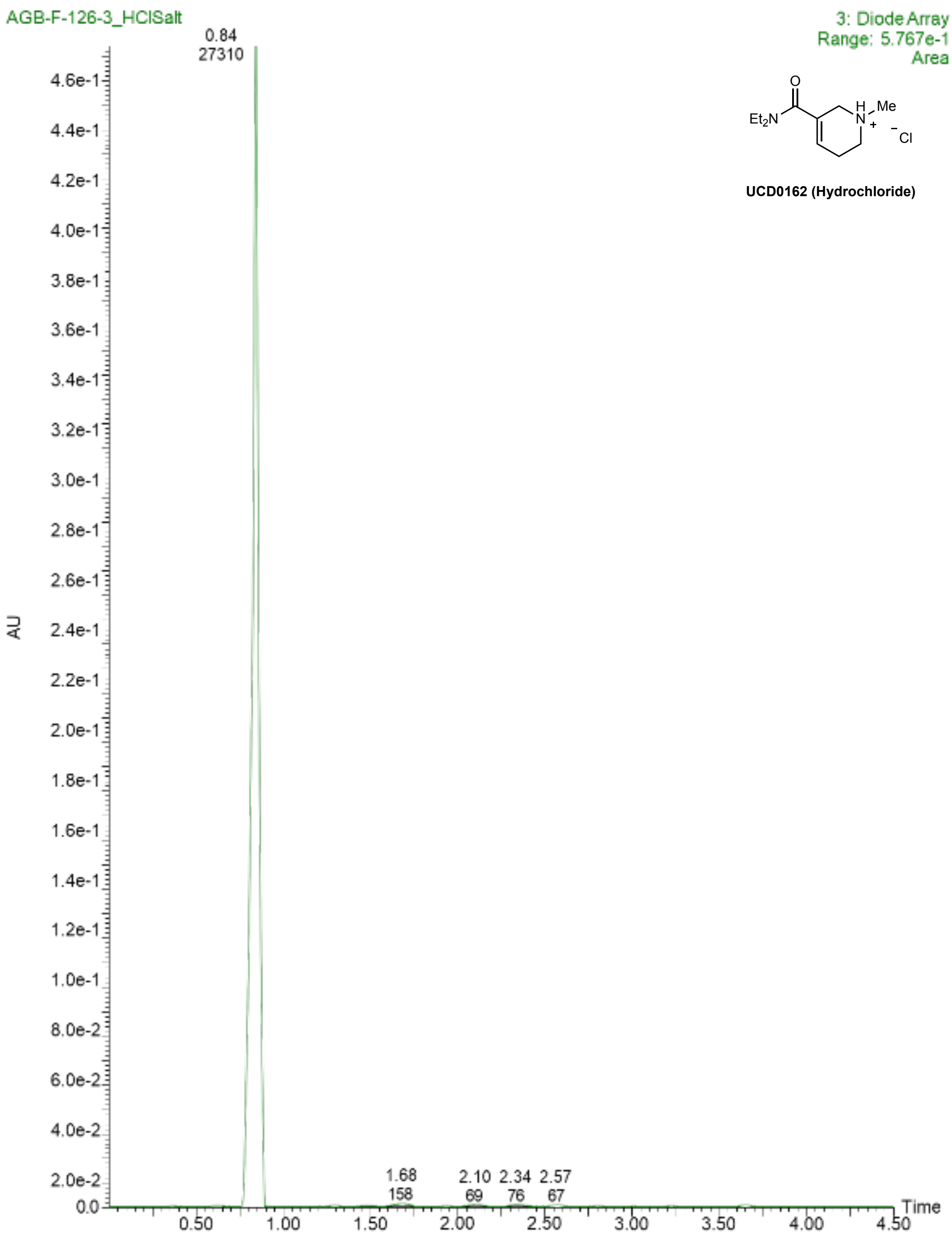

6. Additional characterization of UCD0068

DEPT-135

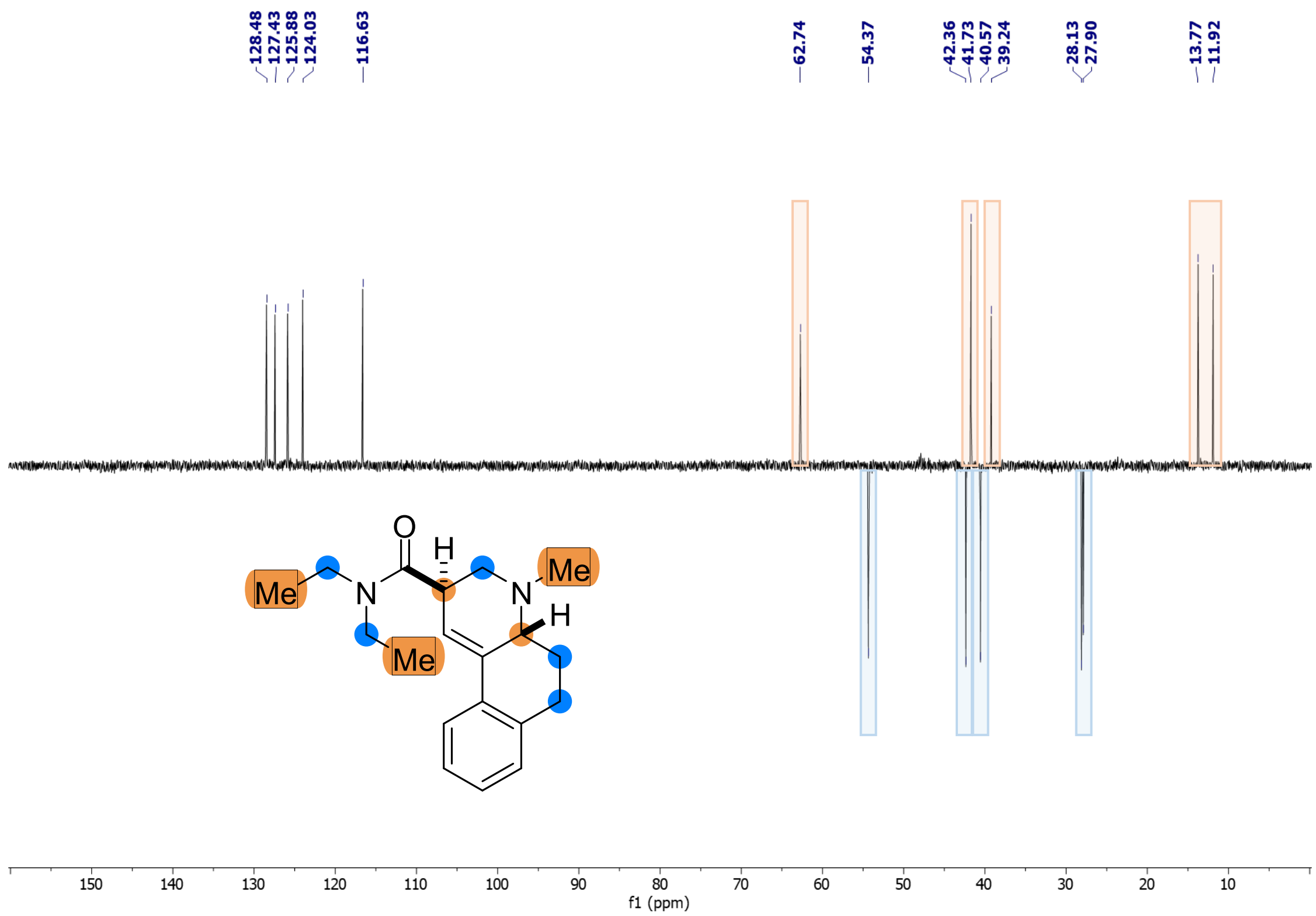

DEPT-135 identified CH_2 (blue), CH, (orange) and CH_3 (orange) carbons within the structure.

HSQC

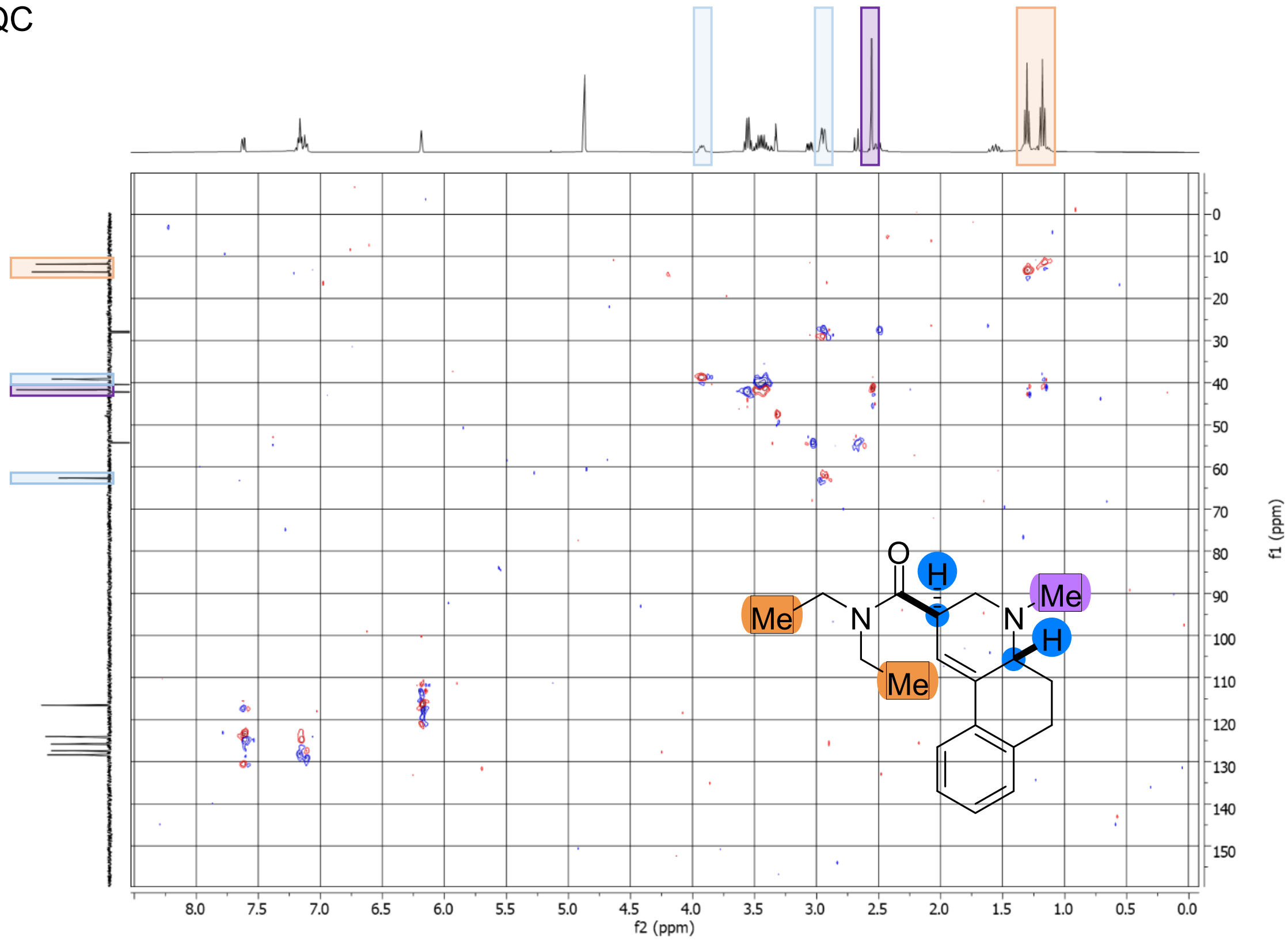

HSQC shows correlations between DEPT-135 and ^{1}H for methine and methylene signals.

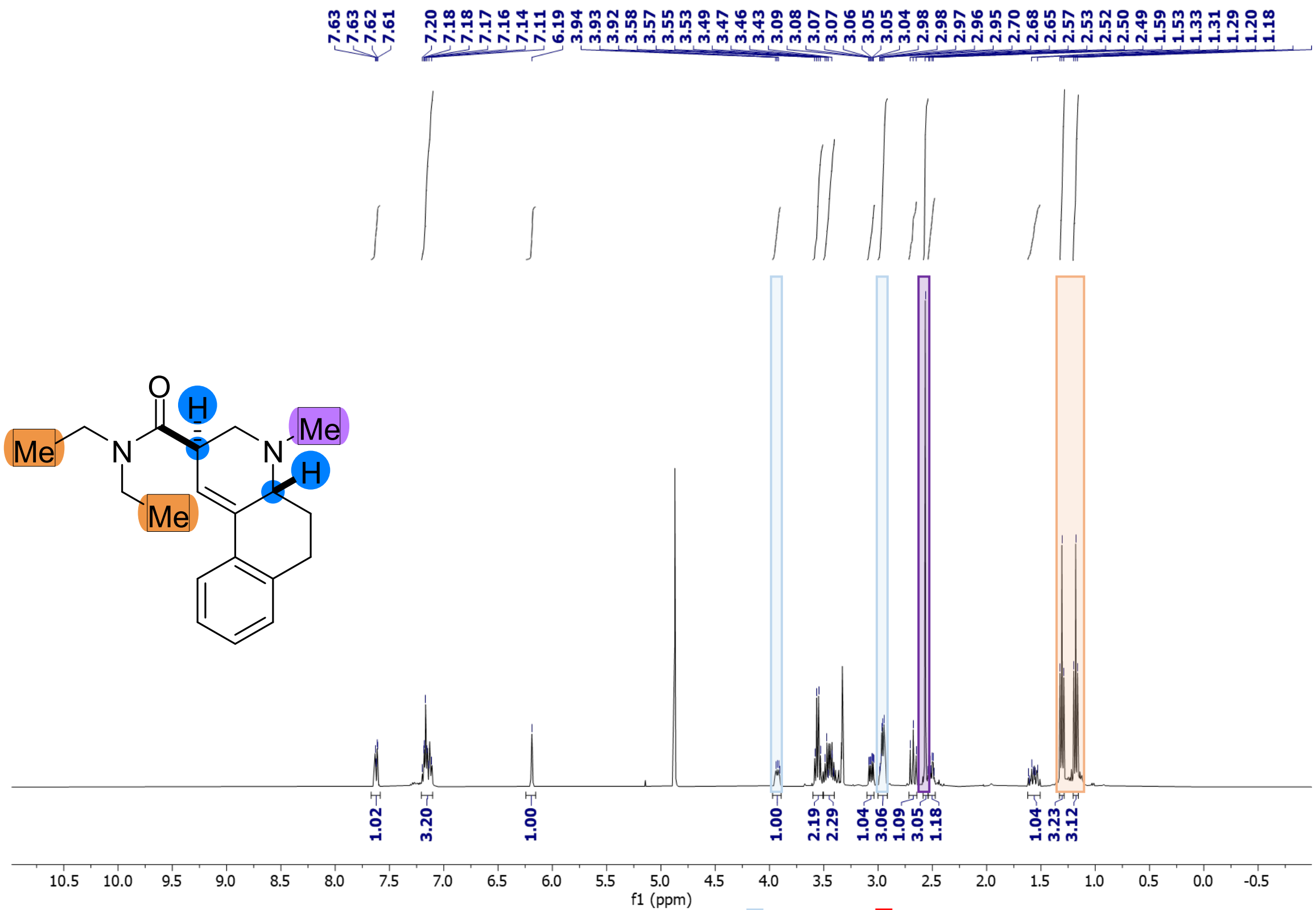

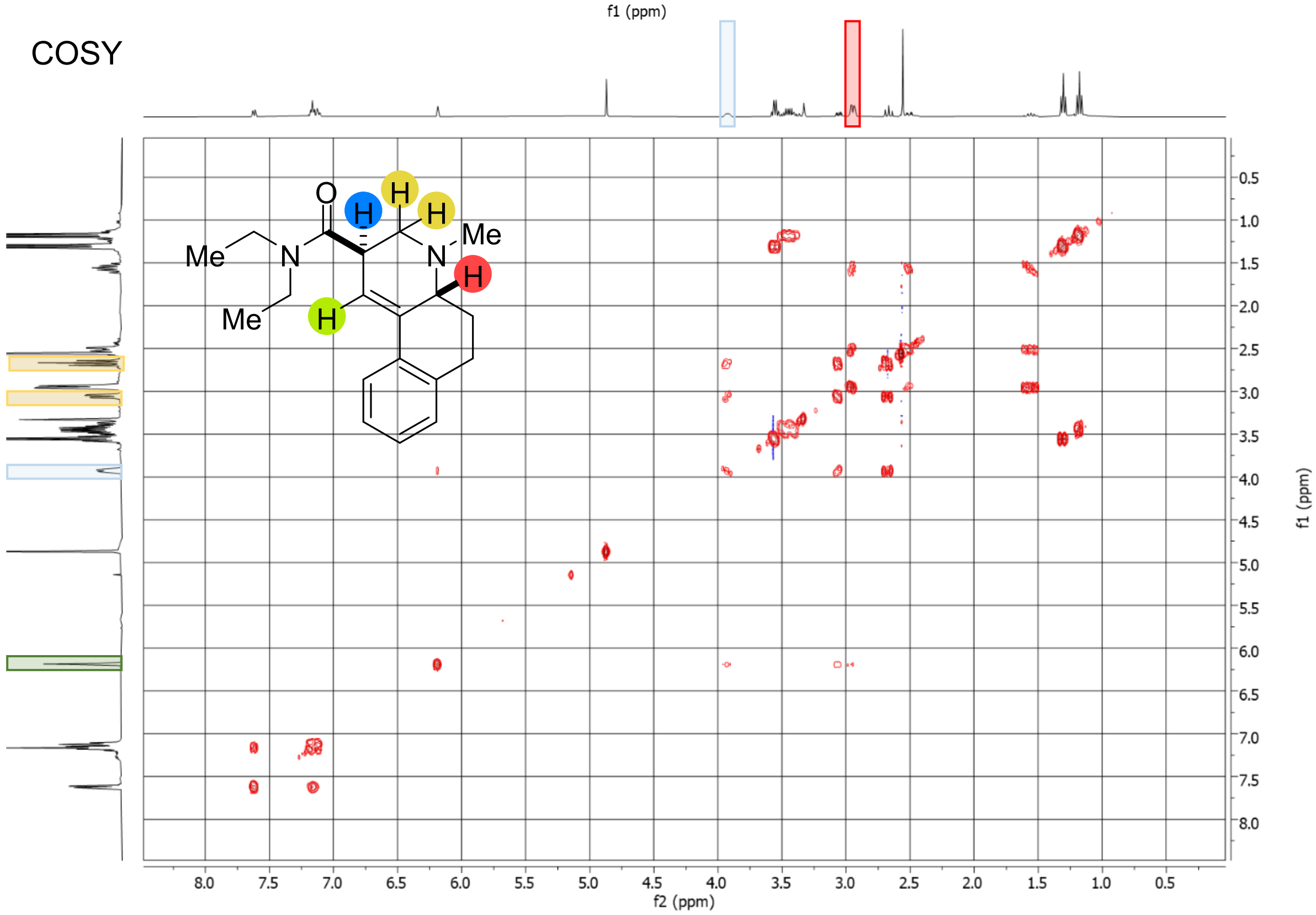

Olefin and methine correlations through homonuclear correlation spectroscopy (COSY).

2D NOE

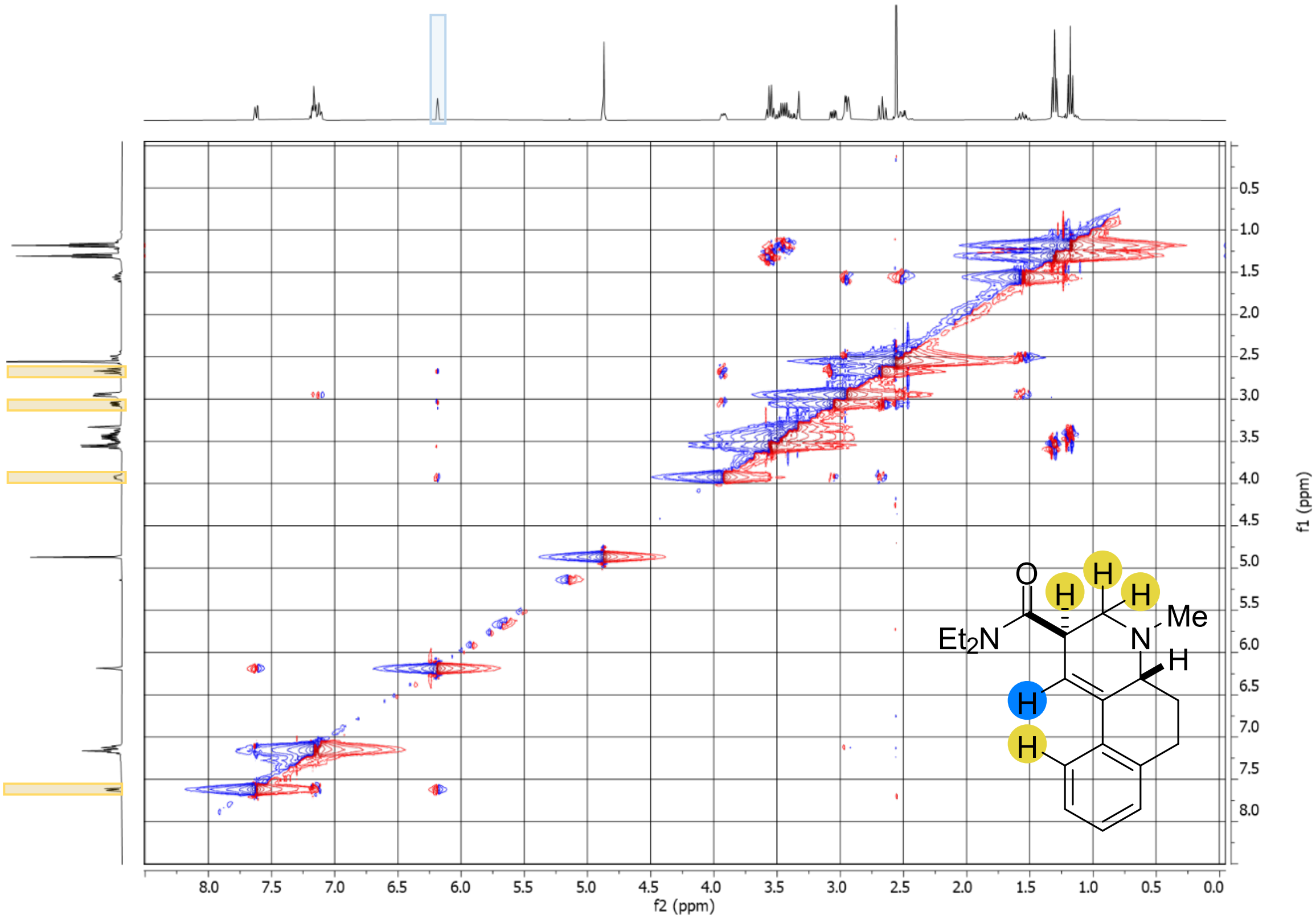

Observed correlations through 2D NOE shows correlations between olefin (blue) and neighboring protons.

1D NOE

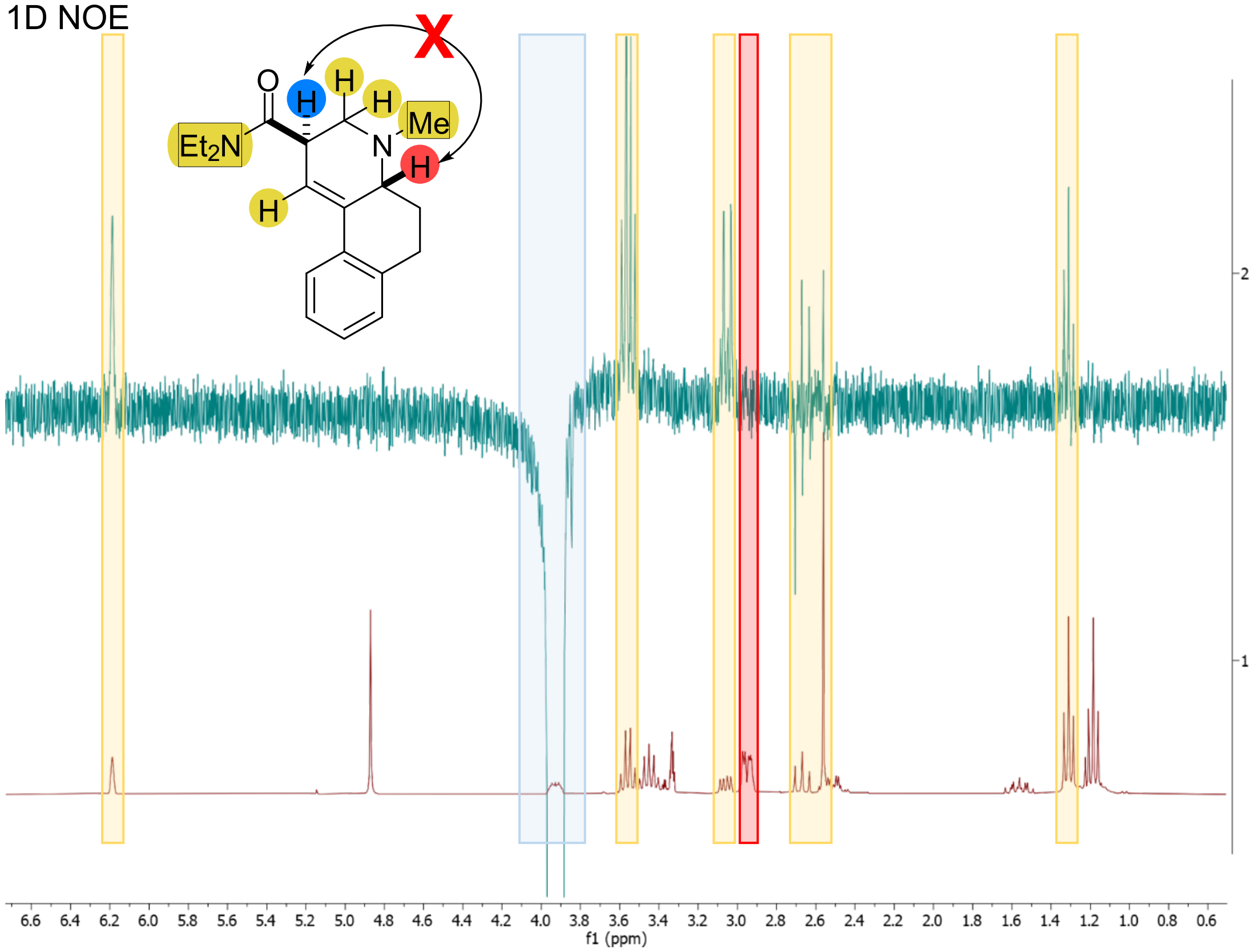

Observed correlations through 1D Selective NOESY shows correlations between methine (blue) and neighboring protons (yellow). No correlation is observed between the two methines.

7. ^{1}H and ^{13}C NMR Spectra

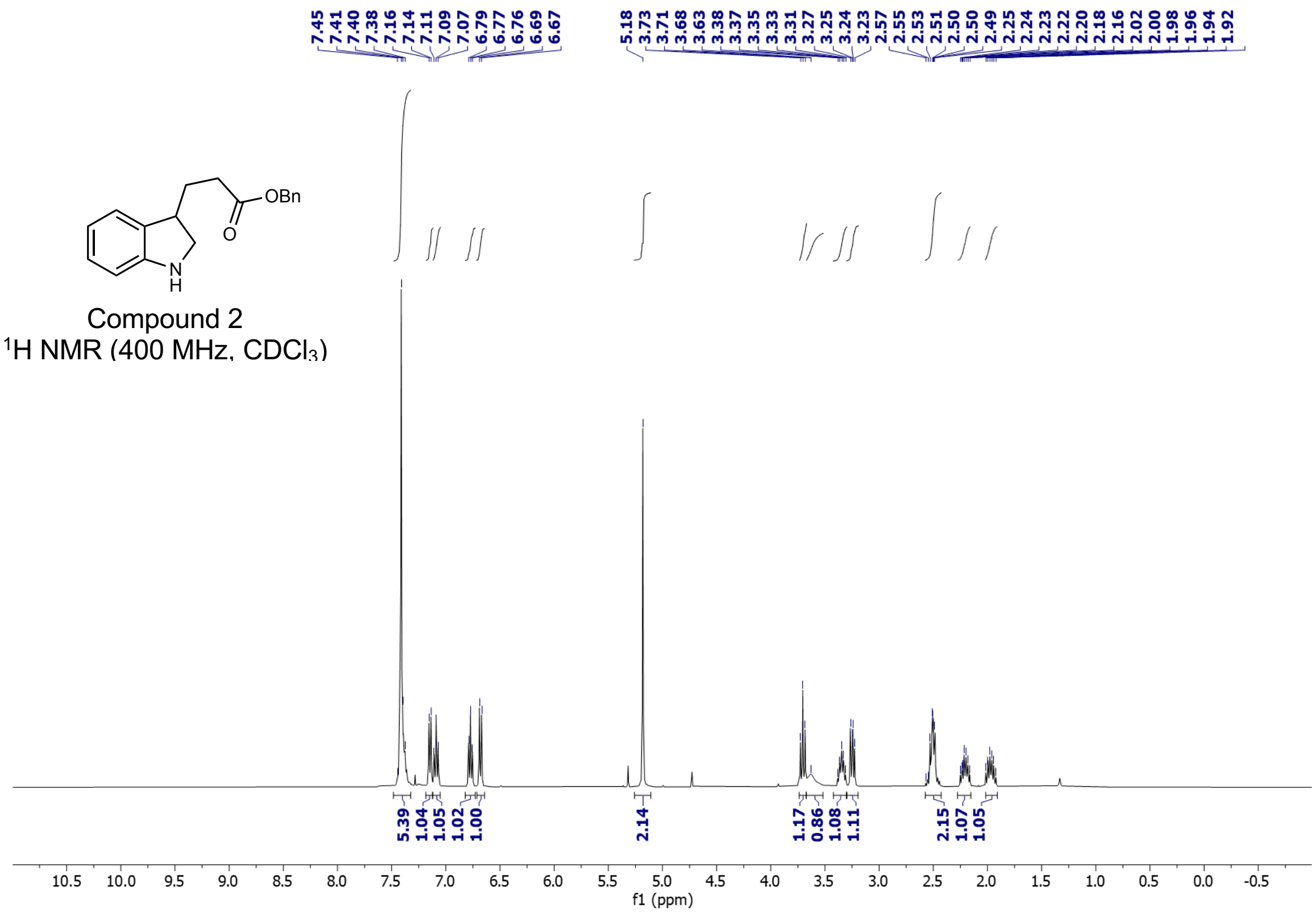

Compound 2
^{1}H NMR (400 MHz, $CDCl_3$)

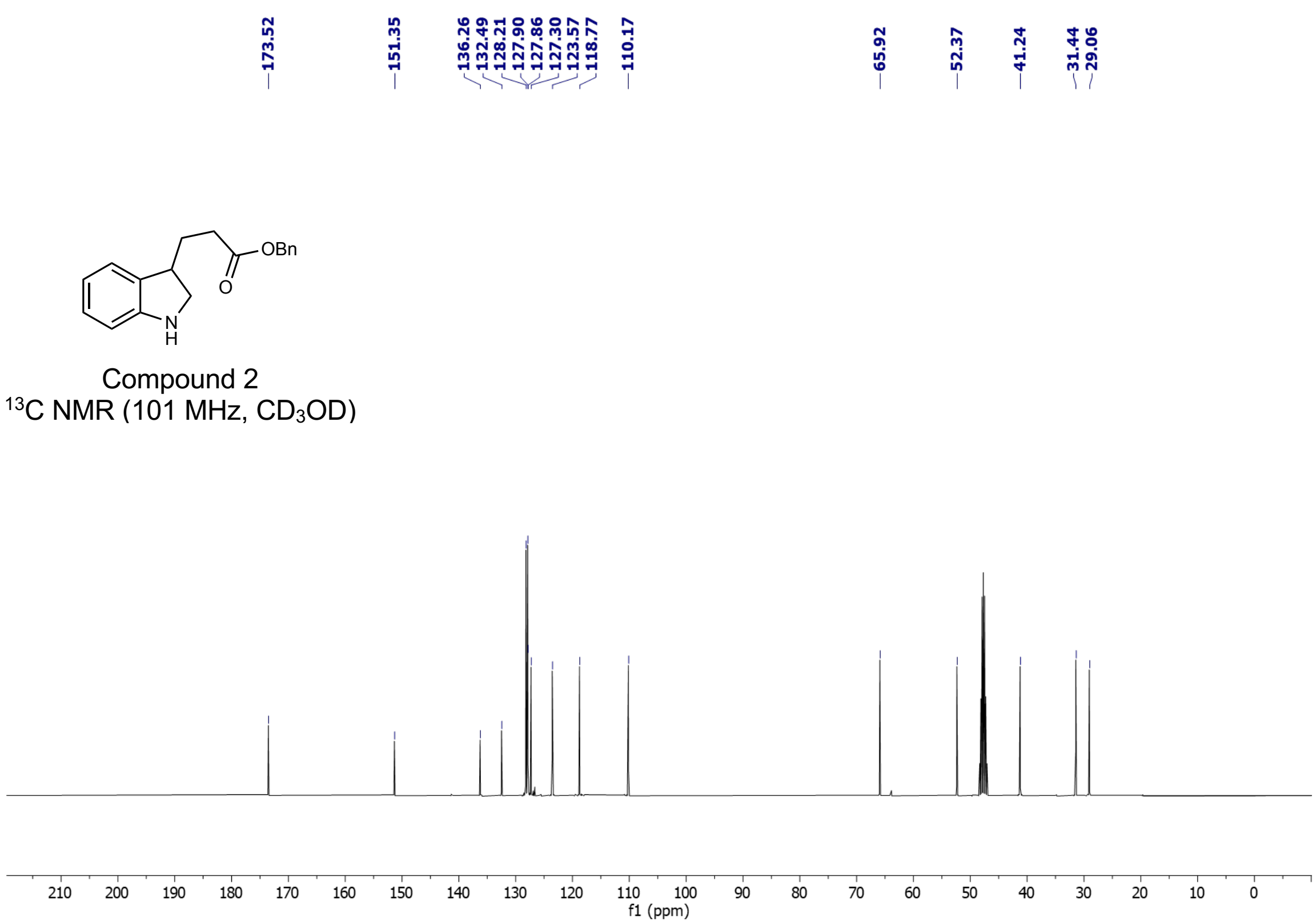

Compound 2
^{13}C NMR (101 MHz, CD_3OD)

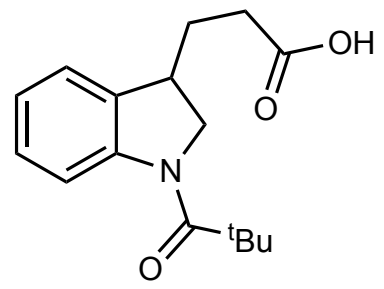

Compound 3

^{1}H NMR (600 MHz, $CDCl_3$)

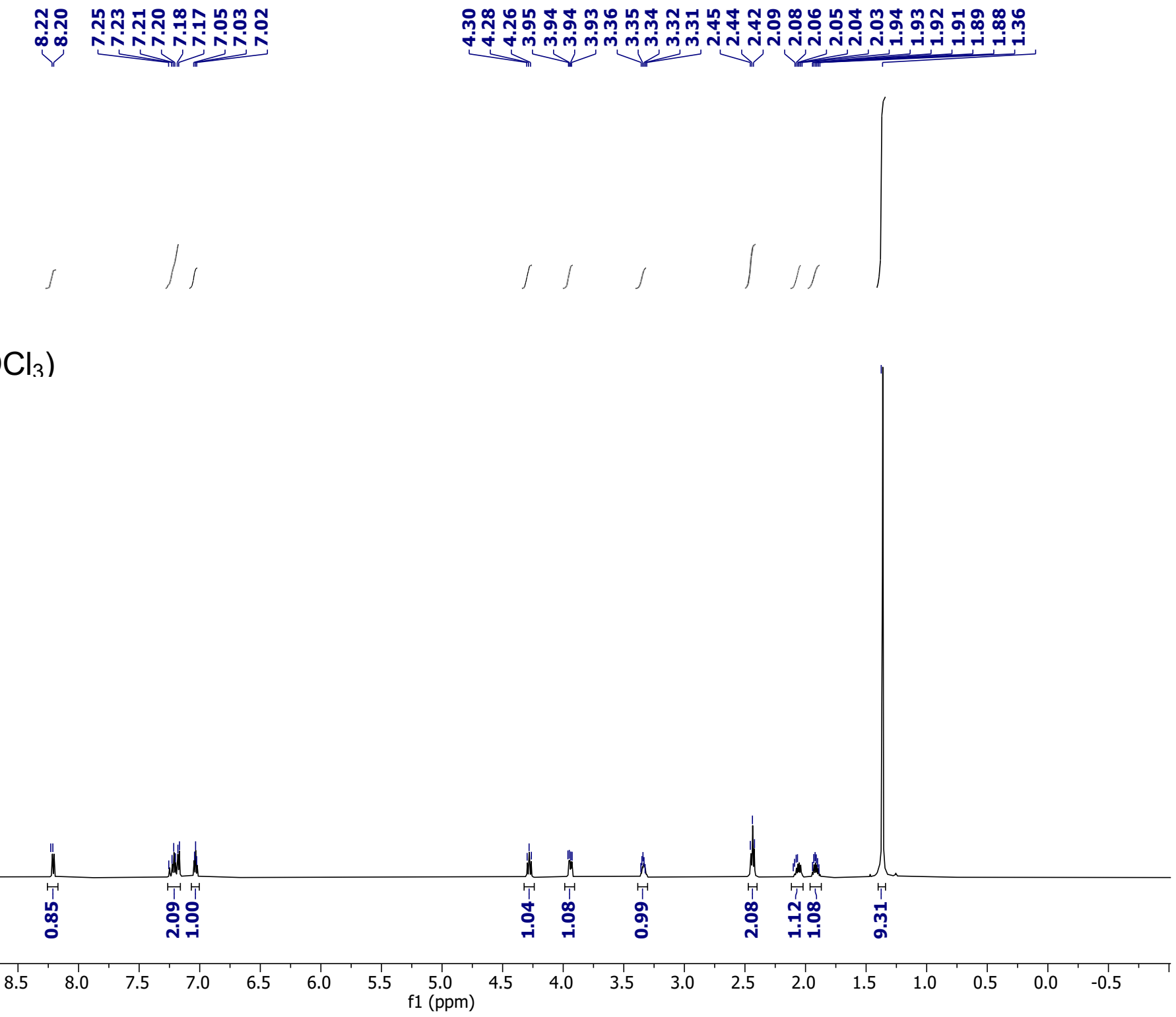

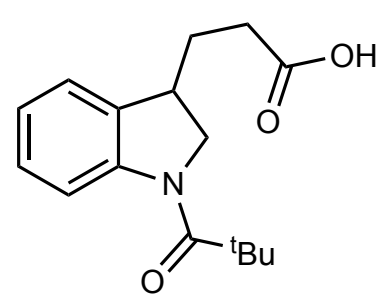

Compound 3

^{13}C NMR (150 MHz, $CDCl_3$)

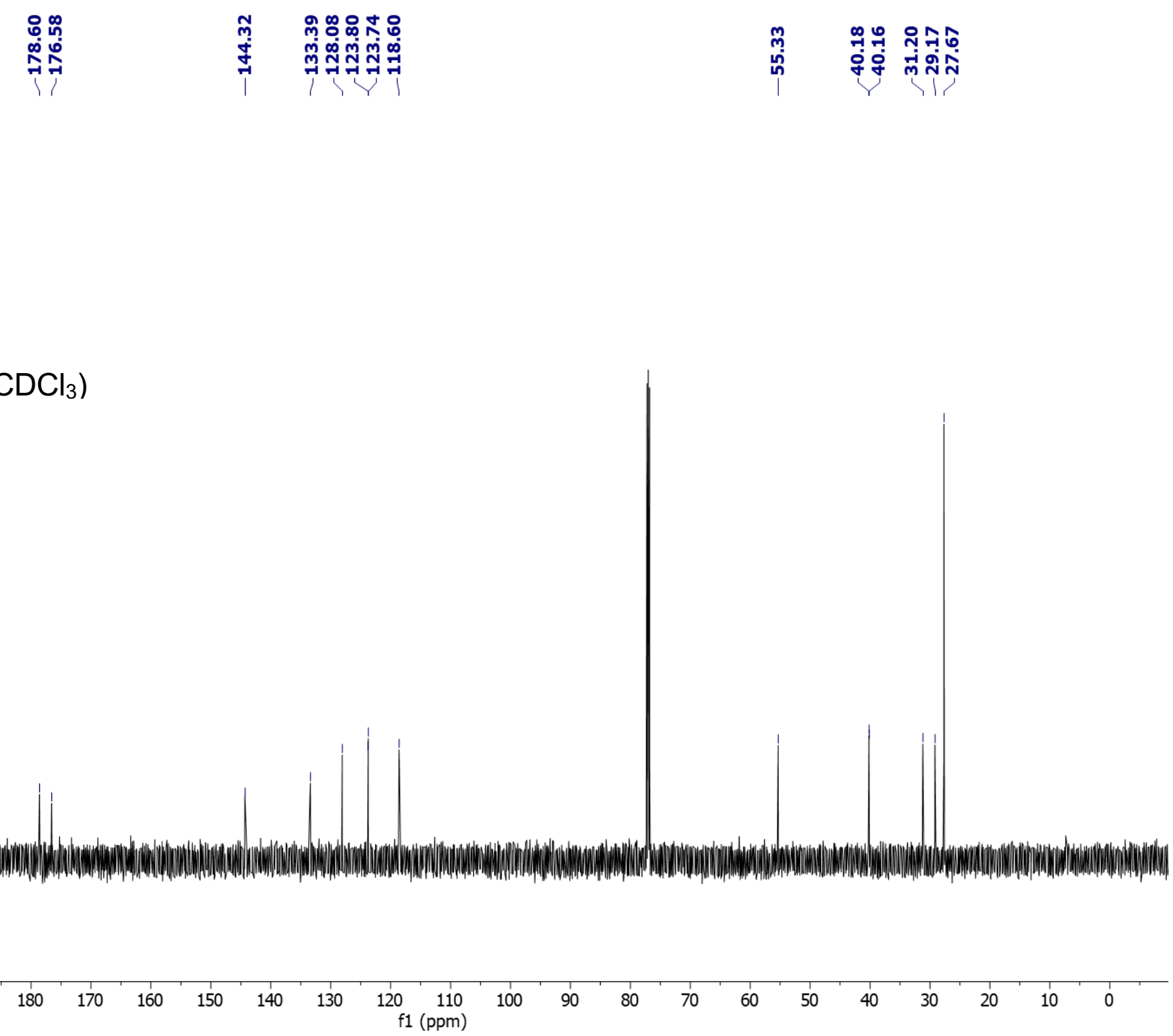

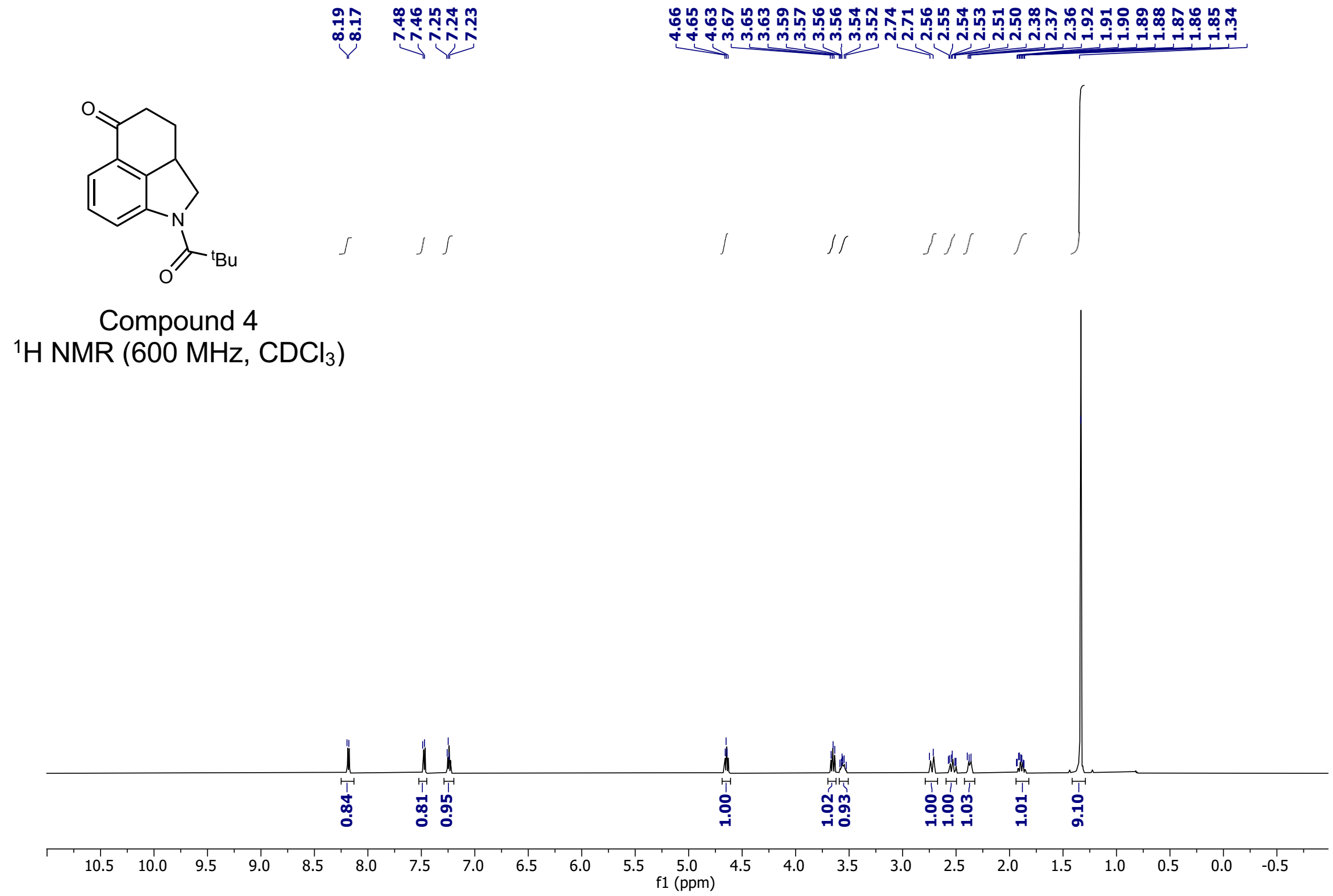

Compound 4
^{1}H NMR (600 MHz, $CDCl_3$)

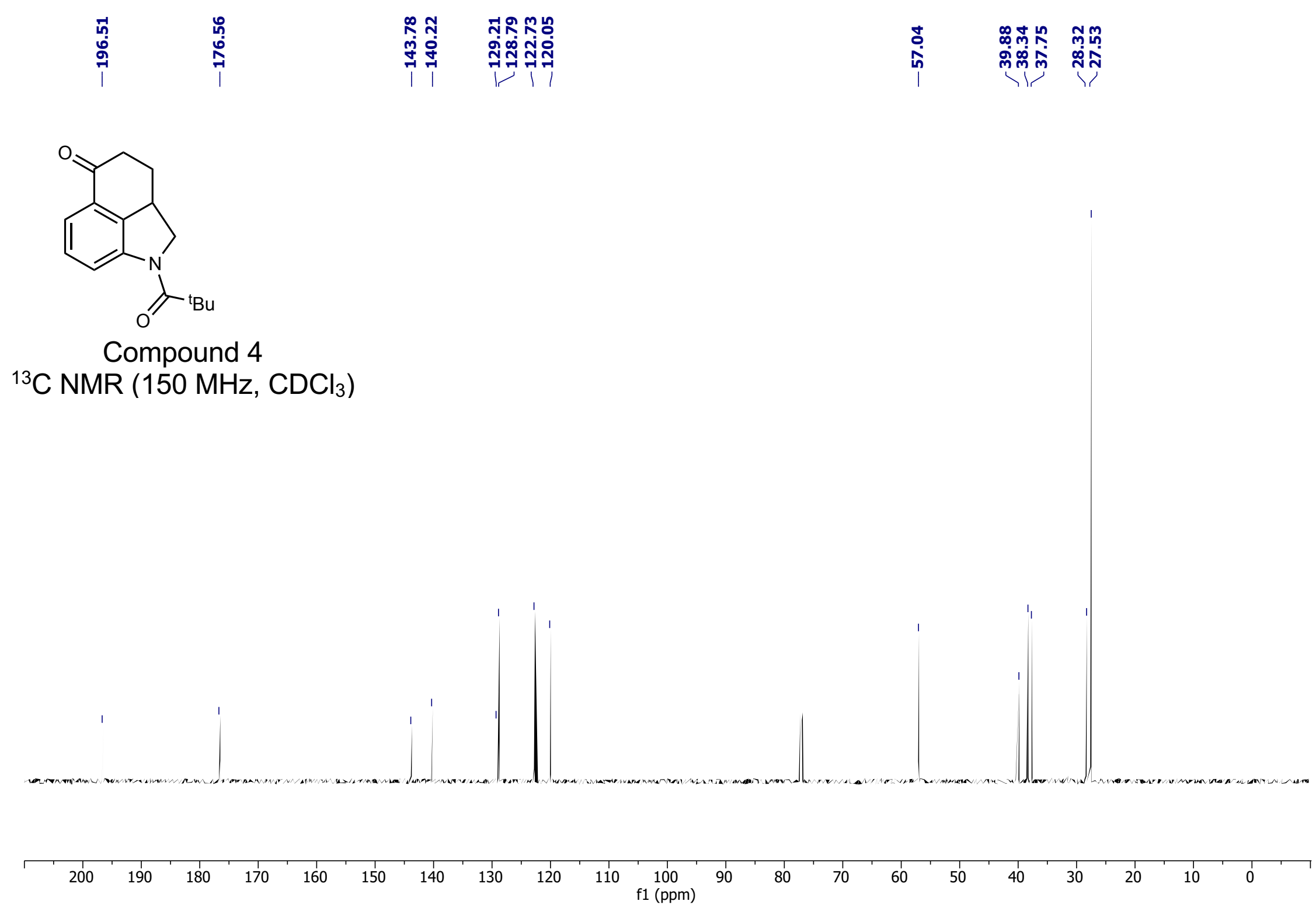

Compound 4
^{13}C NMR (150 MHz, $CDCl_3$)

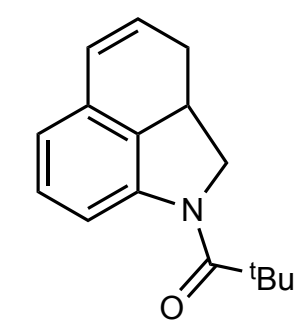

Compound 5
^{1}H NMR (600 MHz, $CDCl_3$)

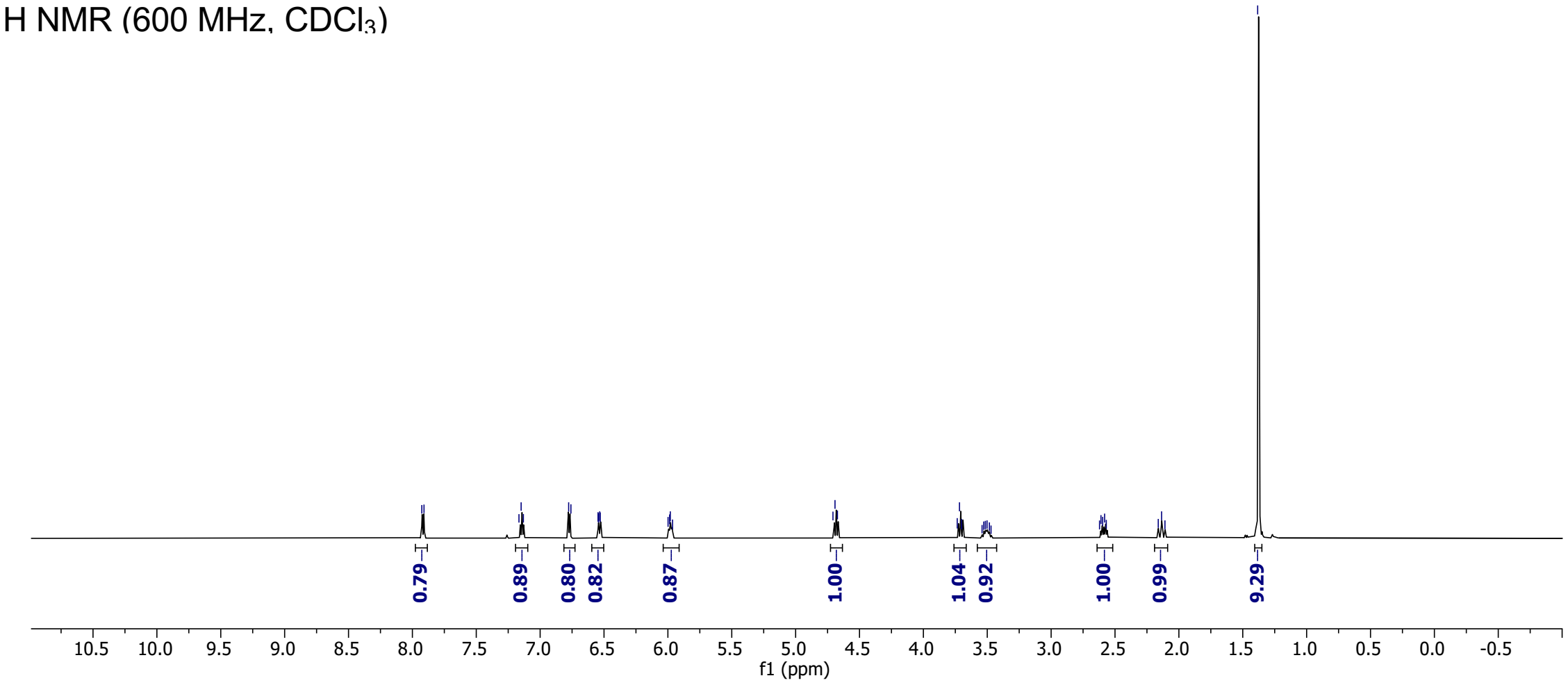

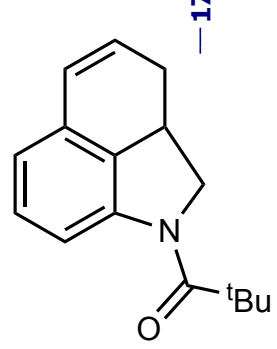

Compound 5
^{13}C NMR (150 MHz, $CDCl_3$)

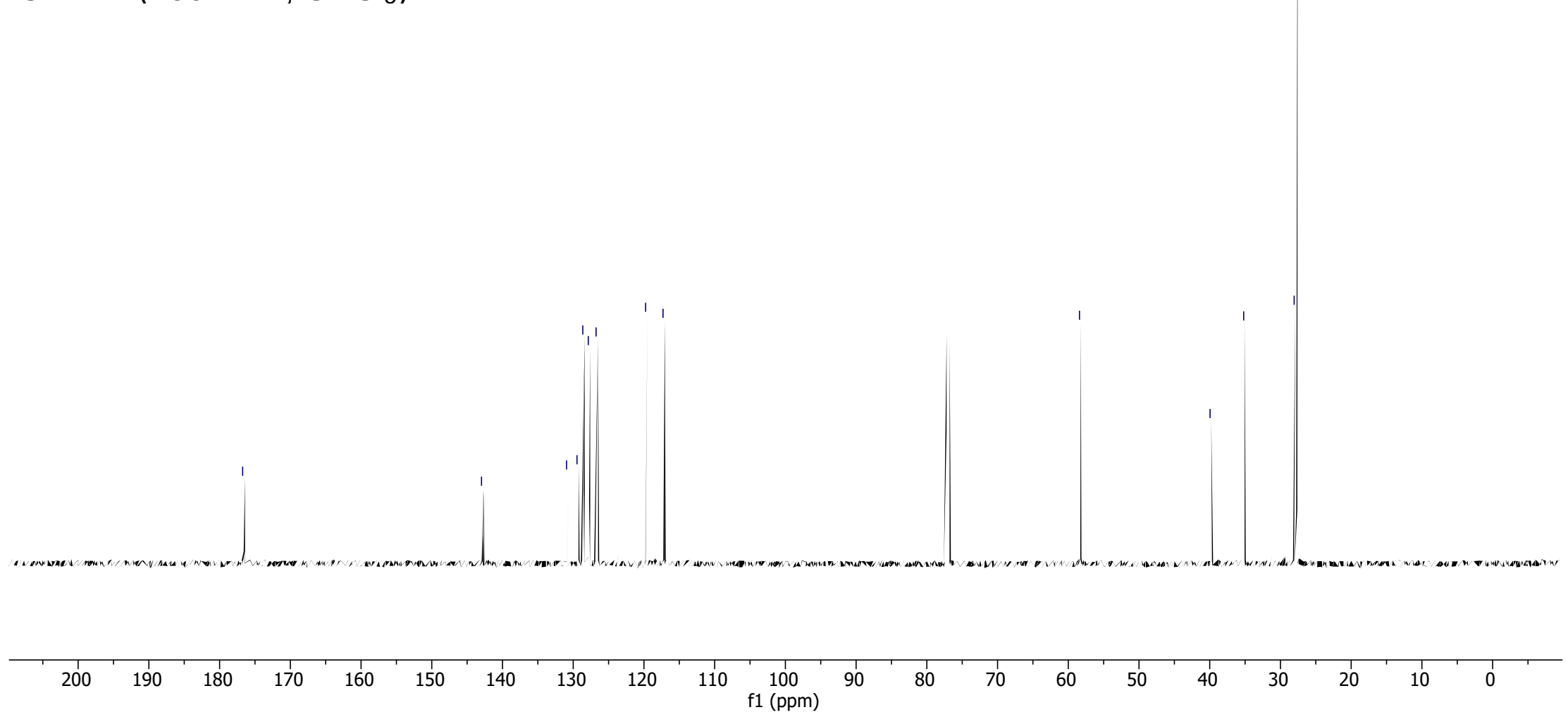

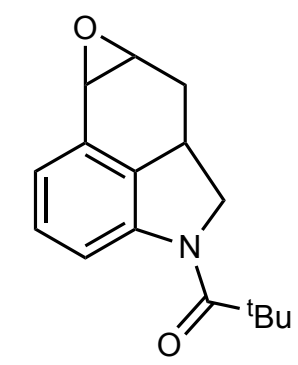

Compound 6
^{1}H NMR (600 MHz, $CDCl_3$)

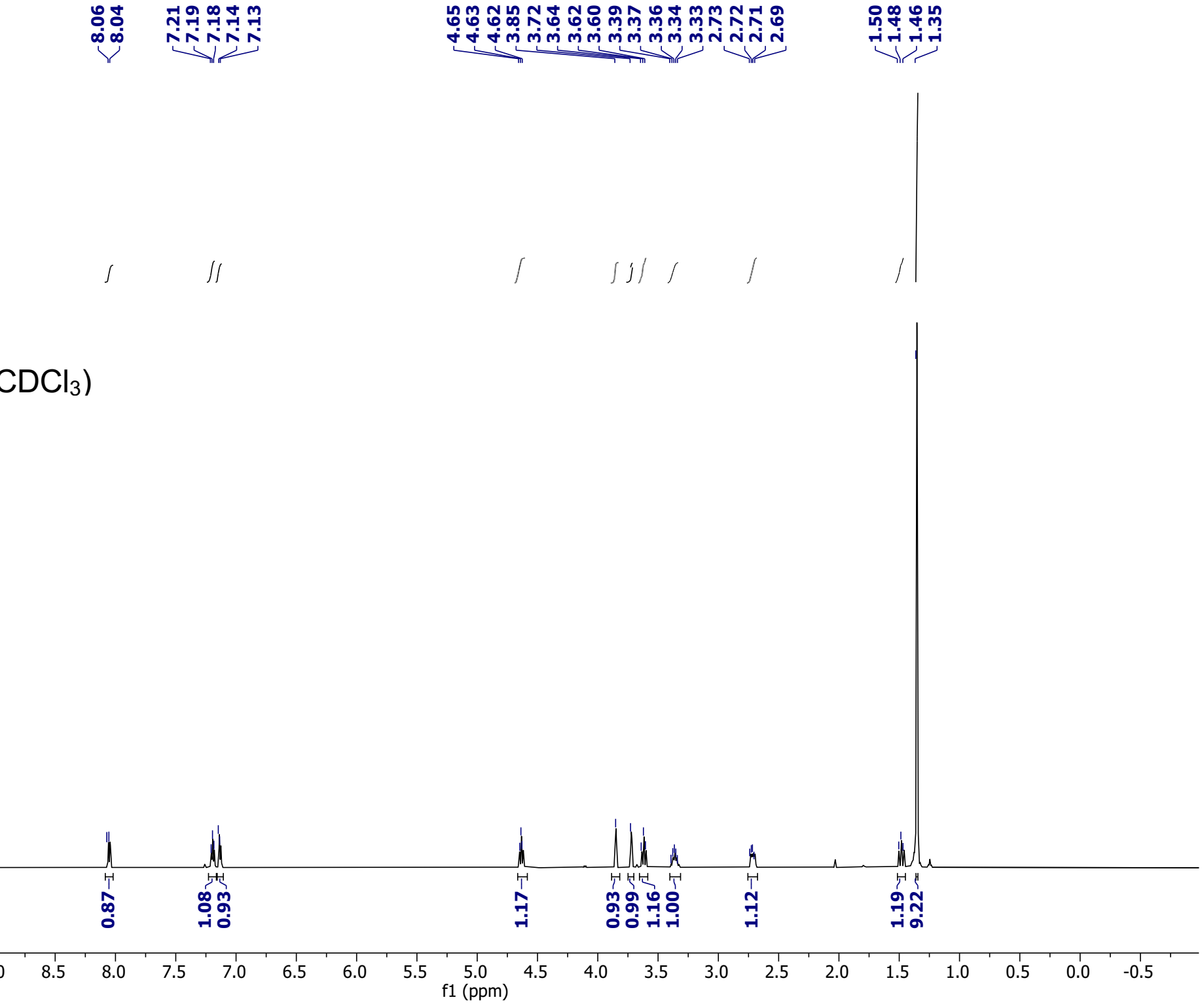

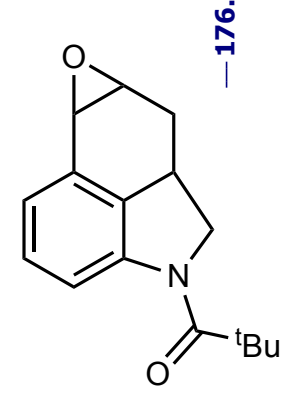

Compound 6
^{13}C NMR (150 MHz, $CDCl_3$)

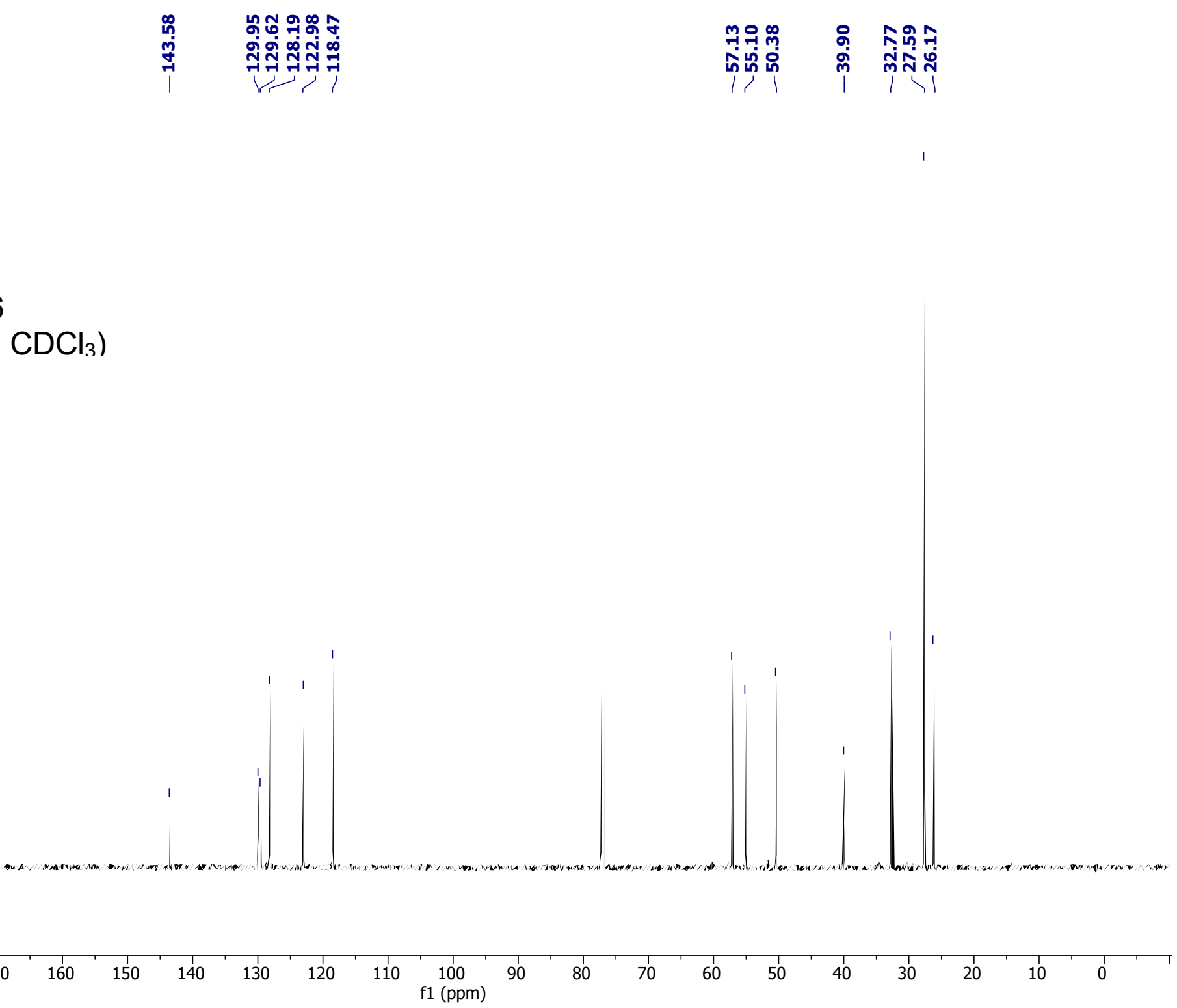

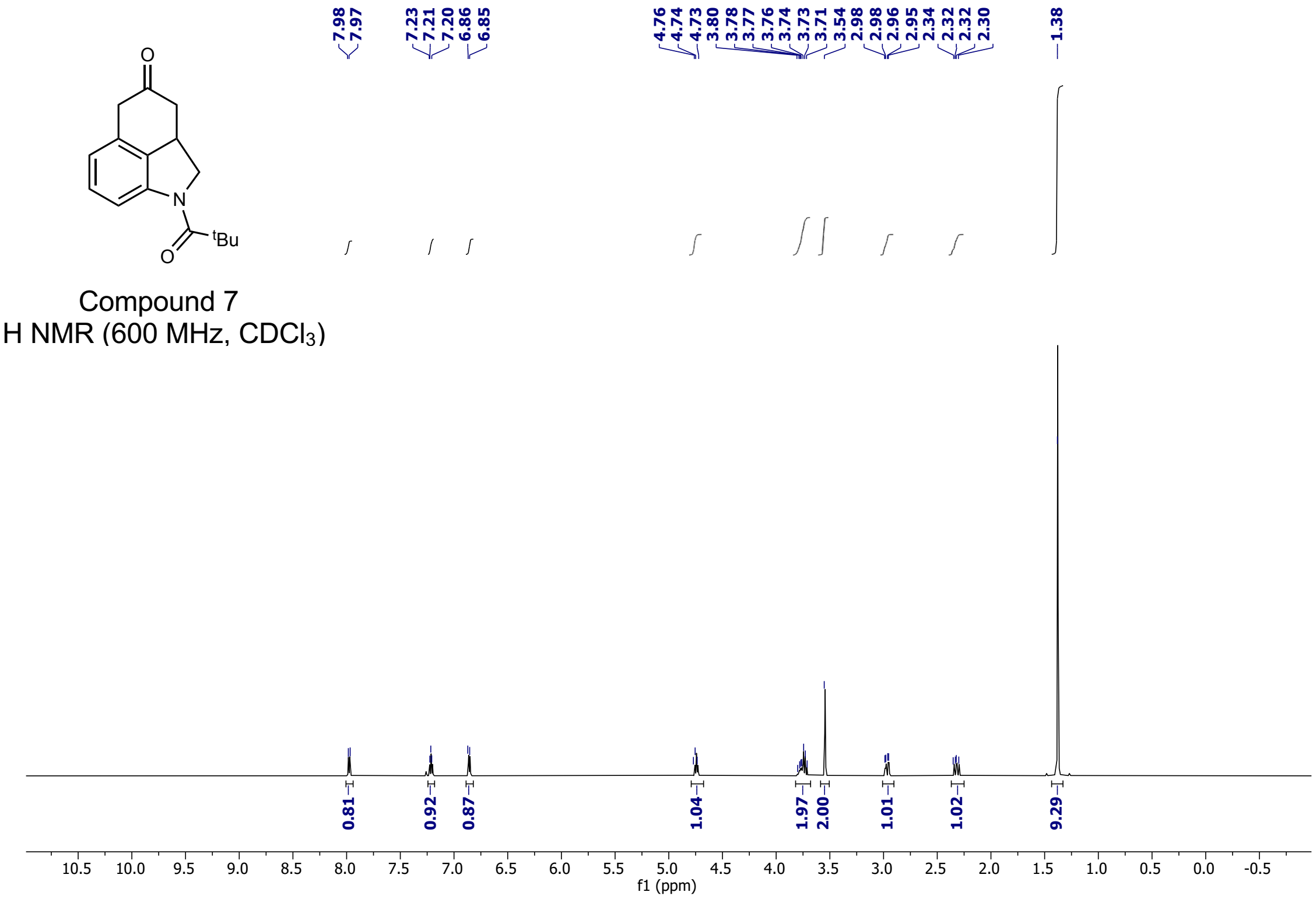

Compound 7
^1H NMR (600 MHz, $CDCl_3$)

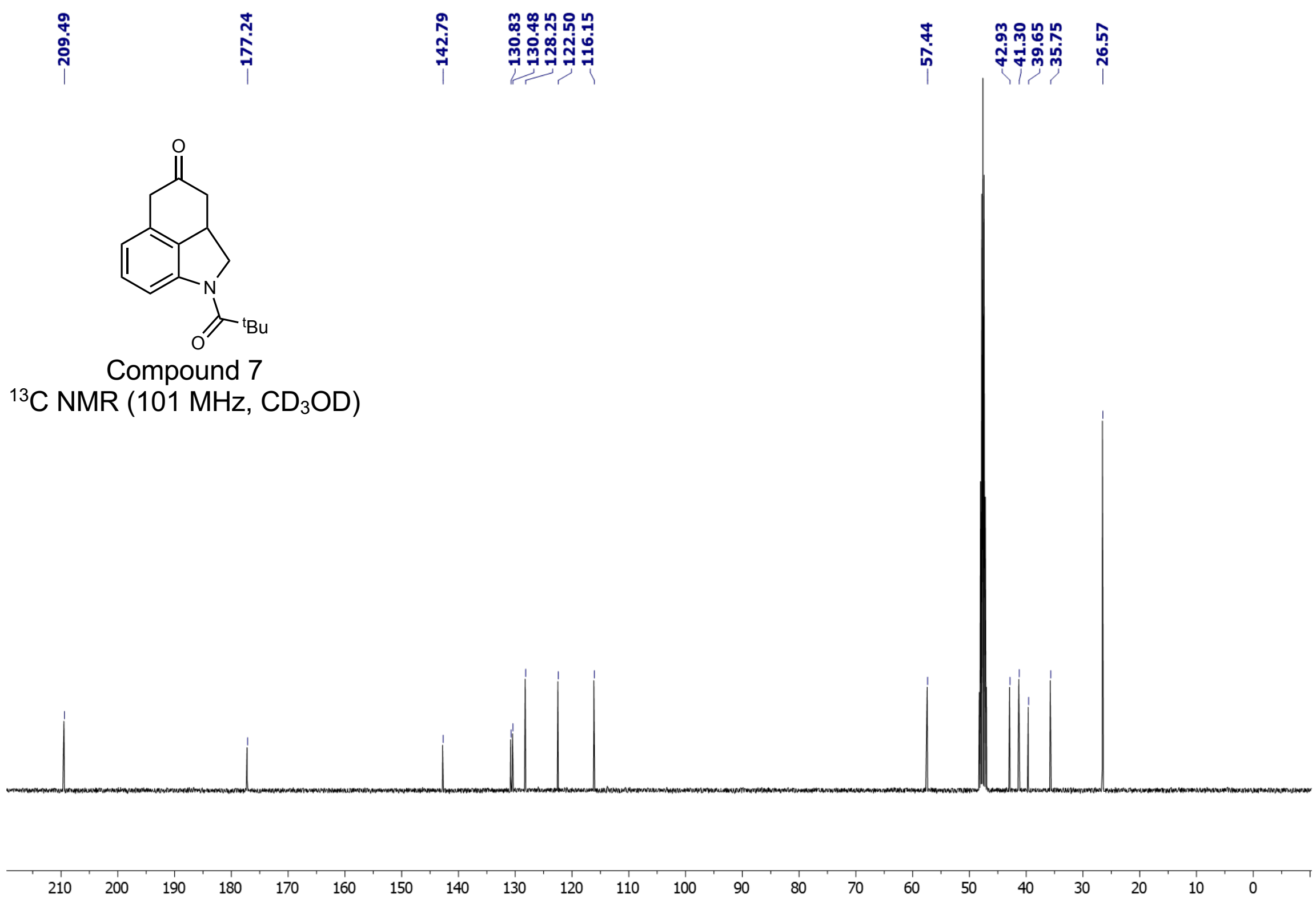

Compound 7
^{13}C NMR (101 MHz, CD_3OD)

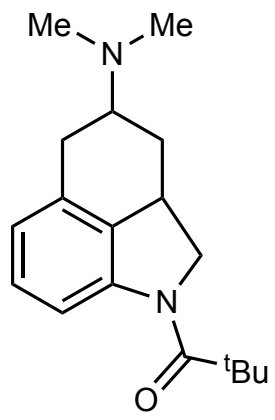

Compound 8
^{1}H NMR (600 MHz, $CDCl_3$,
mixture of diastereomers)

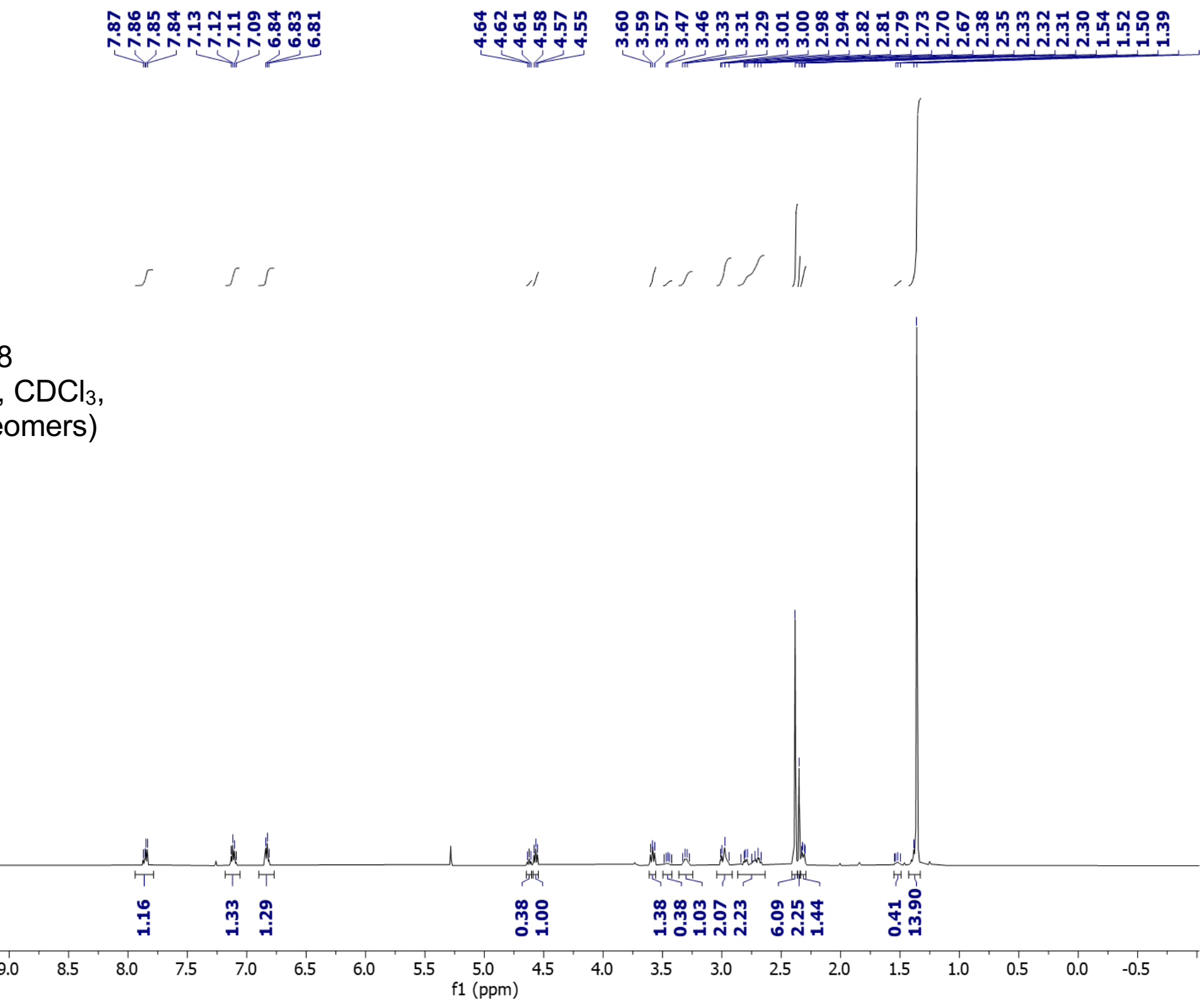

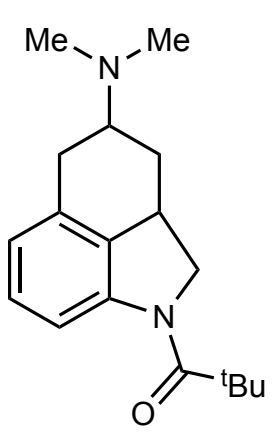

Compound 8
^{13}C NMR (150 MHz, $CDCl_3$,
mixture of diastereomers)

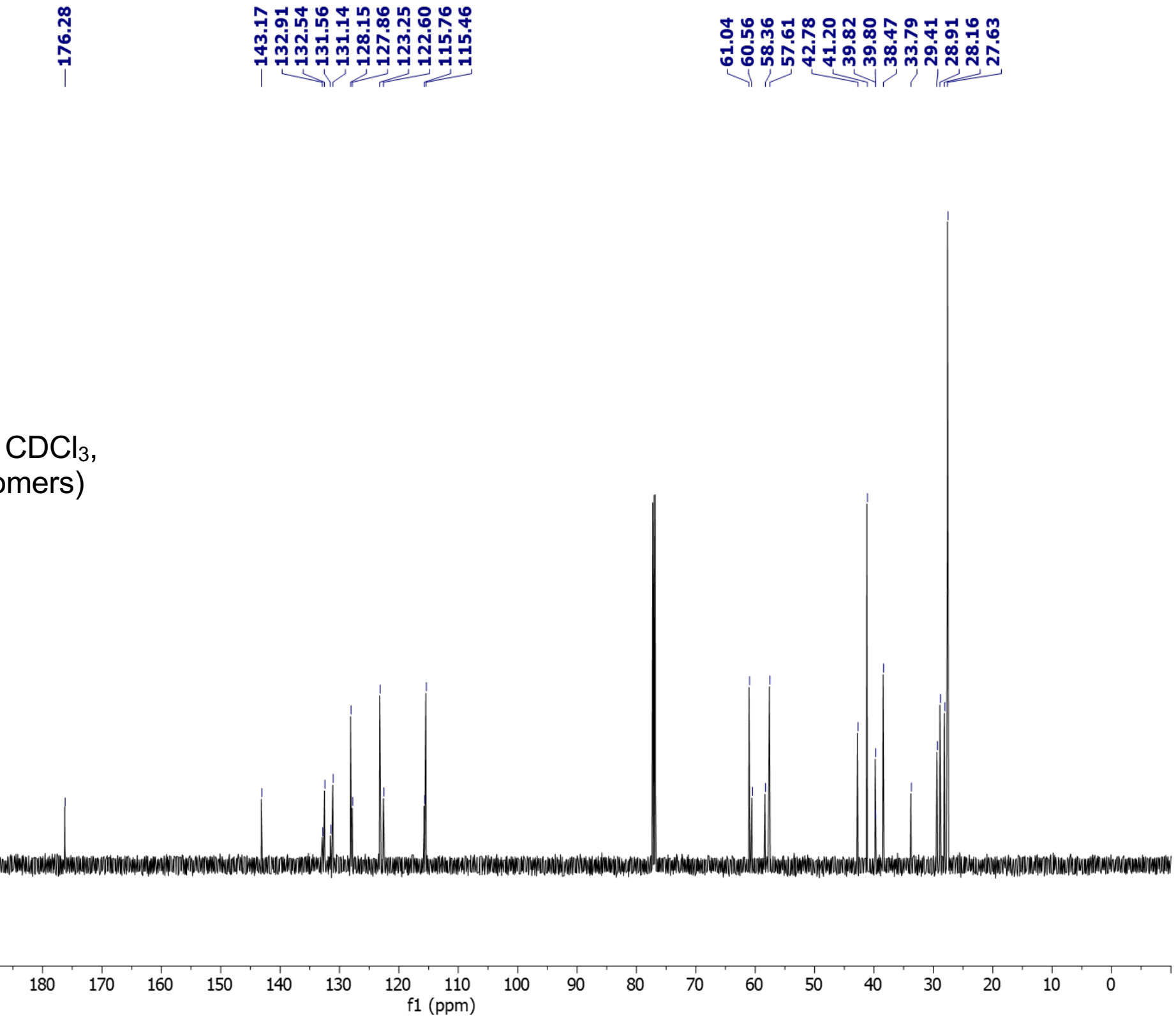

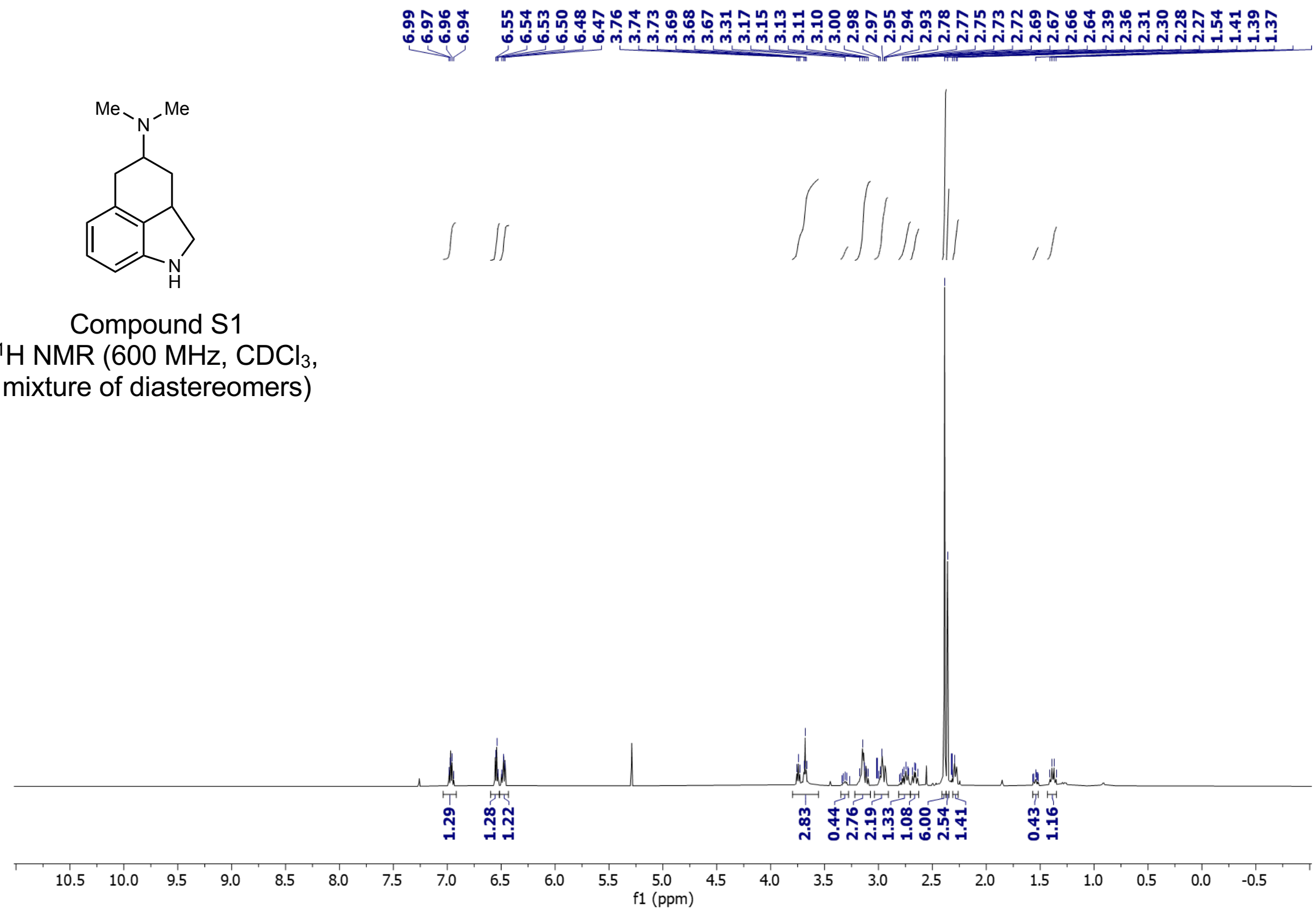

Compound S1
^{1}H NMR (600 MHz, $CDCl_3$, mixture of diastereomers)

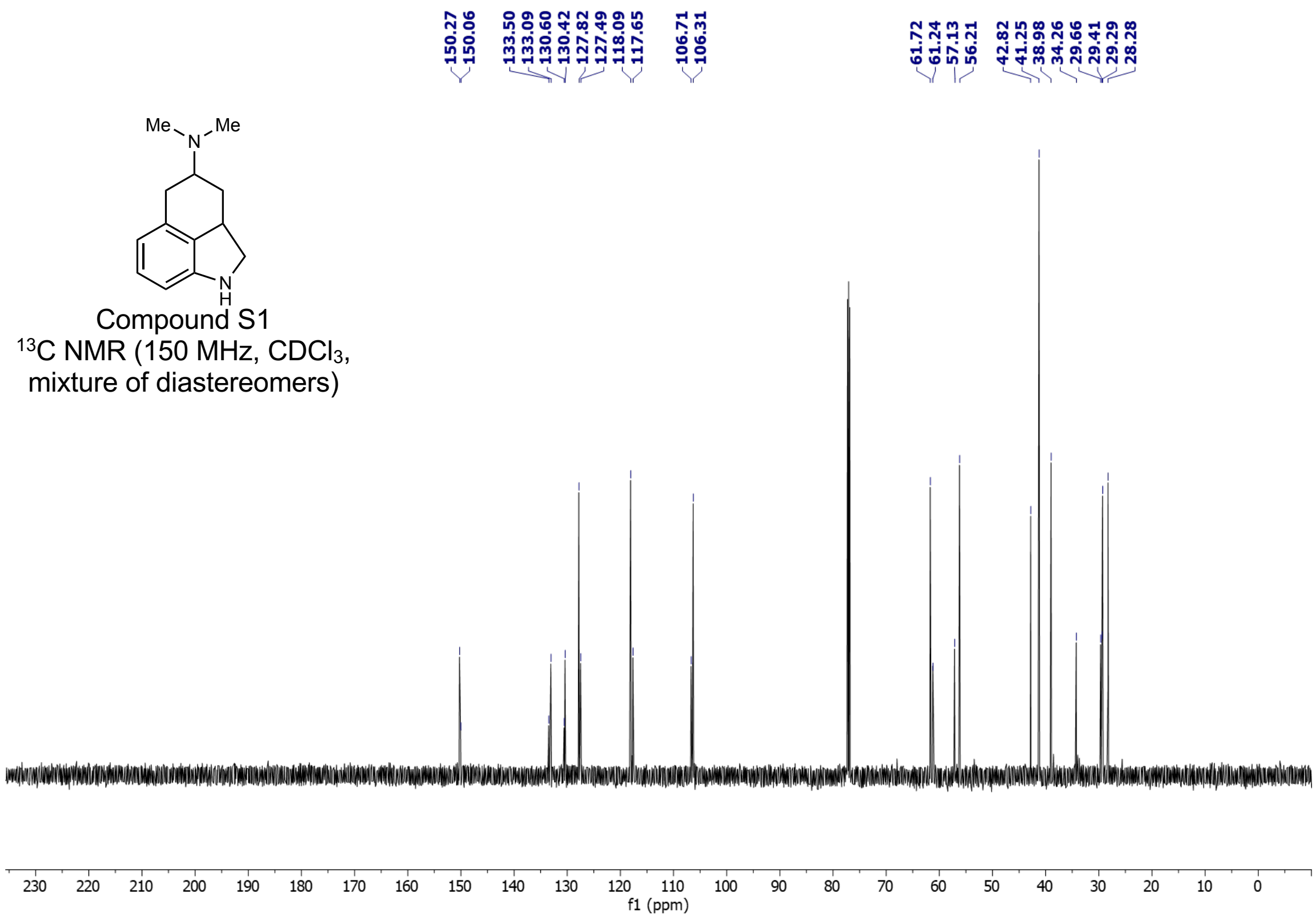

Compound S1
^{13}C NMR (150 MHz, $CDCl_3$, mixture of diastereomers)

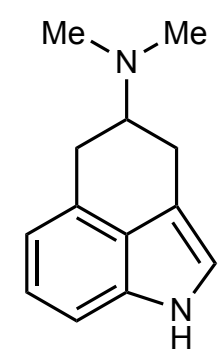

UCD0094 freebase
^{1}H NMR (600 MHz, $CDCl_3$)

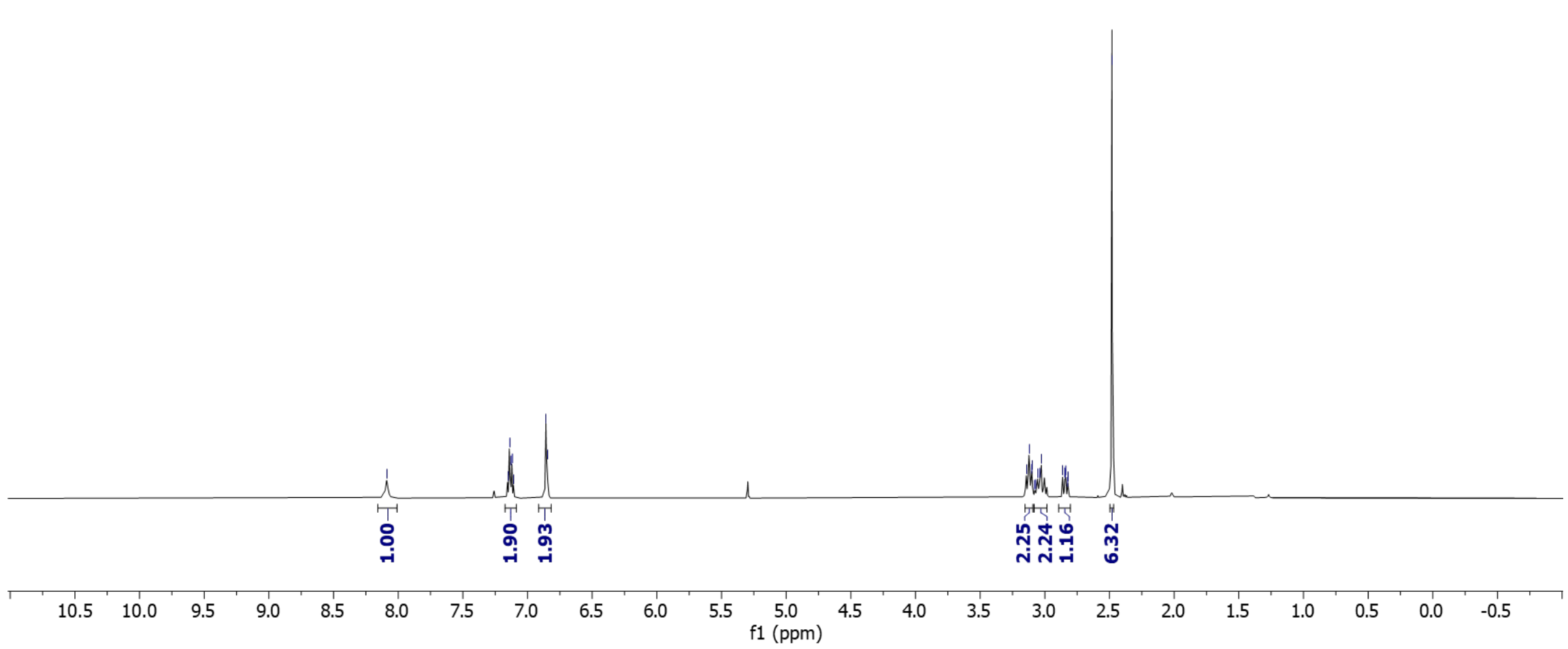

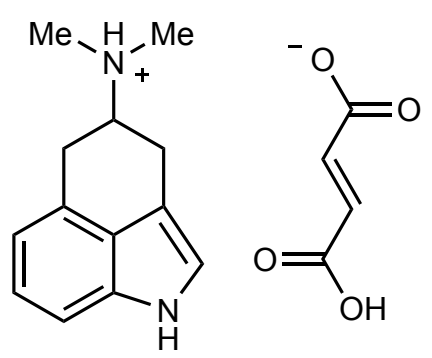

UCD0094 fumarate
^{1}H NMR (600 MHz, CD_3OD)

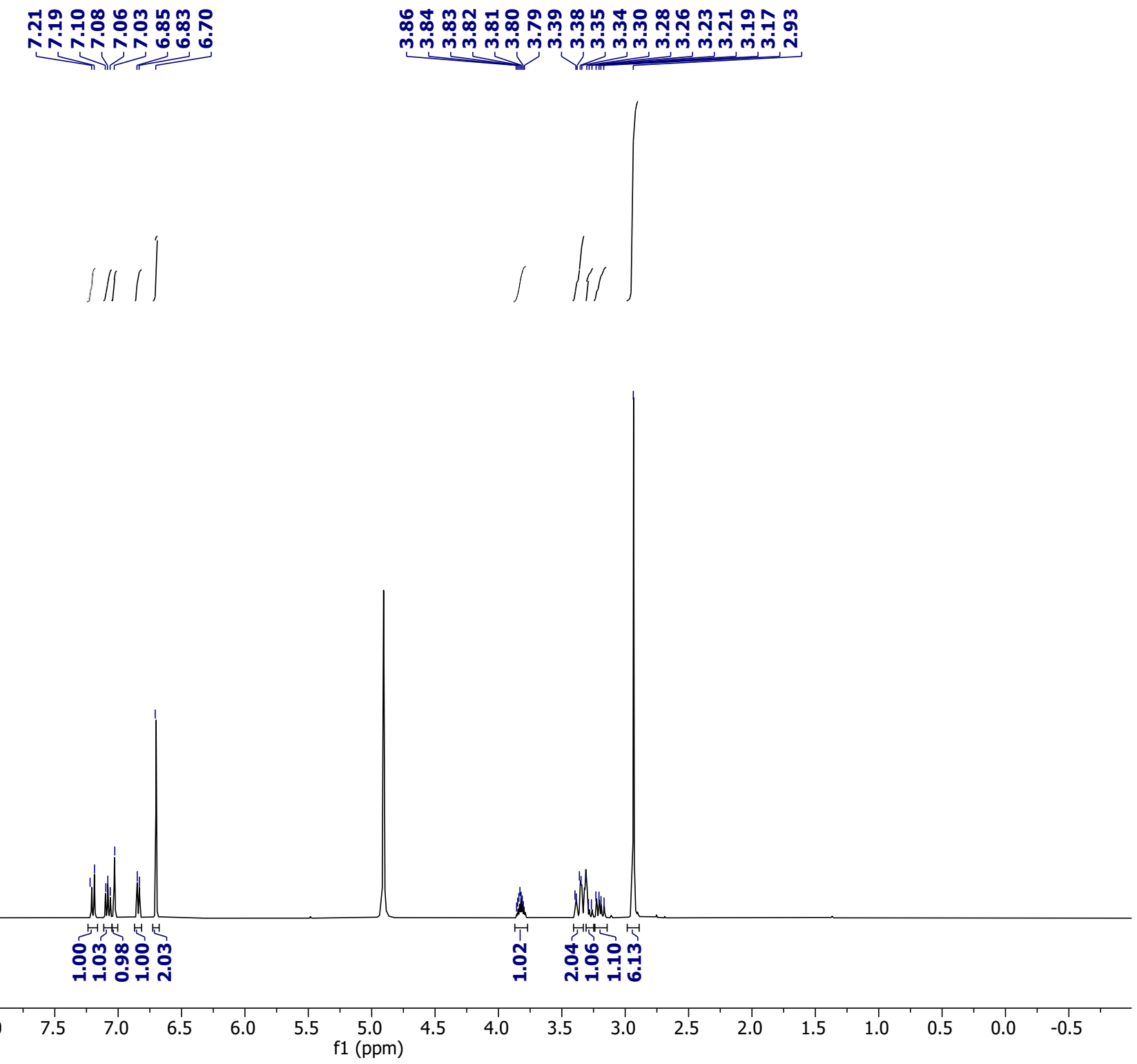

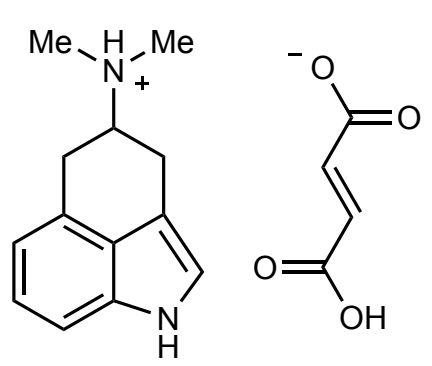

UCD0094 fumarate
^{13}C NMR (150 MHz, CD_3OD)

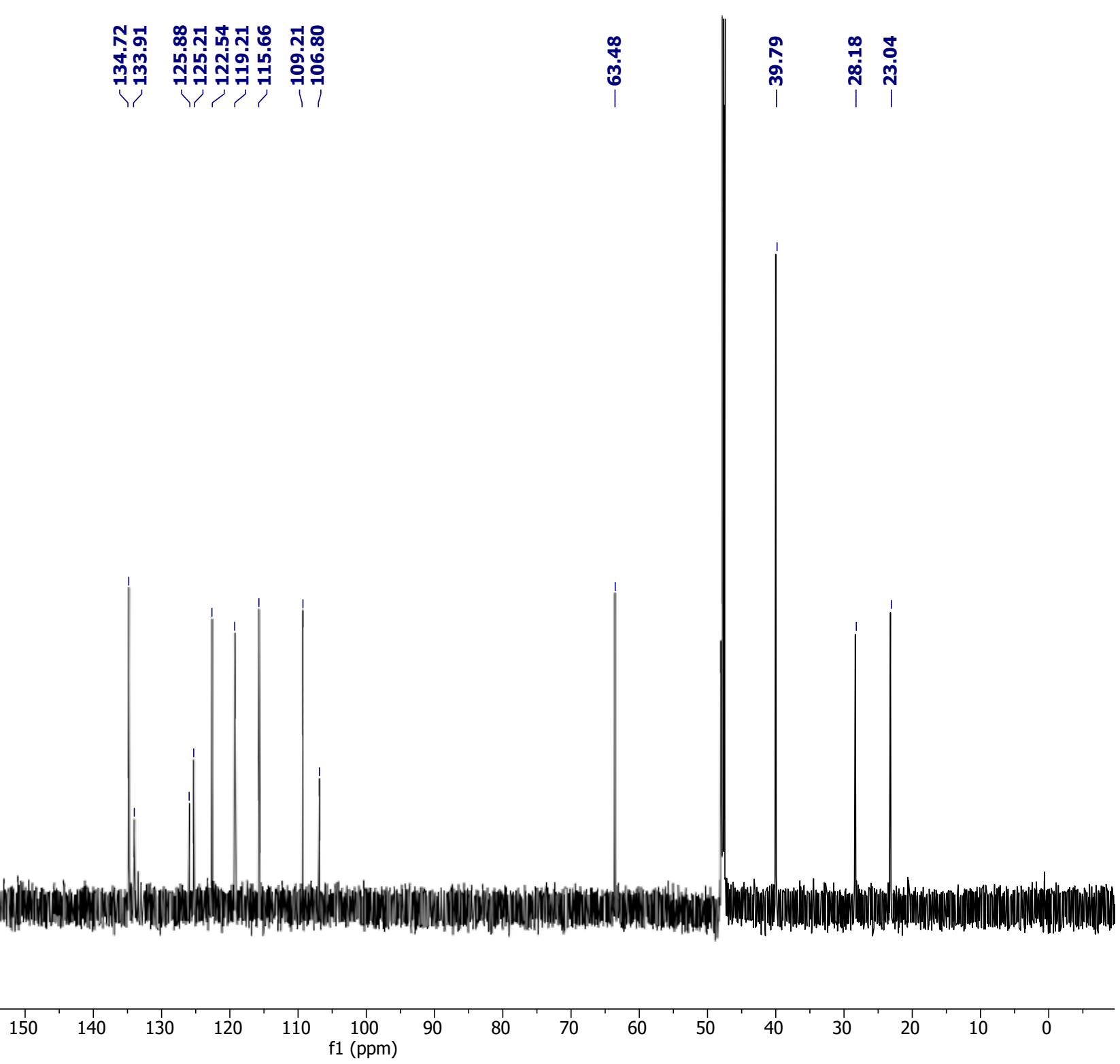

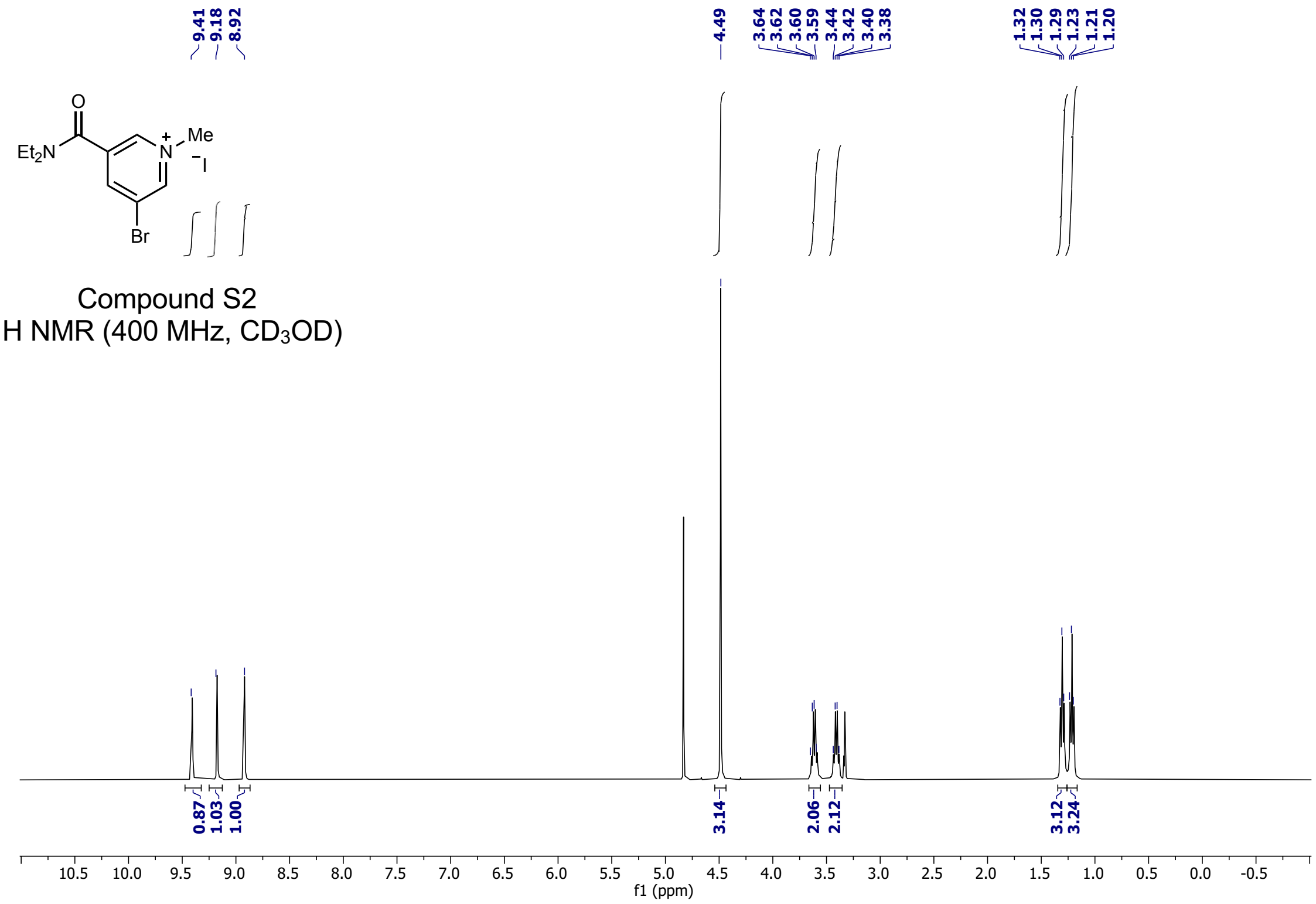

Compound S2
^{1}H NMR (400 MHz, CD_3OD)

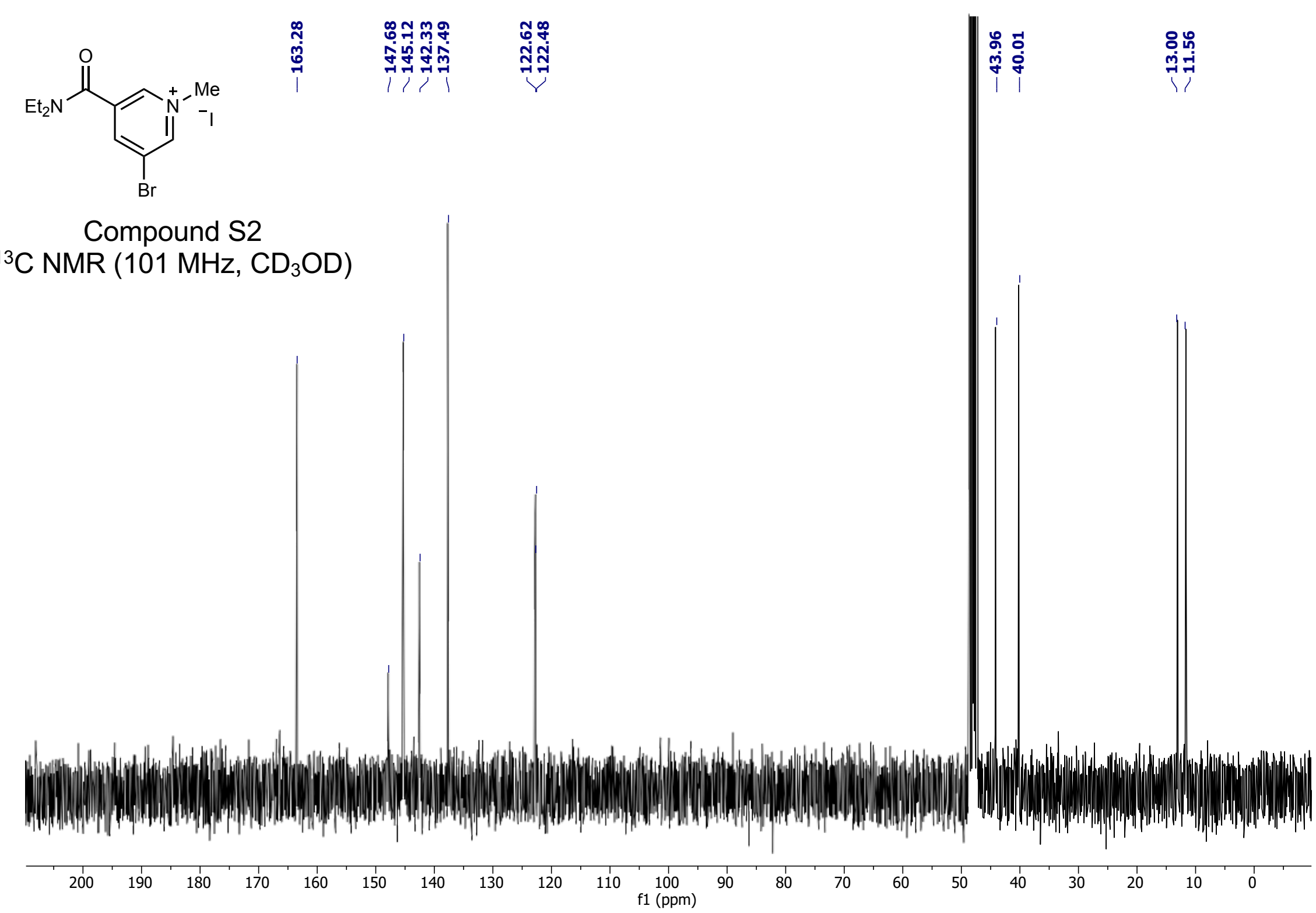

Compound S2
^{13}C NMR (101 MHz, CD_3OD)

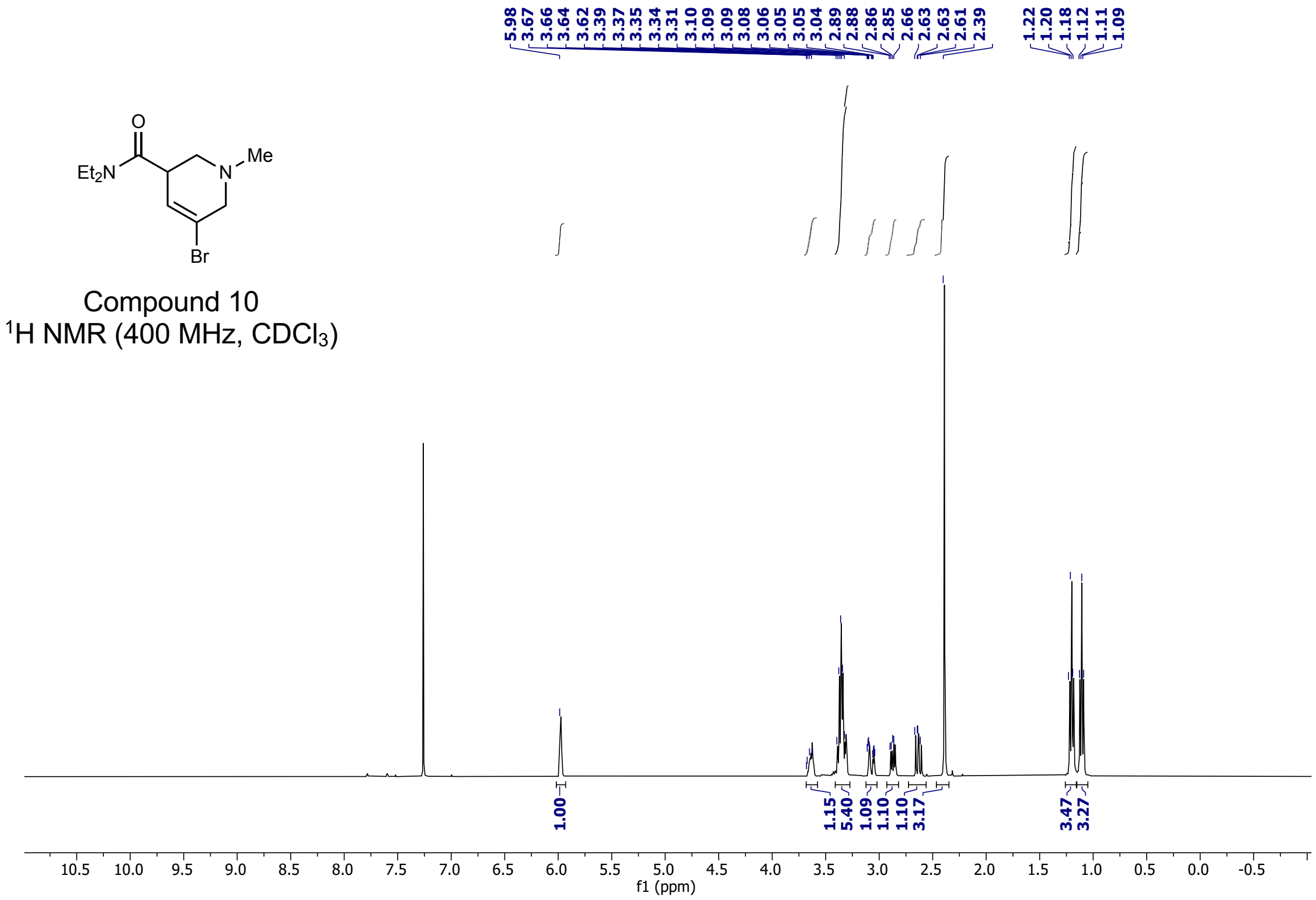

Compound 10
^{1}H NMR (400 MHz, $CDCl_3$)

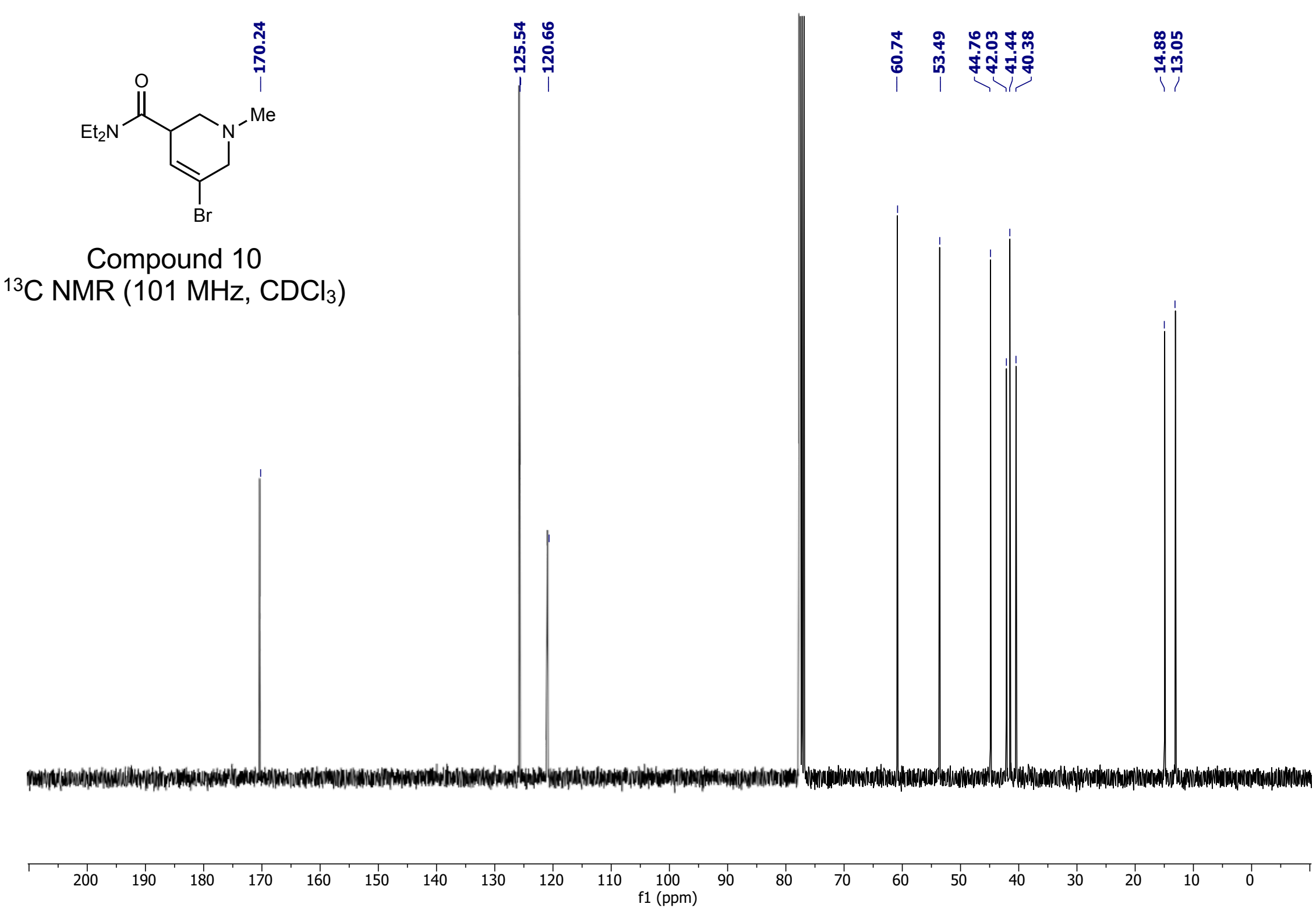

Compound 10
^{13}C NMR (101 MHz, $CDCl_3$)

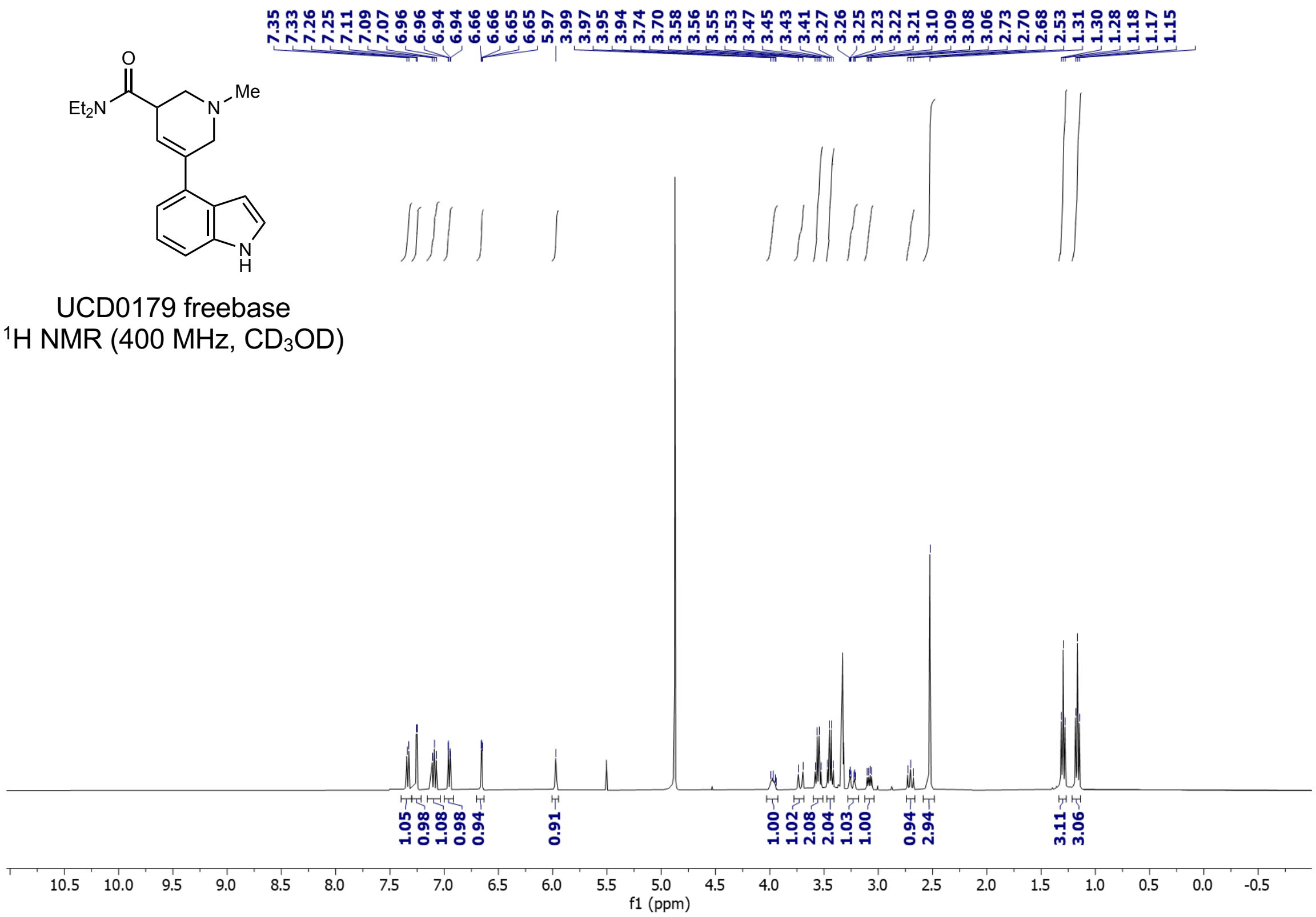

UCD0179 freebase
^{1}H NMR (400 MHz, CD_3OD)

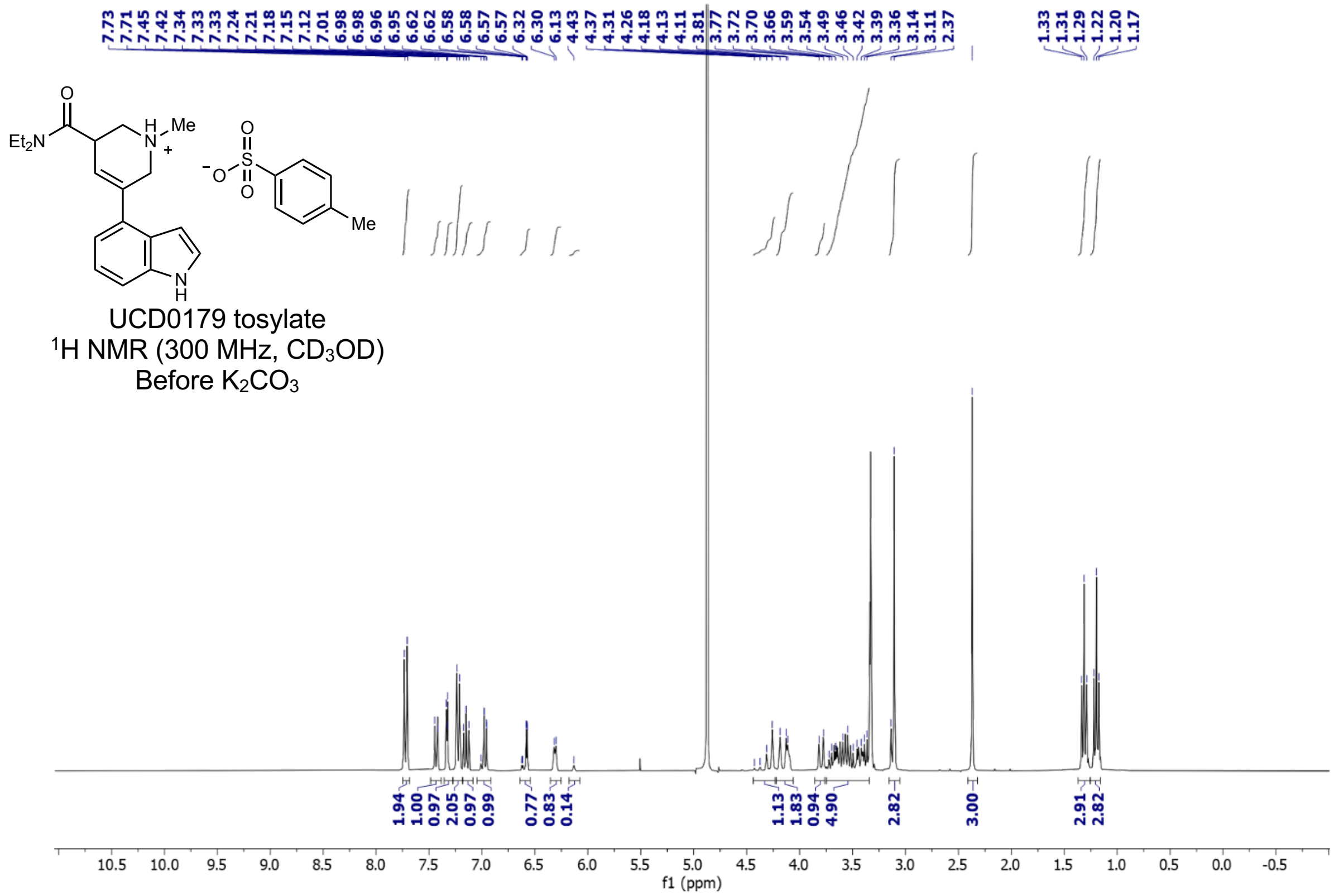

UCD0179 tosylate
^{1}H NMR (300 MHz, CD_3OD)
Before K_2CO_3

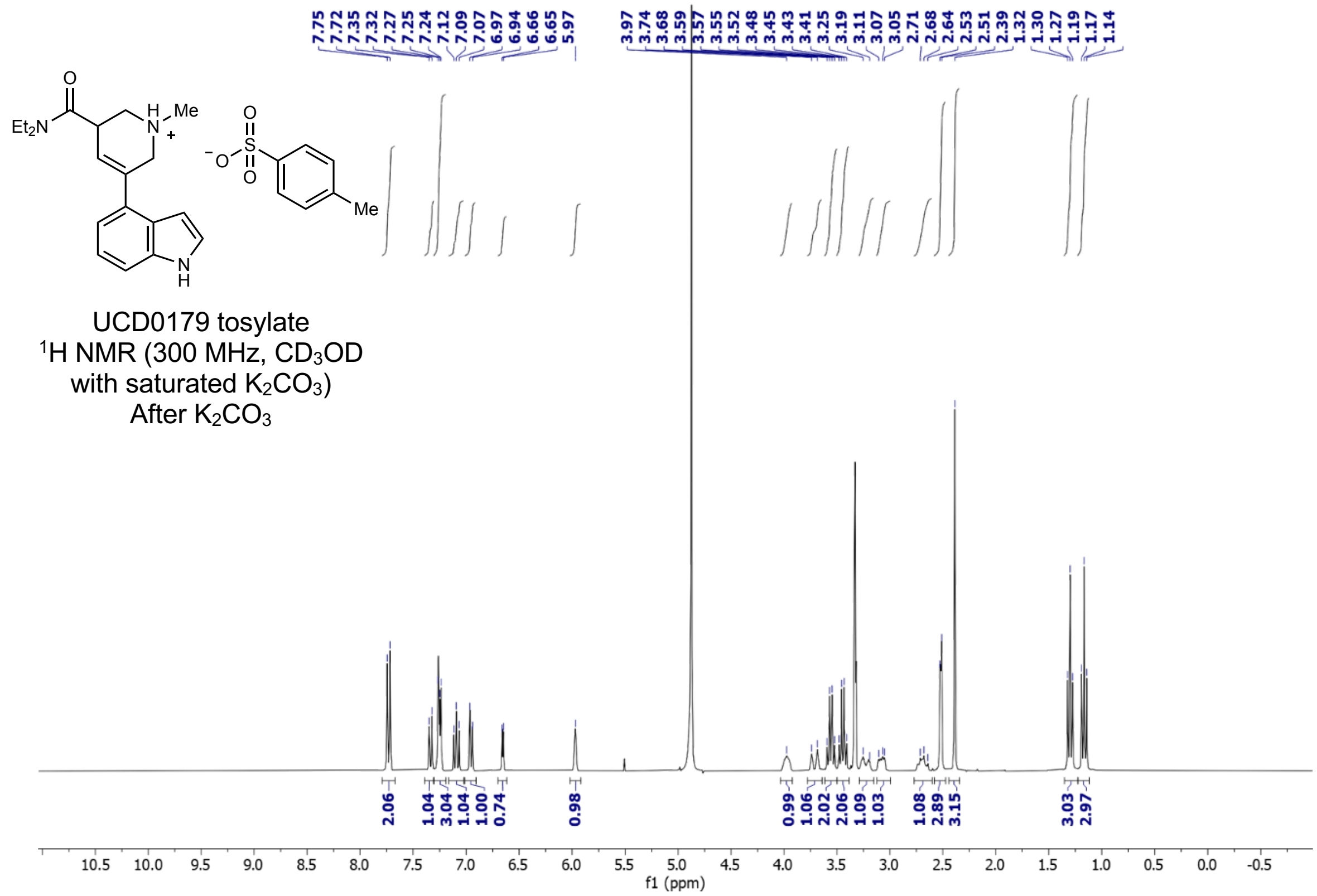

UCD0179 tosylate
^{1}H NMR (300 MHz, CD_3OD
with saturated K_2CO_3)
After K_2CO_3

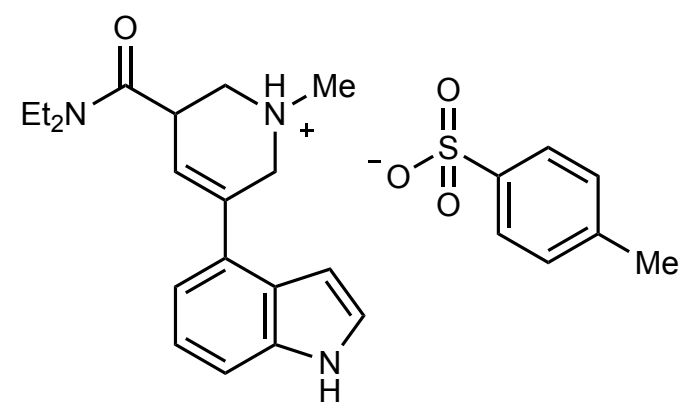

UCD0179 tosylate
^{13}C NMR (101 MHz, CD_3OD)

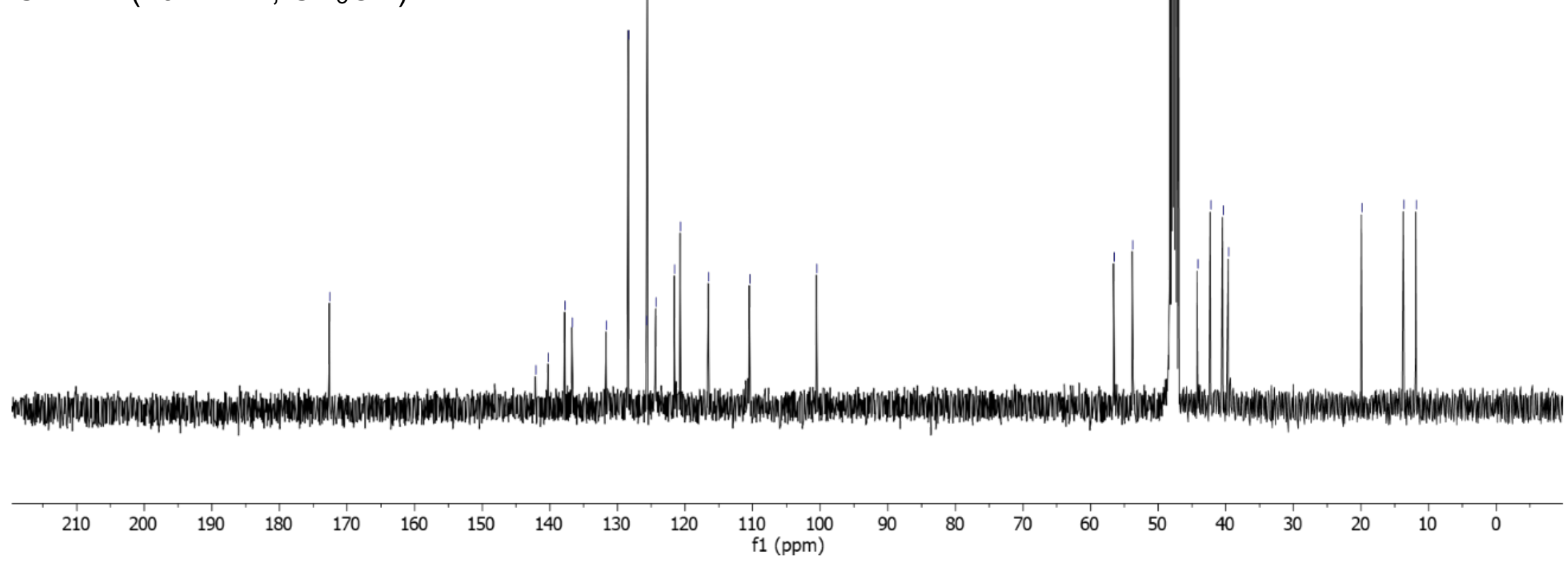

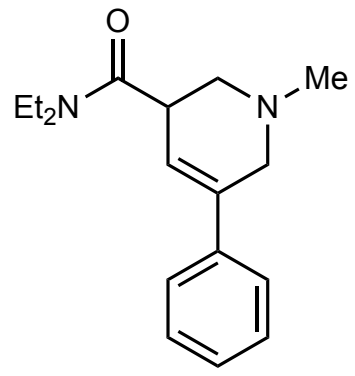

UCD0120 freebase
^{1}H NMR (300 MHz, $CDCl_3$)

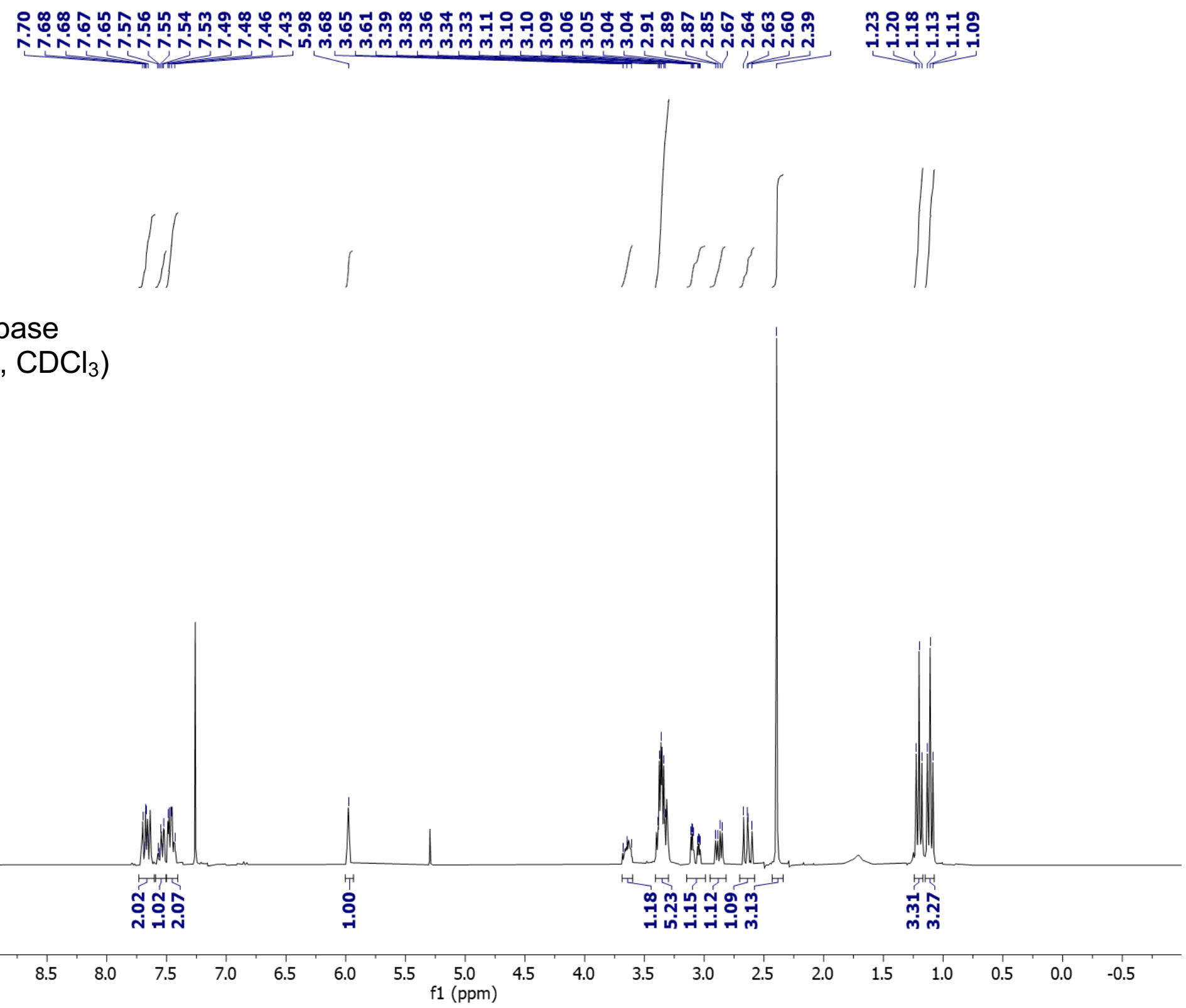

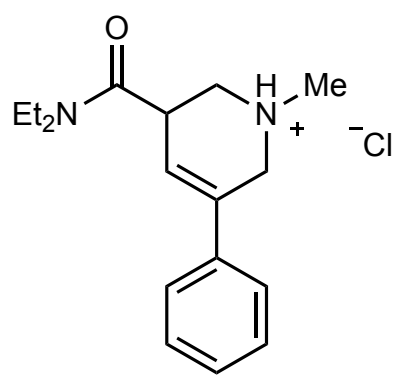

UCD0120 hydrochloride
^{1}H NMR (300 MHz, CD_3OD)

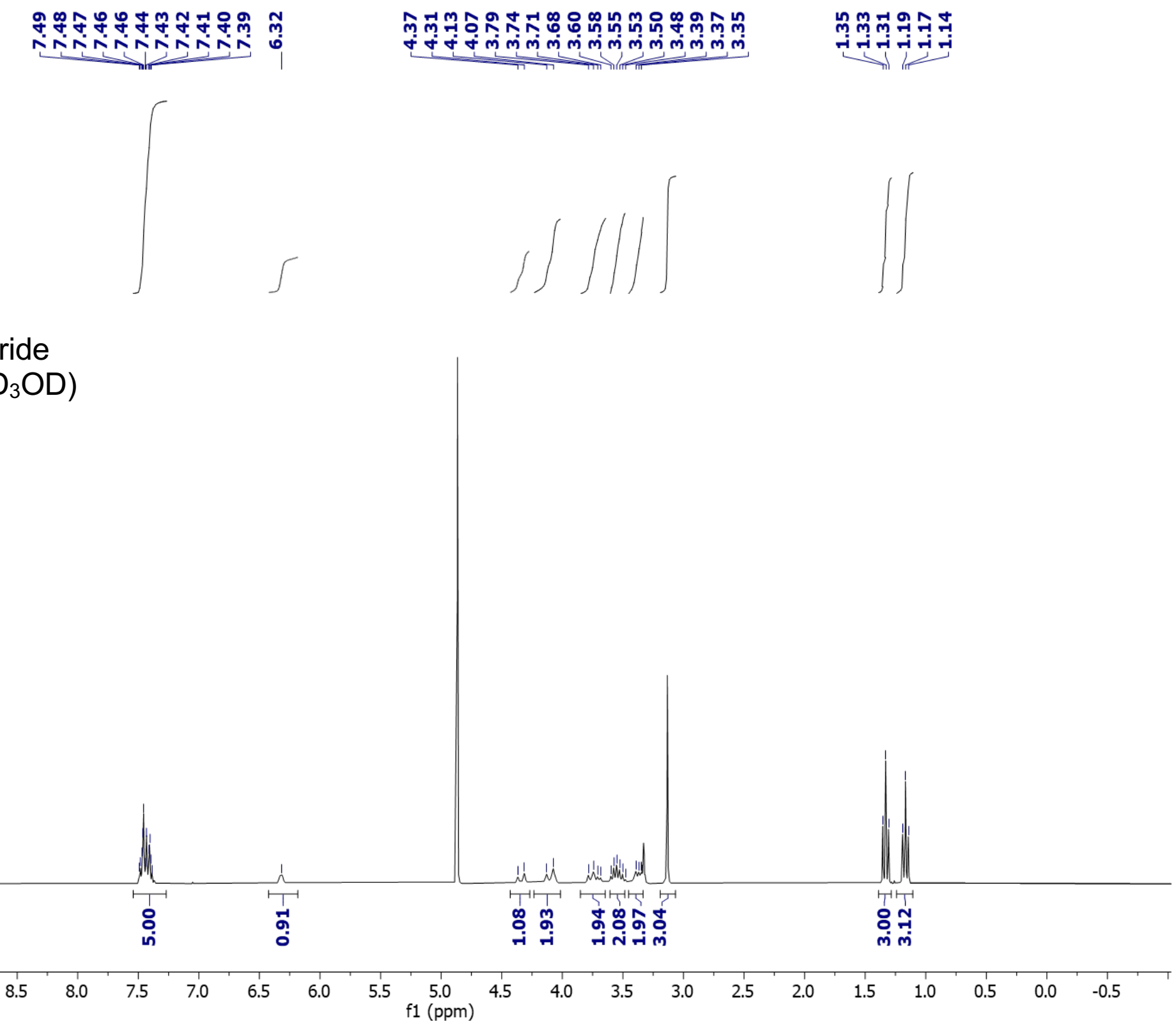

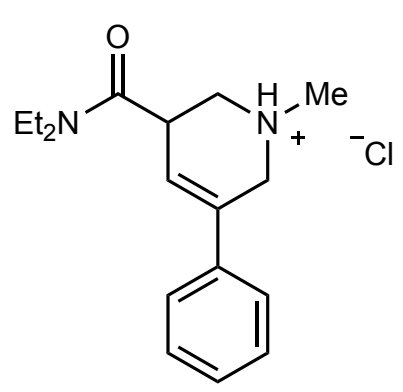

UCD0120 hydrochloride
^{13}C NMR (101 MHz, CD_3OD)

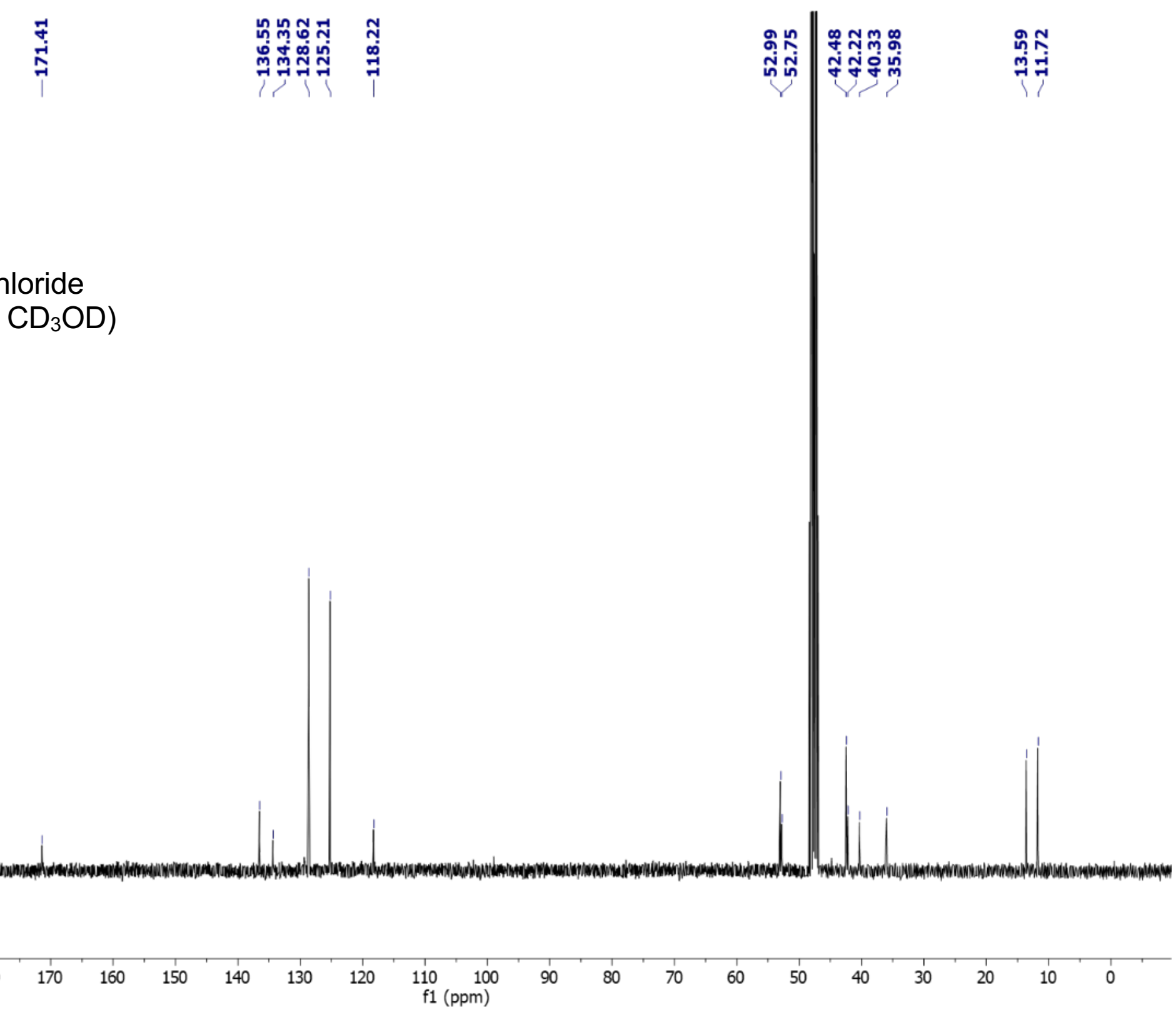

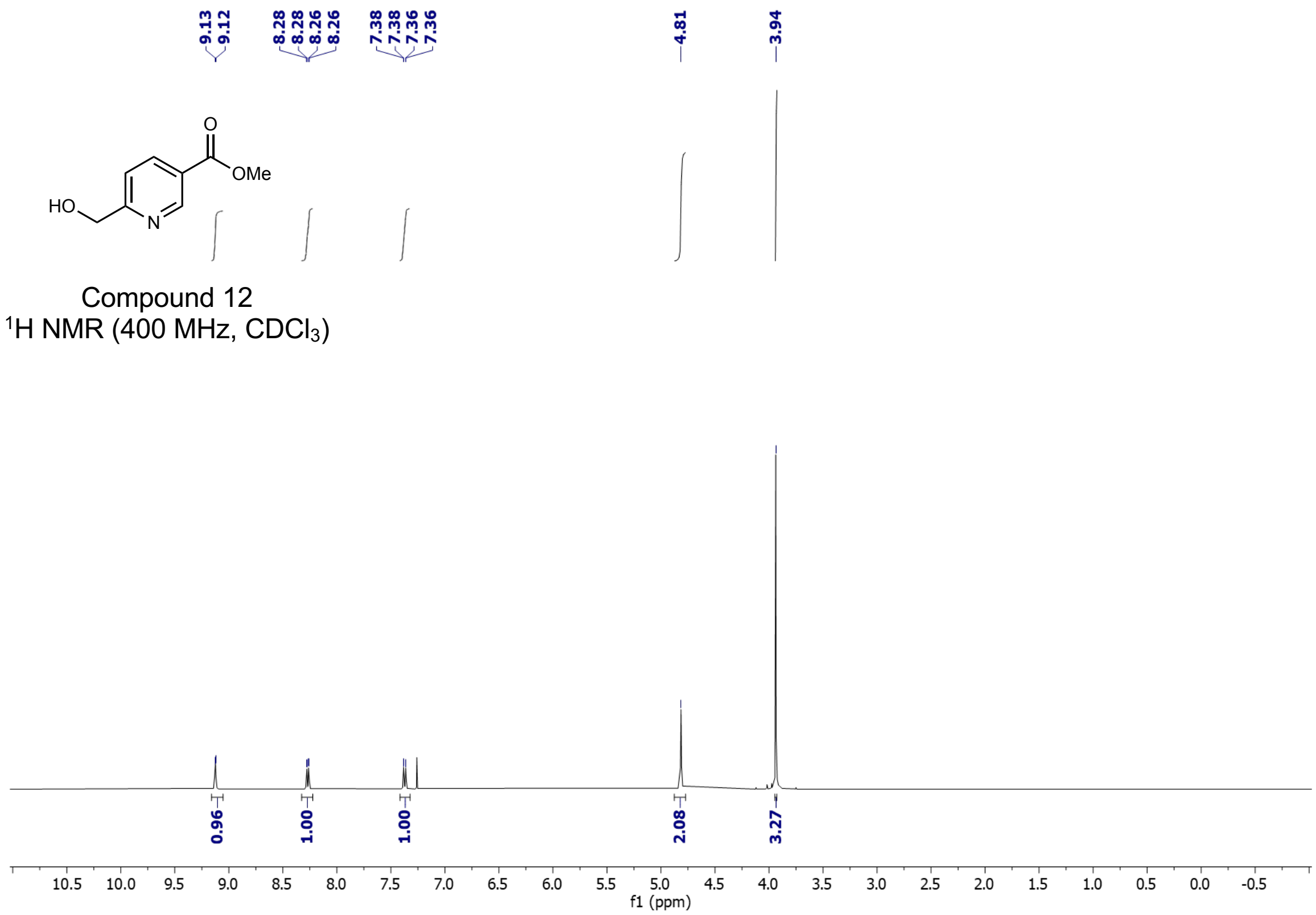

Compound 12
^{1}H NMR (400 MHz, $CDCl_3$)

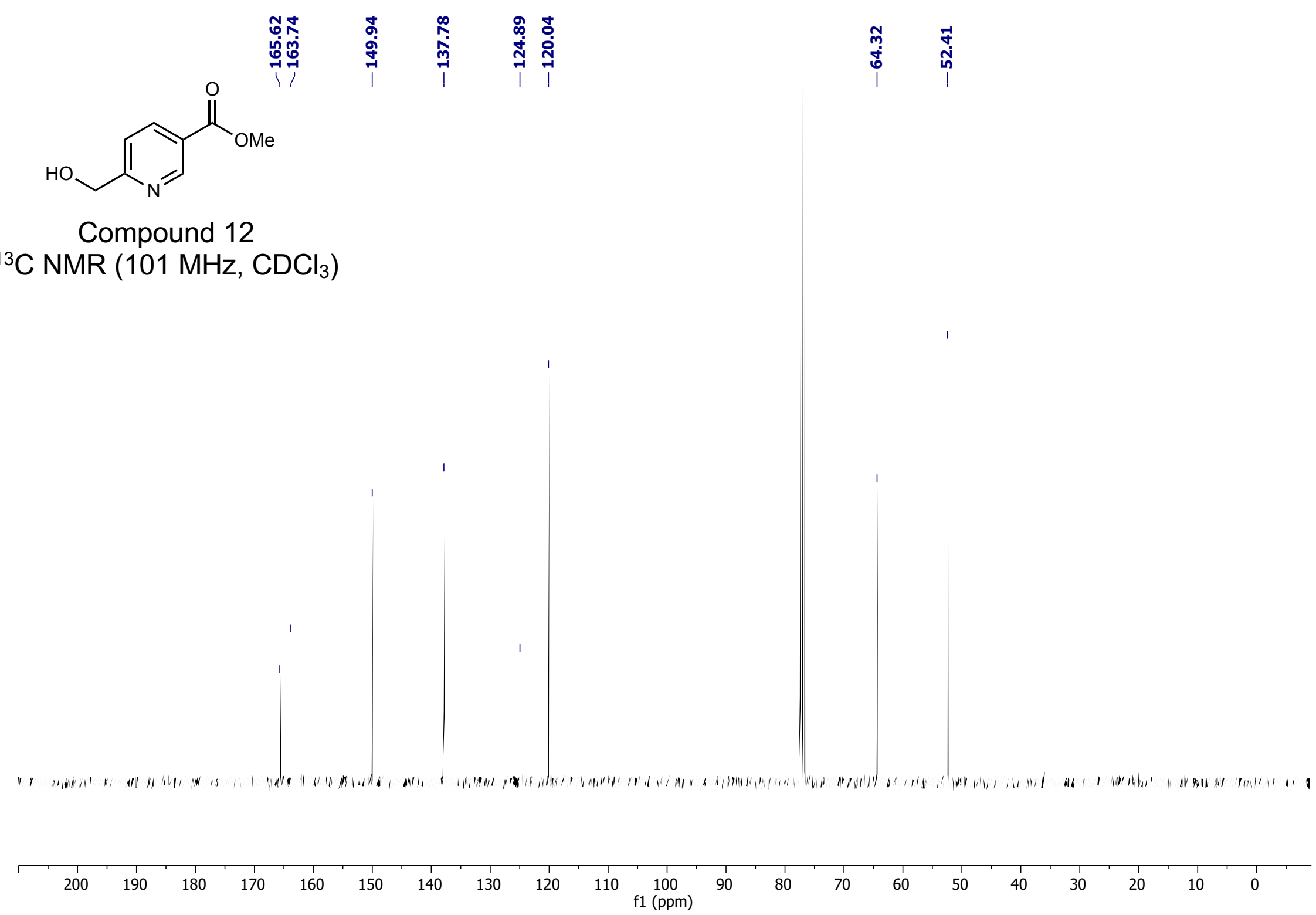

Compound 12
^{13}C NMR (101 MHz, $CDCl_3$)

Compound S4
^{1}H NMR (400 MHz, $CDCl_3$)

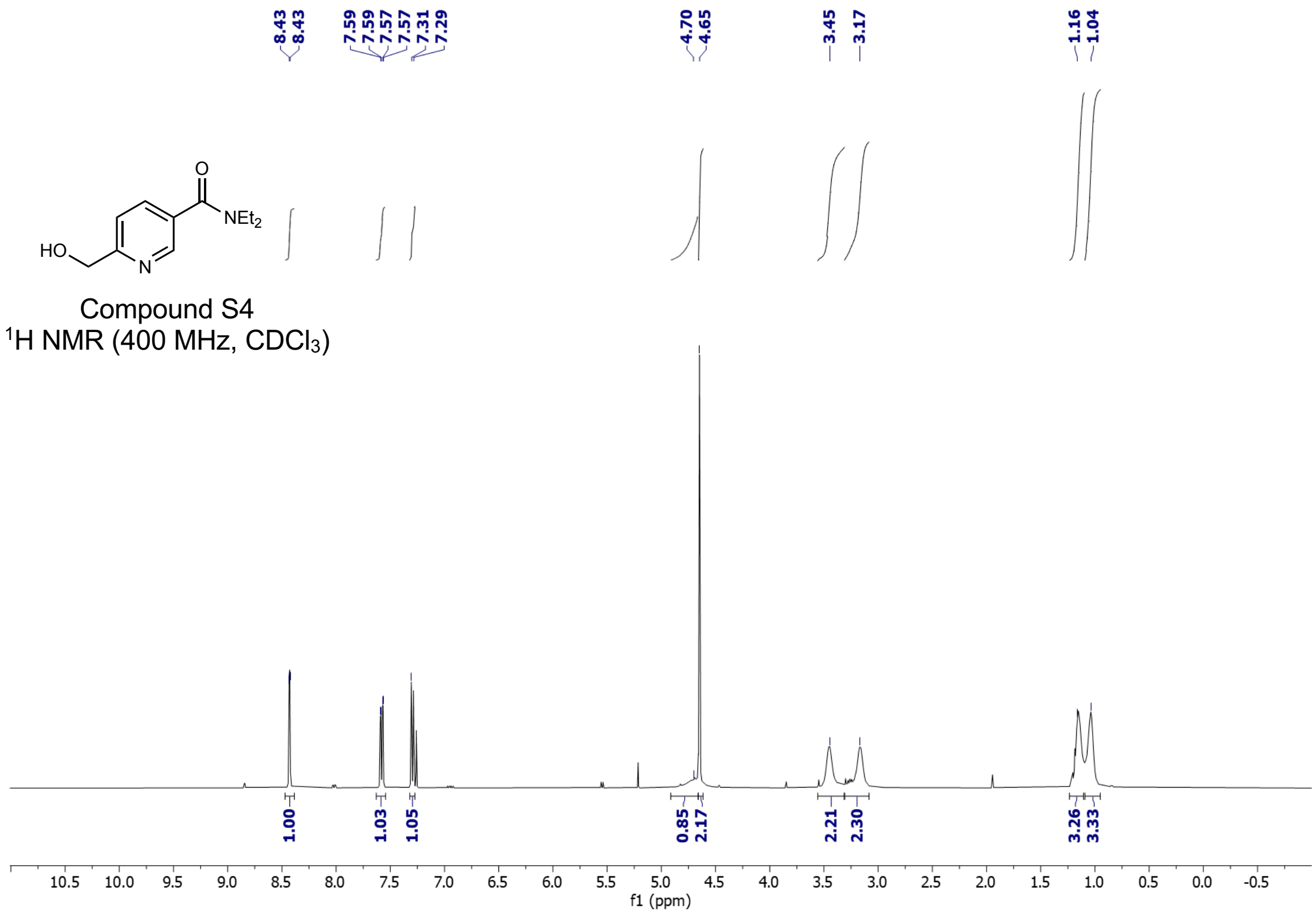

Compound S4
^{13}C NMR (101 MHz, $CDCl_3$)

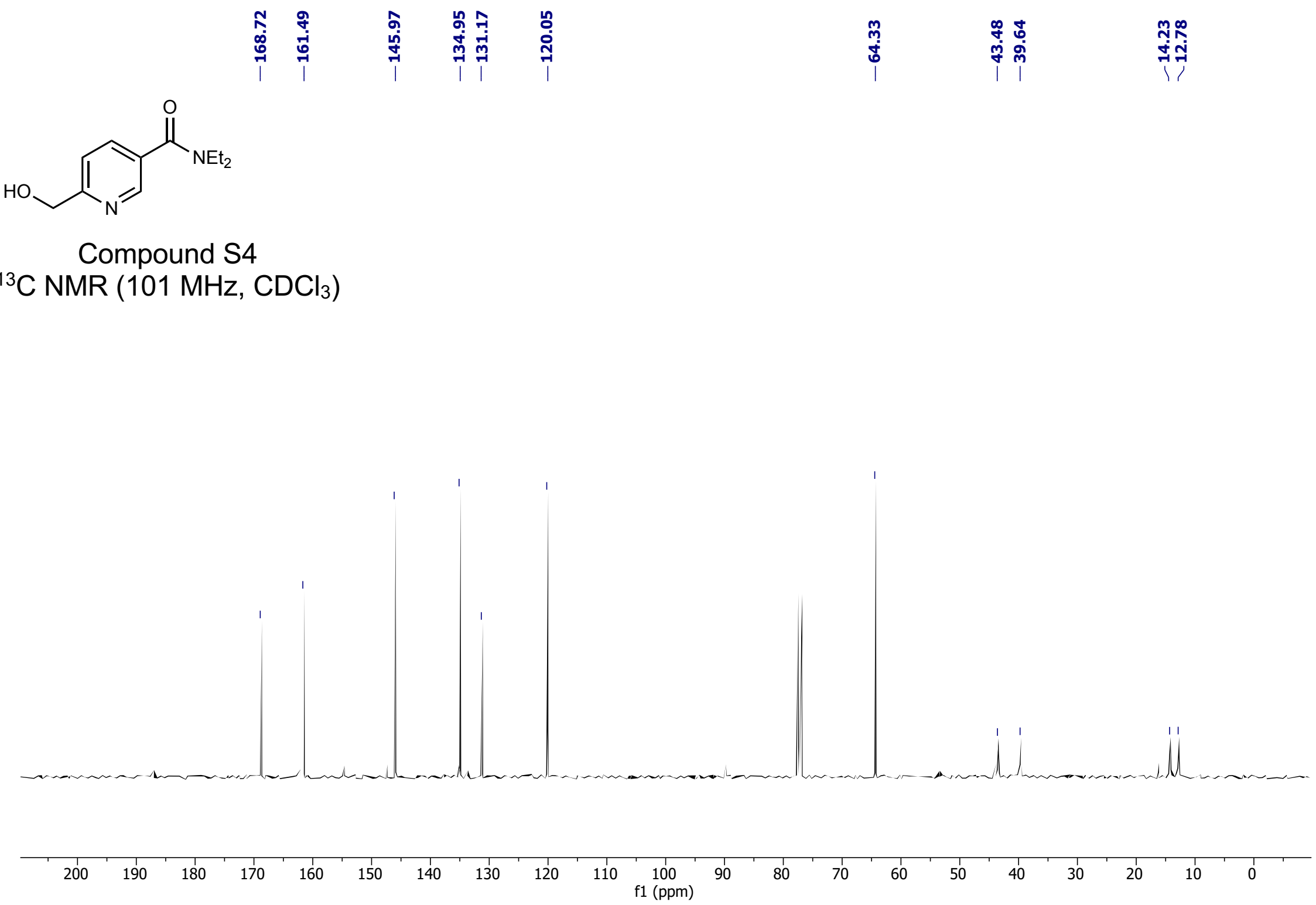

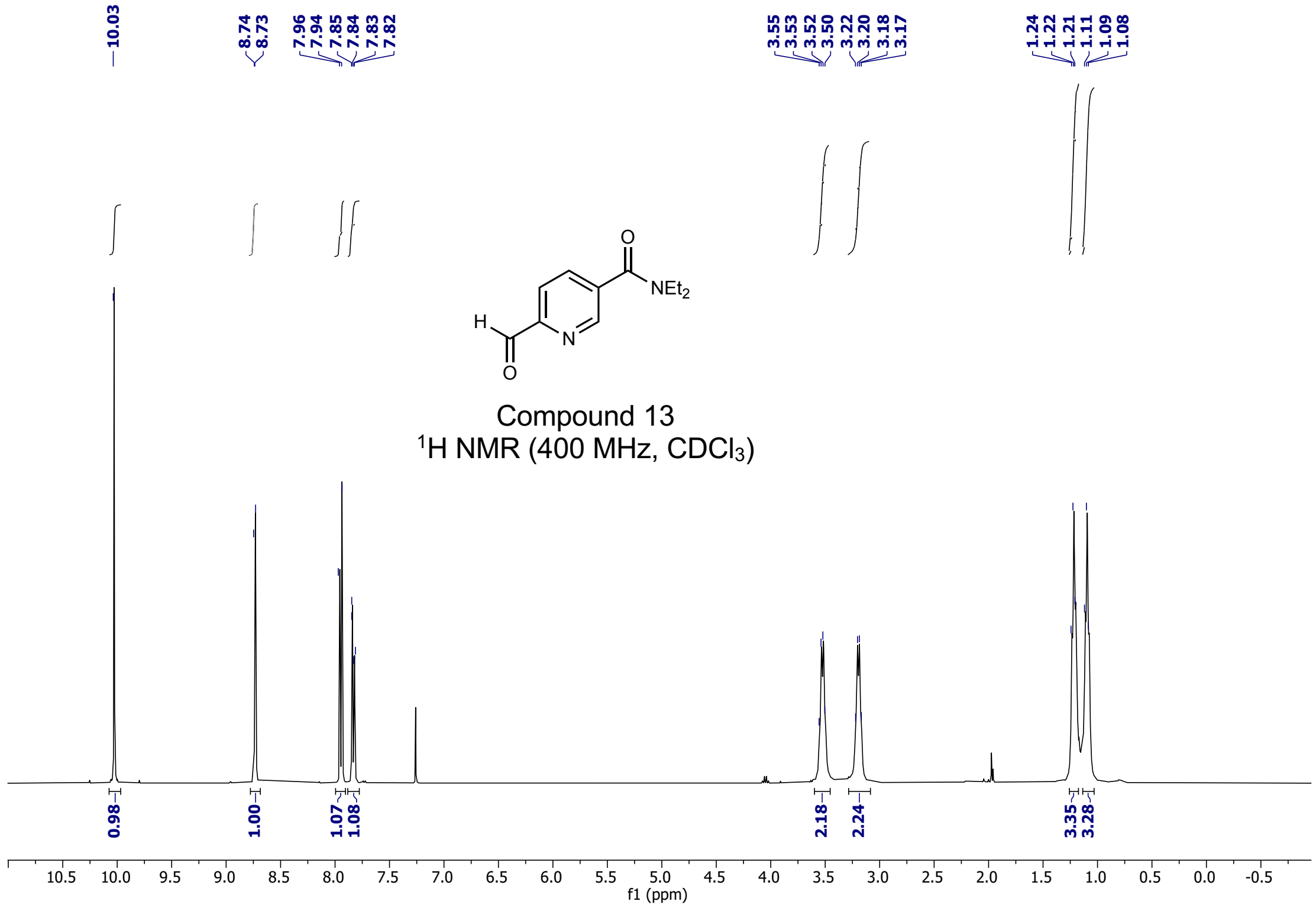
10.03
8.74
8.73
7.96
7.94
7.85
7.84
7.83
7.82
3.55
3.53
3.52
3.50
3.22
3.20
3.18
3.17
1.24
1.22
1.21
1.11
1.09
1.08
NEt2
H
N
O
Compound 13
1H NMR (400 MHz, CDCl3)
0.98
1.00
1.07
1.08
2.18
2.24
3.35
3.28
f1 (ppm)

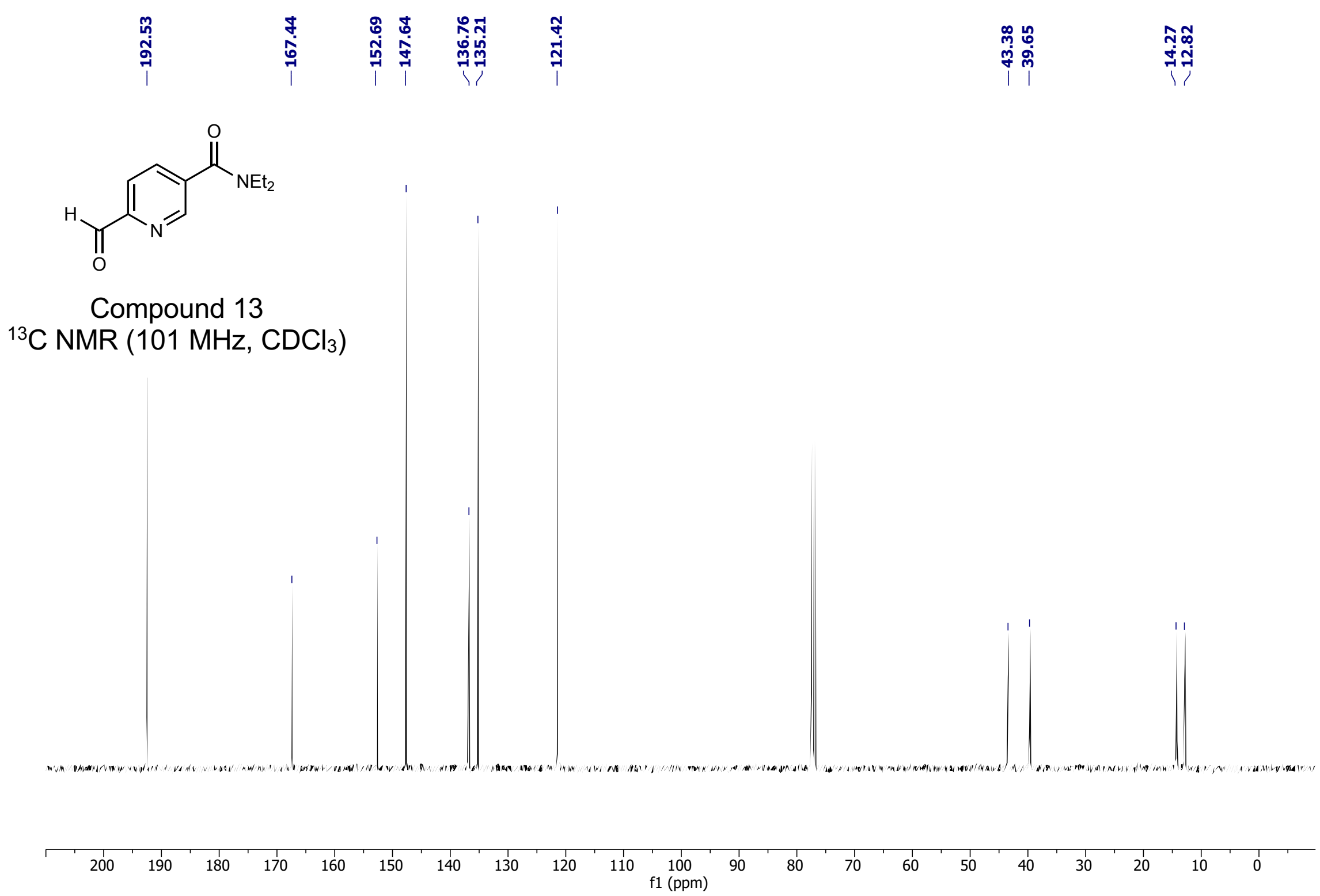
192.53
167.44
152.69
147.64
136.76
135.21
121.42
43.38
39.65
14.27
12.82
NEt2
H
N
O
Compound 13
13C NMR (101 MHz, CDCl3)
f1 (ppm)

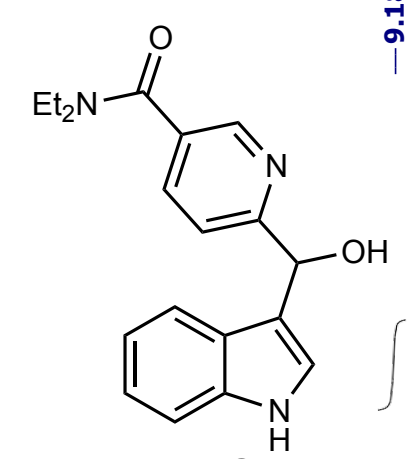

Compound S5

^{1}H NMR (400 MHz, $CDCl_3$)

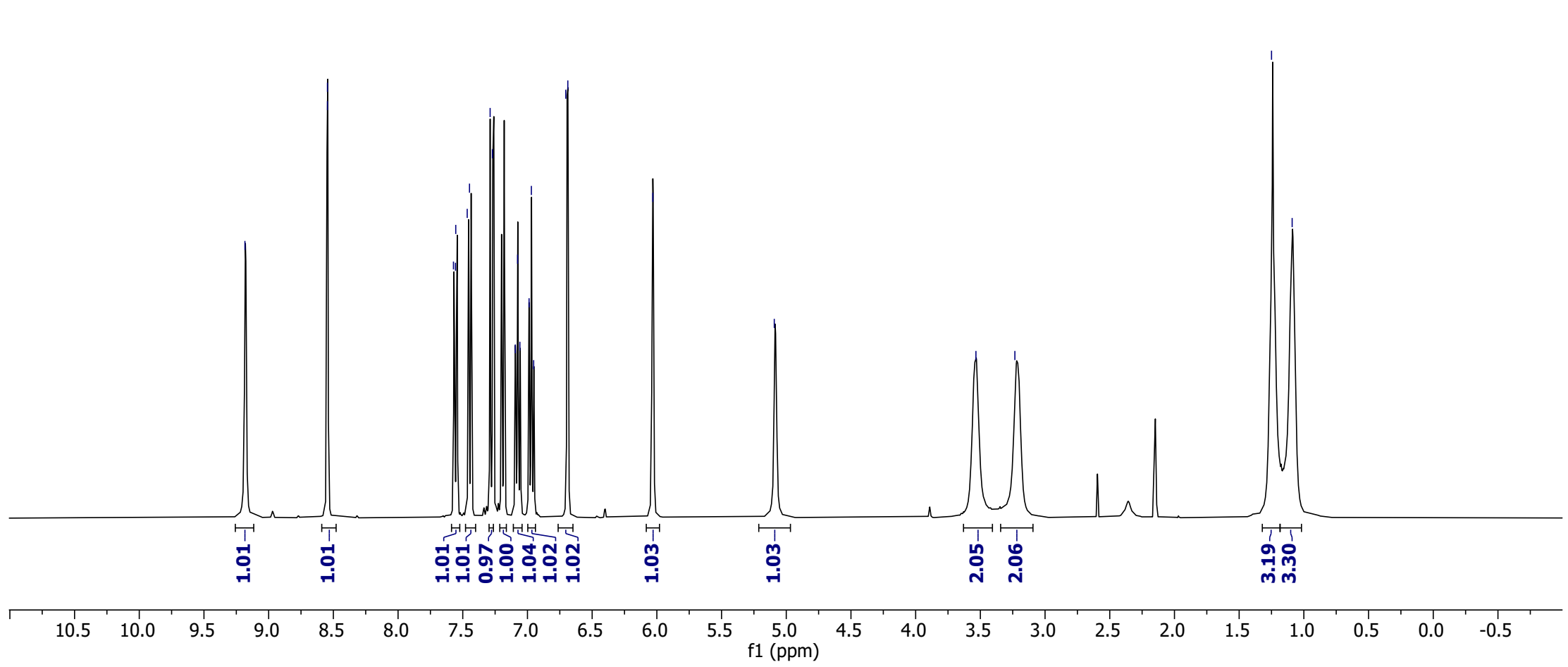

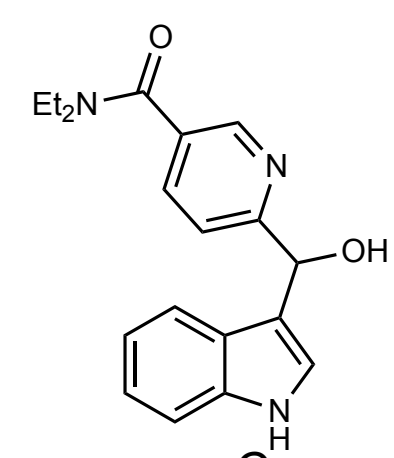

Compound 13

^{13}C NMR (101 MHz, $CDCl_3$)

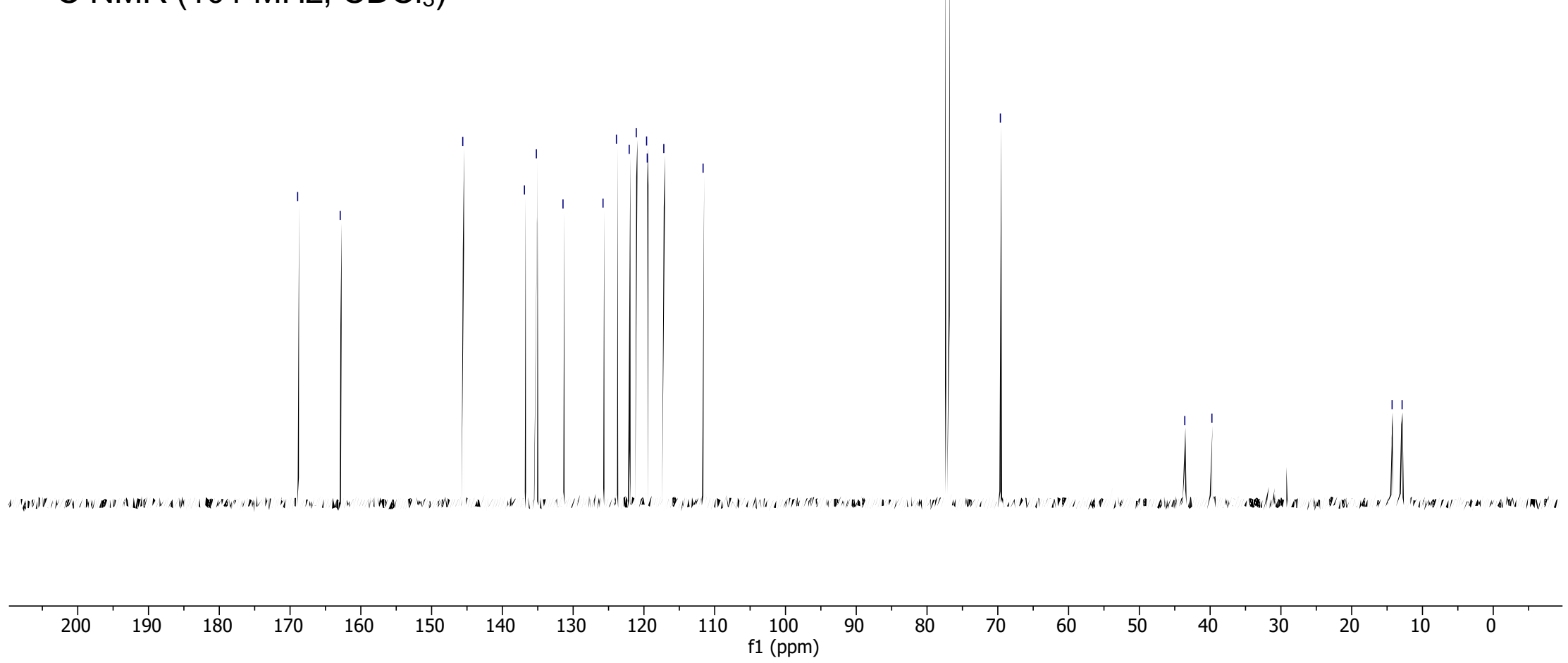

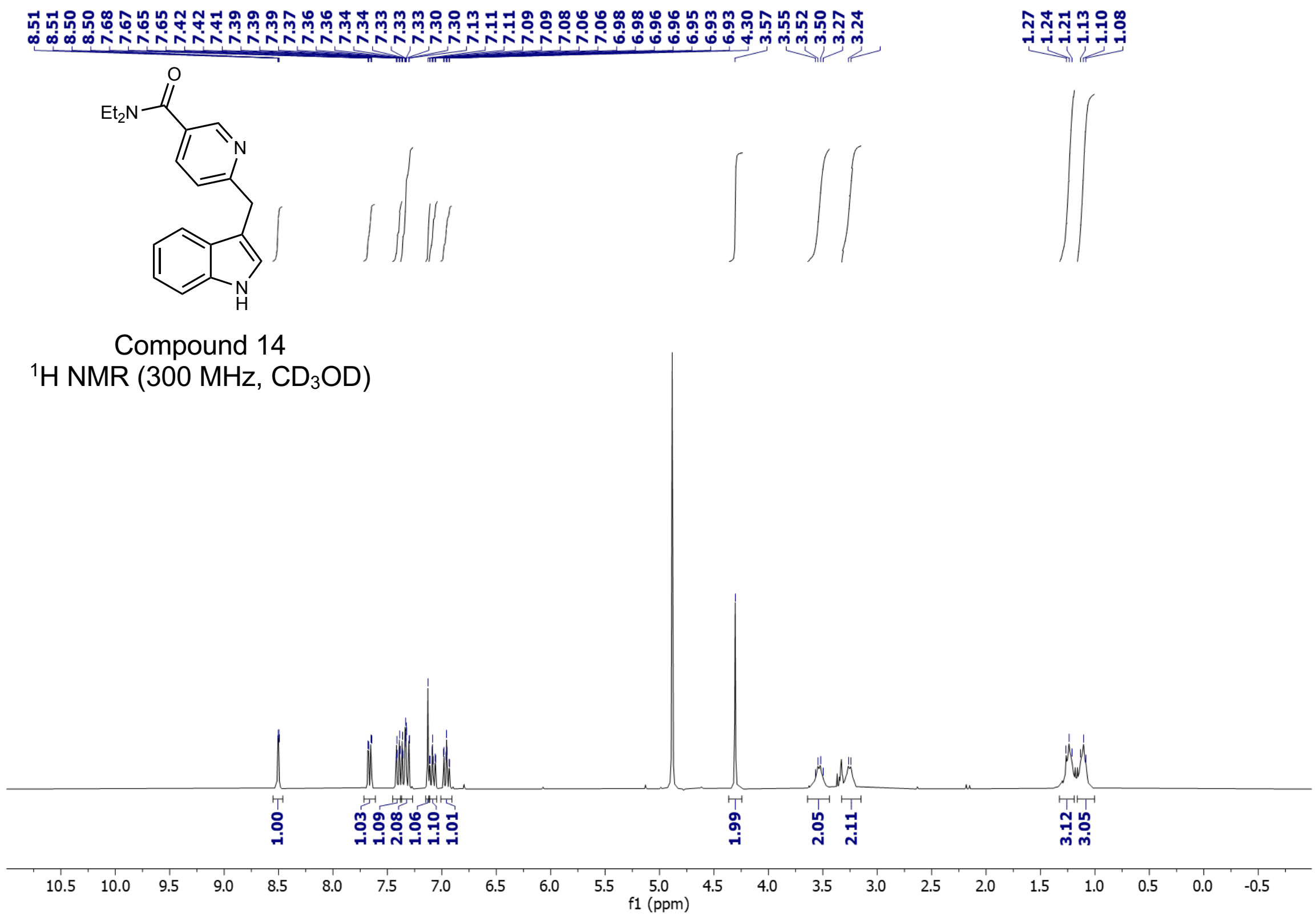

Compound 14
^{1}H NMR (300 MHz, CD_3OD)

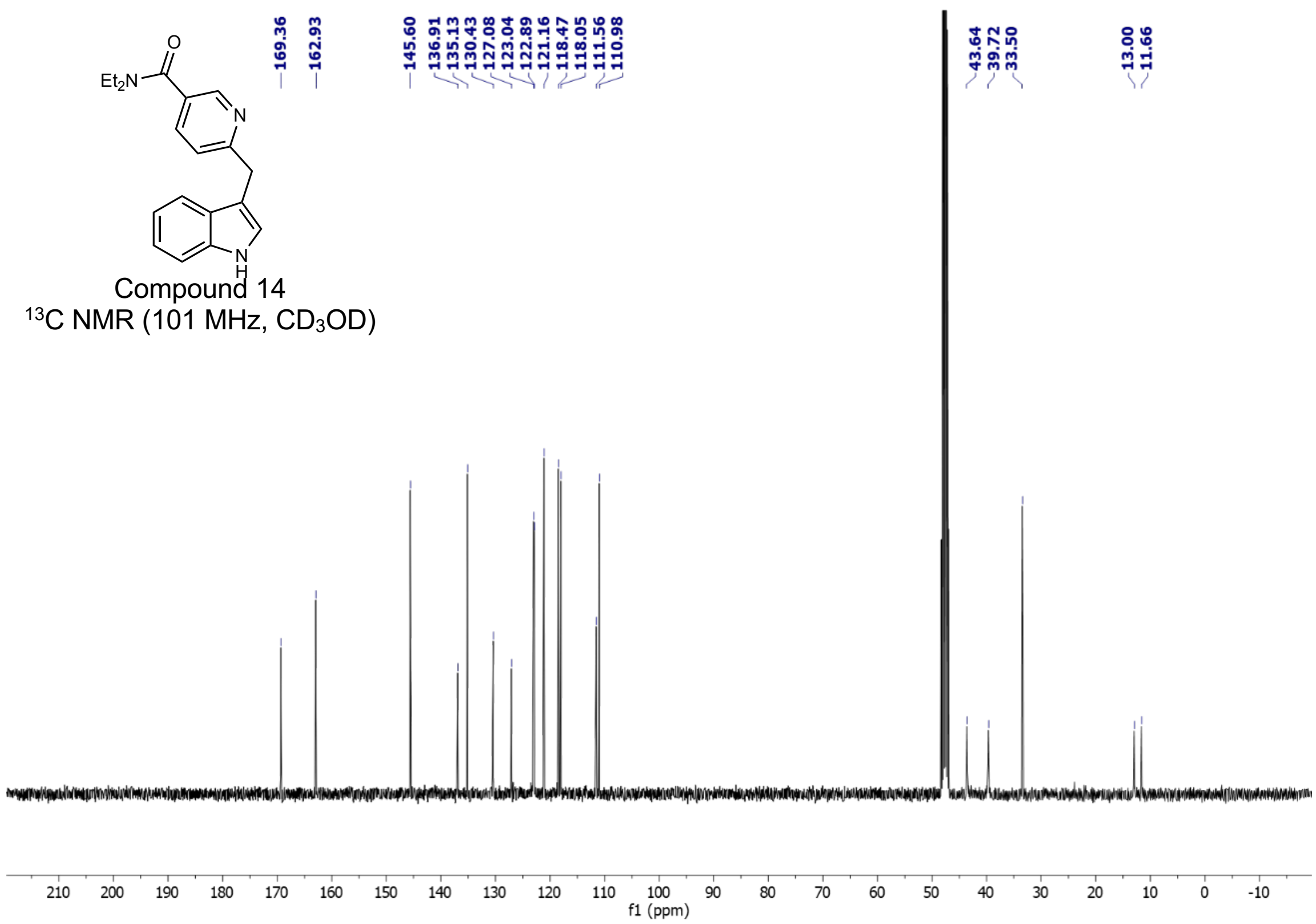

Compound 14
^{13}C NMR (101 MHz, CD_3OD)

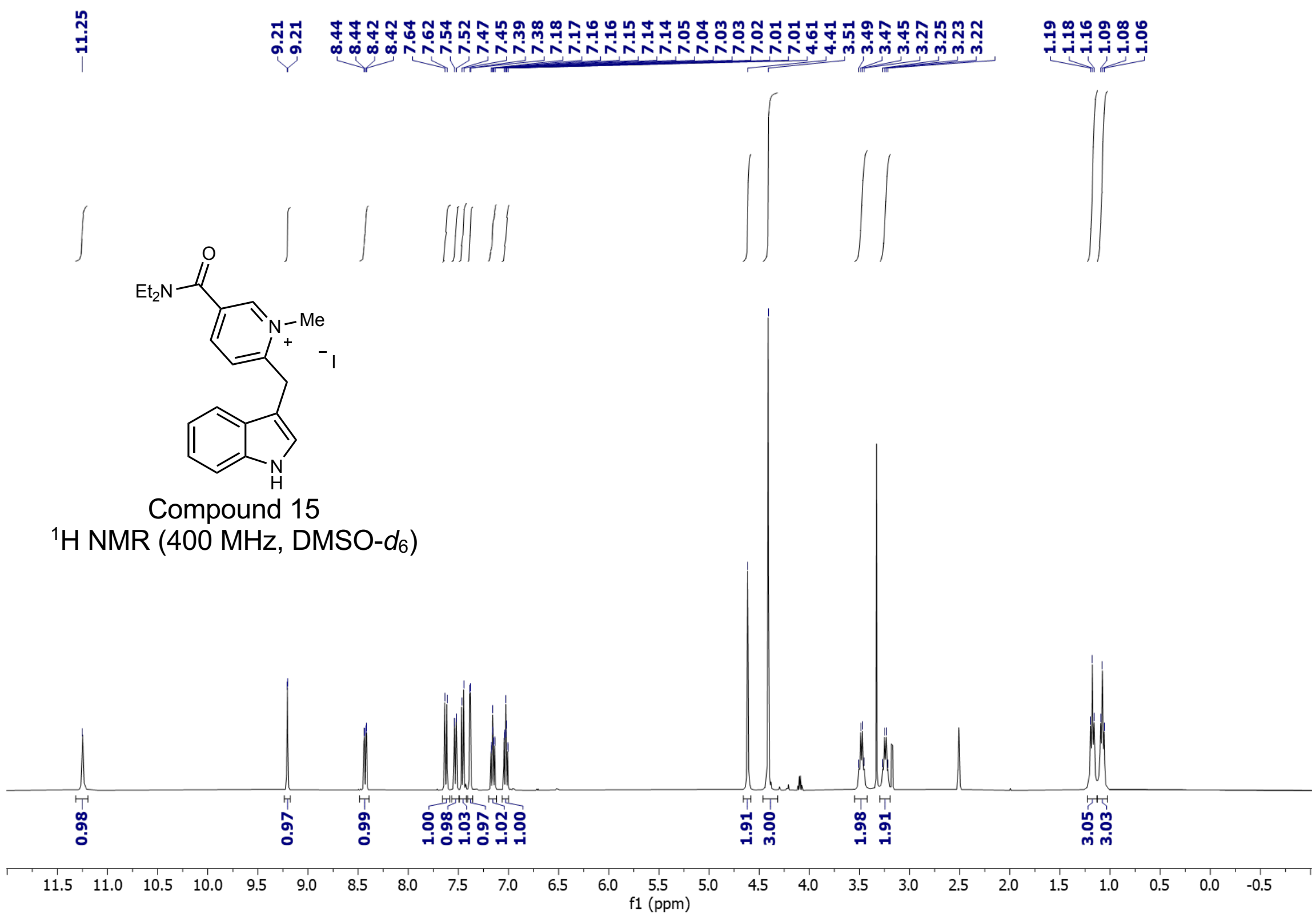

Compound 15
^{1}H NMR (400 MHz, DMSO-d_6)

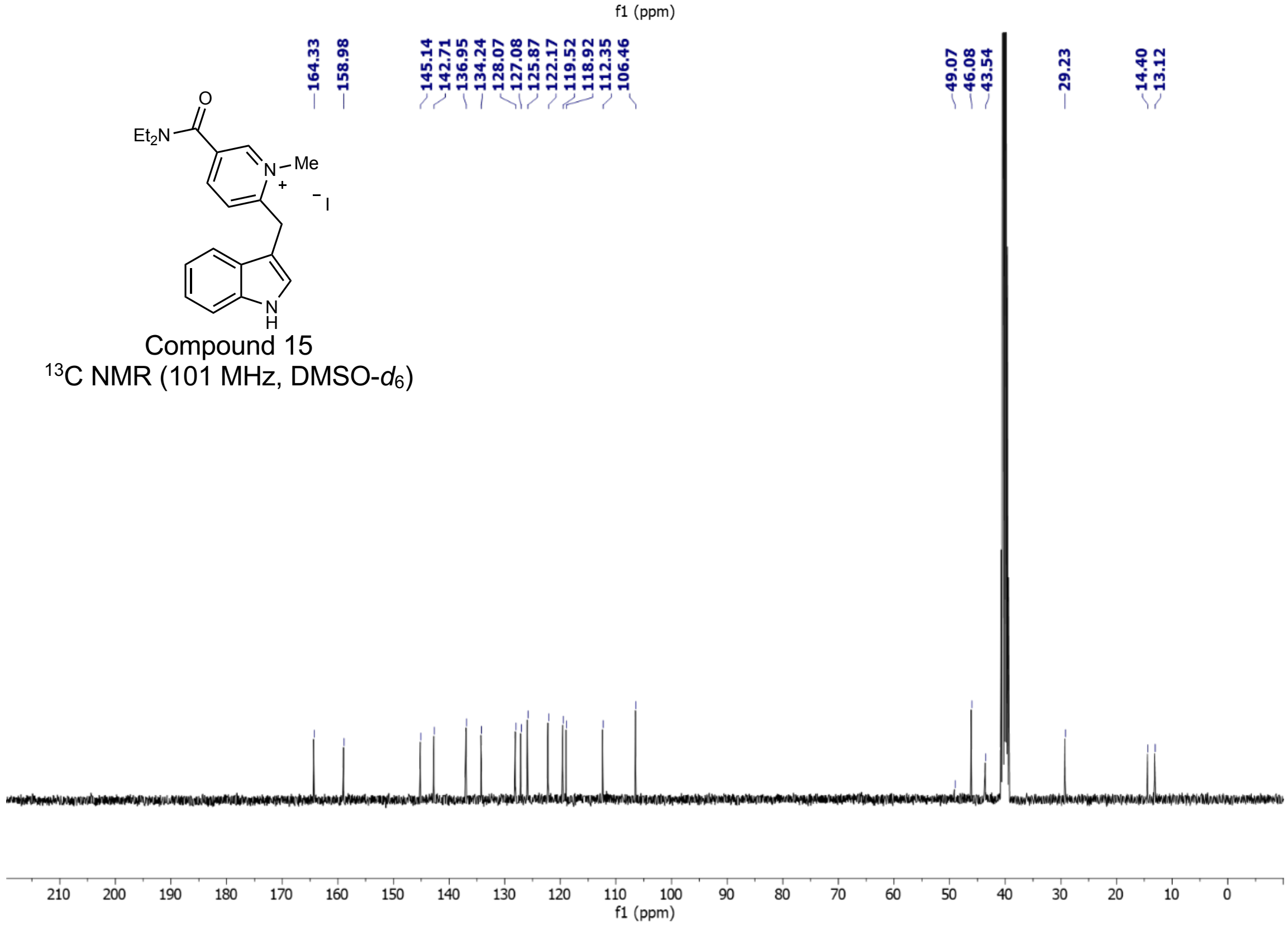

Compound 15
^{13}C NMR (101 MHz, DMSO-d_6)

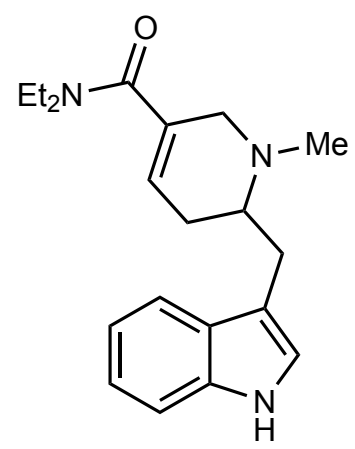

UCD0121 freebase

^{1}H NMR (400 MHz, CD_3OD)

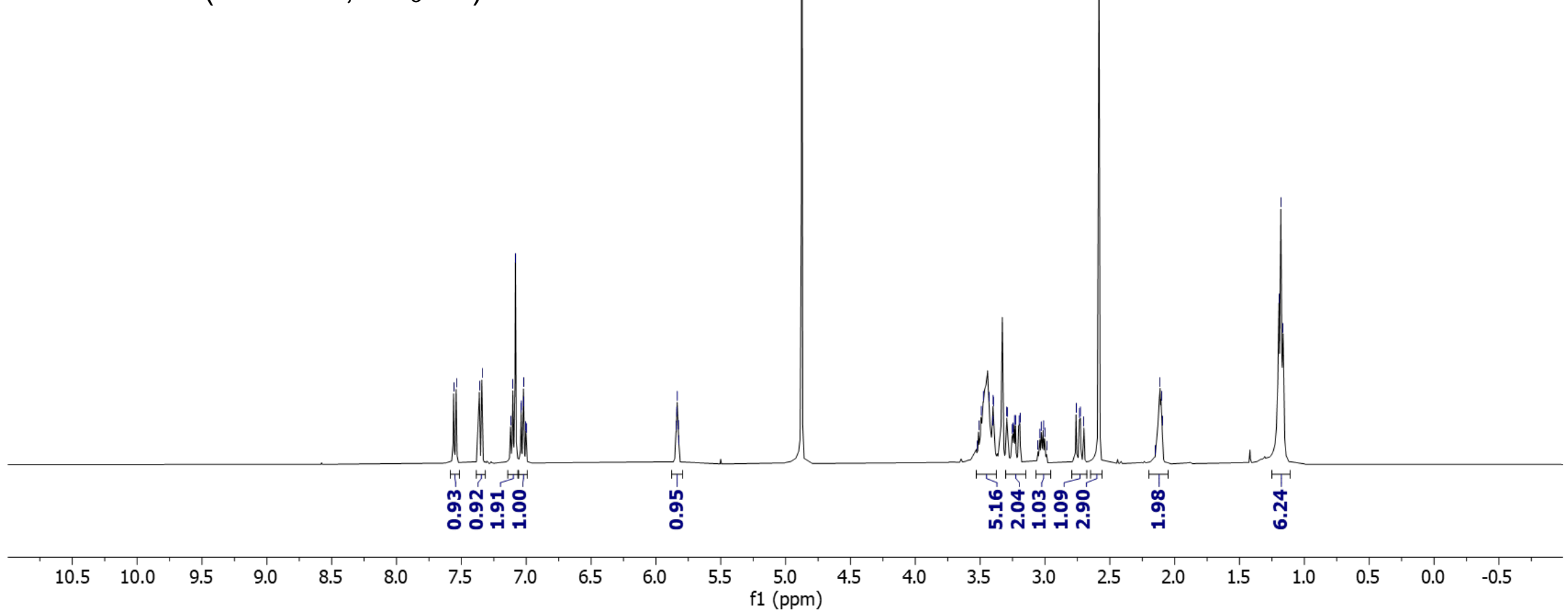

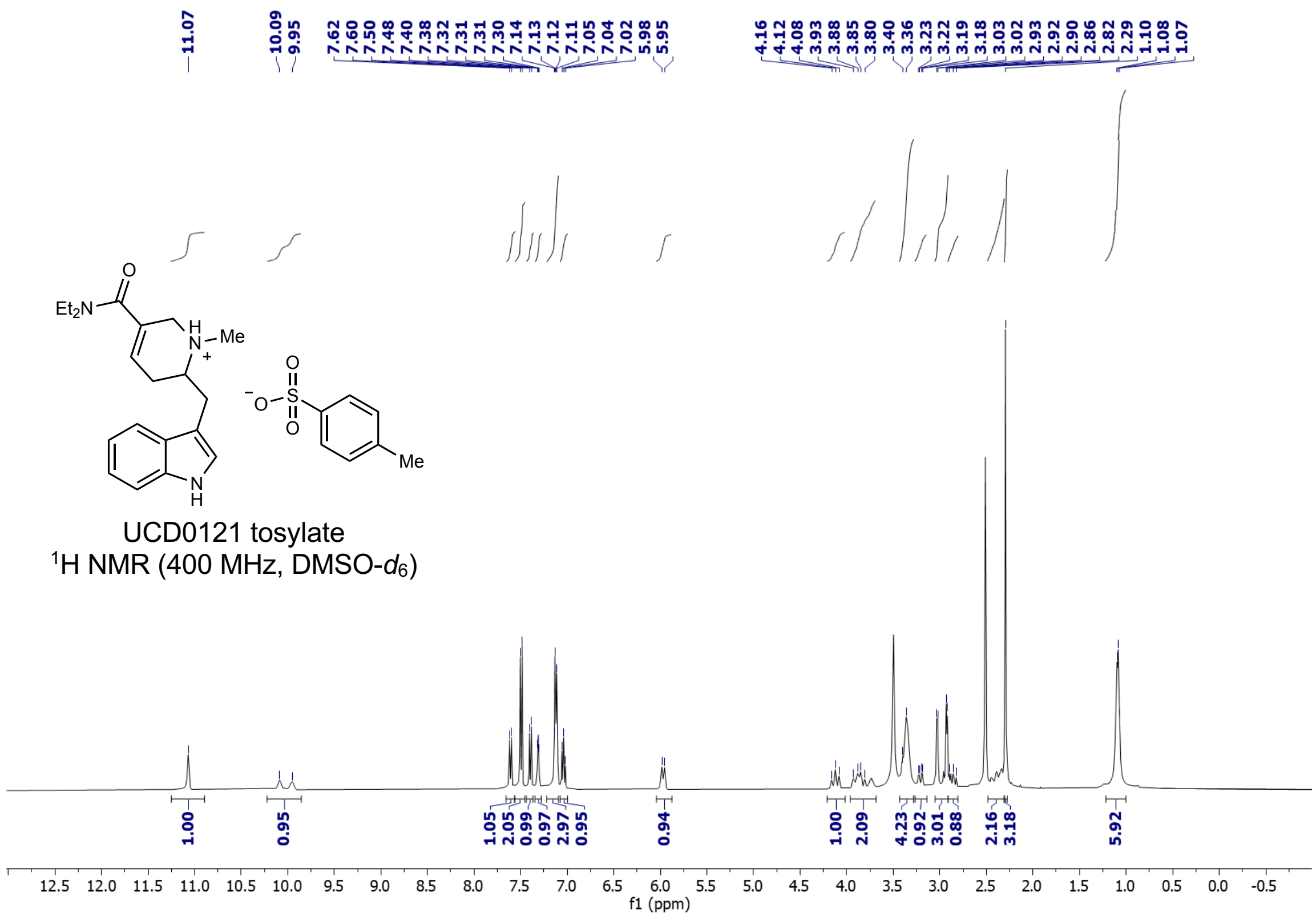
UCD0121 tosylate
1H NMR (400 MHz, DMSO-d6)
f1 (ppm)

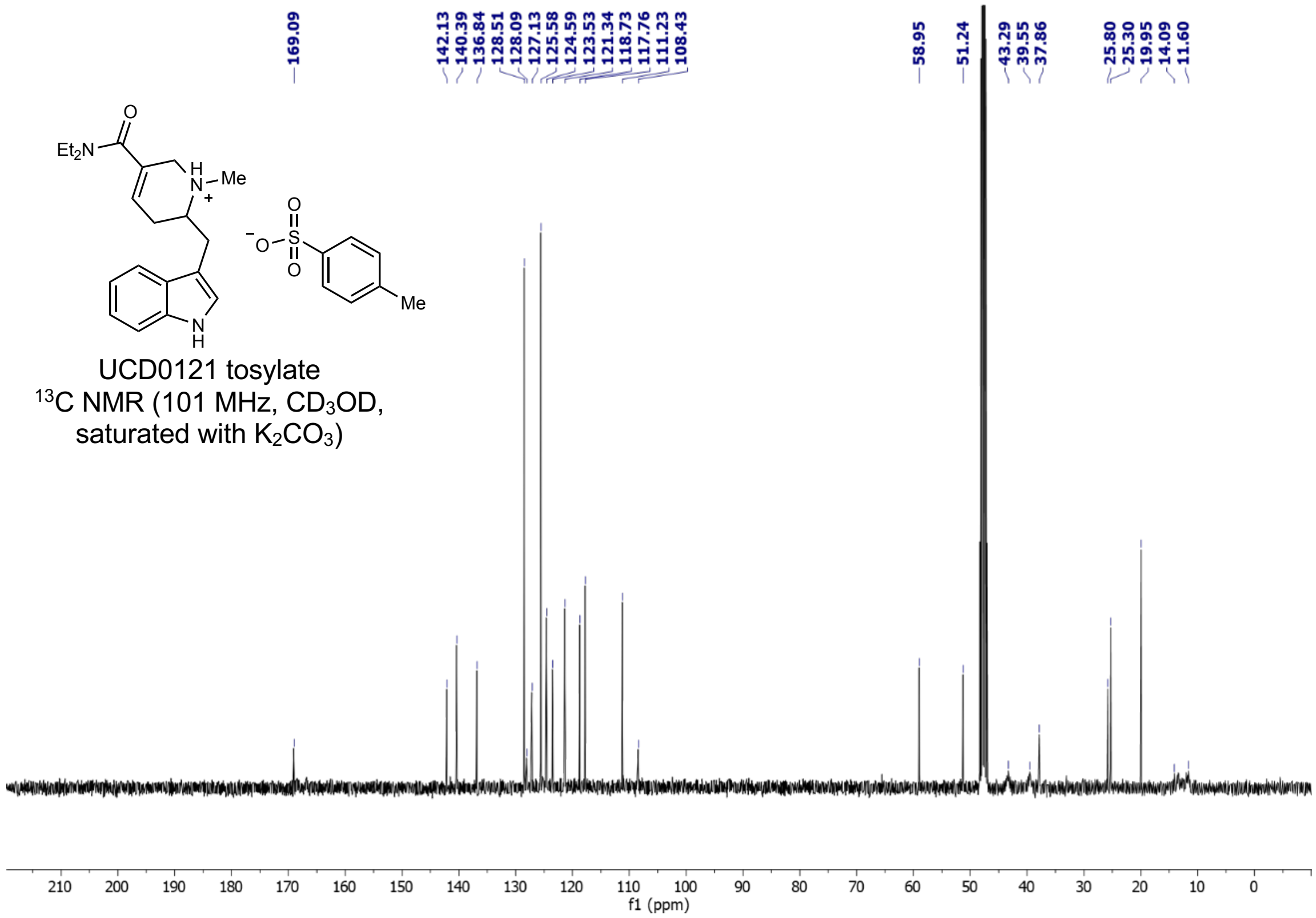
UCD0121 tosylate
13C NMR (101 MHz, CD3OD, saturated with K2CO3)
f1 (ppm)

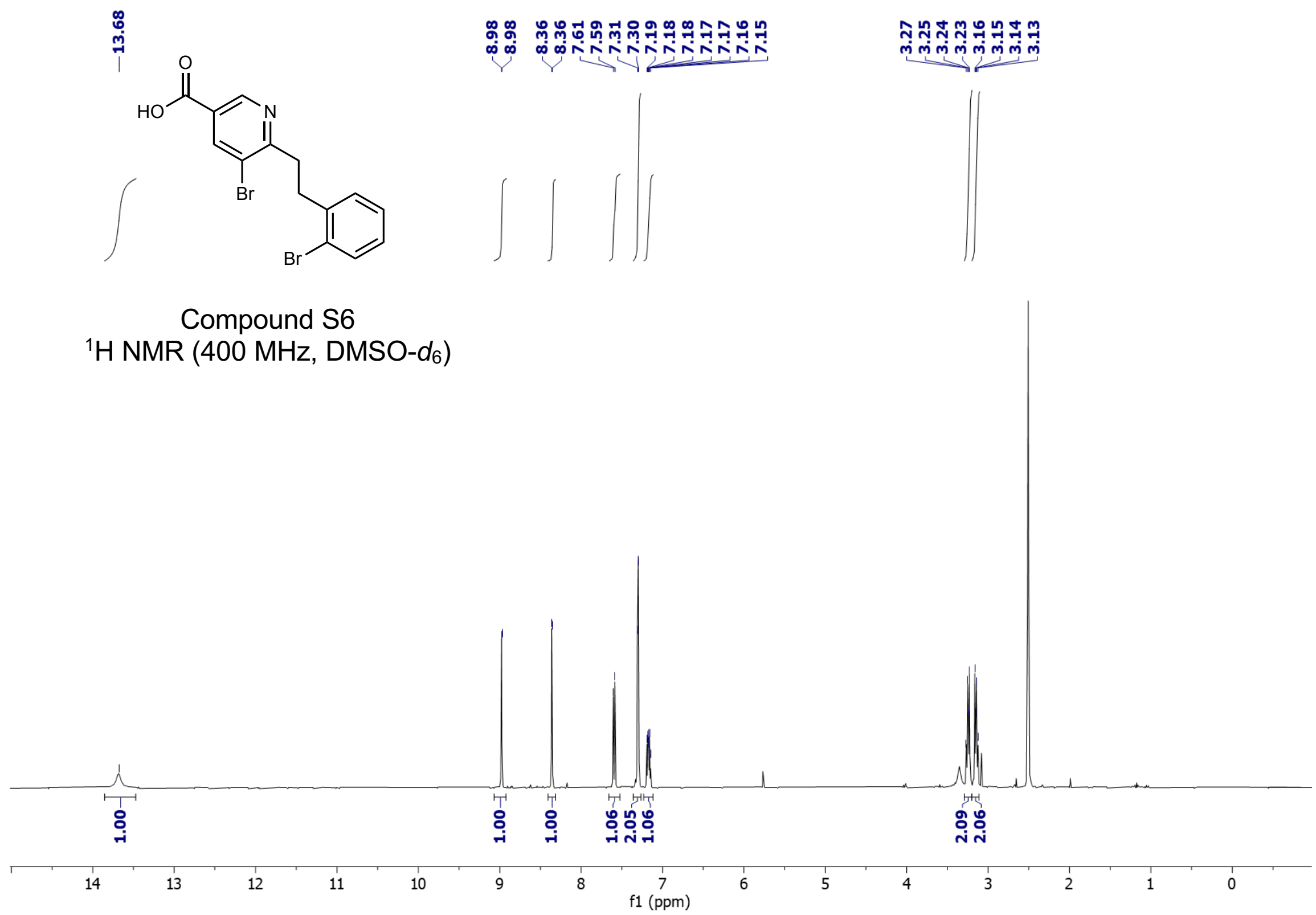

Compound S6
^{1}H NMR (400 MHz, DMSO-d_6)

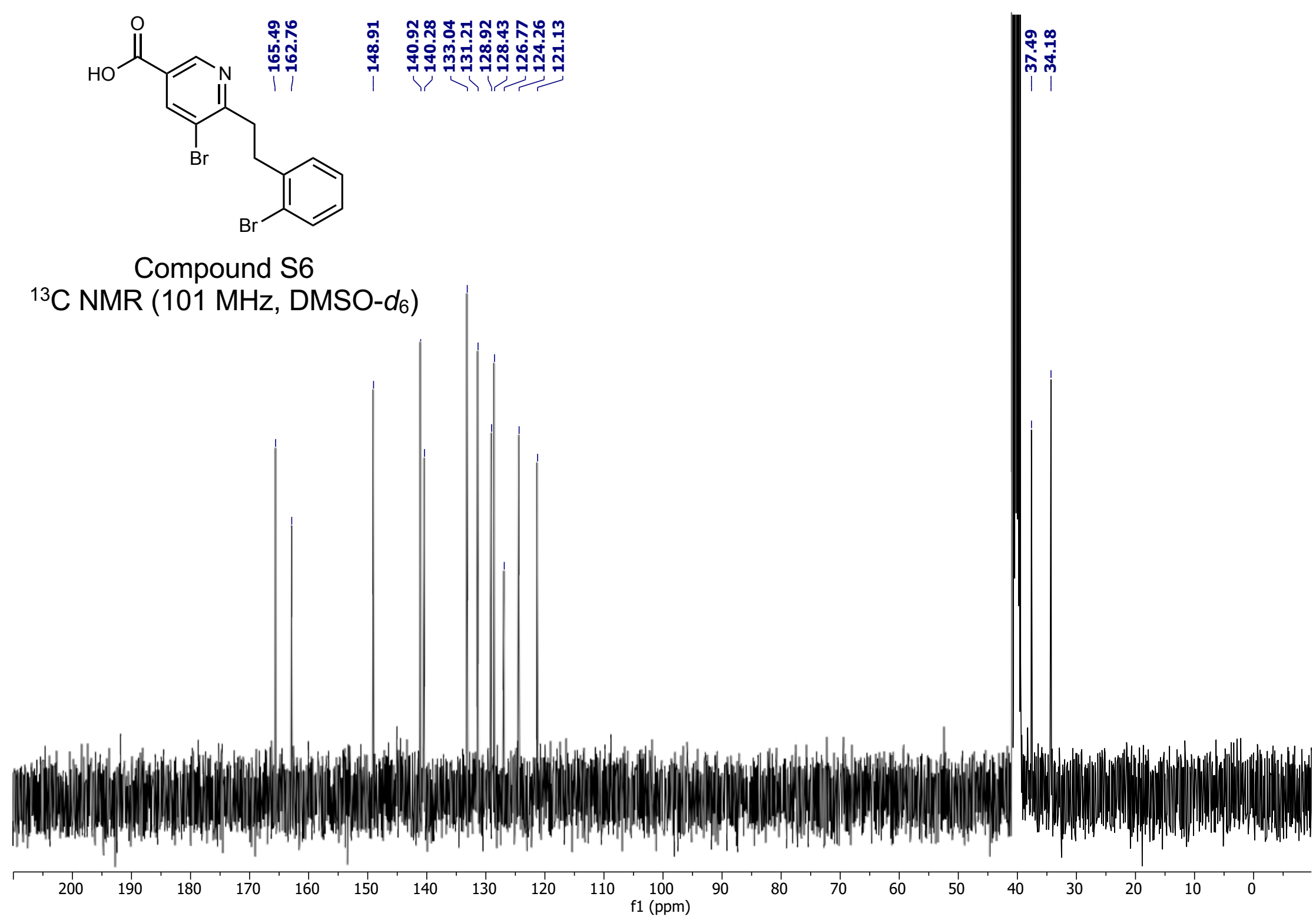

Compound S6
^{13}C NMR (101 MHz, DMSO-d_6)

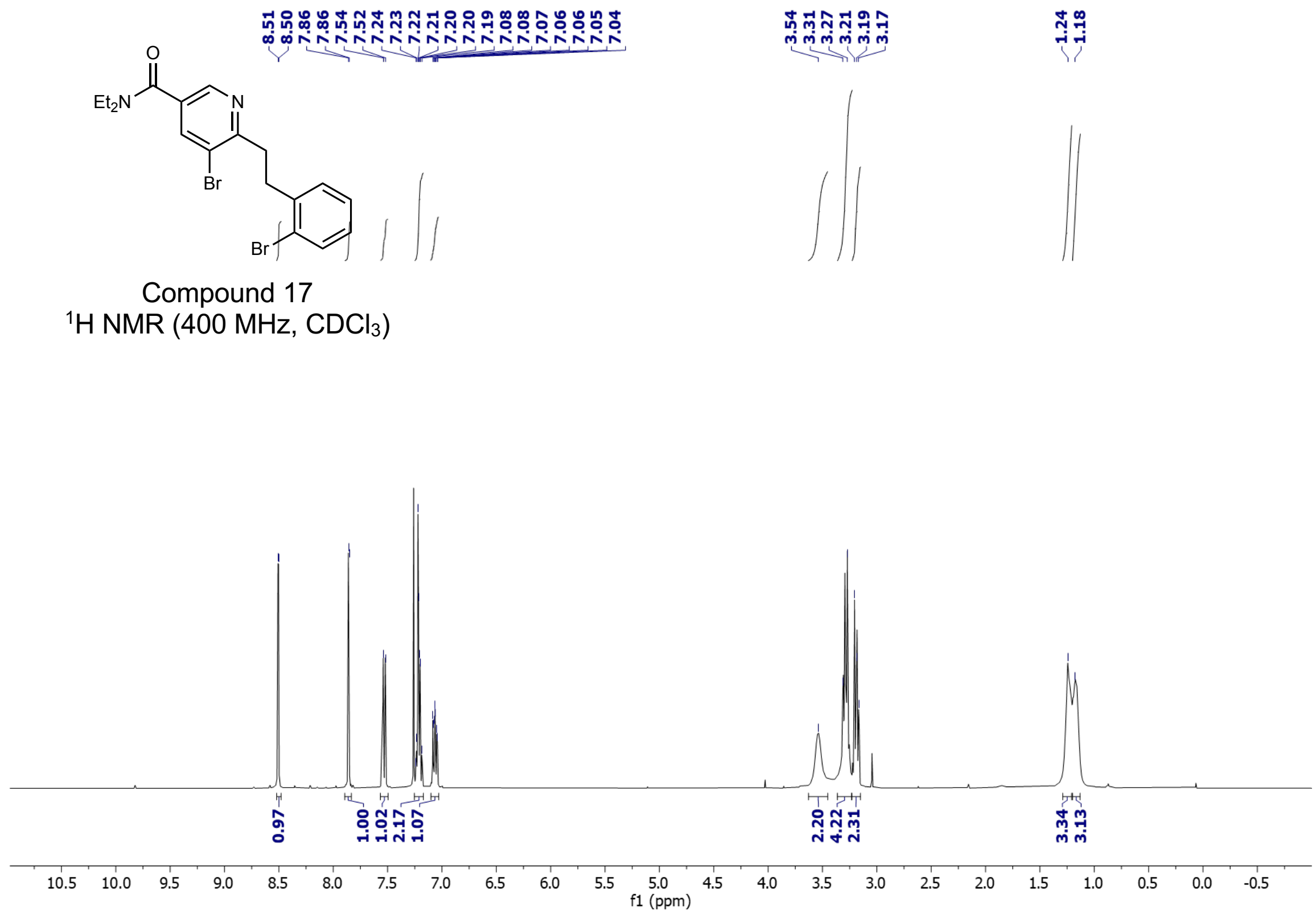

Compound 17
^{1}H NMR (400 MHz, $CDCl_3$)

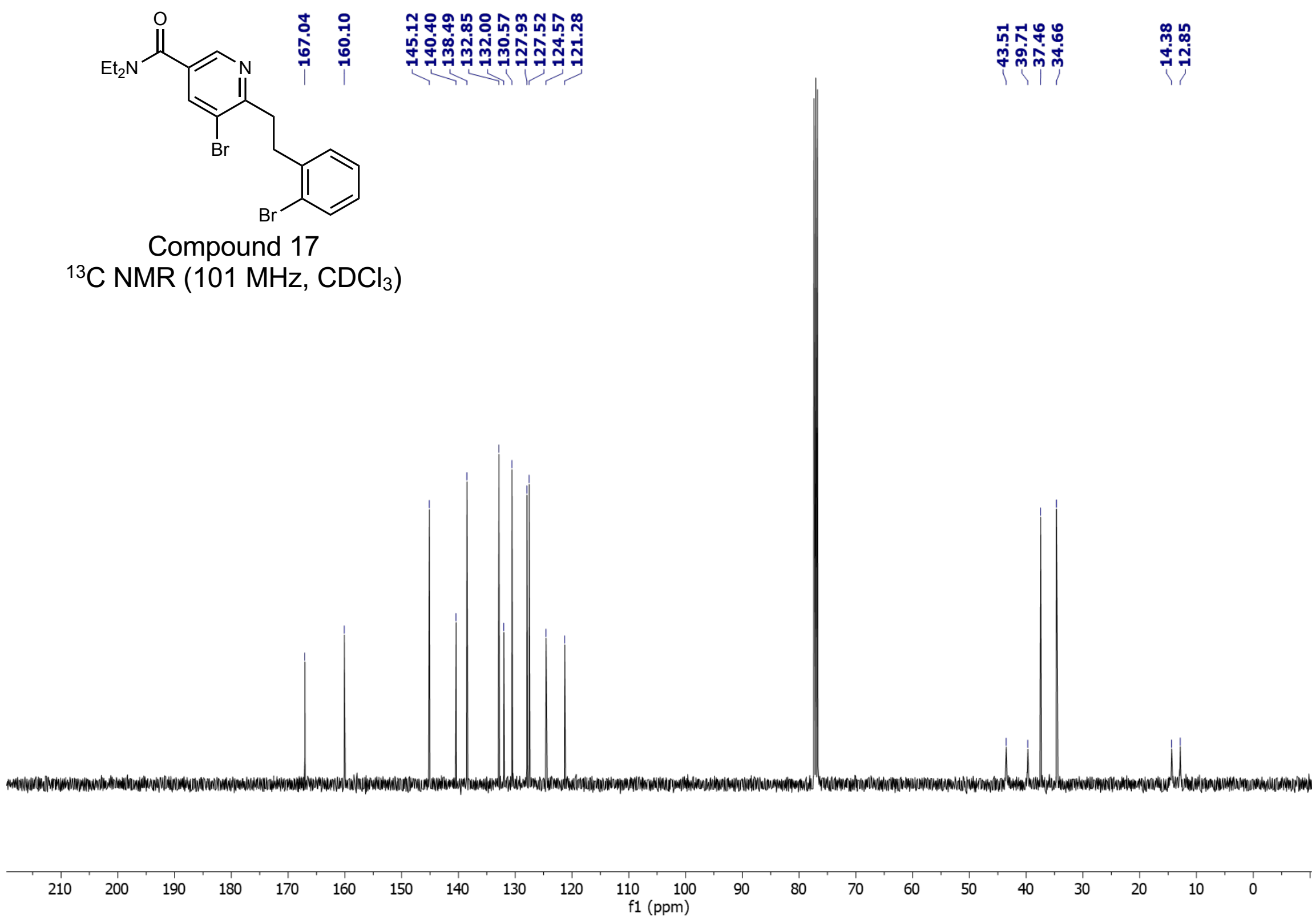

Compound 17
^{13}C NMR (101 MHz, $CDCl_3$)

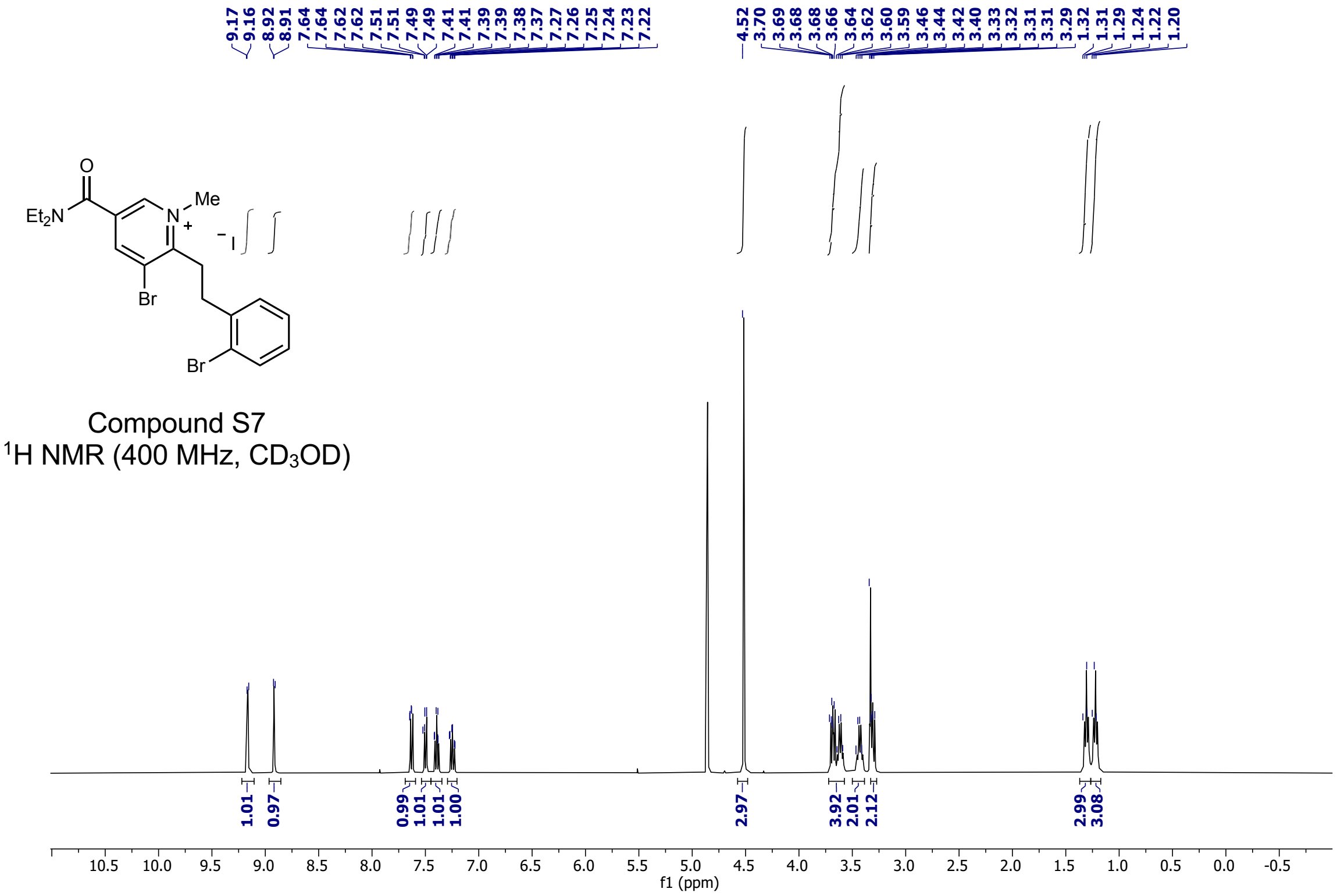

Compound S7
^1H NMR (400 MHz, CD_3OD)

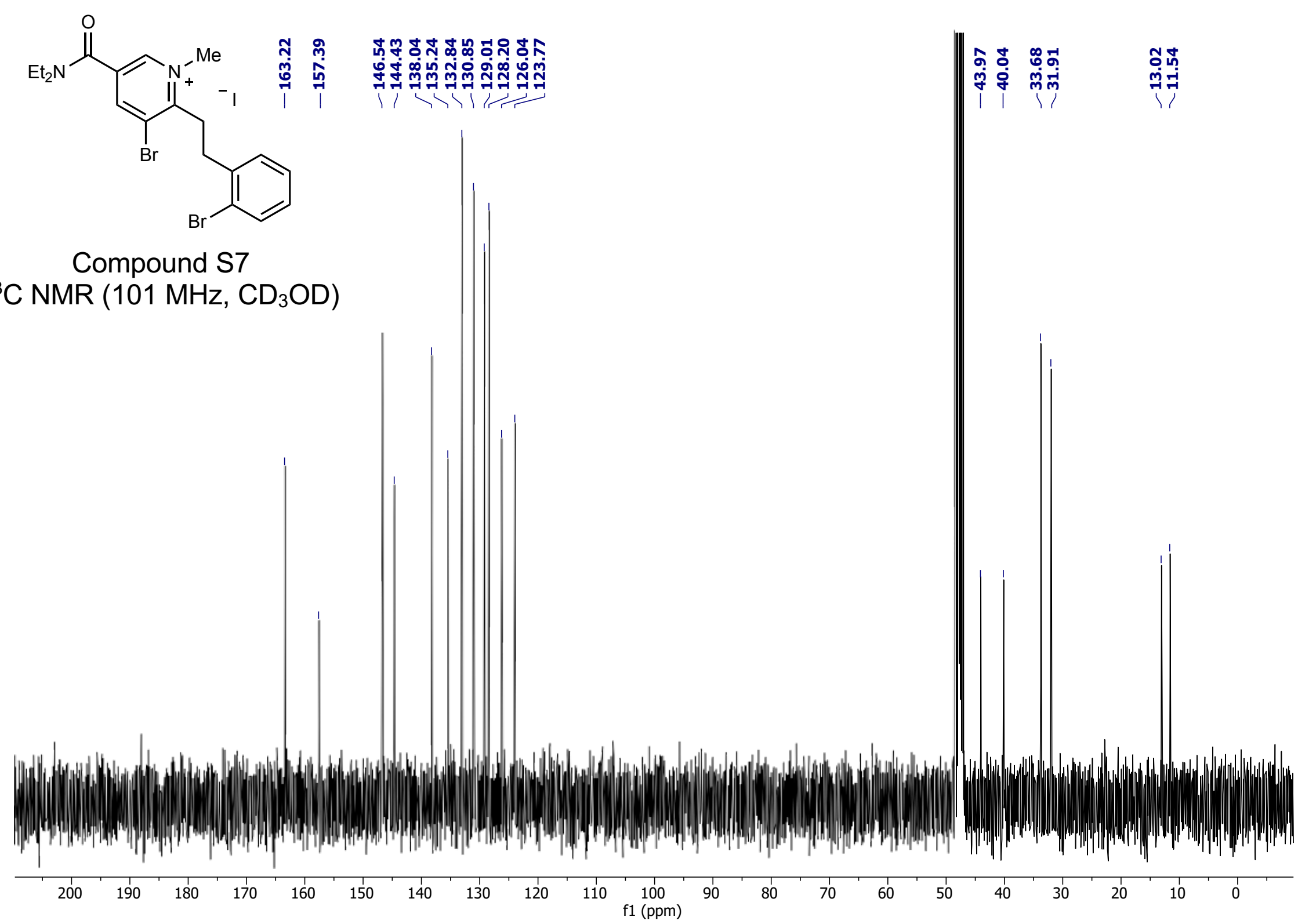

Compound S7
^{13}C NMR (101 MHz, CD_3OD)

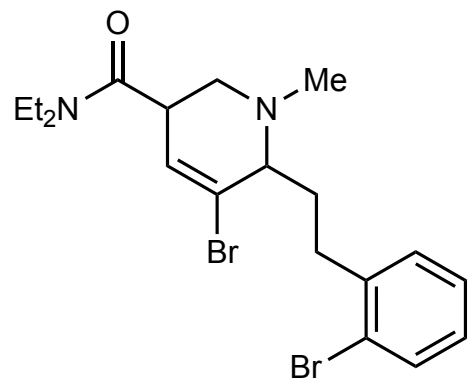

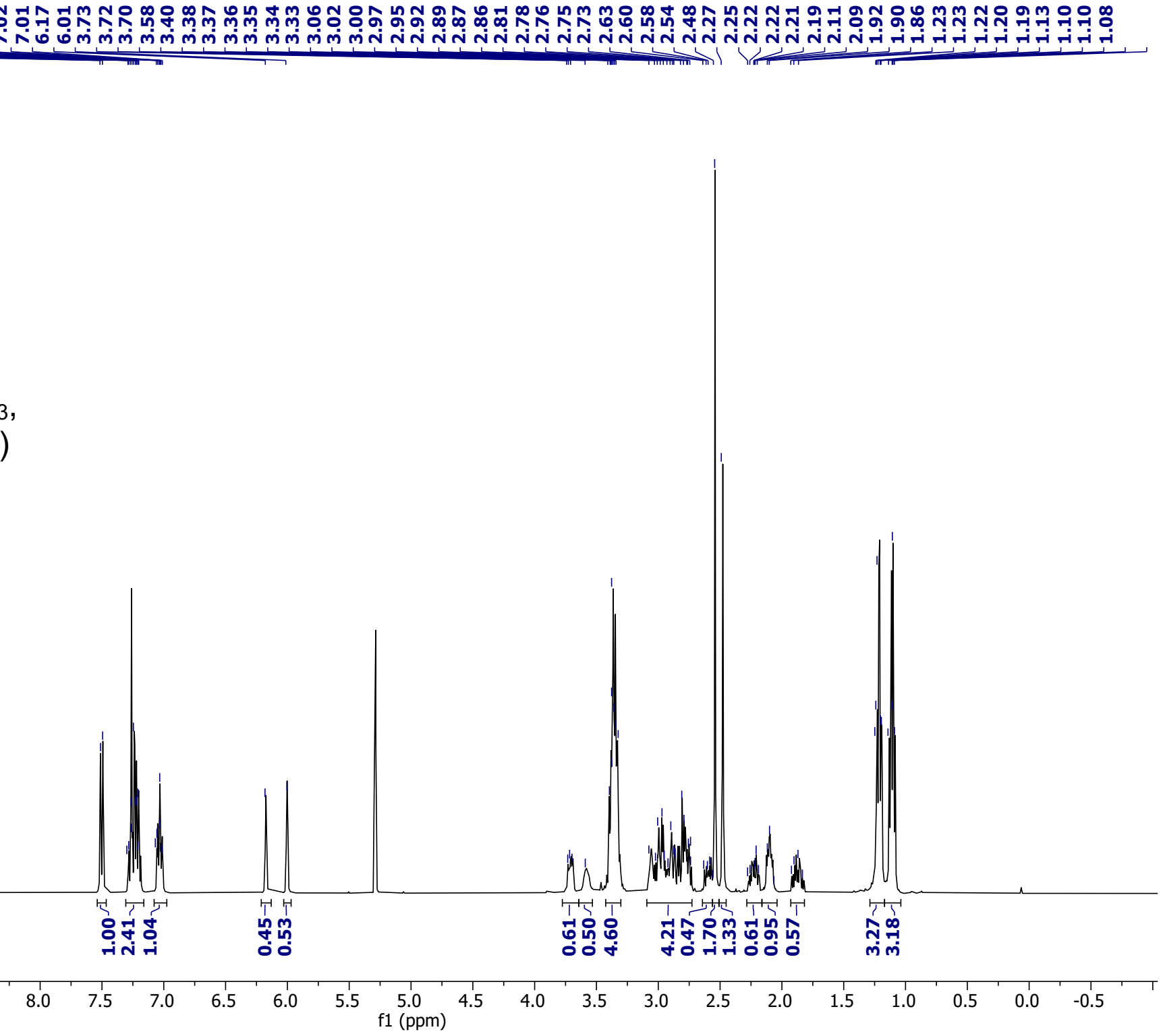

Compound 18
^{1}H NMR (400 MHz, $CDCl_3$, mixture of diastereomers)

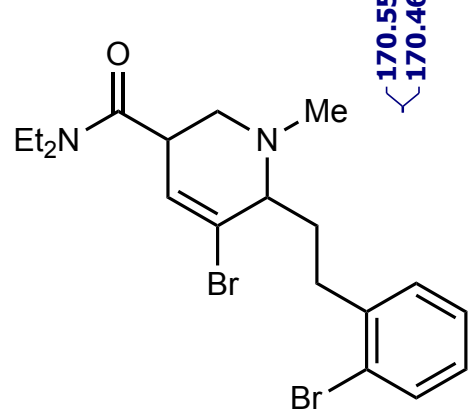

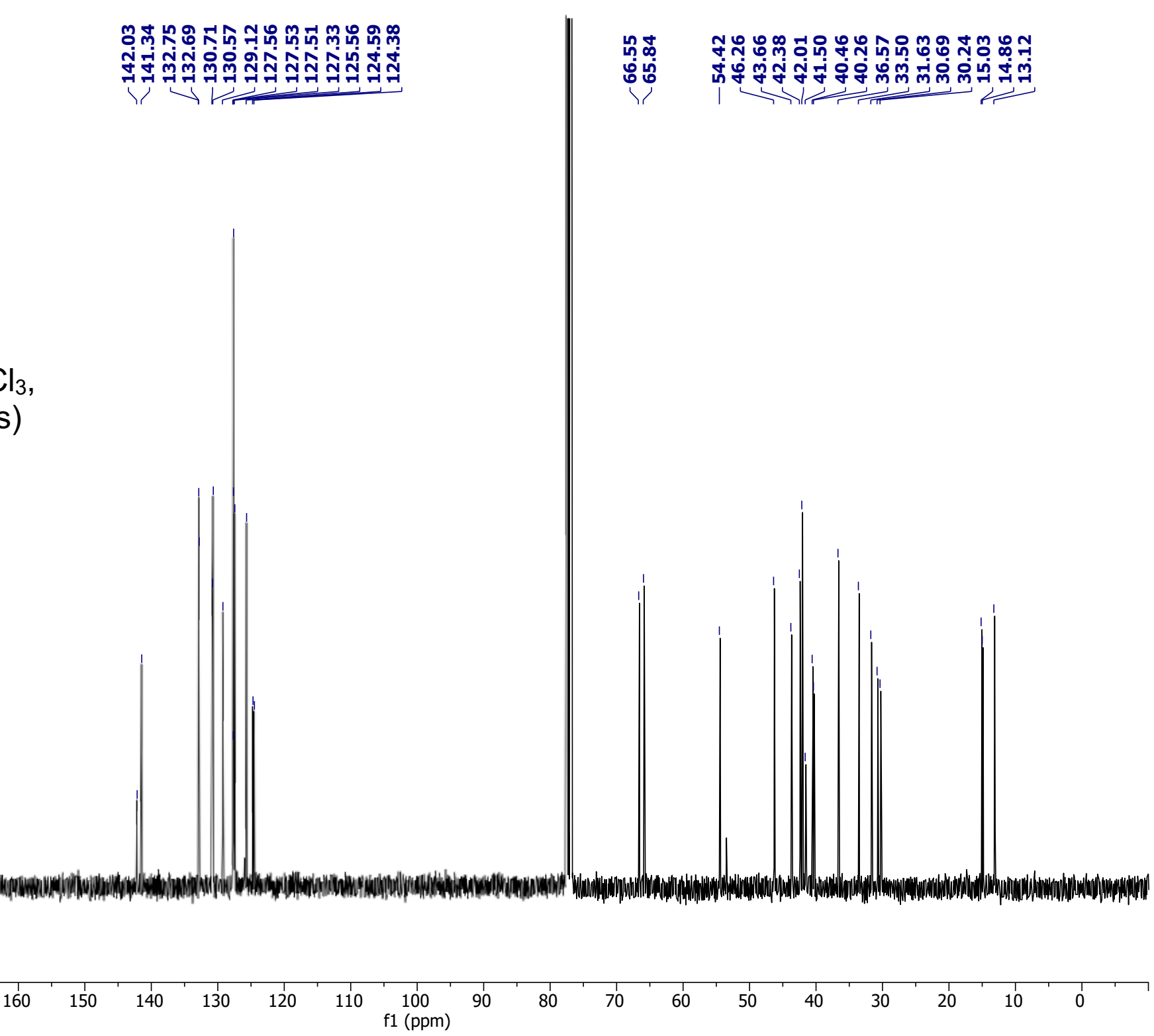

Compound 18
^{13}C NMR (101 MHz, $CDCl_3$, mixture of diastereomers)

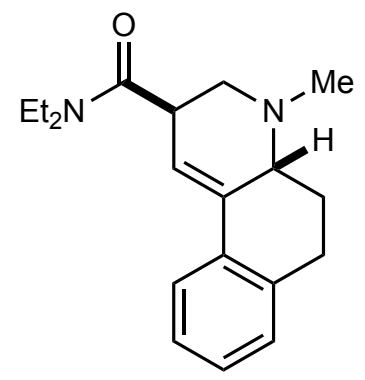

UCD0068 freebase
^{1}H NMR (400 MHz, CD_3OD)

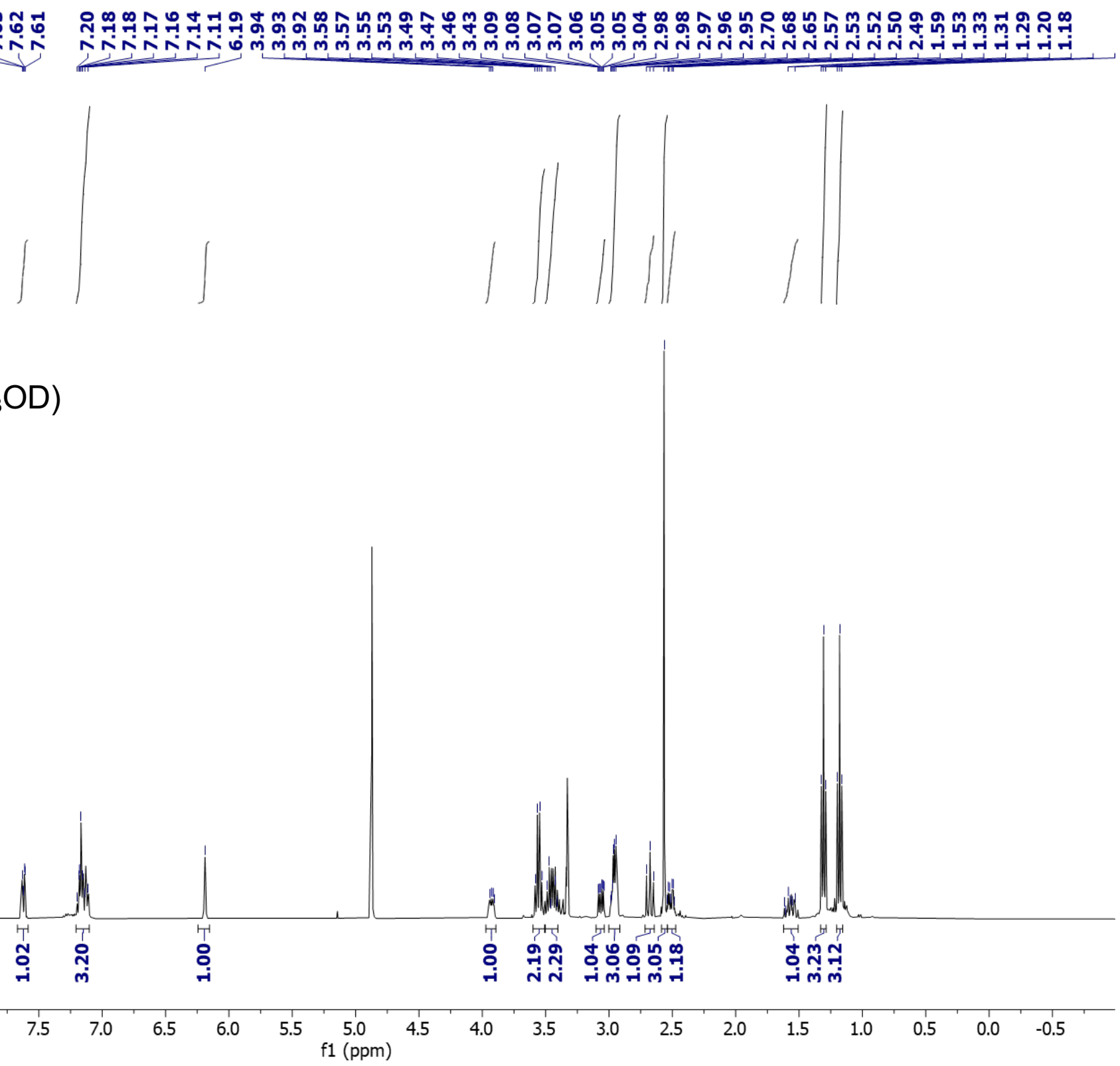

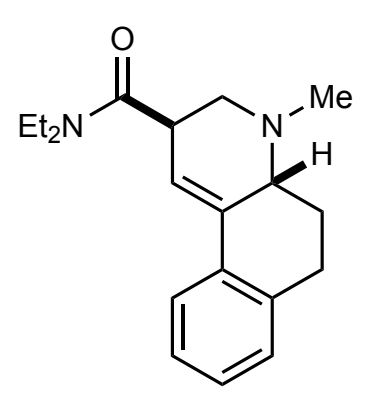

UCD0068 freebase
^{13}C NMR (101 MHz, CD_3OD)

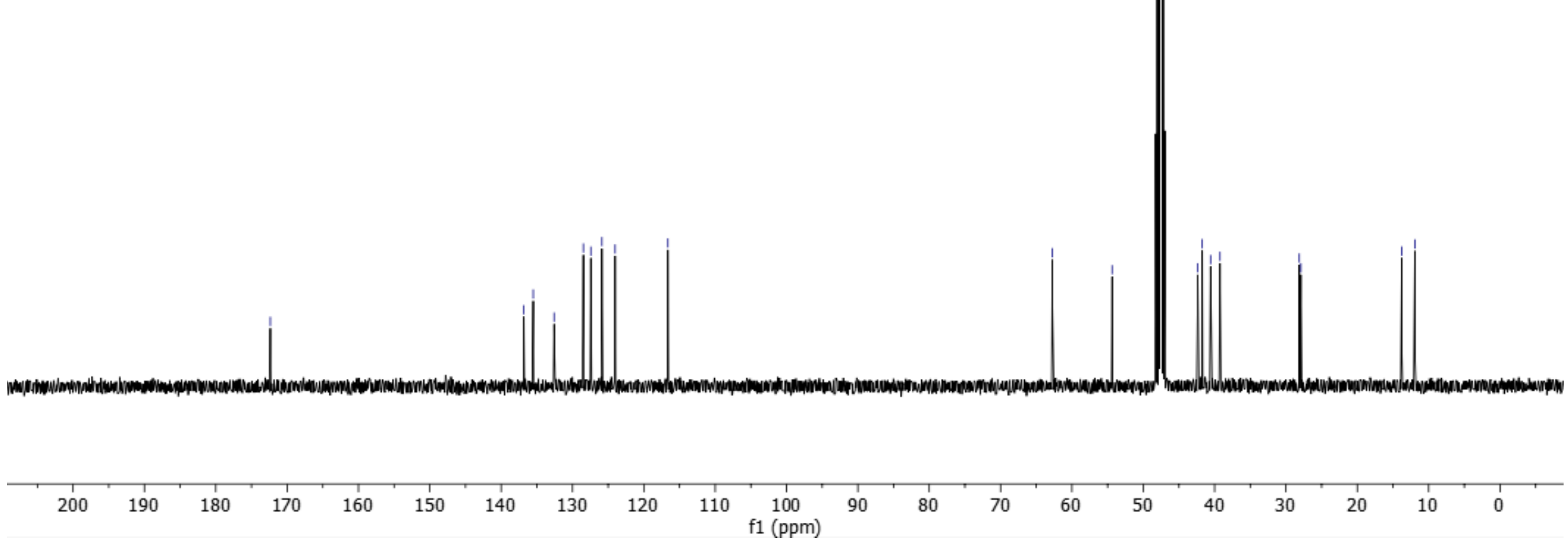

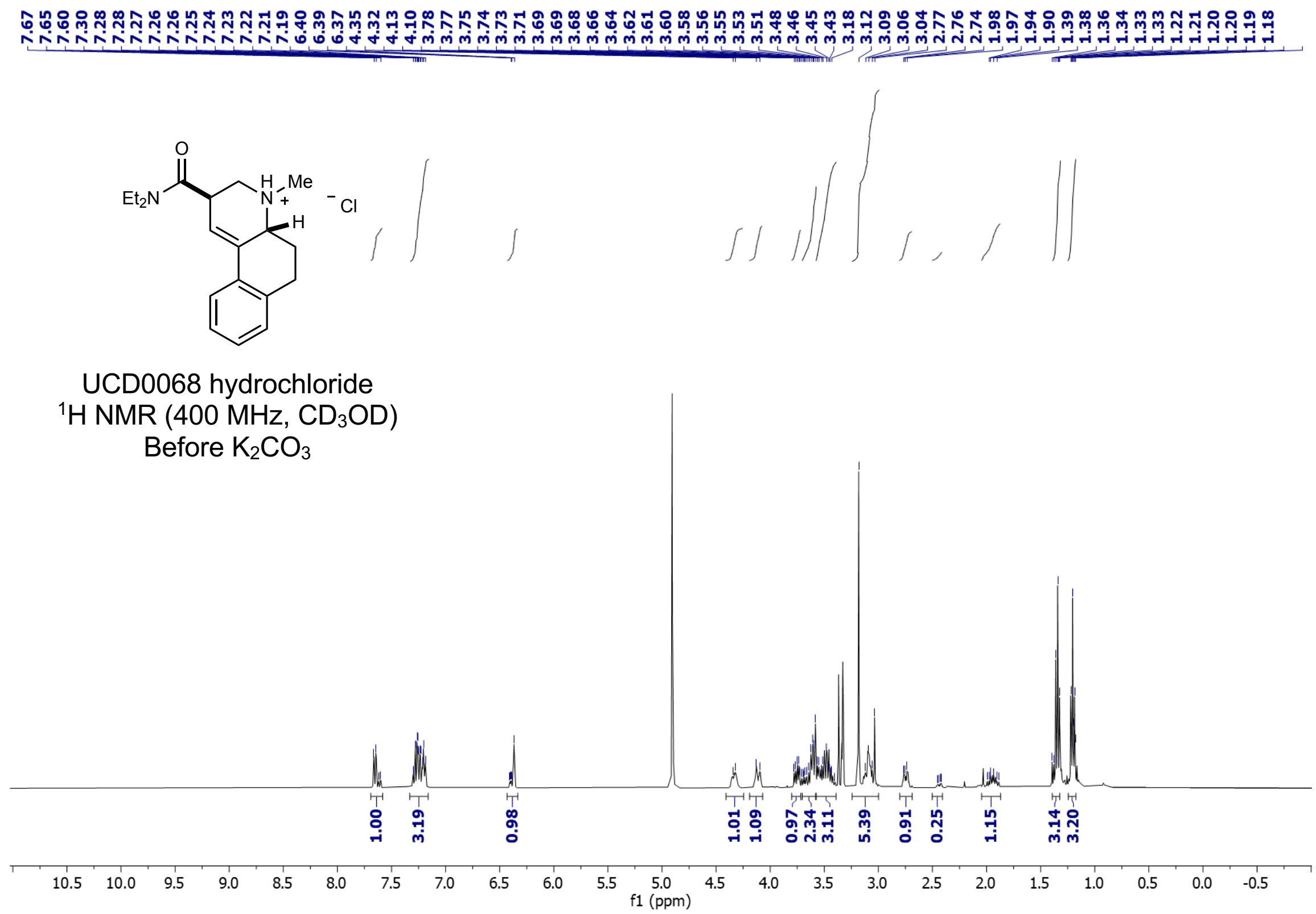
Et2N
H
Me
N+
- Cl
H
UCD0068 hydrochloride
1H NMR (400 MHz, CD3OD)
Before K2CO3
f1 (ppm)

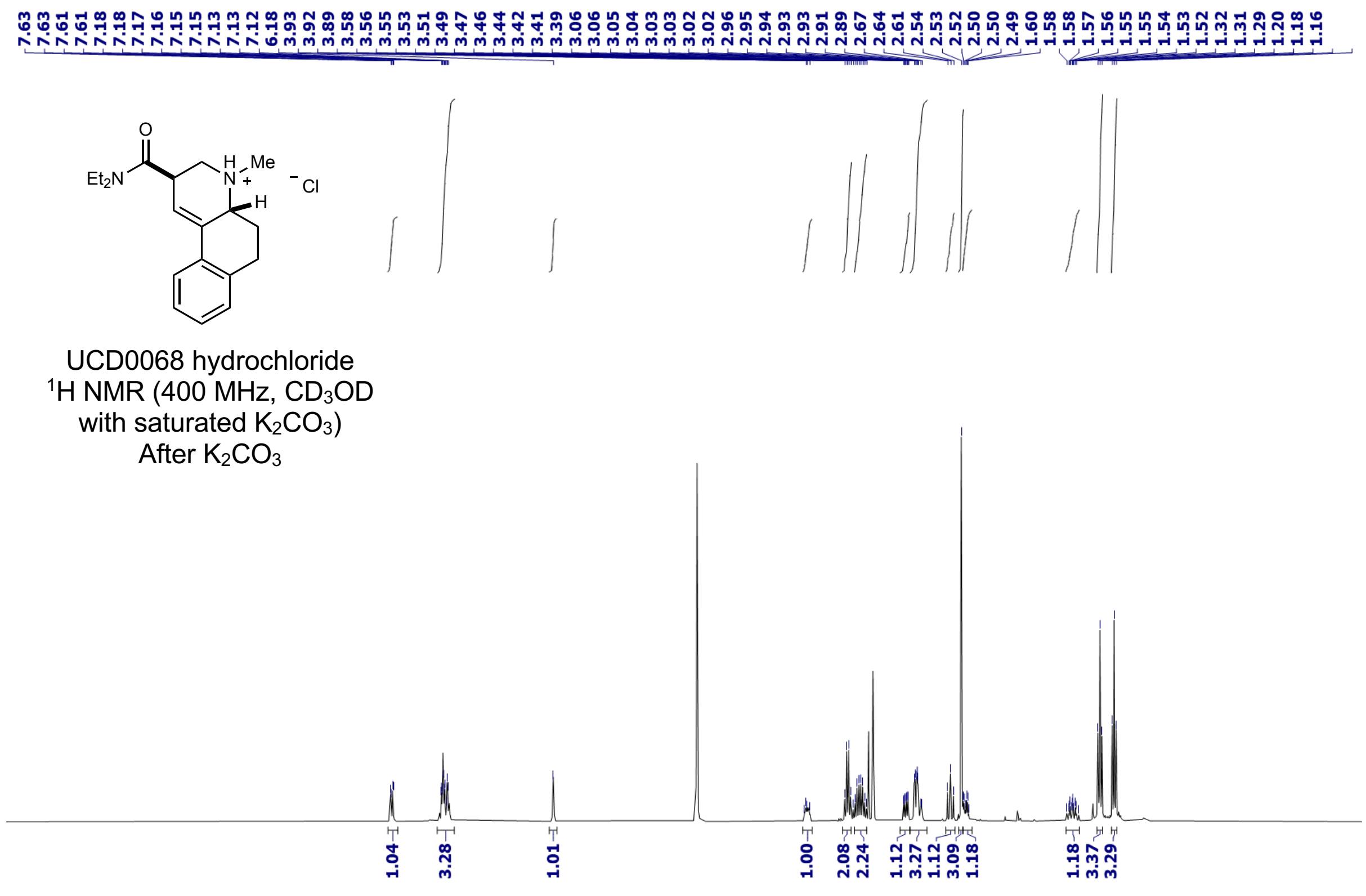
Et2N
H
Me
N+
- Cl
H
UCD0068 hydrochloride
1H NMR (400 MHz, CD3OD
with saturated K2CO3)
After K2CO3

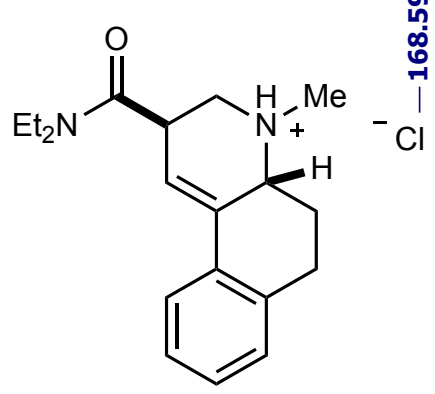

UCD0068 hydrochloride
^{13}C NMR (101 MHz, DMSO-d_6)

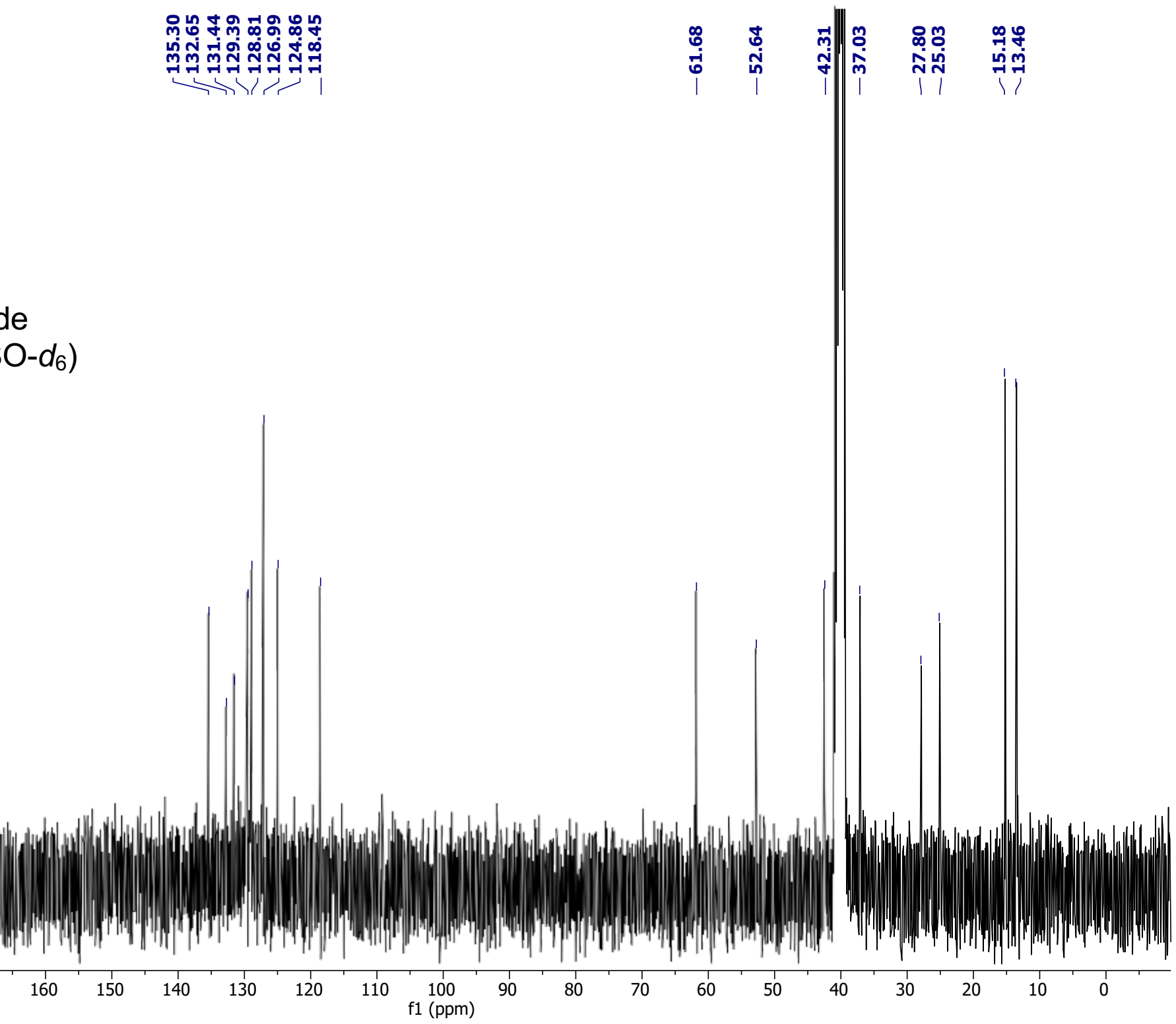

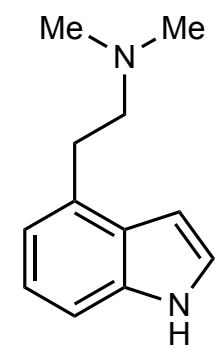

UCD0076 freebase
^{1}H NMR (300 MHz, $CDCl_3$)

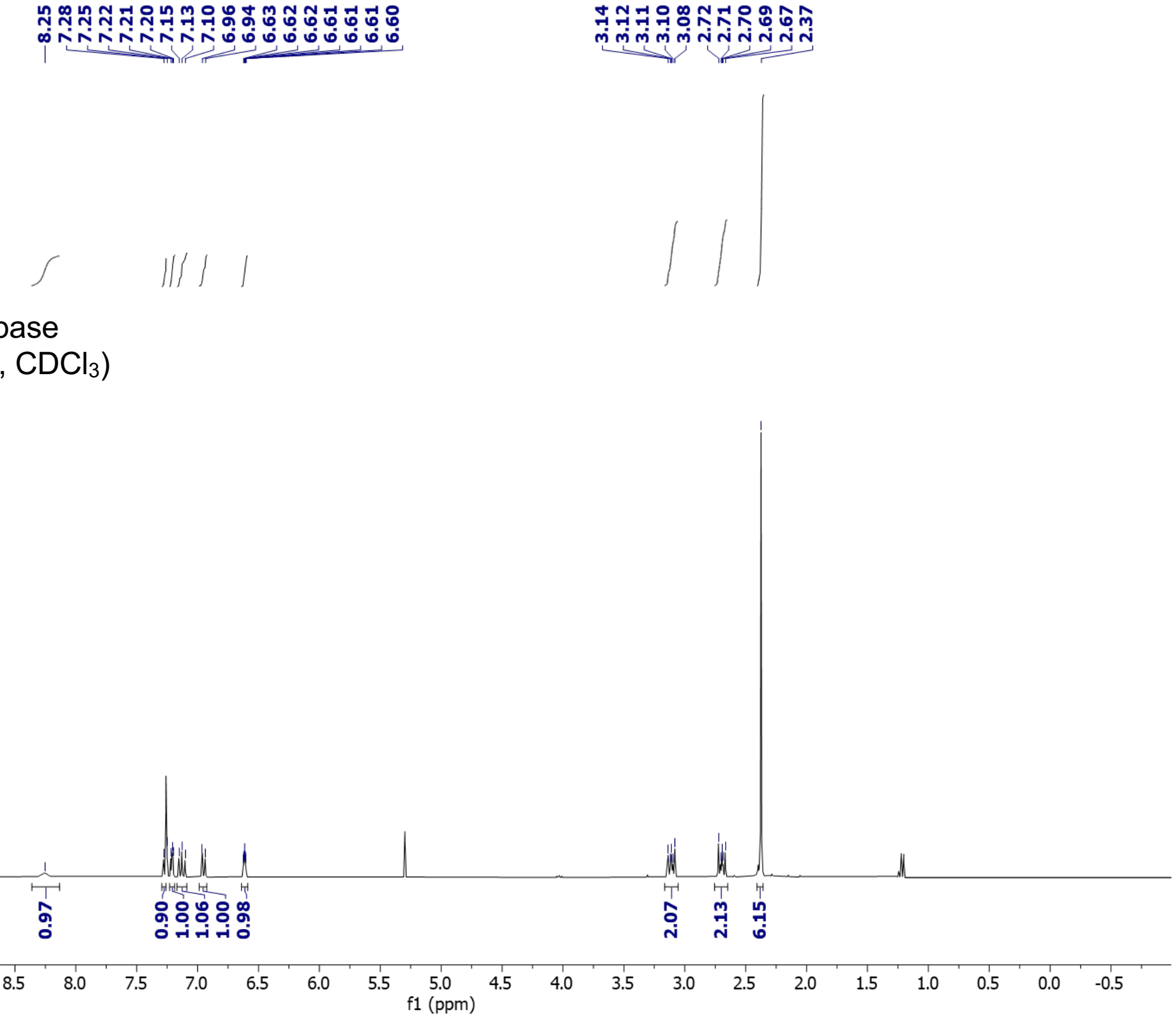

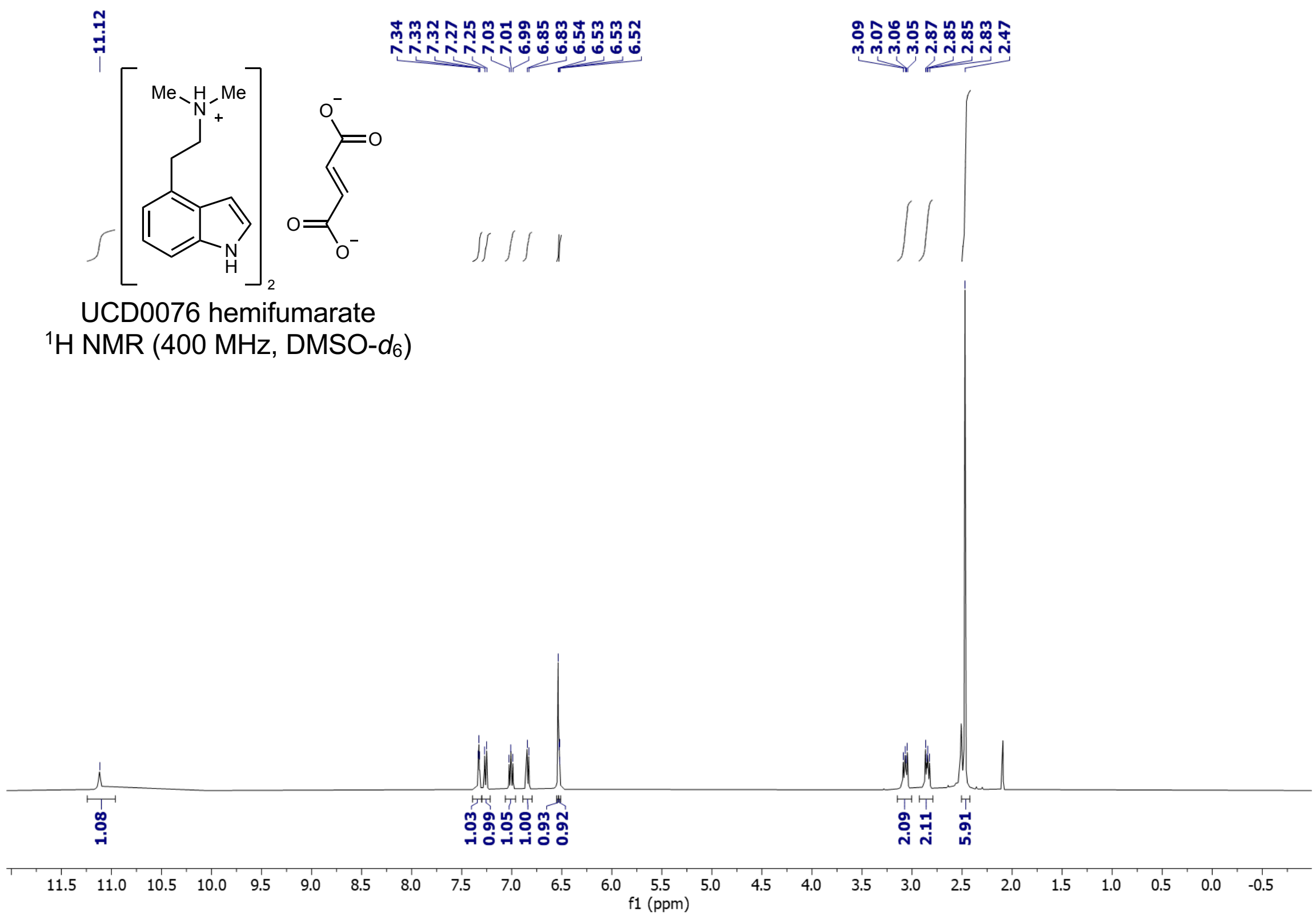
11.12
7.34
7.33
7.32
7.27
7.25
7.03
7.01
6.99
6.85
6.83
6.54
6.53
6.53
6.52
3.09
3.07
3.06
3.05
2.87
2.85
2.85
2.83
2.47
UCD0076 hemifumarate
1H NMR (400 MHz, DMSO-d6)
1.08
1.03
0.99
1.05
1.00
0.93
0.92
2.09
2.11
5.91
f1 (ppm)

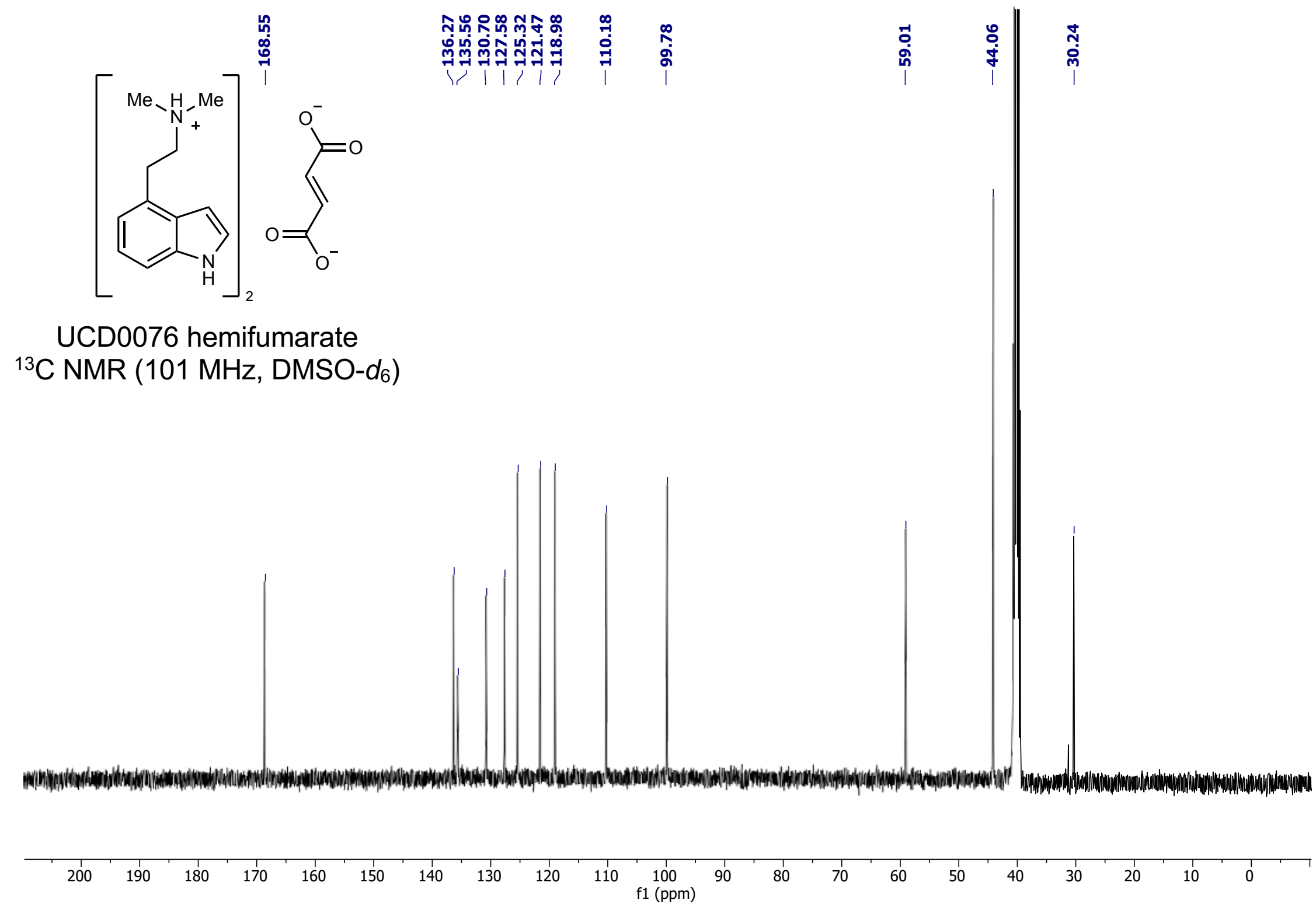
168.55
136.27
135.56
130.70
127.58
125.32
121.47
118.98
110.18
99.78
59.01
44.06
30.24
UCD0076 hemifumarate
13C NMR (101 MHz, DMSO-d6)
f1 (ppm)

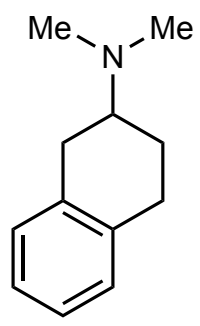

UCD0830 freebase
^{1}H NMR (600 MHz, $CDCl_3$)

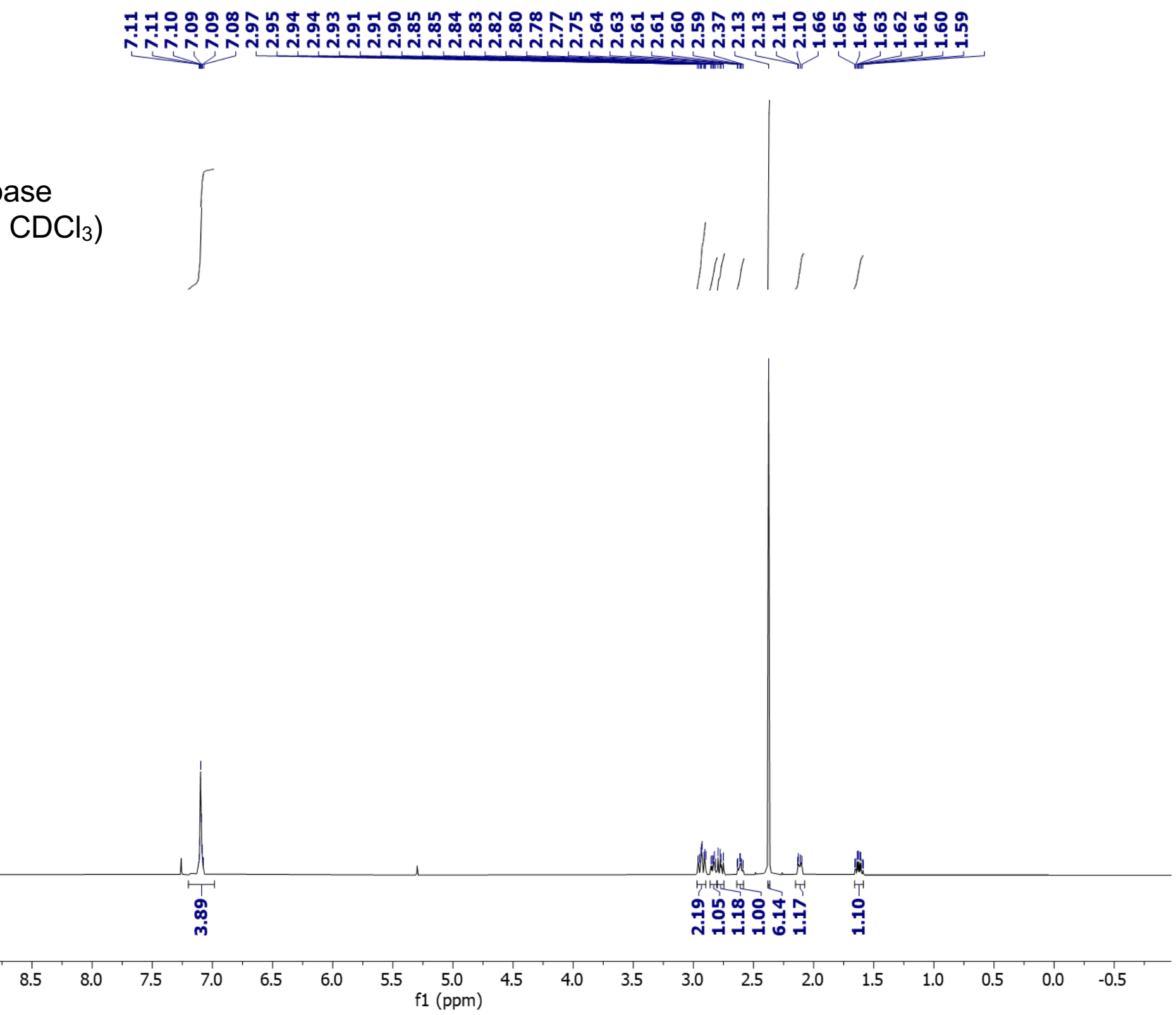

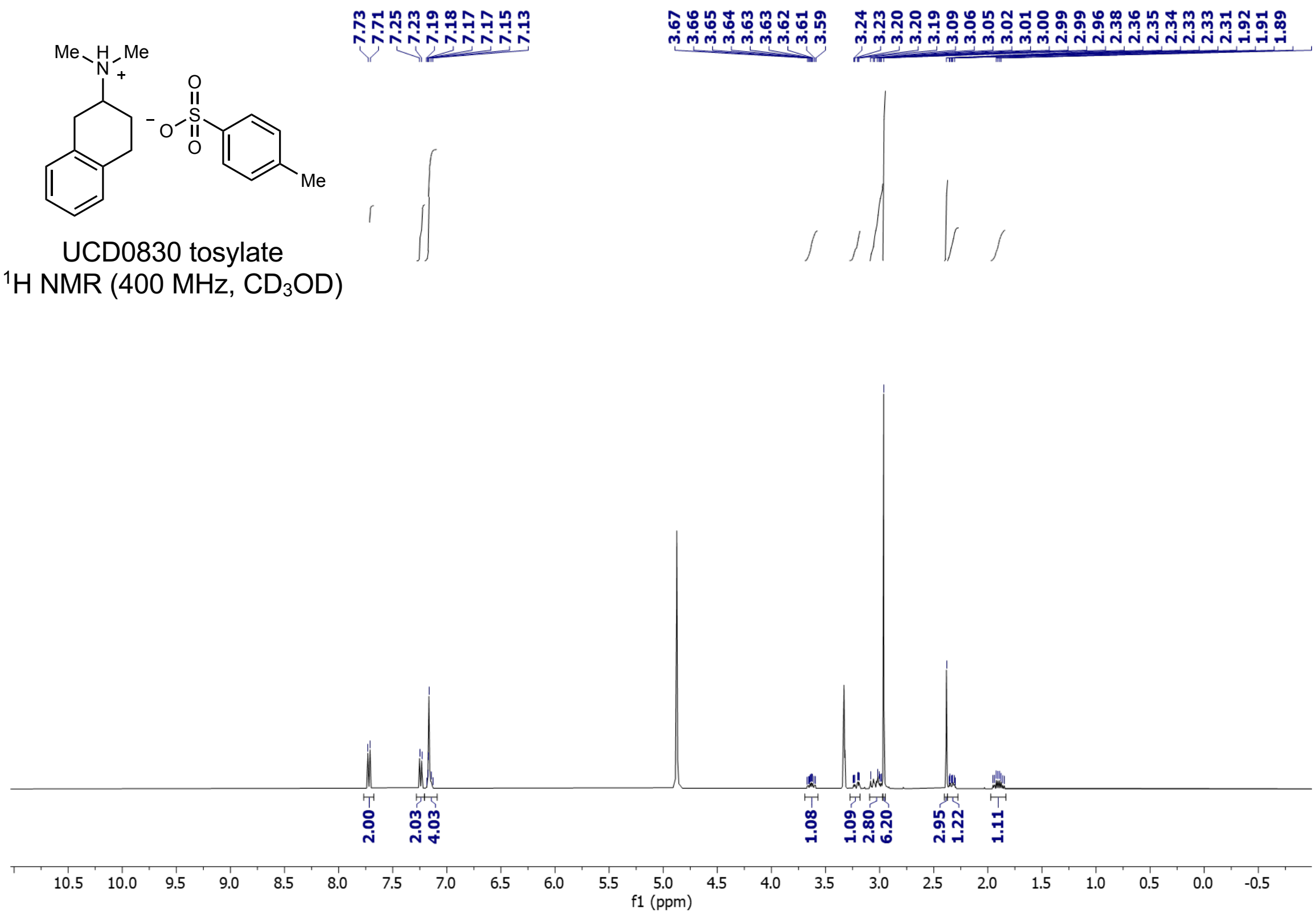
UCD0830 tosylate
^{1}H NMR (400 MHz, CD_3OD)
7.73 7.71 7.25 7.23 7.19 7.18 7.17 7.17 7.15 7.13
3.67 3.66 3.65 3.64 3.63 3.63 3.62 3.61 3.59 3.24 3.23 3.20 3.20 3.19 3.09 3.06 3.05 3.02 3.01 3.00 2.99 2.99 2.96 2.38 2.36 2.35 2.34 2.33 2.33 2.31 1.92 1.91 1.89
2.00 2.03 4.03 1.08 1.09 2.80 6.20 2.95 1.22 1.11
f1 (ppm)

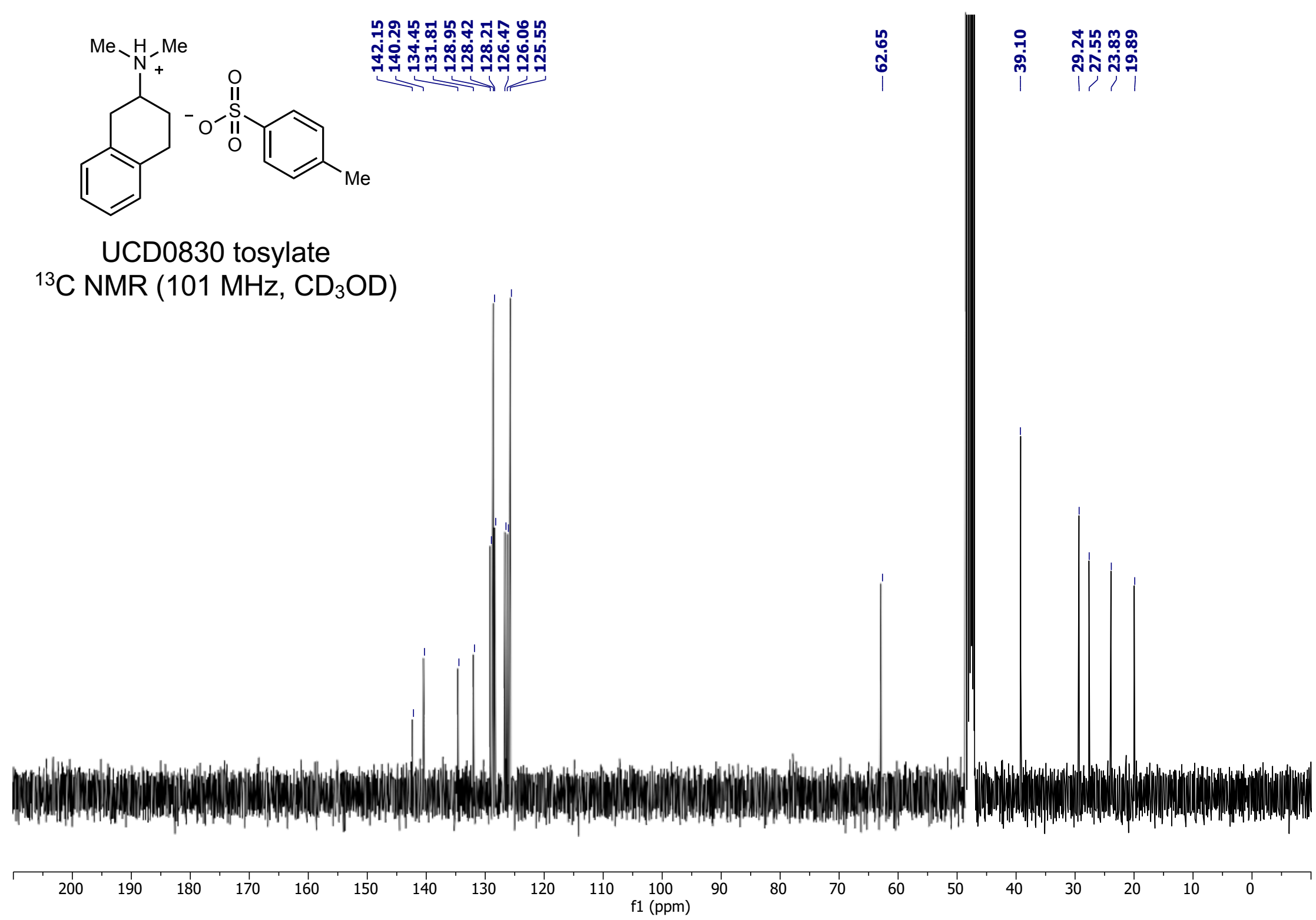
UCD0830 tosylate
^{13}C NMR (101 MHz, CD_3OD)
142.15 140.29 134.45 131.81 128.95 128.42 128.21 126.47 126.06 125.55
62.65
39.10
29.24 27.55 23.83 19.89
f1 (ppm)

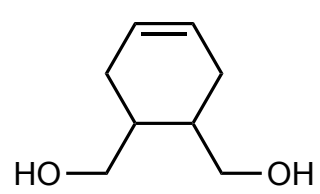

Compound 24
^{1}H NMR (400 MHz, $CDCl_3$)

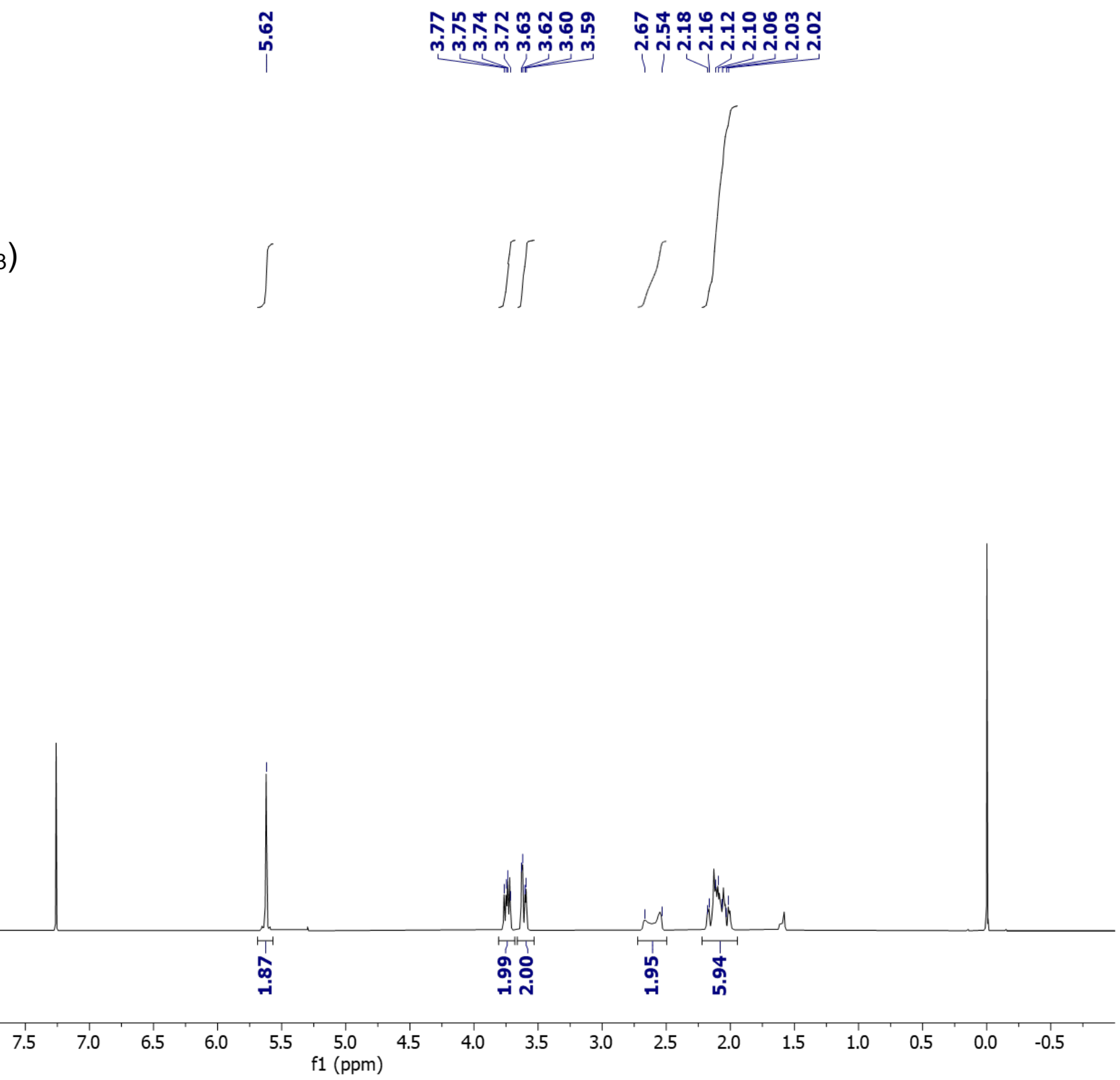

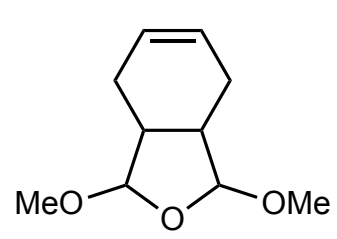

Compound 25
^{1}H NMR (400 MHz, $CDCl_3$)

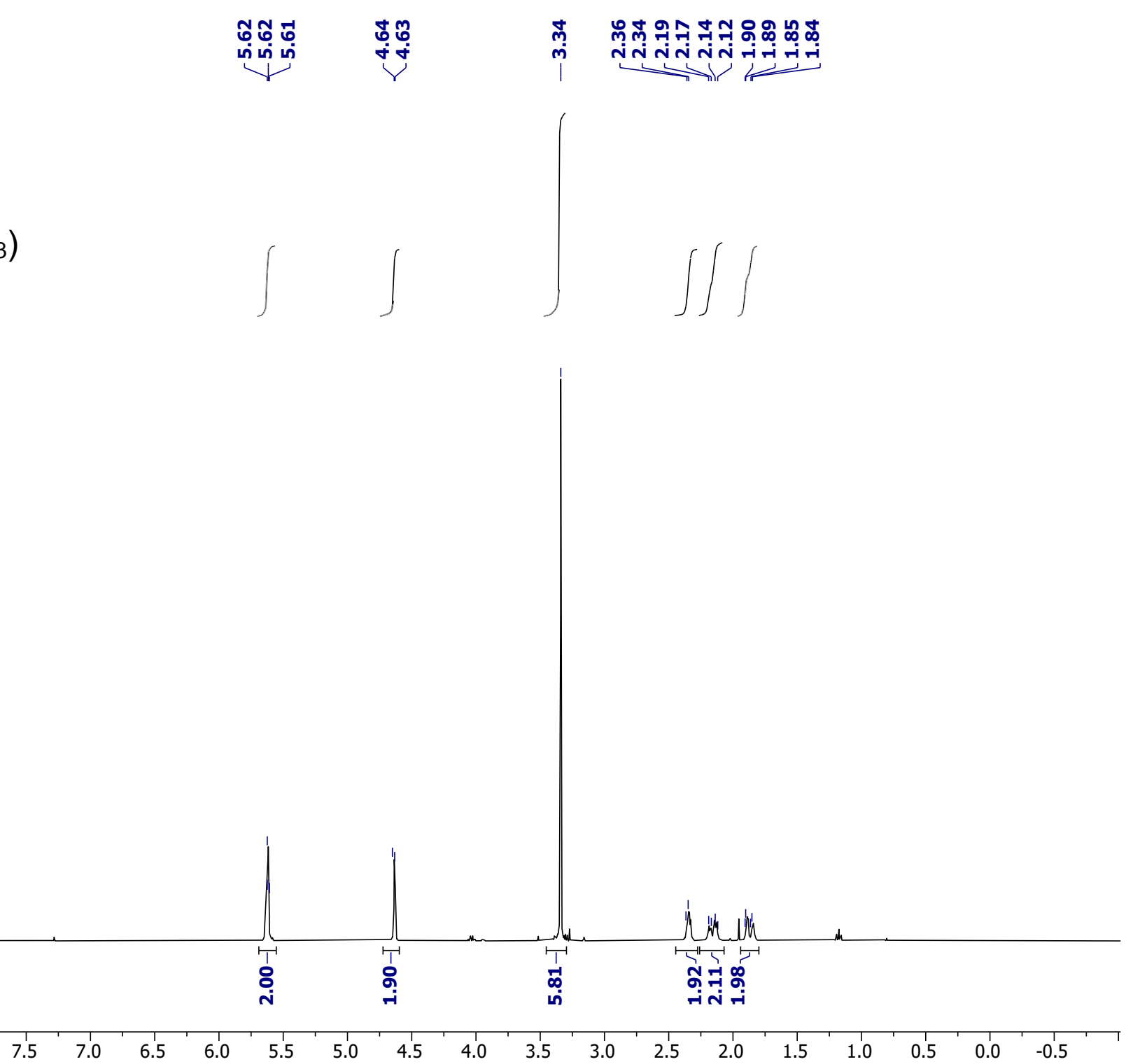

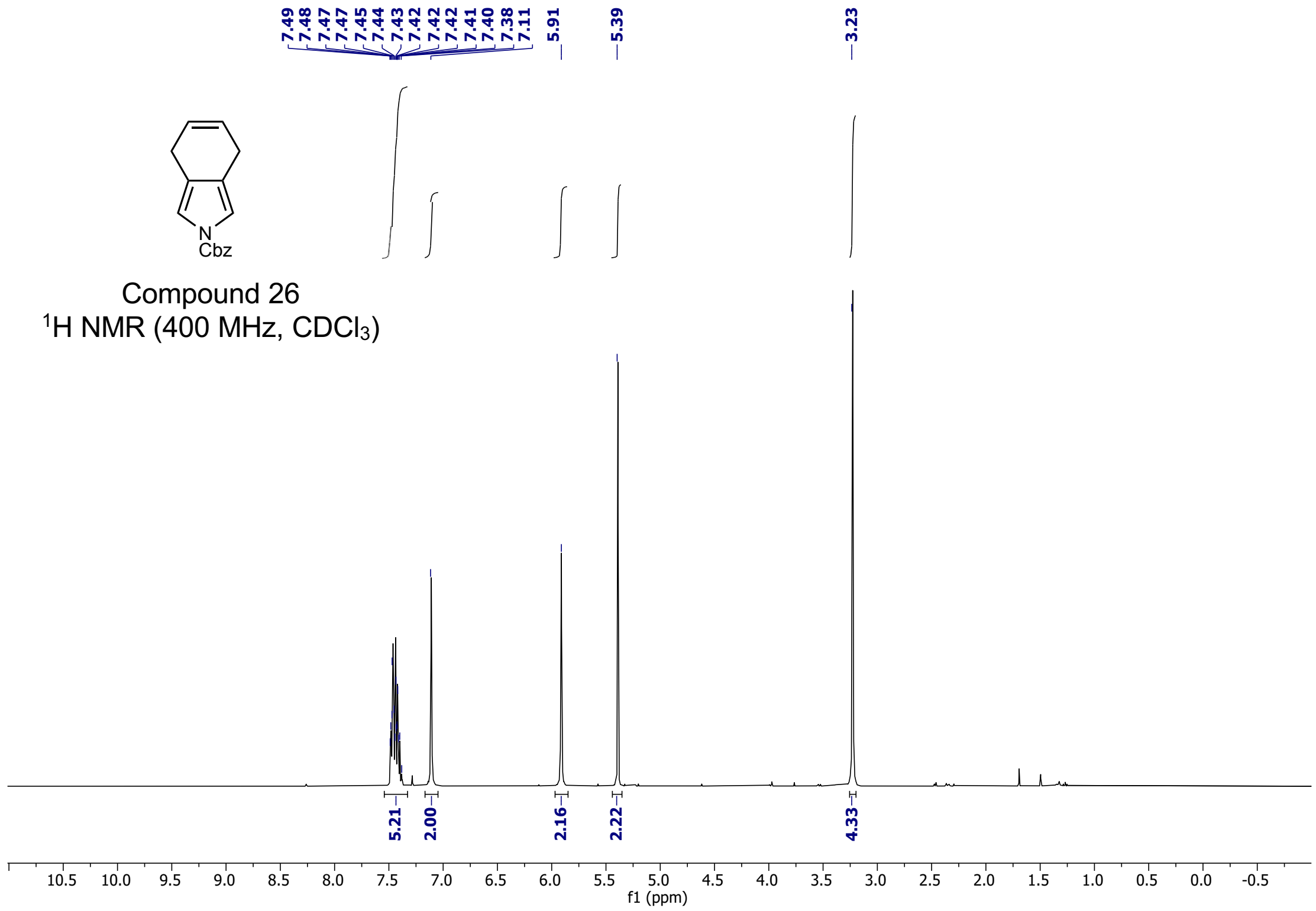

Compound 26
^{1}H NMR (400 MHz, $CDCl_3$)

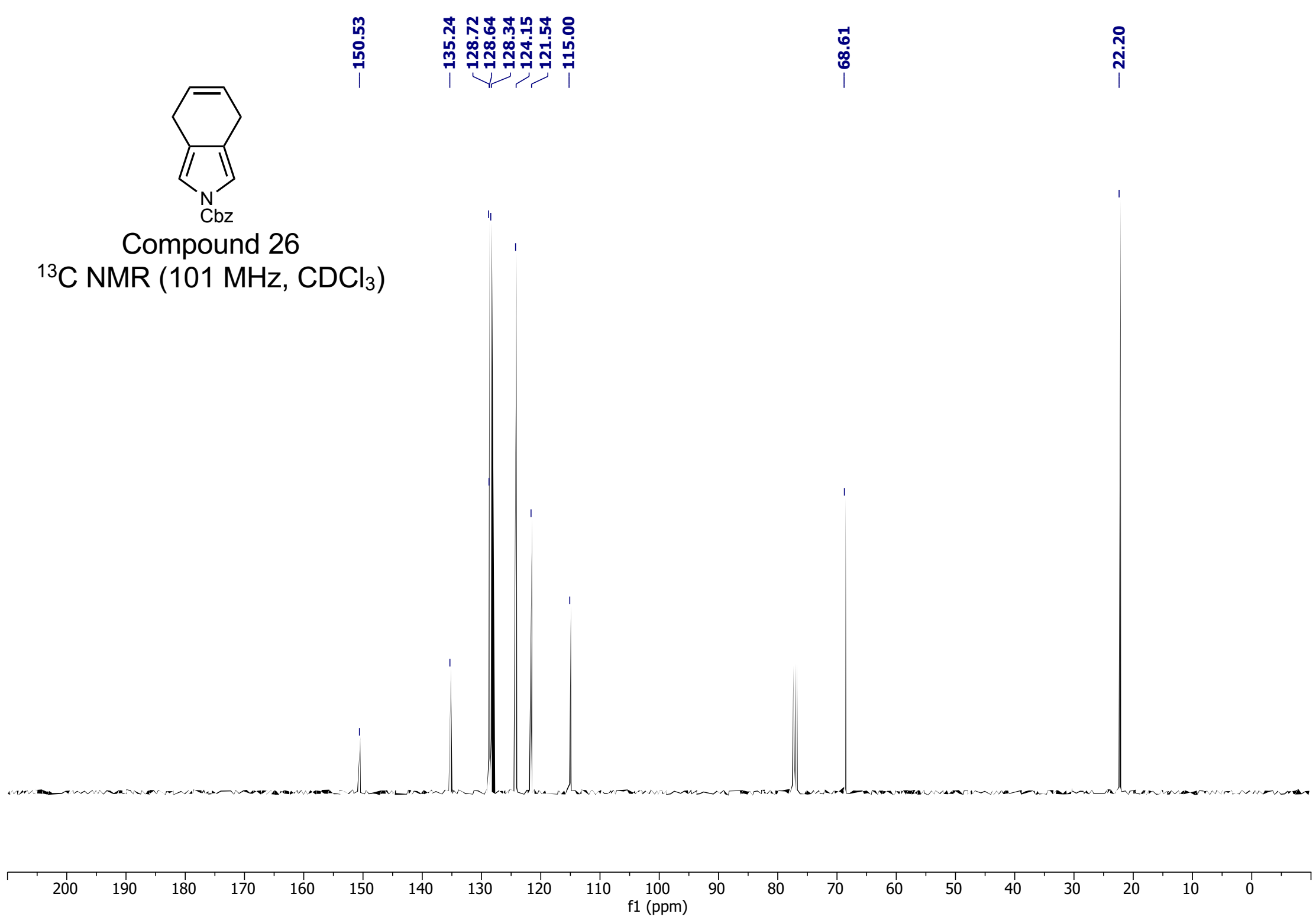

Compound 26
^{13}C NMR (101 MHz, $CDCl_3$)

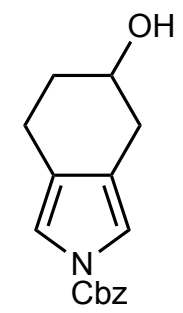

Compound 27
^{1}H NMR (400 MHz, $CDCl_3$)

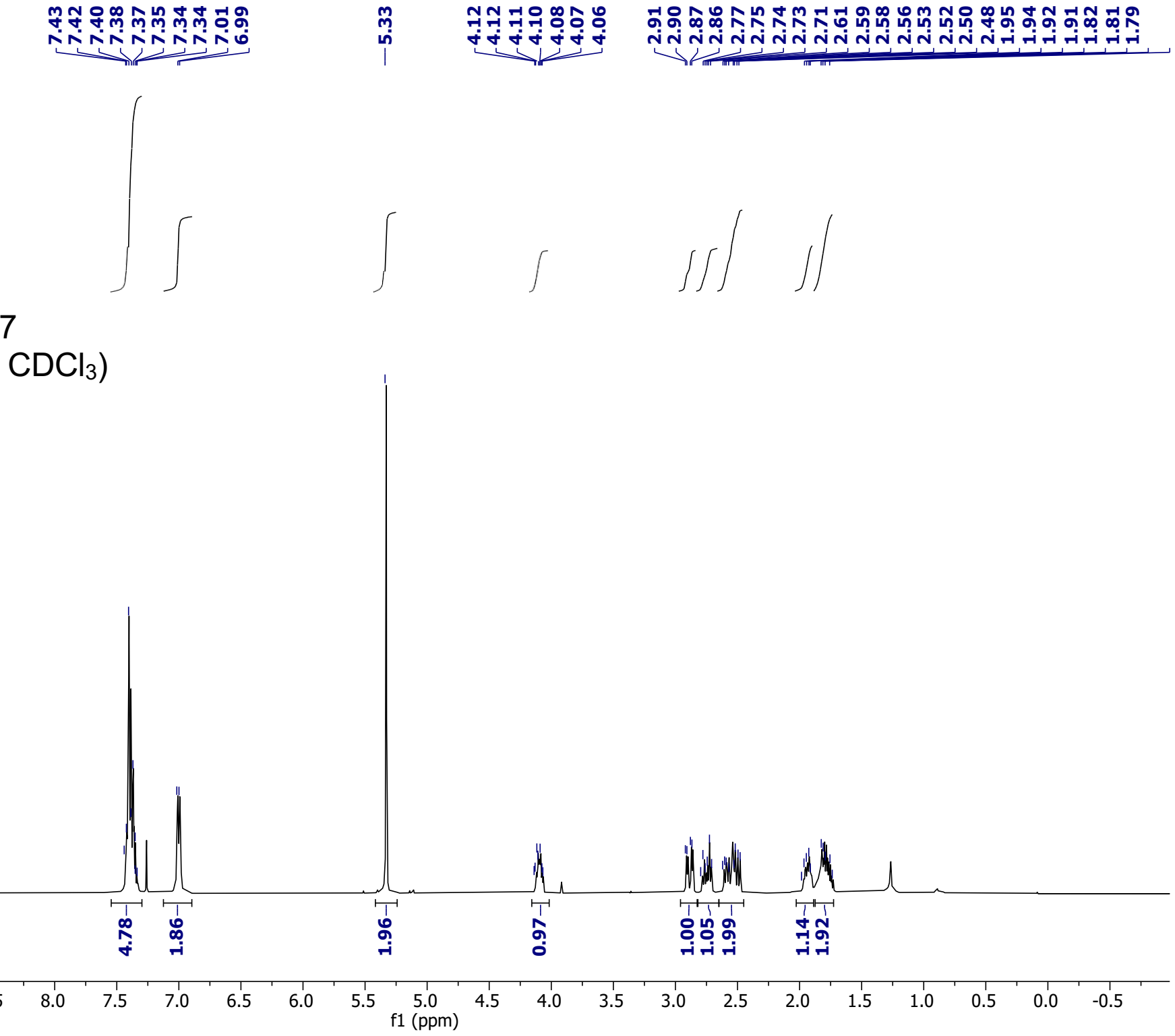

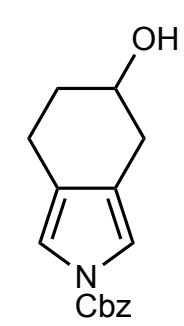

Compound 27
^{13}C NMR (101 MHz, $CDCl_3$)

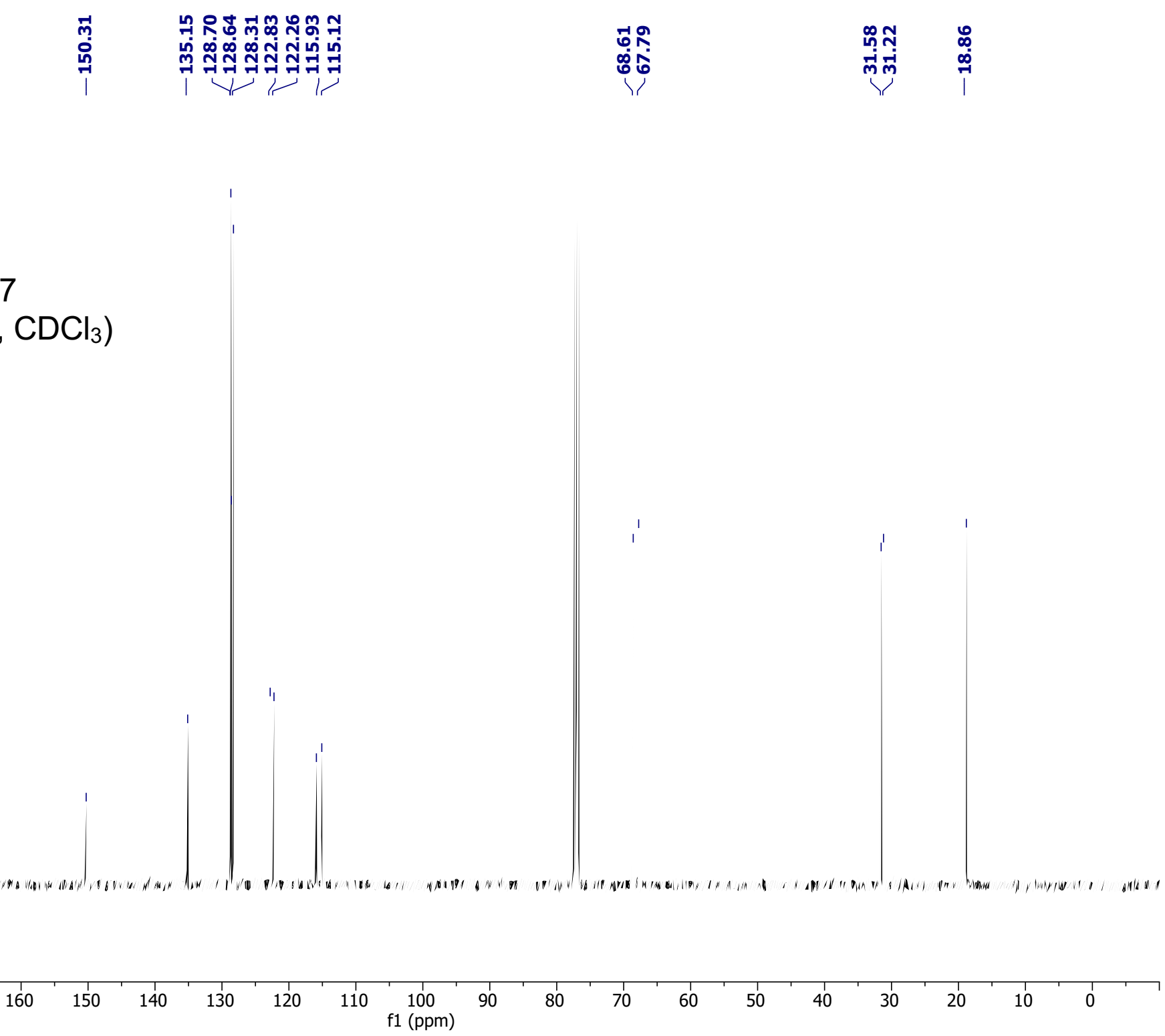

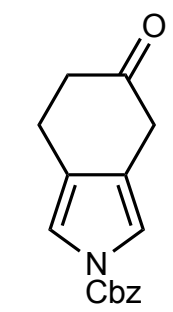

Compound 28
^{1}H NMR (400 MHz, $CDCl_3$)

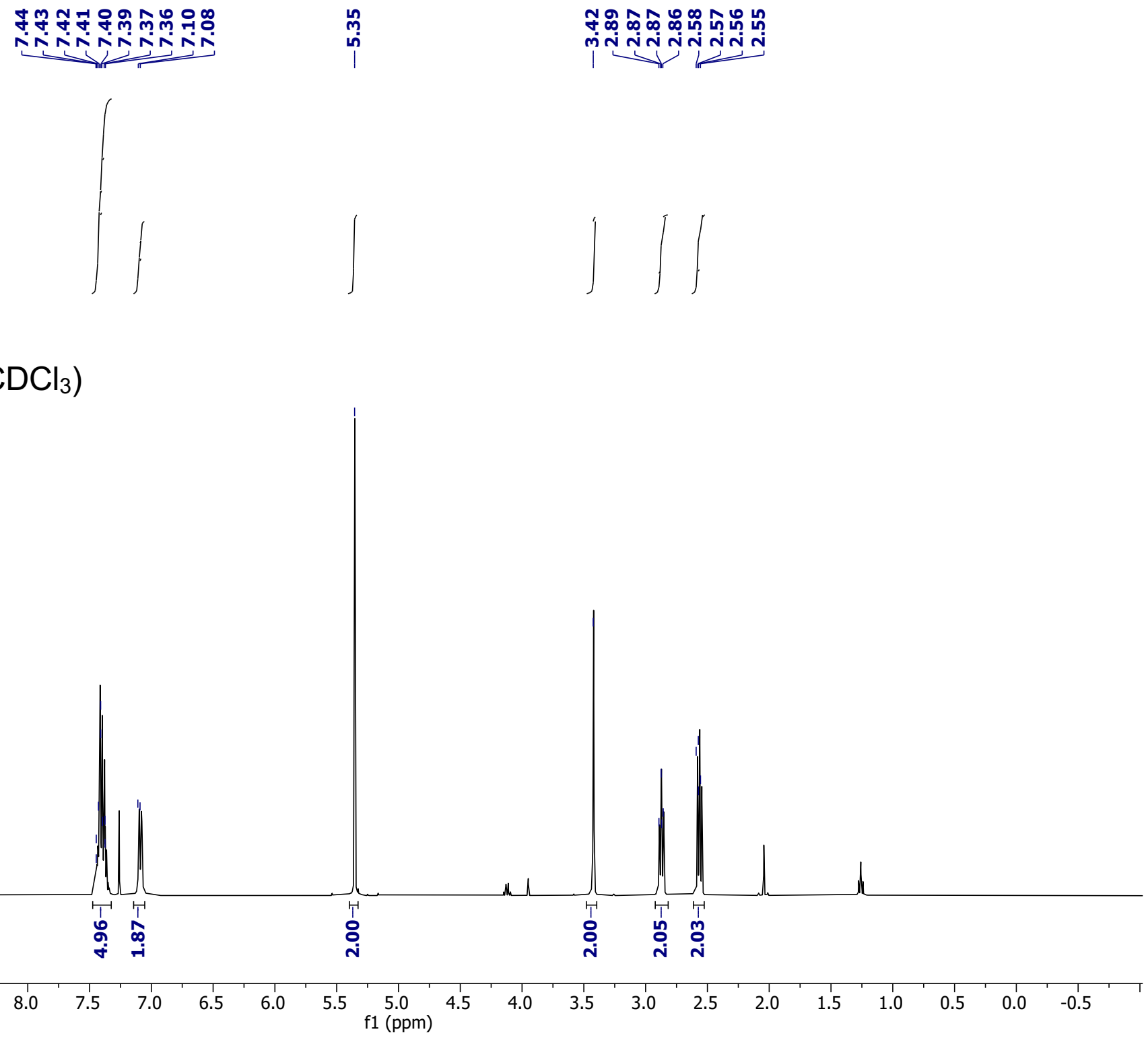

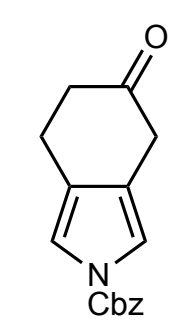

Compound 28
^{13}C NMR (101 MHz, $CDCl_3$)

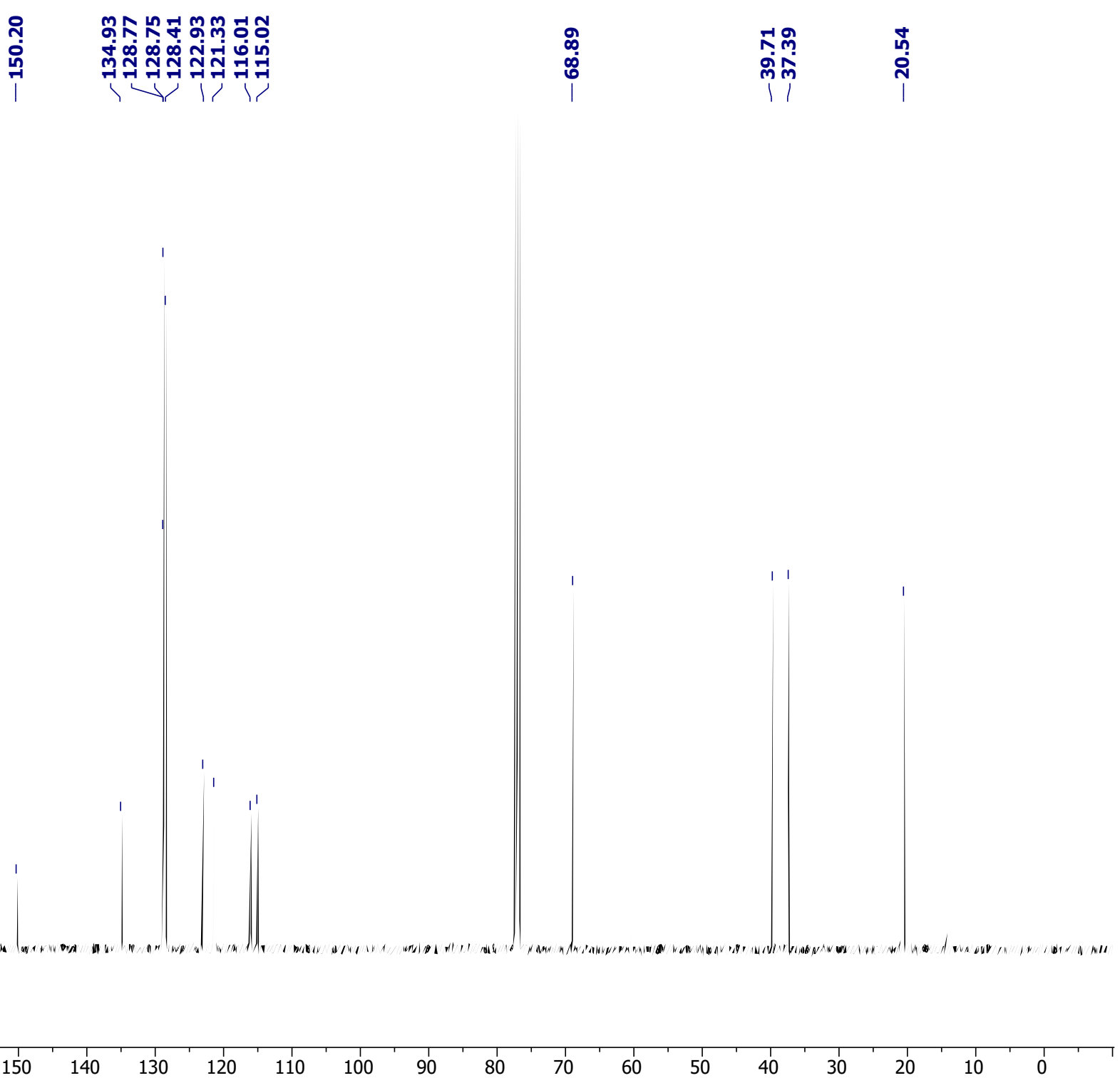

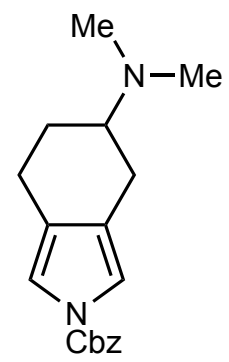

Compound 29
^{1}H NMR (600 MHz, $CDCl_3$)

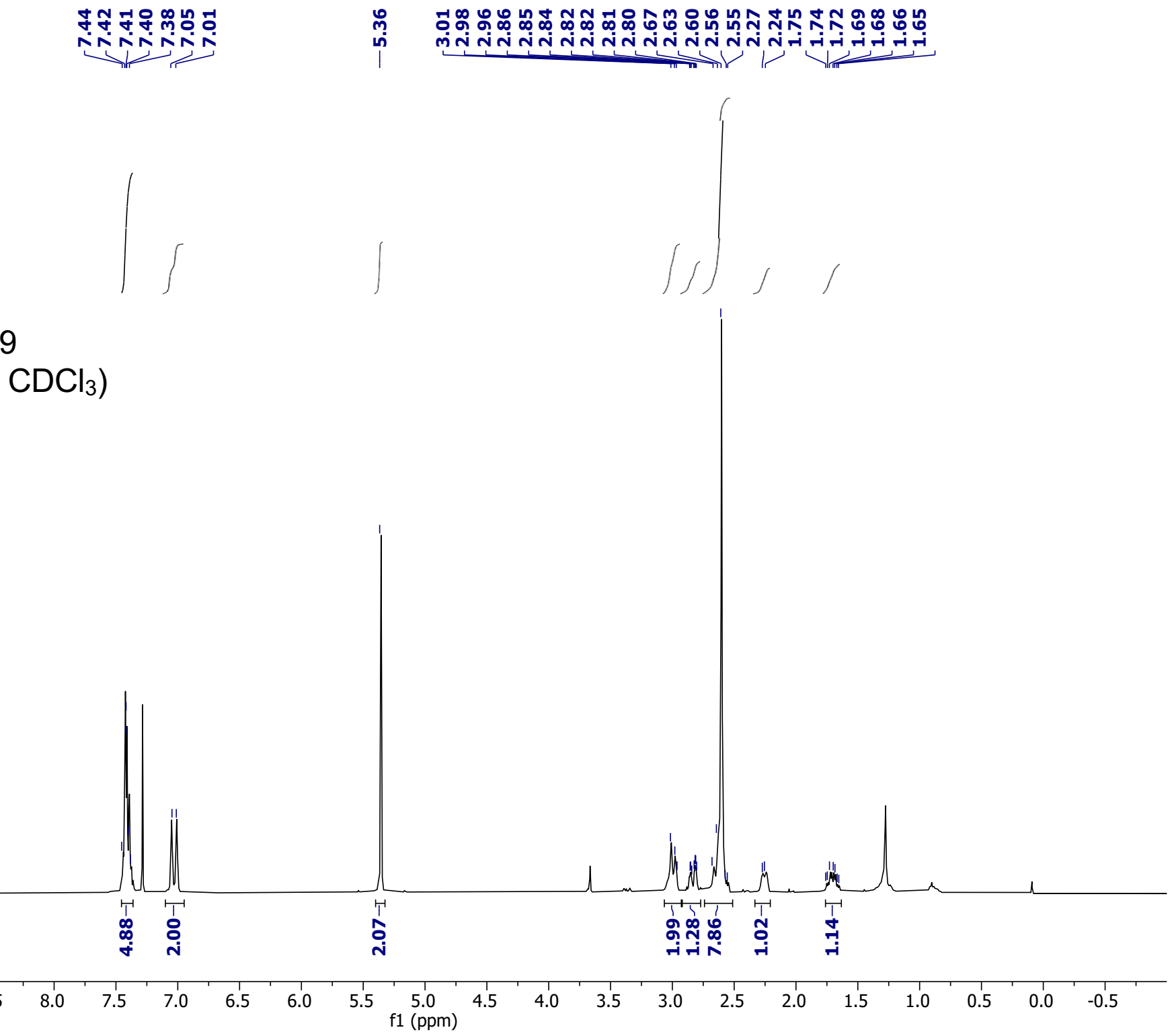

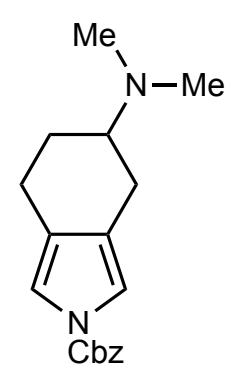

Compound 29
^{13}C NMR (150 MHz, $CDCl_3$)

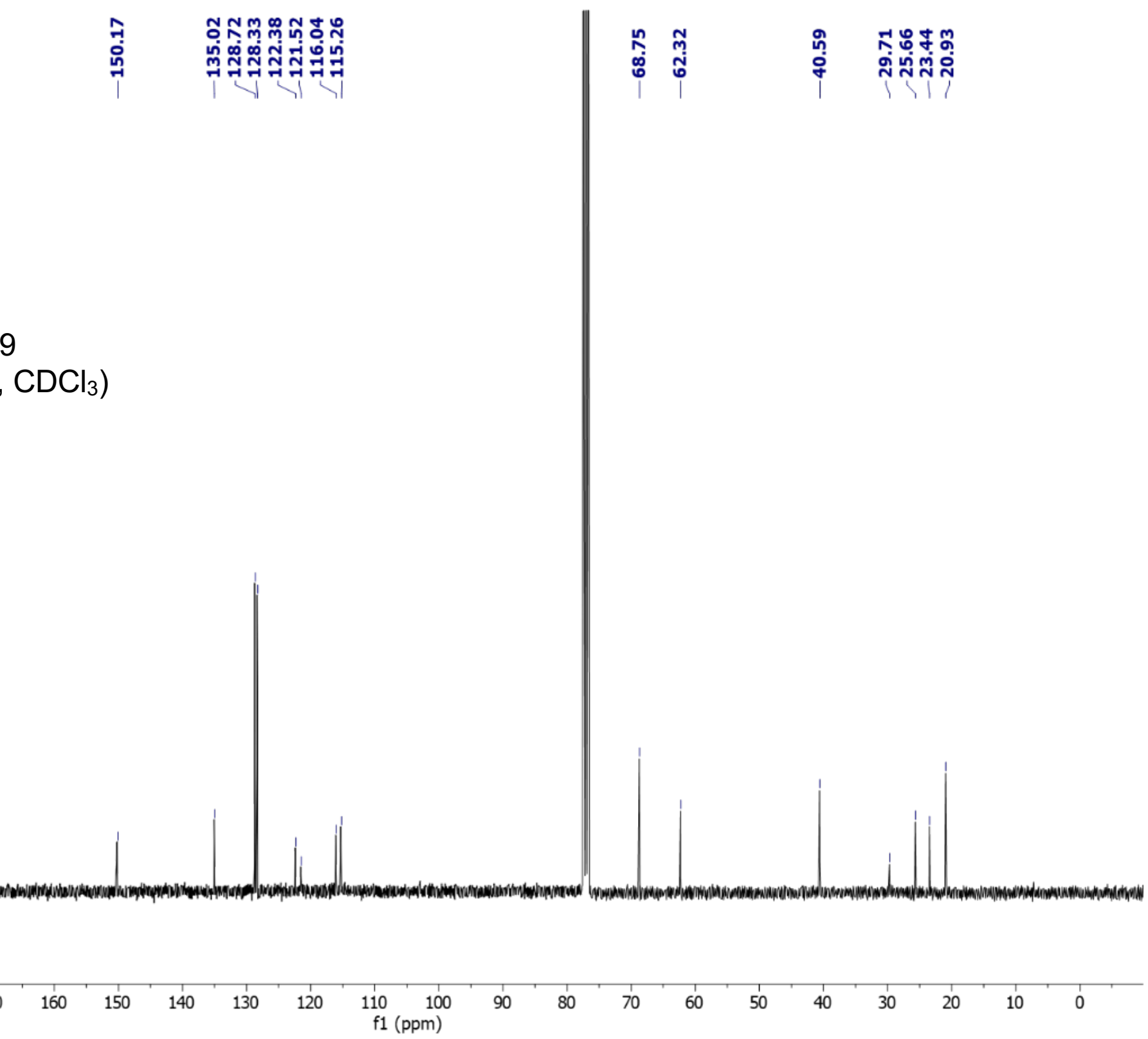

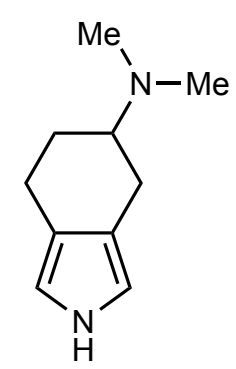

UCD0829 freebase
^1H NMR (400 MHz, CD_3OD)

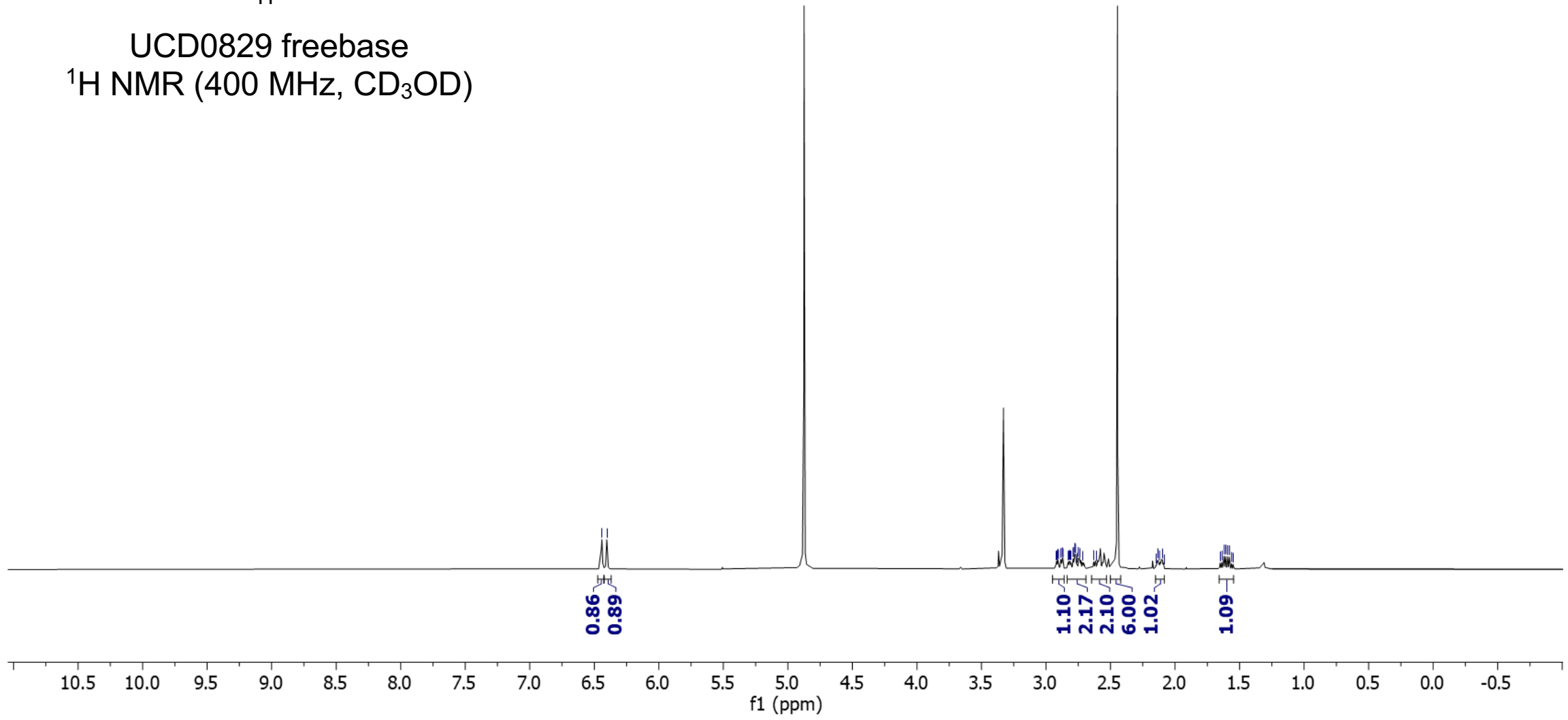

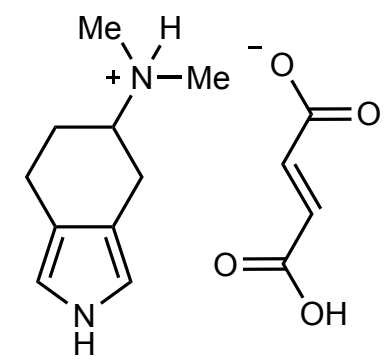

UCD0829 fumarate
^1H NMR (400 MHz, CD_3OD)

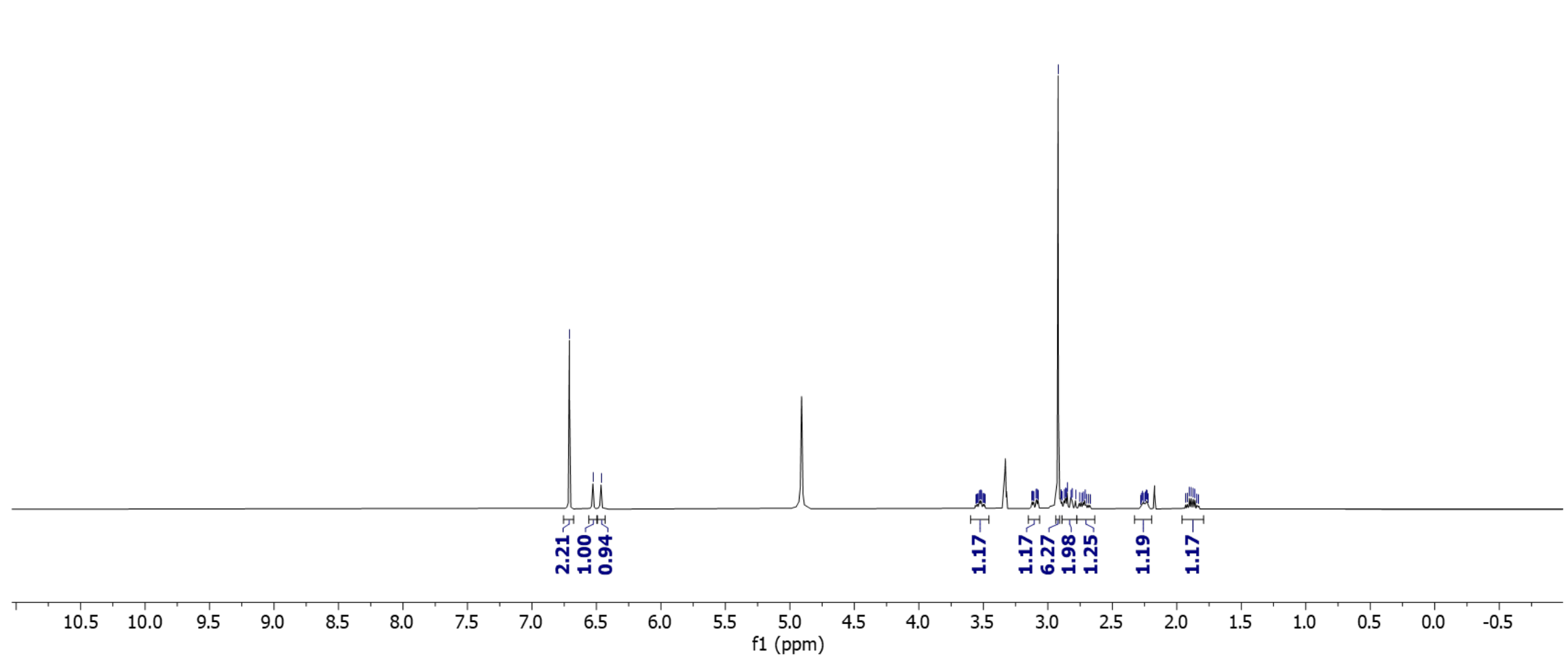

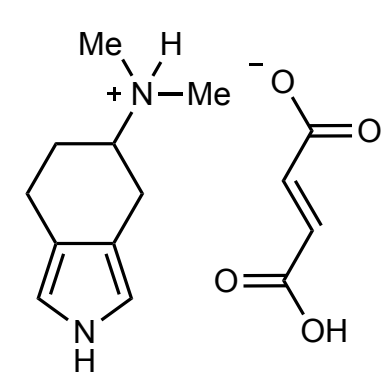

UCD0829 fumarate
^{13}C NMR (101 MHz, CD_3OD)

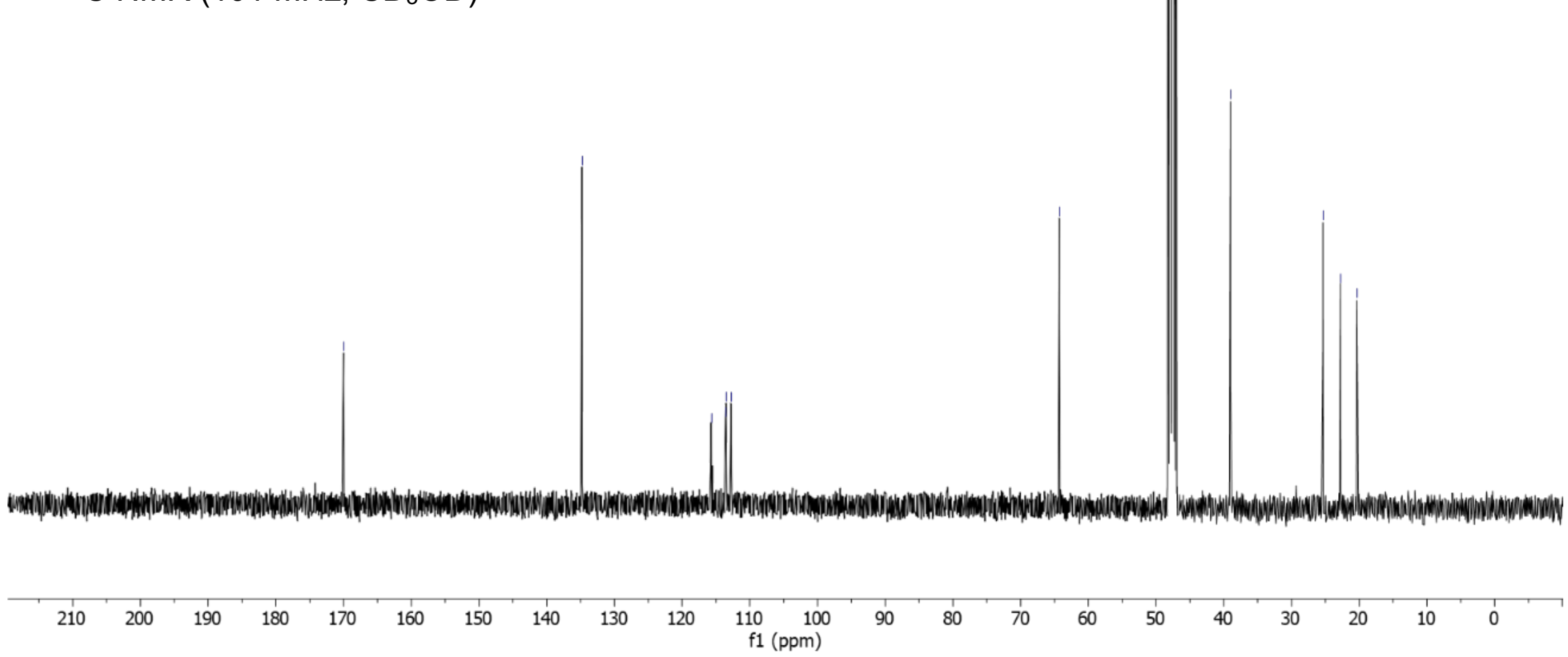

UCD0162 freebase
^1H NMR (400 MHz, CD_3OD)

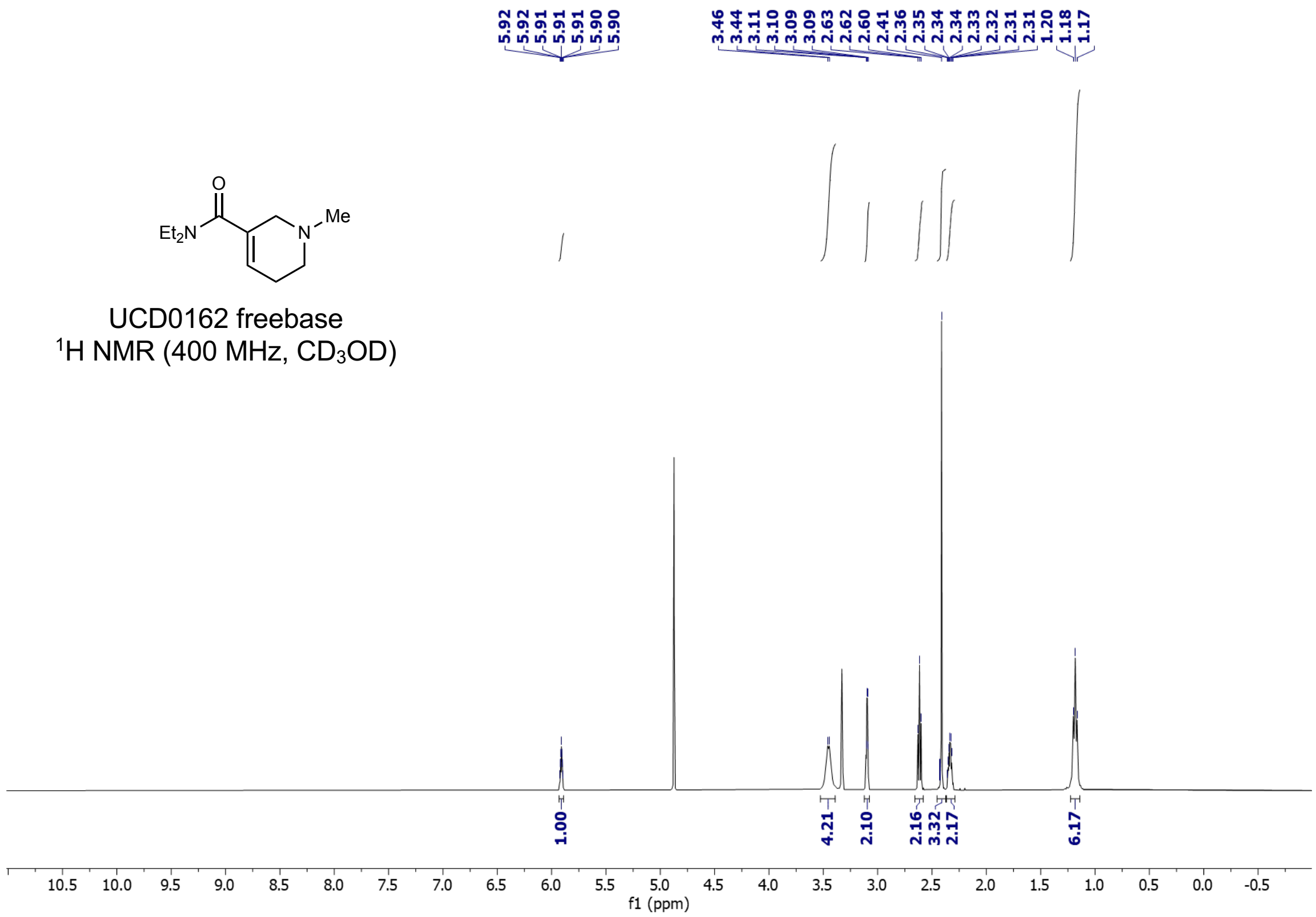

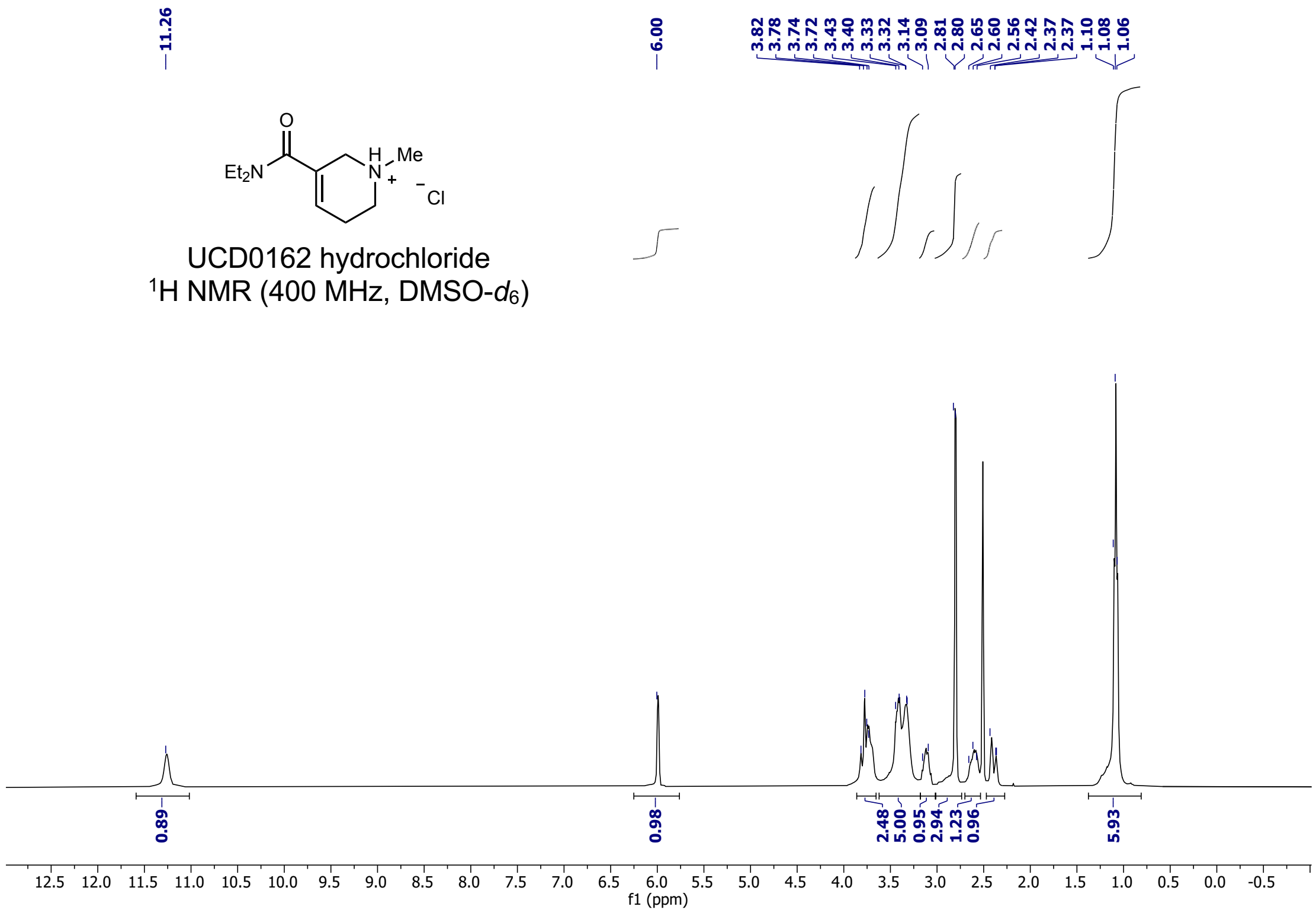
UCD0162 hydrochloride
^{1}H NMR (400 MHz, DMSO-d_6)
11.26
6.00
3.82
3.78
3.74
3.72
3.43
3.40
3.33
3.32
3.14
3.09
2.81
2.80
2.65
2.60
2.56
2.42
2.37
2.37
1.10
1.08
1.06
0.89
0.98
2.48
5.00
0.95
2.94
1.23
0.96
5.93
f1 (ppm)

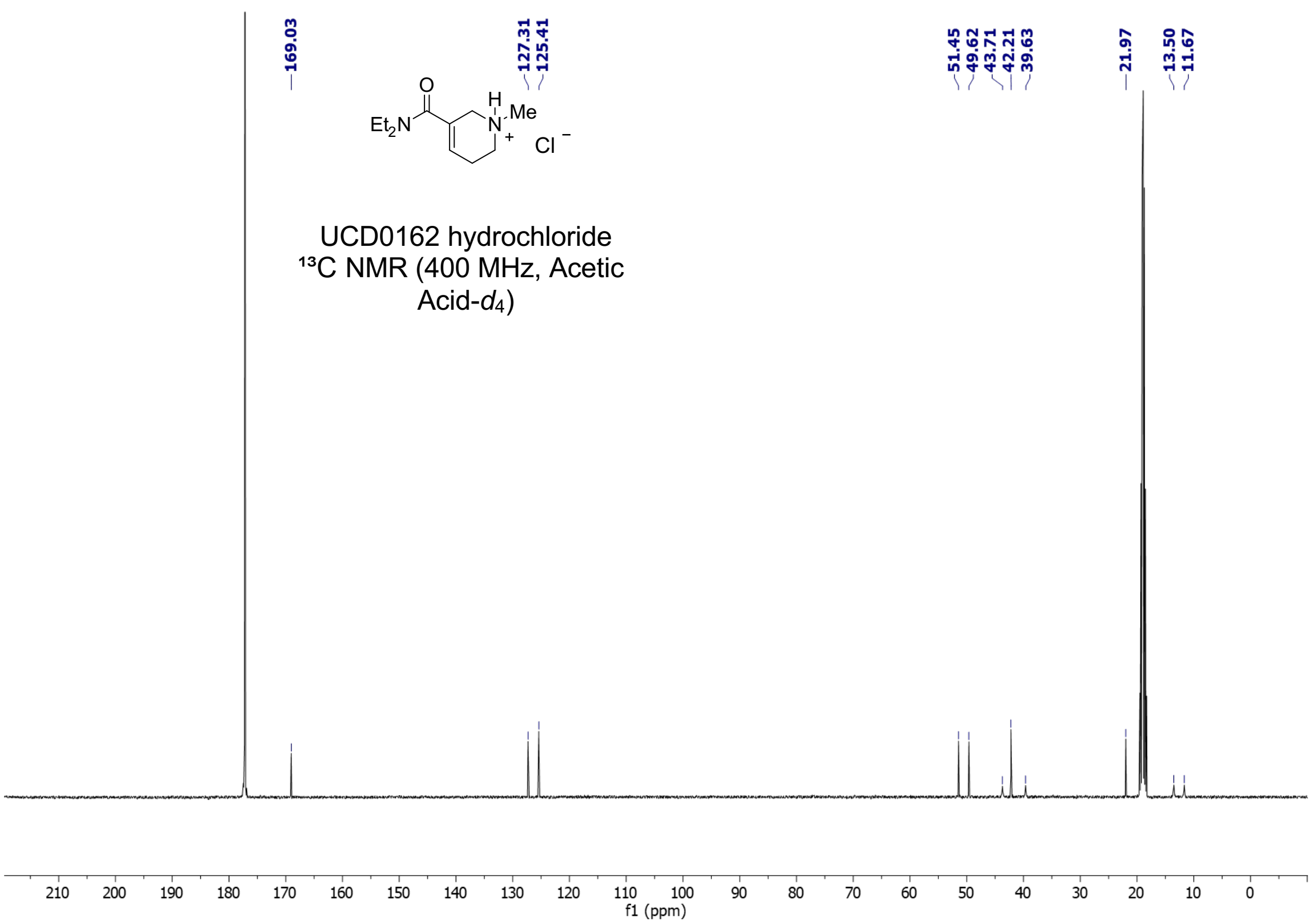
UCD0162 hydrochloride
^{13}C NMR (400 MHz, Acetic Acid-d_4)
169.03
127.31
125.41
51.45
49.62
43.71
42.21
39.63
21.97
13.50
11.67
f1 (ppm)

8. X-Ray Crystallography

Crystal Structure of 37

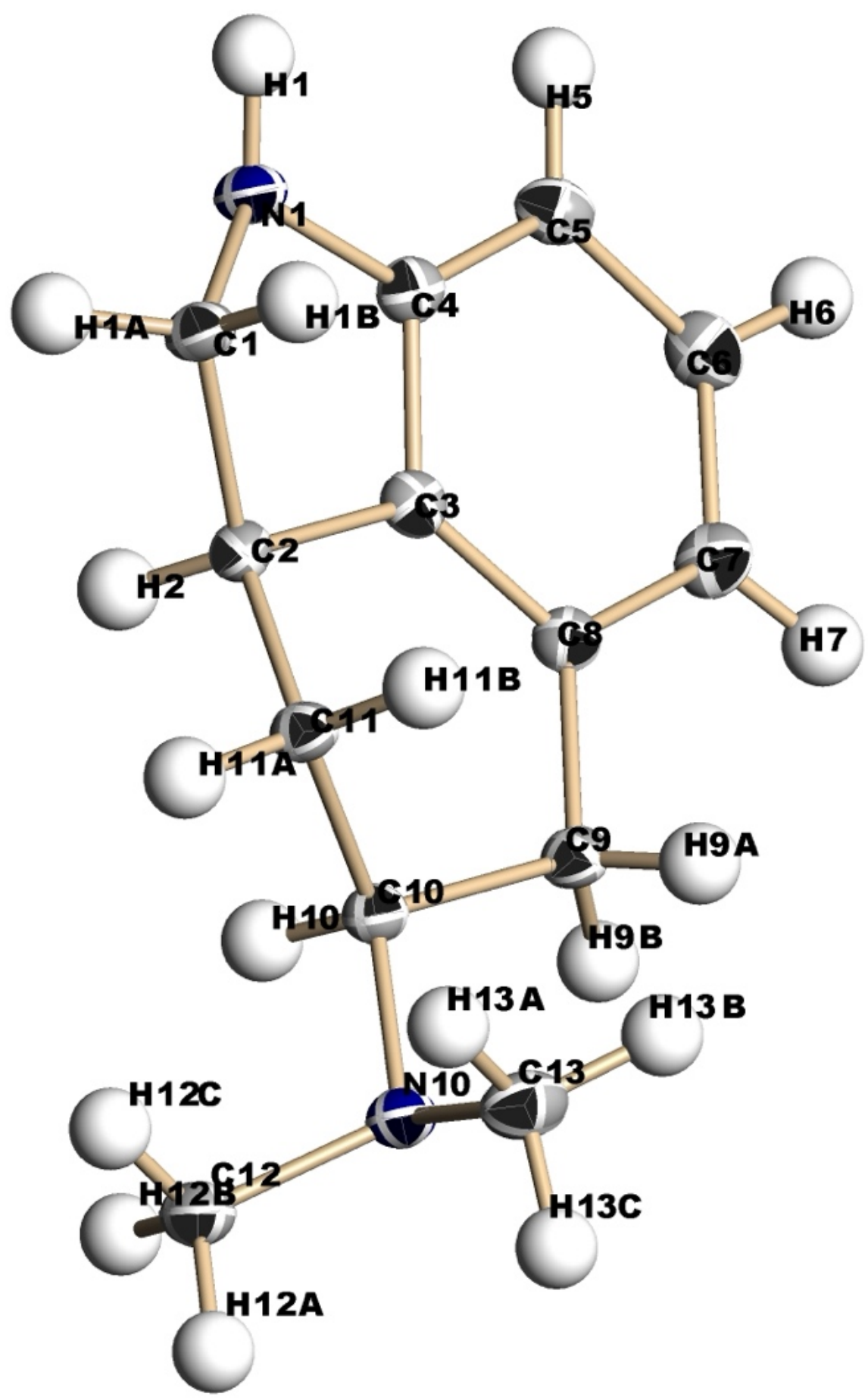

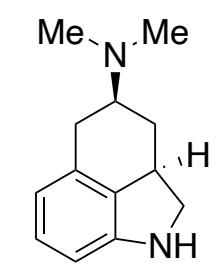

37

Preparation of 37: Purified **37** was dissolved in THF, filter paper was used as cover for the vial and the solvent was allowed to slowly evaporate at ambient temperature until light red/brown crystals formed along the bottom of the vial.

JF3128 Duo Cu
A colorless plate with approximate orthogonal dimensions 0.079 x 0.238 x 0.460mm^3 was placed and optically centered on the Bruker Duo[18] APEXII CCD system at -183 °C (90 K). Indexing of the unit cell used a random set of reflections collected from three series of 0.5° wide ω-scans, 10 seconds per frame, and 30 frames per series that were well distributed in reciprocal space using MoKa radiation. Data were collected [CuKα] with 0.5° wide scans, variable time per frame

dependent upon detector 2θ angle and varying φ and omega angles such that nearly all unique reflections were collected at least once. The crystal to detector distance was 5.00cm, thus providing a complete sphere of data to θmax= 70.05°.

Structural determination and Refinement

All crystallographic calculations were performed on a Surface Pro7 with Intel i7-1065G7 at 1.30GHz with four cores, eight processors and 16GB of extended memory. Data collected were corrected for Lorentz and polarization effects with Saint[18] and absorption using Blessing's method and merged as incorporated with the program Sadabs.[19,20] The SHELXTL[21] program package was implemented to determine the probable space group and set up the initial files. System symmetry, systematic absences and intensity statistics indicated the noncentrosymmetric orthorhombic space group $P2_12_12_1$ (no. 19). The structure was determined by direct methods with the molecule being located using the program XT.[22] The structure was refined with XL.[23] The 8615 data collected were merged based upon identical indices to 4869, then merged for least squares refinement to 2080 unique data [R(int)=0.0233]. All nonhydrogen atoms were refined anisotropically. Hydrogen atoms were initially idealized and then allowed to refine freely throughout the final refinement stages. The final structure was refined to convergence with R(F)=2.79%, wR(F^2)=7.12%, GOF=1.049 for all 2080 unique reflections [R(F)=2.71%, wR(F^2)=7.06% for those 2031 data with Fo > 4σ(Fo)]. The final difference-Fourier map was featureless indicating that the structure is both correct and complete. An empirical correction for extinction was also attempted and found to be 0.0029(6) and applied. The structure's absolute structure parameters were determined to be: Flack(x),[24] 0.02(16); Hooft(y),[25] 0.02(14) and the Parsons(z)6, 0.02(15) indicating that the structure's absolute configuration has been determined reliably; these values would be close to
1.0 if the structure were inverted. In this case the absolute configuration is quite difficult due to the heaviest atoms being 2 nitrogen atoms.

Crystal data and structure refinement for [$C_{13}H_{18}N_2$] (37).

Identification code	JF3128FMI (AGB-F-136-3-D2)	
Empirical formula	C13 H18 N2	
Formula weight	202.29	
Temperature	90(2) K	
Wavelength	1.54178 Å	
Crystal system	Orthorhombic	
Space group	$P2_12_12_1$	
Unit cell dimensions	a = 8.0578(5) Å	α= 90°.
	b = 8.9267(5) Å	β= 90°.
	c = 15.4431(9) Å	γ = 90°.
Volume	1110.82(11) $Å^3$	
Z	4	
Density (calculated)	1.210 Mg/m^3	
Absorption coefficient	0.550 mm^{-1}	
F(000)	440	
Crystal size	0.460 x 0.238 x 0.079 mm^3	

Crystal color and habit	Colorless Plate
Diffractometer	Bruker APEX-II CCD
Theta range for data collection	5.725 to 70.053°.
Index ranges	-9<=h<=8, -10<=k<=10, -18<=l<=18
Reflections collected	4869
Independent reflections	2080 [R(int) = 0.0233]
Observed reflections (I > 2sigma(I))	2031
Completeness to theta = 67.679°	98.5 %
Absorption correction	Semi-empirical from equivalents
Max. and min. transmission	0.9009 and 0.8127
Solution method	SHELXT (Sheldrick, 2014)
Refinement method	SHELXL-2018/3 (Sheldrick, 2018) Full-matrix least-squares on F^2
Data / restraints / parameters	2080 / 0 / 209
Goodness-of-fit on F^2	1.049
Final R indices [I>2sigma(I)]	R1 = 0.0271, wR2 = 0.0706
R indices (all data)	R1 = 0.0279, wR2 = 0.0712
Absolute structure parameter	0.03(16)
Extinction coefficient	0.0029(6)
Largest diff. peak and hole	0.208 and -0.136 e.Å^{-3}

Atomic coordinates (x 10^4) and equivalent isotropic displacement parameters (Å^{2}x 10^3) for JF3128FMI. U(eq) is defined as one third of the trace of the orthogonalized U^{ij} tensor.

	x	y	z	U(eq)
C(1)	5249(2)	1142(2)	6020(1)	21(1)
N(1)	5595(2)	224(1)	5242(1)	21(1)
C(2)	5805(2)	2711(2)	5727(1)	18(1)
C(3)	5417(2)	2629(2)	4779(1)	18(1)
C(4)	5337(2)	1131(2)	4520(1)	19(1)
C(5)	5031(2)	785(2)	3659(1)	22(1)
C(6)	4752(2)	1985(2)	3090(1)	24(1)
C(7)	4736(2)	3467(2)	3367(1)	23(1)
C(8)	5061(2)	3809(2)	4237(1)	19(1)
C(9)	4900(2)	5363(2)	4630(1)	20(1)
C(10)	5604(2)	5451(2)	5556(1)	18(1)

N(10)	5261(2)	6941(1)	5936(1)	20(1)
C(11)	5031(2)	4108(2)	6119(1)	19(1)
C(12)	6438(2)	7280(2)	6630(1)	24(1)
C(13)	3559(2)	7108(2)	6251(1)	27(1)

Bond lengths [Å] and angles [°] for JF3128FMI.

C(1)-N(1)	1.4810(18)
C(1)-C(2)	1.5384(19)
C(1)-H(1A)	1.00(2)
C(1)-H(1B)	1.00(2)
N(1)-C(4)	1.3936(19)
N(1)-H(1)	0.90(2)
C(2)-C(3)	1.499(2)
C(2)-C(11)	1.520(2)
C(2)-H(2)	0.954(19)
C(3)-C(8)	1.376(2)
C(3)-C(4)	1.398(2)
C(4)-C(5)	1.387(2)
C(5)-C(6)	1.403(2)
C(5)-H(5)	0.944(19)
C(6)-C(7)	1.390(2)
C(6)-H(6)	0.98(2)
C(7)-C(8)	1.402(2)
C(7)-H(7)	0.94(2)
C(8)-C(9)	1.5196(18)
C(9)-C(10)	1.540(2)
C(9)-H(9A)	1.01(2)
C(9)-H(9B)	0.98(2)
C(10)-N(10)	1.4804(18)
C(10)-C(11)	1.5512(18)
C(10)-H(10)	0.986(18)
N(10)-C(13)	1.462(2)
N(10)-C(12)	1.463(2)
C(11)-H(11A)	0.99(2)
C(11)-H(11B)	1.03(2)

C(12)-H(12A)	0.98(2)
C(12)-H(12B)	0.99(2)
C(12)-H(12C)	0.96(2)
C(13)-H(13A)	1.02(2)
C(13)-H(13B)	1.01(2)
C(13)-H(13C)	0.99(2)

N(1)-C(1)-C(2)	102.17(12)
N(1)-C(1)-H(1A)	111.3(12)
C(2)-C(1)-H(1A)	112.4(12)
N(1)-C(1)-H(1B)	109.8(11)
C(2)-C(1)-H(1B)	110.5(11)
H(1A)-C(1)-H(1B)	110.4(17)
C(4)-N(1)-C(1)	107.41(11)
C(4)-N(1)-H(1)	120.3(14)
C(1)-N(1)-H(1)	117.5(14)
C(3)-C(2)-C(11)	110.11(12)
C(3)-C(2)-C(1)	100.49(12)
C(11)-C(2)-C(1)	120.68(13)
C(3)-C(2)-H(2)	109.4(12)
C(11)-C(2)-H(2)	108.9(11)
C(1)-C(2)-H(2)	106.6(11)
C(8)-C(3)-C(4)	123.28(14)
C(8)-C(3)-C(2)	126.92(13)
C(4)-C(3)-C(2)	109.63(13)
C(5)-C(4)-N(1)	131.62(13)
C(5)-C(4)-C(3)	119.72(14)
N(1)-C(4)-C(3)	108.67(13)
C(4)-C(5)-C(6)	117.29(13)
C(4)-C(5)-H(5)	121.3(11)
C(6)-C(5)-H(5)	121.4(11)
C(7)-C(6)-C(5)	122.37(14)
C(7)-C(6)-H(6)	118.5(11)
C(5)-C(6)-H(6)	119.0(11)
C(6)-C(7)-C(8)	120.03(14)
C(6)-C(7)-H(7)	119.2(13)

C(8)-C(7)-H(7)	120.7(13)
C(3)-C(8)-C(7)	117.05(13)
C(3)-C(8)-C(9)	118.26(13)
C(7)-C(8)-C(9)	124.47(13)
C(8)-C(9)-C(10)	112.70(11)
C(8)-C(9)-H(9A)	108.4(12)
C(10)-C(9)-H(9A)	109.1(12)
C(8)-C(9)-H(9B)	109.7(12)
C(10)-C(9)-H(9B)	109.2(12)
H(9A)-C(9)-H(9B)	107.7(17)
N(10)-C(10)-C(9)	110.19(12)
N(10)-C(10)-C(11)	114.62(12)
C(9)-C(10)-C(11)	111.82(12)
N(10)-C(10)-H(10)	103.5(10)
C(9)-C(10)-H(10)	108.2(11)
C(11)-C(10)-H(10)	108.0(10)
C(13)-N(10)-C(12)	110.10(13)
C(13)-N(10)-C(10)	113.48(12)
C(12)-N(10)-C(10)	110.82(12)
C(2)-C(11)-C(10)	106.75(12)
C(2)-C(11)-H(11A)	111.0(11)
C(10)-C(11)-H(11A)	109.8(11)
C(2)-C(11)-H(11B)	110.6(10)
C(10)-C(11)-H(11B)	110.5(10)
H(11A)-C(11)-H(11B)	108.2(16)
N(10)-C(12)-H(12A)	111.2(13)
N(10)-C(12)-H(12B)	109.4(12)
H(12A)-C(12)-H(12B)	107.5(18)
N(10)-C(12)-H(12C)	112.8(13)
H(12A)-C(12)-H(12C)	108.9(17)
H(12B)-C(12)-H(12C)	106.9(18)
N(10)-C(13)-H(13A)	112.2(13)
N(10)-C(13)-H(13B)	109.8(12)
H(13A)-C(13)-H(13B)	110.3(18)
N(10)-C(13)-H(13C)	107.9(14)
H(13A)-C(13)-H(13C)	110.1(19)

H(13B)-C(13)-H(13C)	106.4(18)

Anisotropic displacement parameters ($Å^2$x 10^3) for JF3128FMI. The anisotropic displacement factor exponent takes the form: $-2\pi^2[h^2 a^{*2}U^{11} + ... + 2 h k a^* b^* U^{12}]$

	U^{11}	U^{22}	U^{33}	U^{23}	U^{13}	U^{12}
C(1)	29(1)	15(1)	19(1)	1(1)	-2(1)	0(1)
N(1)	27(1)	15(1)	20(1)	0(1)	0(1)	0(1)
C(2)	20(1)	17(1)	19(1)	2(1)	-2(1)	0(1)
C(3)	16(1)	21(1)	18(1)	0(1)	2(1)	-1(1)
C(4)	16(1)	19(1)	21(1)	0(1)	3(1)	1(1)
C(5)	22(1)	21(1)	22(1)	-5(1)	4(1)	-1(1)
C(6)	27(1)	30(1)	16(1)	-2(1)	3(1)	-2(1)
C(7)	27(1)	24(1)	18(1)	5(1)	4(1)	-2(1)
C(8)	18(1)	19(1)	18(1)	2(1)	2(1)	-2(1)
C(9)	24(1)	17(1)	20(1)	4(1)	0(1)	-2(1)
C(10)	19(1)	16(1)	20(1)	1(1)	0(1)	-1(1)
N(10)	22(1)	16(1)	22(1)	-1(1)	0(1)	0(1)
C(11)	24(1)	16(1)	17(1)	1(1)	0(1)	-1(1)
C(12)	31(1)	19(1)	24(1)	-1(1)	-3(1)	-1(1)
C(13)	27(1)	20(1)	34(1)	-2(1)	5(1)	2(1)

Hydrogen coordinates (x 10^4) and isotropic displacement parameters ($Å^2$x 10^3) for JF3128FMI.

	x	y	z	U(eq)
H(1A)	5890(30)	780(20)	6531(14)	26(5)
H(1B)	4040(20)	1130(20)	6146(12)	19(4)
H(1)	5240(30)	-730(30)	5243(14)	32(5)
H(2)	6980(20)	2750(20)	5806(12)	15(4)
H(5)	4990(30)	-220(20)	3467(12)	18(4)
H(6)	4470(30)	1770(20)	2487(13)	23(5)
H(7)	4470(30)	4230(30)	2967(14)	34(5)

H(9A)	3680(30)	5640(20)	4643(13)	27(5)
H(9B)	5470(30)	6100(20)	4263(13)	24(5)
H(10)	6820(20)	5421(19)	5520(11)	14(4)
H(11A)	5400(30)	4250(20)	6724(13)	19(4)
H(11B)	3760(20)	4030(20)	6119(12)	16(4)
H(12A)	6250(30)	8280(20)	6865(14)	26(5)
H(12B)	7590(30)	7250(20)	6395(14)	31(5)
H(12C)	6400(30)	6570(20)	7096(14)	28(5)
H(13A)	3340(30)	6470(30)	6784(15)	32(6)
H(13B)	2750(30)	6850(20)	5775(15)	29(5)
H(13C)	3380(30)	8180(30)	6386(15)	38(6)

Torsion angles [°] for JF3128FMI.

C(2)-C(1)-N(1)-C(4)	-32.86(16)
N(1)-C(1)-C(2)-C(3)	31.62(15)
N(1)-C(1)-C(2)-C(11)	152.71(13)
C(11)-C(2)-C(3)-C(8)	25.5(2)
C(1)-C(2)-C(3)-C(8)	153.86(15)
C(11)-C(2)-C(3)-C(4)	-149.81(12)
C(1)-C(2)-C(3)-C(4)	-21.46(16)
C(1)-N(1)-C(4)-C(5)	-159.62(17)
C(1)-N(1)-C(4)-C(3)	20.10(17)
C(8)-C(3)-C(4)-C(5)	6.0(2)
C(2)-C(3)-C(4)-C(5)	-178.44(14)
C(8)-C(3)-C(4)-N(1)	-173.72(14)
C(2)-C(3)-C(4)-N(1)	1.80(17)
N(1)-C(4)-C(5)-C(6)	177.31(16)
C(3)-C(4)-C(5)-C(6)	-2.4(2)
C(4)-C(5)-C(6)-C(7)	-1.6(2)
C(5)-C(6)-C(7)-C(8)	2.2(3)
C(4)-C(3)-C(8)-C(7)	-5.3(2)
C(2)-C(3)-C(8)-C(7)	179.97(16)
C(4)-C(3)-C(8)-C(9)	169.52(14)
C(2)-C(3)-C(8)-C(9)	-5.2(2)
C(6)-C(7)-C(8)-C(3)	1.2(2)

C(6)-C(7)-C(8)-C(9)	-173.28(15)
C(3)-C(8)-C(9)-C(10)	14.84(19)
C(7)-C(8)-C(9)-C(10)	-170.76(14)
C(8)-C(9)-C(10)-N(10)	-174.32(12)
C(8)-C(9)-C(10)-C(11)	-45.61(17)
C(9)-C(10)-N(10)-C(13)	78.67(16)
C(11)-C(10)-N(10)-C(13)	-48.50(18)
C(9)-C(10)-N(10)-C(12)	-156.86(13)
C(11)-C(10)-N(10)-C(12)	75.97(16)
C(3)-C(2)-C(11)-C(10)	-52.51(16)
C(1)-C(2)-C(11)-C(10)	-168.79(13)
N(10)-C(10)-C(11)-C(2)	-168.11(12)
C(9)-C(10)-C(11)-C(2)	65.56(16)

Hydrogen bonds for JF3128FMI [Å and °].

D-H...A	d(D-H)	d(H...A)	d(D...A)	<(DHA)
C(9)-H(9A)...N(1)#1	1.01(2)	2.61(2)	3.513(2)	148.4(15)
N(1)-H(1)...N(10)#2	0.90(2)	2.34(2)	3.1319(17)	147.2(19)

Symmetry transformations used to generate equivalent atoms:

#1 x-1/2,-y+1/2,-z+1 #2 x,y-1,z

Crystal Structure of 41

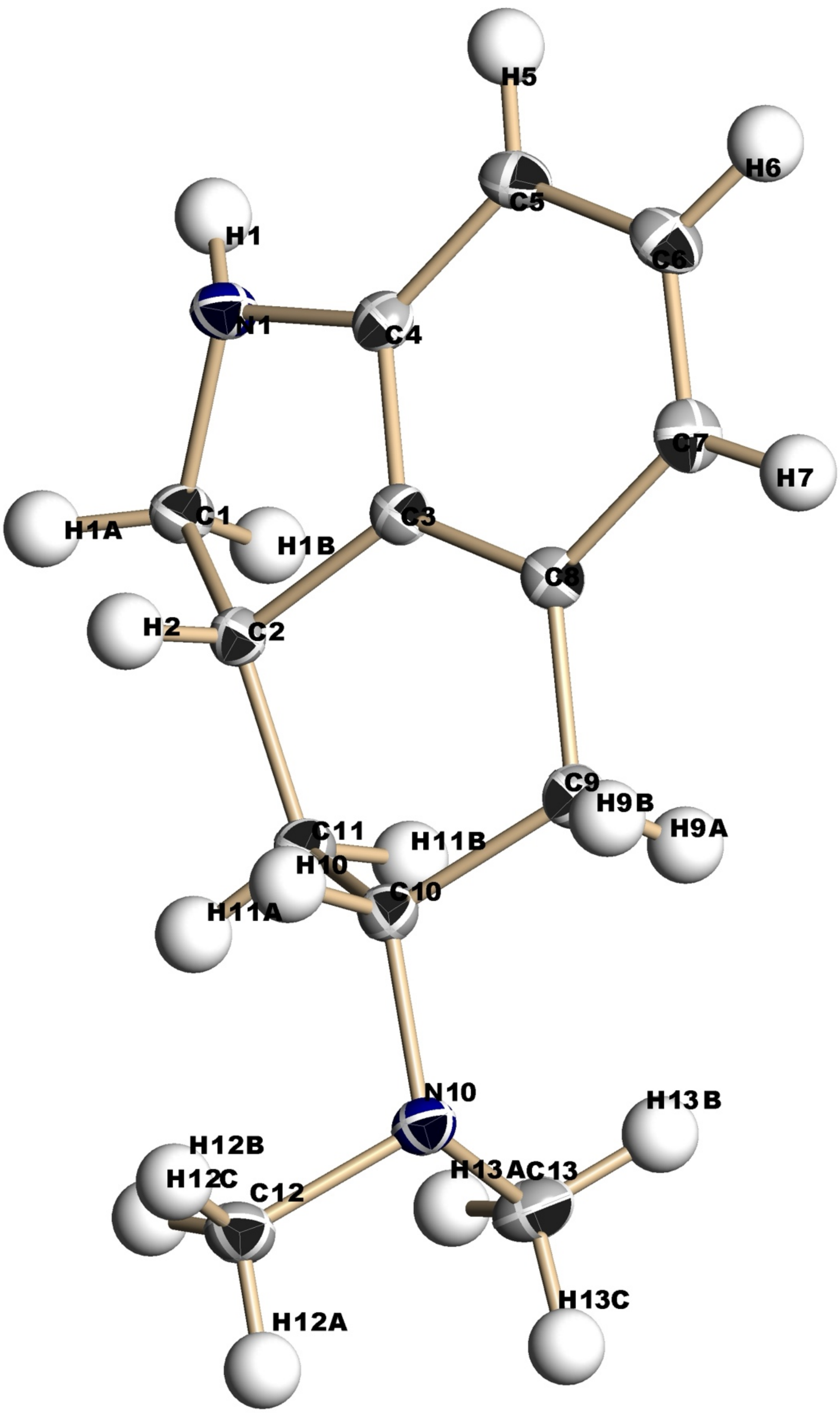

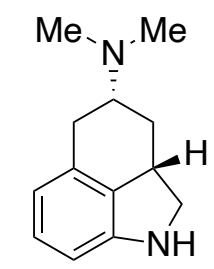

41

Preparation of 41: Purified **41** was dissolved in EtOAc, filter paper was used as cover for the vial and the solvent was allowed to slowly evaporate at ambient temperature until brown crystals formed along the bottom of the vial.

JF3129 APEX2 Mo (AGB-F-153-D1)
An orange block with approximate orthogonal dimensions 0.082 x 0.239 x 0.368mm^3 was placed and optically centered on the Bruker Duo[18] APEXII CCD system at -183°C(90K). Indexing of the unit cell used a random set of reflections collected from three series of 0.5° wide ω-scans, 10 seconds per frame, and 30 frames per series that were well distributed in reciprocal space. Five ω-scan data frame series were collected [MoK_α] with 0.3° wide scans, 15 seconds per frame and 606 frames collected per series at varying φ angles and plus/minus 32 degrees for the detector. The crystal to detector distance was 5.15cm, thus providing a complete sphere of data to $2\theta_{max}$=54.97°.

Structural determination and Refinement:
All crystallographic calculations were performed on a Surface Pro7 with Intel i7-1065G7 at 1.30GHz with four cores, eight processors and 16GB of extended memory. Data collected were corrected for Lorentz and polarization effects with Saint[18] and absorption using Blessing's method and merged as incorporated with the program Sadabs.[19,20] The SHELXTL[21] program package was implemented to determine the probable space group and set up the initial files. System symmetry, systematic absences and intensity statistics indicated the non-centrosymmetric orthorhombic space group $P2_12_12_1$ (no. 19). The structure was determined by direct methods with the molecule being located using the program XT.[22] Found to be identical but inverted form of AGB-F-136-3-D2. The structure was refined with XL.[23] The 22280 data collected were merged based upon identical indices to 9900, then merged for least squares refinement to 2564 unique data [R(int)=0.0283]. All non-hydrogen atoms were refined anisotropically. Hydrogen atoms were initially idealized then allowed to refine freely throughout the final refinement stages. The final structure was refined to convergence with R(F)=3.48%, wR(F^2)=8.24%, GOF=1.063 for all 2564 unique reflections [R(F)=3.21%, wR(F^2)=8.01% for those 2401 data with Fo > 4σ(Fo)]. The final difference-Fourier map was featureless indicating that the structure is both correct and complete. An empirical correction for extinction was also attempted and found to be negative and therefore not applied. The structure's absolute structure parameters were determined to be: Flack(x),[24] 0.1(9); Hooft(y),[25] 0.3(8) and the Parsons(z),[23] 0.6(9) indicating that the structure's absolute configuration has been determined reliably; these values would be close to 1.0 if the structure were inverted. Wrong inverted model yields: Flack(x),[24] 0.9(9); Hooft(y),[25] 0.7(8) and the Parsons(z),[23] 0.4(9).

Crystal data and structure refinement for [$C_{13}H_{18}N_2$] (41).

Identification code	JF3129FMI
Empirical formula	C13 H18 N2 (AGB-F-153-D1)
Formula weight	202.29
Temperature	90(2) K
Wavelength	0.71073 Å

Crystal system	Orthorhombic
Space group	$P2_12_12_1$
Unit cell dimensions	a = 8.0520(3) Å α= 90°.
	b = 8.9307(3) Å β= 90°.
	c = 15.4510(5) Å γ = 90°.
Volume	1111.08(7) Å^3
Z	4
Density (calculated)	1.209 Mg/m^3
Absorption coefficient	0.072 mm^{-1}
F(000)	440
Crystal size	0.368 x 0.239 x 0.082 mm^3
Crystal color and habit	Orange Block
Diffractometer	Bruker APEX-II CCD
Theta range for data collection	2.634 to 27.523°.
Index ranges	-10<=h<=10, -11<=k<=11, -19<=l<=20
Reflections collected	9900
Independent reflections	2564 [R(int) = 0.0283]
Observed reflections (I > 2sigma(I))	2401
Completeness to theta = 25.242°	100.0 %
Absorption correction	Semi-empirical from equivalents
Max. and min. transmission	0.9881 and 0.9071
Solution method	SHELXT (Sheldrick, 2014)
Refinement method	SHELXL-2018/3 (Sheldrick, 2018) Full-matrix least-squares on F^2
Data / restraints / parameters	2564 / 0 / 208
Goodness-of-fit on F^2	1.063
Final R indices [I>2sigma(I)]	R1 = 0.0321, wR2 = 0.0801
R indices (all data)	R1 = 0.0348, wR2 = 0.0824
Absolute structure parameter	0.1(9)
Largest diff. peak and hole	0.202 and -0.202 e.Å^{-3}

Atomic coordinates (x 10^4) and equivalent isotropic displacement parameters (Å^{2}x 10^3) for JF3129FMI. U(eq) is defined as one third of the trace of the orthogonalized U^{ij} tensor.

	x	y	z	U(eq)
C(1)	4753(2)	8859(2)	3980(1)	20(1)

N(1)	4406(2)	9778(2)	4759(1)	20(1)
C(2)	4195(2)	7290(2)	4272(1)	17(1)
C(3)	4583(2)	7369(2)	5221(1)	17(1)
C(4)	4665(2)	8868(2)	5480(1)	18(1)
C(5)	4971(2)	9213(2)	6342(1)	21(1)
C(6)	5246(2)	8013(2)	6911(1)	23(1)
C(7)	5262(2)	6533(2)	6633(1)	22(1)
C(8)	4936(2)	6188(2)	5760(1)	18(1)
C(9)	5097(2)	4637(2)	5370(1)	19(1)
C(10)	4399(2)	4551(2)	4442(1)	17(1)
N(10)	4736(2)	3060(1)	4063(1)	19(1)
C(11)	4967(2)	5893(2)	3881(1)	18(1)
C(12)	3558(2)	2721(2)	3369(1)	23(1)
C(13)	6439(2)	2891(2)	3748(1)	26(1)

Bond lengths [Å] and angles [°] for JF3129FMI.

C(1)-N(1)	1.484(2)
C(1)-C(2)	1.539(2)
C(1)-H(1A)	0.99(2)
C(1)-H(1B)	0.99(2)
N(1)-C(4)	1.394(2)
N(1)-H(1)	0.90(2)
C(2)-C(3)	1.501(2)
C(2)-C(11)	1.519(2)
C(2)-H(2)	0.971(18)
C(3)-C(8)	1.373(2)
C(3)-C(4)	1.399(2)
C(4)-C(5)	1.389(2)
C(5)-C(6)	1.404(2)
C(5)-H(5)	0.961(19)
C(6)-C(7)	1.390(2)
C(6)-H(6)	0.99(2)
C(7)-C(8)	1.408(2)
C(7)-H(7)	0.96(2)
C(8)-C(9)	1.516(2)

C(9)-C(10)	1.542(2)
C(9)-H(9A)	0.99(2)
C(9)-H(9B)	0.992(19)
C(10)-N(10)	1.480(2)
C(10)-C(11)	1.549(2)
C(10)-H(10)	0.973(18)
N(10)-C(13)	1.462(2)
N(10)-C(12)	1.463(2)
C(11)-H(11A)	1.00(2)
C(11)-H(11B)	1.01(2)
C(12)-H(12A)	0.96(2)
C(12)-H(12B)	0.98(2)
C(12)-H(12C)	0.97(2)
C(13)-H(13A)	1.03(2)
C(13)-H(13B)	1.02(2)
C(13)-H(13C)	0.99(2)
N(1)-C(1)-C(2)	102.13(12)
N(1)-C(1)-H(1A)	111.3(12)
C(2)-C(1)-H(1A)	112.8(12)
N(1)-C(1)-H(1B)	109.3(12)
C(2)-C(1)-H(1B)	110.9(12)
H(1A)-C(1)-H(1B)	110.0(17)
C(4)-N(1)-C(1)	107.33(12)
C(4)-N(1)-H(1)	122.2(15)
C(1)-N(1)-H(1)	116.0(14)
C(3)-C(2)-C(11)	110.01(13)
C(3)-C(2)-C(1)	100.56(13)
C(11)-C(2)-C(1)	120.73(13)
C(3)-C(2)-H(2)	108.7(11)
C(11)-C(2)-H(2)	109.7(12)
C(1)-C(2)-H(2)	106.3(11)
C(8)-C(3)-C(4)	123.50(14)
C(8)-C(3)-C(2)	126.83(14)
C(4)-C(3)-C(2)	109.50(13)
C(5)-C(4)-N(1)	131.55(14)

C(5)-C(4)-C(3)	119.64(14)
N(1)-C(4)-C(3)	108.82(13)
C(4)-C(5)-C(6)	117.37(14)
C(4)-C(5)-H(5)	121.0(11)
C(6)-C(5)-H(5)	121.6(11)
C(7)-C(6)-C(5)	122.23(15)
C(7)-C(6)-H(6)	119.5(11)
C(5)-C(6)-H(6)	118.1(11)
C(6)-C(7)-C(8)	120.16(15)
C(6)-C(7)-H(7)	119.6(12)
C(8)-C(7)-H(7)	120.2(12)
C(3)-C(8)-C(7)	116.83(14)
C(3)-C(8)-C(9)	118.57(14)
C(7)-C(8)-C(9)	124.37(14)
C(8)-C(9)-C(10)	112.57(12)
C(8)-C(9)-H(9A)	108.3(12)
C(10)-C(9)-H(9A)	109.0(12)
C(8)-C(9)-H(9B)	108.9(11)
C(10)-C(9)-H(9B)	110.6(11)
H(9A)-C(9)-H(9B)	107.3(16)
N(10)-C(10)-C(9)	110.24(12)
N(10)-C(10)-C(11)	114.85(12)
C(9)-C(10)-C(11)	112.00(12)
N(10)-C(10)-H(10)	104.5(11)
C(9)-C(10)-H(10)	107.9(11)
C(11)-C(10)-H(10)	106.9(10)
C(13)-N(10)-C(12)	110.00(14)
C(13)-N(10)-C(10)	113.41(12)
C(12)-N(10)-C(10)	110.91(13)
C(2)-C(11)-C(10)	106.97(12)
C(2)-C(11)-H(11A)	110.8(12)
C(10)-C(11)-H(11A)	110.0(11)
C(2)-C(11)-H(11B)	111.0(11)
C(10)-C(11)-H(11B)	109.8(11)
H(11A)-C(11)-H(11B)	108.3(17)
N(10)-C(12)-H(12A)	111.3(13)

N(10)-C(12)-H(12B)	108.8(12)
H(12A)-C(12)-H(12B)	108.5(18)
N(10)-C(12)-H(12C)	112.9(12)
H(12A)-C(12)-H(12C)	107.5(17)
H(12B)-C(12)-H(12C)	107.8(17)
N(10)-C(13)-H(13A)	111.8(13)
N(10)-C(13)-H(13B)	110.3(12)
H(13A)-C(13)-H(13B)	109.2(18)
N(10)-C(13)-H(13C)	108.2(14)
H(13A)-C(13)-H(13C)	109.0(18)
H(13B)-C(13)-H(13C)	108.3(18)

Anisotropic displacement parameters (Å^{2}x 10^3) for JF3129FMI. The anisotropic displacement factor exponent takes the form: $-2\pi^2[h^2 a^{*2}U^{11} + ... + 2 h k a^* b^* U^{12}]$

	U^{11}	U^{22}	U^{33}	U^{23}	U^{13}	U^{12}
C(1)	27(1)	15(1)	18(1)	1(1)	-1(1)	0(1)
N(1)	26(1)	16(1)	18(1)	-1(1)	0(1)	0(1)
C(2)	19(1)	16(1)	17(1)	1(1)	-1(1)	0(1)
C(3)	14(1)	19(1)	18(1)	0(1)	2(1)	-1(1)
C(4)	15(1)	19(1)	19(1)	0(1)	2(1)	1(1)
C(5)	22(1)	21(1)	20(1)	-4(1)	3(1)	-1(1)
C(6)	25(1)	30(1)	15(1)	-3(1)	3(1)	-2(1)
C(7)	25(1)	24(1)	16(1)	5(1)	3(1)	-2(1)
C(8)	16(1)	19(1)	18(1)	1(1)	2(1)	-2(1)
C(9)	21(1)	17(1)	18(1)	3(1)	1(1)	-2(1)
C(10)	17(1)	15(1)	18(1)	0(1)	0(1)	-1(1)
N(10)	20(1)	16(1)	21(1)	-1(1)	0(1)	1(1)
C(11)	22(1)	15(1)	16(1)	1(1)	0(1)	-1(1)
C(12)	29(1)	18(1)	22(1)	-1(1)	-2(1)	-2(1)
C(13)	25(1)	20(1)	34(1)	-3(1)	5(1)	1(1)

Hydrogen coordinates (x 10^4) and isotropic displacement parameters (Å^{2}x 10^3) for JF3129FMI.

	x	y	z	U(eq)
H(1A)	4130(30)	9230(20)	3469(13)	23(5)
H(1B)	5960(30)	8880(20)	3857(13)	23(5)
H(1)	4760(30)	10730(30)	4740(14)	34(6)
H(2)	3000(20)	7260(20)	4199(12)	16(4)
H(5)	5020(30)	10240(20)	6535(12)	20(5)
H(6)	5530(20)	8250(20)	7517(13)	24(5)
H(7)	5530(30)	5750(20)	7035(13)	27(5)
H(9A)	6290(30)	4370(20)	5360(13)	24(5)
H(9B)	4530(20)	3910(20)	5752(12)	19(4)
H(10)	3190(20)	4604(19)	4479(11)	13(4)
H(11A)	4590(30)	5750(20)	3272(13)	20(4)
H(11B)	6220(30)	5960(20)	3883(12)	21(5)
H(12A)	3750(30)	1740(20)	3135(13)	24(5)
H(12B)	2430(30)	2760(20)	3604(13)	25(5)
H(12C)	3620(30)	3430(20)	2894(13)	22(5)
H(13A)	6670(30)	3560(30)	3216(16)	36(6)
H(13B)	7260(30)	3150(30)	4225(15)	33(6)
H(13C)	6600(30)	1840(30)	3582(14)	34(6)

Torsion angles [°] for JF3129FMI.

C(2)-C(1)-N(1)-C(4)	32.87(16)
N(1)-C(1)-C(2)-C(3)	-31.65(15)
N(1)-C(1)-C(2)-C(11)	-152.70(14)
C(11)-C(2)-C(3)-C(8)	-25.5(2)
C(1)-C(2)-C(3)-C(8)	-153.86(15)
C(11)-C(2)-C(3)-C(4)	149.89(13)
C(1)-C(2)-C(3)-C(4)	21.49(17)
C(1)-N(1)-C(4)-C(5)	159.61(18)
C(1)-N(1)-C(4)-C(3)	-20.11(17)
C(8)-C(3)-C(4)-C(5)	-6.0(2)
C(2)-C(3)-C(4)-C(5)	178.46(14)
C(8)-C(3)-C(4)-N(1)	173.76(15)

C(2)-C(3)-C(4)-N(1)	-1.78(17)
N(1)-C(4)-C(5)-C(6)	-177.45(16)
C(3)-C(4)-C(5)-C(6)	2.2(2)
C(4)-C(5)-C(6)-C(7)	1.8(3)
C(5)-C(6)-C(7)-C(8)	-2.4(3)
C(4)-C(3)-C(8)-C(7)	5.3(2)
C(2)-C(3)-C(8)-C(7)	-179.96(16)
C(4)-C(3)-C(8)-C(9)	-169.48(15)
C(2)-C(3)-C(8)-C(9)	5.3(2)
C(6)-C(7)-C(8)-C(3)	-1.1(2)
C(6)-C(7)-C(8)-C(9)	173.35(16)
C(3)-C(8)-C(9)-C(10)	-14.7(2)
C(7)-C(8)-C(9)-C(10)	170.97(15)
C(8)-C(9)-C(10)-N(10)	174.43(12)
C(8)-C(9)-C(10)-C(11)	45.24(18)
C(9)-C(10)-N(10)-C(13)	-78.99(16)
C(11)-C(10)-N(10)-C(13)	48.64(18)
C(9)-C(10)-N(10)-C(12)	156.65(13)
C(11)-C(10)-N(10)-C(12)	-75.72(17)
C(3)-C(2)-C(11)-C(10)	52.31(16)
C(1)-C(2)-C(11)-C(10)	168.63(13)
N(10)-C(10)-C(11)-C(2)	167.99(12)
C(9)-C(10)-C(11)-C(2)	-65.27(17)

Hydrogen bonds for JF3129FMI [Å and °].

D-H...A	d(D-H)	d(H...A)	d(D...A)	<(DHA)
N(1)-H(1)...N(10)#1	0.90(2)	2.33(2)	3.1335(18)	149(2)
C(9)-H(9A)...N(1)#2	0.99(2)	2.63(2)	3.515(2)	148.7(15)

Symmetry transformations used to generate equivalent atoms:

#1 x,y+1,z #2 x+1/2,-y+3/2,-z+1

9. Pharmacokinetic studies of UCD0120 and UCD0179

Table S6. Parameters of MS/MS analysis, Chromatograph peaks and retention times for target analytes.

Analytes	Peak Shape	Retention time	Precursor	Product Ion	Ratio[c]	DP (volts)	CE (volts)	CXP (volts)
DMT-D4	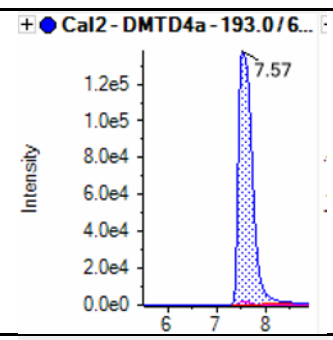	7.57	193	60[a]	>1,000	16	17	18
				148 (negligible)[b]		16	15	8
UCD179	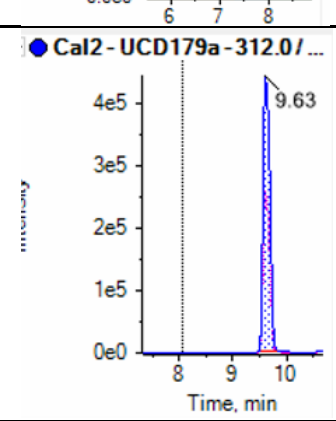	9.63	312	211[a]	1.7	41	29	10
				269[b]		41	25	34
UCD120	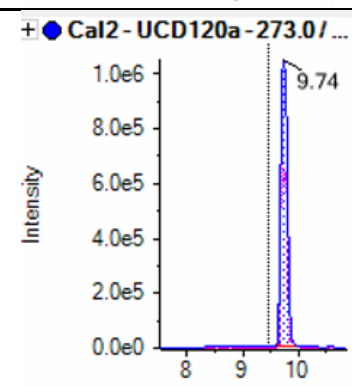	9.74	273	230[a]	1.7	51	23	32
				172[b]		51	25	18

Note:
[a] quantifier
[b] qualifier
[c] The ratio of quantifier and qualifier. The ratio between the quantifier and qualifier ions can vary depending on the instrument and the specific parameter settings used.

Table S7. Examples of calibration curves and detection limit

Analyte	Calibration Curve	R^2	Instrumental detection limit (µg/kg)[a]
**UCD0179**	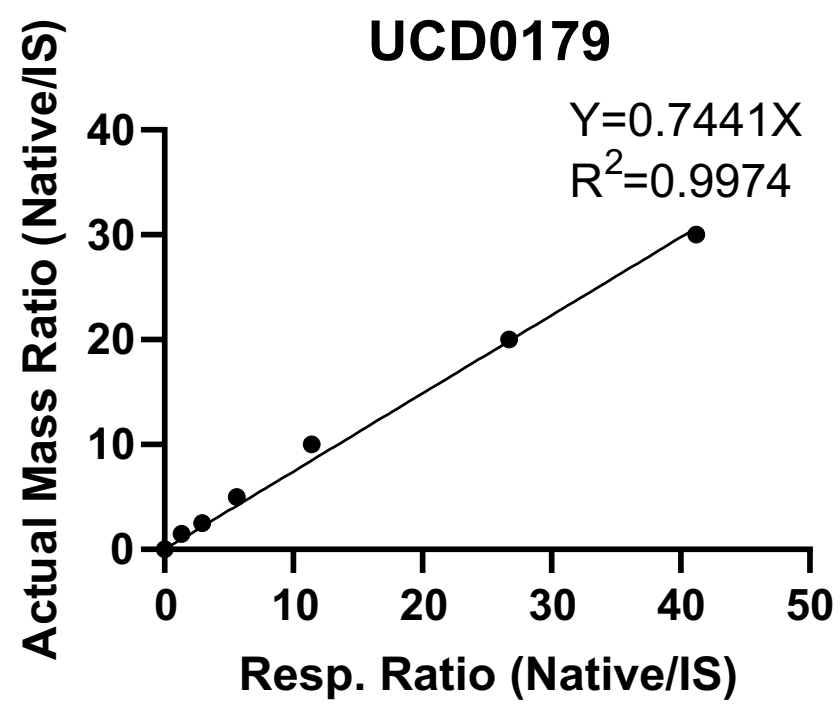	0.9974	7.5
**UCD0120**	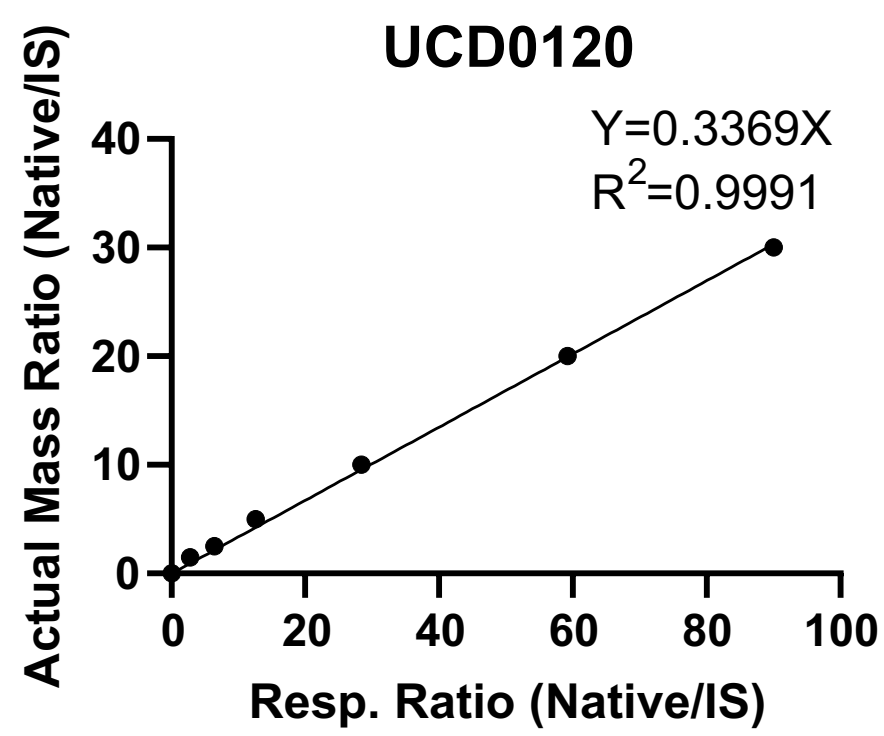	0.9991	7.5

[a] The instrumental detection limit was estimated by serially diluting each analyte and defining the lowest concentration at which a chromatographic peak could still be visually distinguished from background noise.

10. Supplemental References

1. Hanwell, M. D.; Curtis, D. E.; Lonie, D. C.; Vandermeersch, T.; Zurek, E.; Hutchison, G. R. Avogadro: An Advanced Semantic Chemical Editor, Visualization, and Analysis Platform. *J. Cheminformatics* **2012**, *4*, 17. https://doi.org/10.1186/1758-2946-4-17.
2. Halgren, T. A. Merck Molecular Force Field. I. Basis, Form, Scope, Parameterization, and Performance of MMFF94. *J. Comput. Chem.* **1996**, *17* (5–6), 490–519. https://doi.org/10.1002/(SICI)1096-987X(199604)17:5/6%253C490::AID-JCC1%253E3.0.CO;2-P
3. Bannwarth, C.; Ehlert, S.; Grimme, S. GFN2-xTB—An Accurate and Broadly Parametrized Self-Consistent Tight-Binding Quantum Chemical Method with Multipole Electrostatics and Density-Dependent Dispersion Contributions. *J. Chem. Theory Comput.* **2019**, *15* (3), 1652–1671. https://doi.org/10.1021/acs.jctc.8b01176.
4. Pracht, P.; Bohle, F.; Grimme, S. Automated Exploration of the Low-Energy Chemical Space with Fast Quantum Chemical Methods. *Phys. Chem. Chem. Phys.* **2020**, *22* (14), 7169–7192. https://doi.org/10.1039/C9CP06869D.
5. Ehlert, S.; Stahn, M.; Spicher, S.; Grimme, S. Robust and Efficient Implicit Solvation Model for Fast Semiempirical Methods. *J. Chem. Theory Comput*. **17**, 4250–4261 (2021).
6. The PyMOL Molecular Graphics System, Version 3.0 Schrödinger, LLC.
7. El-Hachem, N.; Haibe-Kains, B.; Khalil, A.; Kobeissy, F. H.; Nemer, G. AutoDock and AutoDockTools for Protein-Ligand Docking: Beta-Site Amyloid Precursor Protein Cleaving Enzyme 1(BACE1) as a Case Study. *Methods Mol. Biol.* **2017**, *1598*, 391–403. https://doi.org/10.1007/978-1-4939-6952-4_20
8. Trott, O.; Olson, A. J. AutoDock Vina: Improving the Speed and Accuracy of Docking with a New Scoring Function, Efficient Optimization, and Multithreading. *J. Comput. Chem.* **2010**, *31* (2), 455–461. https://doi.org/10.1002/jcc.21334.
9. Gumpper, R. H.; Jain, M. K.; Kim, K.; Sun, R.; Sun, N.; Xu, Z.; DiBerto, J. F.; Krumm, B. E.; Kapolka, N. J.; Kaniskan, H. Ü.; Nichols, D. E.; Jin, J.; Fay, J. F.; Roth, B. L. The Structural Diversity of Psychedelic Drug Actions Revealed. *Nat. Commun.* **2025**, *16* (1), 2734. https://doi.org/10.1038/s41467-025-57956-7.
10. Vargas, M. V., Dunlap, L. E., Dong, C., Carter, S. J., Tombari, R. J., Jami, S. A., Cameron, L. P., Patel, S. D., Hennessey, J. J., Saeger, H. N., McCorvy, J. D., Gray, J. A., Tian, L. & Olson, D. E. Psychedelics promote neuroplasticity through the activation of intracellular 5-HT2A receptors. *Science.* **379**, 700–706 (2023).
11. Bonniwell, E. M.; Alabdali, R.; Hennessey, J. J.; McKee, J. L.; Cavalco, N. G.; Lammers, J. C.; Moore, E. J.; Franchini, L.; Orlandi, C.; McCorvy, J. D. Serotonin 5-HT2C Receptor Signaling Analysis Reveals Psychedelic Biased Agonism. *ACS Chem. Neurosci.*, **16**, 3899–3914 (2025).
12. Halberstadt, A. L. Automated detection of the head-twitch response using wavelet scalograms and a deep convolutional neural network. *Sci. Rep.* **10**, 8344 (2020).
13. Hatzipantelis, C. J.; Cameron, L. P.; Liu, M.; Patel, S. D.; Saeger, H. N.; Vernier, A. M. M.; Khatib, Y. A.; Willis, B. J.; Lanoue, L.; Fiehn; O. F.; Olson, D. E. Indolethylamine N-Methyltransferase Deletion Impacts Mouse Behavior without Disrupting Endogenous Psychedelic Tryptamine Production. *ACS Chem. Neurosci.* **16**, 4011–4024 (2025).
14. Jabre, N. D.; Respondek, T.; Ulku, S. A.; Korostelova, N.; Kondanko, J. J. A Divergent Strategy for Attaching Polypyridyl Ligands to Peptides. *J. Org. Chem*. **2010**, 75, 650–659.
15. Tuck J. R; Dunlap, L. E.; Olson, D. E. Synthetic Strategies toward Lysergic Acid Diethylamide: Ergoline Synthesis via α-Arylation, Borrowing Hydrogen Alkylation, and C–H Insertion. *J. Org. Chem.* **2023**, 88, 19, 13712 – 13719

16. Crapster, J. A.; Guzei, I. A.; Blackwell, H. E. *Angew. Chem. Int.* **2013**, 52, 5079–5084
17. Siemon, T.; Steinhauer, S.; Christmann, M. Synthesis of (+)-Darwinolide, a Biofilm-Penetrating Anti-MRSA Agent. *Angew. Chem. Int. Ed.* **2019**, 58, 1120–1122.
18. Bruker (2019) APEXII (Version 2019.0) and (2016) SAINT (Version 8.37a). Bruker AXS Inc., Madison, Wisconsin, USA.
19. An Empirical Correction for Absorption Anisotropy, Blessing, R. H. (1995). Acta Cryst., A51, 33-38.
20. Sheldrick, G.M., SADABS (2016) Version 2016/2, 'Siemens Area Detector Absorption Correction' Universität Göttingen: Göttingen, Germany.
21. Sheldrick, G.M., (2002). SHELXTL. Version 6.1. Bruker AXS Inc., Madison, Wisconsin, USA.
22. Sheldrick, G. M., (2015) SHELXT – Integrated space-group and crystal-structure determination. Acta Crystallogr A Found Adv, 71(Pt 1), 3–8. doi: 10.1107/S2053273314026370
23. Sheldrick, G. M., (2018). SHELXL2018/3. Universität Göttingen: Göttingen, Germany.
24. On Enantiomorph-Polarity Estimation, Flack, H.D. (1983). Acta Cryst., A39, 876-881.
25. Hooft, R.W.W, Straver, L.H. & Spek, A.L. (2008). J. Appl, Cryst. 41, 96-103. Thompson,A.L. & Watkin, D.J. (2009). Tetrahedron
